# Effectiveness of Enhanced Performance Feedback on Appropriate Use of Blood Transfusions: A Comparison of 2 Cluster Randomized Trials

**DOI:** 10.1001/jamanetworkopen.2022.0364

**Published:** 2022-02-24

**Authors:** Simon J. Stanworth, Rebecca Walwyn, John Grant-Casey, Suzanne Hartley, Lauren Moreau, Fabiana Lorencatto, Jill Francis, Natalie Gould, Nick Swart, Megan Rowley, Steve Morris, Jeremy Grimshaw, Amanda Farrin, Robbie Foy

**Affiliations:** 1 NHS Blood and Transplant, John Radcliffe Hospital, Oxford, United Kingdom; 2 Oxford University Hospitals NHS Foundation Trust, Oxford, United Kingdom; 3 Radcliffe Department of Medicine and Oxford Biomedical Research Center Haematology Theme, University of Oxford, Oxford, United Kingdom; 4 Clinical Trials Research Unit, Leeds Institute for Clinical Trials Research, University of Leeds, Leeds, United Kingdom; 5 Division of Psychology and Language Sciences, University College London, London, United Kingdom; 6 School of Health Sciences City, University of London, London, United Kingdom; 7 School of Health Sciences, University of Melbourne, Melbourne, Victoria, Australia; 8 Department of Applied Health Research, University College London, London, United Kingdom; 9 Scottish National Blood Transfusion Service, Edinburgh, Edinburgh, United Kingdom; 10 Department of Public Health and Primary Care, University of Cambridge, Cambridge, United Kingdom; 11 Faculty of Medicine, University of Ottawa, Ontario, Canada; 12 Clinical Epidemiology Program, Ottawa Hospital Research Institute, Ottawa, Ontario, Canada; 13 Leeds Institute of Health Sciences, University of Leeds, Leeds, United Kingdom

## Abstract

**Importance:**

Auditing and feedback are frequently used to improve patient care. However, it remains unclear how to optimize feedback effectiveness for the appropriate use of treatments such as blood transfusion, a common but costly procedure that is more often overused than underused.

**Objective:**

To evaluate 2 theoretically informed feedback interventions to improve the appropriate use of blood transfusions.

**Design, Setting, and Participants:**

Two sequential, linked 2 × 2 cluster randomized trials were performed in hospitals in the UK participating in national audits of transfusion for perioperative anemia and management of hematological disorders. Data were collected for a surgical trial from October 1, 2014, to October 31, 2016, with follow-up completed on October 31, 2016. Data were collected for a hematological trial through follow-up from July 1, 2015, to June 30, 2017. Trial data were analyzed from November 1, 2016, to June 1, 2019.

**Interventions:**

Hospitals were randomized to standard content or enhanced content to improve feedback clarity and usability and to standard support or enhanced support for staff to act on feedback.

**Main Outcomes and Measures:**

The primary end point was appropriateness of transfusions audited at 12 months. Secondary end points included volume of transfusions (aiming for reductions at patient and cluster levels) and transfusion-related adverse events and reactions.

**Results:**

One hundred thirty-five of 152 eligible clusters participated in the surgical audit (2714 patients; mean [SD] age, 74.9 [14.0] years; 1809 women [66.7%]), and 134 of 141 participated in the hematological audit (4439 patients; median age, 72.0 [IQR, 64.0-80.0] years; 2641 men [59.5%]). Fifty-seven of 69 clusters (82.6%) in the surgical audit randomized to enhanced content downloaded reports compared with 52 of 66 clusters (78.8%) randomized to standard reports. Fifty-nine of 68 clusters (86.8%) randomized to enhanced support logged onto the toolkit. The proportion of patients with appropriate transfusions was 0.184 for standard content and 0.176 for enhanced content (adjusted odds ratio [OR], 0.91 [97.5% CI, 0.61-1.36]) and 0.181 for standard support and 0.180 for enhanced support (adjusted OR, 1.05 [97.5% CI, 0.68-1.61]). For the hematological audit, 53 of 66 clusters (80.3%) randomized to enhanced content downloaded the reports compared with 53 of 68 clusters (77.9%) randomized to standard content. Forty-nine of 67 clusters sites (73.1%) assigned to enhanced support logged into the toolkit at least once. The proportion of patients with appropriate transfusions was 0.744 for standard content and 0.714 for enhanced content (adjusted OR, 0.81 [97.5% CI, 0.56-1.12]), and 0.739 for standard support and 0.721 for enhanced support (adjusted OR, 0.96 [97.5% CI, 0.67-1.38]).

**Conclusions and Relevance:**

This comparison of cluster randomized trials found that interventions to improve feedback usability and guide local action were no more effective than standard feedback in increasing the appropriate use of blood transfusions. Auditing and feedback delivered at scale is a complex and costly program; therefore, effective responses may depend on developing robust local quality improvement arrangements, which can be evaluated using rigorous experimental designs embedded within national programs.

**Trial Registration:**

isrctn.org Identifier: ISRCTN15490813

## Key Points

**Question:**

What are the effects of 2 theoretically informed enhancements to feedback delivered by a national clinical audit program on the appropriate use of blood transfusions?

**Findings:**

In this comparison of linked 2 × 2 cluster randomized trials comprising 135 clusters in a surgical trial (2714 patients) and 134 clusters in a hematological trial (4439 patients) in which hospitals were randomized to enhanced content feedback or enhanced support for staff, no benefit of the enhanced interventions over standard feedback was observed in increasing appropriate use of blood transfusions. There was incomplete local engagement with the audit program as a whole across the intervention and standard feedback groups.

**Meaning:**

Given the considerable uncertainty about how to optimize the full potential of auditing and feedback in routine practice, these findings suggest a need to develop and evaluate ways of strengthening local responses to feedback.

## Teaser

This study compares the effects of 2 cluster randomized trials evaluating enhanced content and enhanced support interventions for perioperative anemia and the use of red blood cell and platelet transfusion for the management of hematological malignant disease.

## Introduction

Blood for transfusion is a finite and costly resource that has known risks.^1^ Research advocates restrictive transfusion practice.^2^ In England, the National Comparative Audit of Blood Transfusion (NCABT) assesses the appropriateness of transfusions and provides comparative performance feedback in a rolling program across different clinical specialties. However, repeated audits have found persistent, significant proportions of transfusions judged as inappropriate when compared against best practice recommendations.^1^ Auditing and feedback are generally effective in changing practice, but how to optimize these effects remains uncertain.^3^

The AFFINITIE (Audit and Feedback Interventions to Increase Evidence-Based Transfusion Practice) program aimed to design and evaluate enhanced feedback interventions within the NCABT and thereby increase appropriate use of blood components.^4^ We followed guidance on complex interventions^5^ in designing 2 feedback interventions to address specific weaknesses in feedback cycles identified in prior qualitative research.^3,6,7,8,9,10,11^ We evaluated these enhanced content and enhanced support interventions for 2 transfusion topics: perioperative anemia management (surgical trial) and red blood cell and platelet use in patients with hematological malignant disease (hematological trial).^12^

## Methods

### Study Design and Participants

As described in our trial protocol (Supplement 1), we conducted 2 sequential 2 × 2 factorial, repeated cross-sectional, cluster randomized clinical trials to empirically optimize feedback provided in response to national audits.^4,13^ The NCABT invited all National Health Service (NHS) hospitals to participate. We randomized at the organizational level in which transfusion teams worked (hence termed *clusters*) to prevent contamination between multiple sites covered by single hospital transfusion teams. Eligible clusters could take part in one or both of the audits delivered by the NCABT. For the surgical audit, we randomized clusters after baseline audit data collection, assessing outcomes 12 months after randomization using follow-up audit data. We rerandomized clusters for the hematological trial. Our repeated cross-sectional design anticipated that different patients would be audited during baseline and follow-up while recognizing overlaps in clinical staff administering transfusions in a cluster over time. Ethical approval for this trial was obtained through the UK National Research Ethics Service. We obtained permission for trial participation from hospital research and development departments. Individual patient and clinician consent were waived because the trial involved organization-level interventions. The study followed the Consolidated Standards of Reporting Trials (CONSORT) reporting guideline for cluster randomized trials.

### Randomization

We randomized clusters 1:1:1:1 using a computer-generated minimization program to (1) standard content and standard support, (2) standard content and enhanced support, (3) enhanced content and standard support, and (4) enhanced content and enhanced support. Minimization factors were cluster size (numbers of baseline audit cases), regional transfusion committee (responsible for regional educational oversight of transfusion practice), and previous allocation in the surgical trial (to balance any learning effects). Blinding of local staff receiving feedback was not possible.

### Feedback Interventions

#### Standard Audit and Feedback

The NCABT convenes a writing group consisting of the audit lead (often a clinician with an interest in transfusion), a statistician, and additional clinical staff. This group selects audit standards based on guidelines, the data to be collected, and the basic structure of feedback reports, supported by the NCABT program manager. Manual audit data collection is usually undertaken by local audit staff or transfusion clinicians following established NCABT policies. The resulting reports are fed back to hospital transfusion teams (eg, a transfusion clinician, consultant hematologist, and transfusion laboratory manager) and uploaded via a hospital-specific NCABT audit web page, often 12 months or more after data collection. Feedback typically consists of a detailed report (approximately 50 pages long),^11,14^ a regional presentation slide set, and (sometimes) action plan templates. Each team is responsible for disseminating feedback within its hospital and local action to improve practice.

#### Enhanced Feedback Content

A different NCABT audit writing group received an enhancement guidance manual, developed by the study team, detailing how to write feedback reports that included evidence-based feedback characteristics and behavior change techniques (eg, setting explicit goals and action plans), behaviorally specific audit standards, comparative performance feedback, and recommendations for change.^7,8,9,11^ We supported the writing group to ensure fidelity to the enhancement guidance. We provided 3 levels of enhanced content: a brief report highlighting comparative performance and recommendations for selected key audit standards (maximum of 6 pages), a longer report covering all audit standards (maximum of 34 pages), and the longer, detailed standard report. All feedback reports were uploaded to each hospital site’s individual NCABT webpage as usual. The writing groups for enhanced and standard feedback reports had no prior knowledge of which clusters were assigned to the intervention.

#### Enhanced Feedback Support

Enhanced feedback support consisted of a web-based toolkit for use by hospital transfusion teams in addition to standard support.^11^ It promoted 3 responses to feedback: disseminating findings to all relevant clinical staff involved in transfusion decision-making; localized, team-level goal setting, problem solving, and action planning to change practice; and continued local monitoring of practice. Local staff could access the toolkit via a unique NCABT web link for their own hospital. Hospital transfusion teams received an initial telephone call from an intervention facilitator, offering support and advice on how to use the toolkit.

As described in an intervention protocol for developing feedback enhancements,^11^ we evaluated the feasibility and acceptability of prototypes using semistructured and think-aloud interviews. Responses were mostly positive (eg, on the clarity of structured feedback reports with specific recommendations for action) while also suggesting refinements (eg, a more interactive web-based toolkit). Our Template for Intervention Description and Replication checklist provides a comprehensive account of intervention rationale, content, and delivery (eAppendix 1 in Supplement 2). We also provide sample reports (eAppendix 2 in Supplement 2).

### End Points

The primary outcome for both trials was whether a patient received transfusions all categorized as appropriate or not using the NCABT follow-up audit 12 months after randomization, analyzed at patient level. The surgical and hematological audits applied 11 and 13 standards, respectively, to assess various aspects of quality of care. We derived algorithms that avoided double-counting patients (eg, who might have both preoperative and postoperative transfusions). To minimize the risk of detection bias, we convened an independent panel of 2 clinicians and a statistician to review and approve the final algorithms. The panel considered baseline adherence to ensure that there would be scope for improvement in selecting primary outcomes and clinical relevance in, for example, considering whether to apply strict or relaxed interpretations of adherence to audit standards. The subsequent analysis involved applying the approved algorithm to patient data; no clinical judgment was required at the patient level.

Secondary outcomes consisted of volume of transfusions (aiming for reductions at patient and cluster levels) and transfusion-related adverse events and reactions. We also assessed adherence to a subset of core audit standards for both audit topics.

### Data Collection

Existing NCABT procedures were followed for piloting data collection. The online audit tool incorporated logic and compulsory fields to maximize data completeness. The data items collected depended on the standardized clinical algorithm developed for each topic together with basic patient demographic variables. We gathered cluster-level data on the volume of transfusions from 1 year before randomization to 1 year after randomization, from the Blood Stocks Management Scheme and anonymized patient data on adverse events from the UK-wide Serious Hazards of Transfusion (SHOT) hemovigilance scheme.

As part of a parallel process evaluation (to be fully reported separately), we verified fidelity of intervention delivery by checking uploads on the NCABT website according to allocation.^15^ Initial fidelity of receipt and engagement with the interventions was assessed using objectively collected web analytics data during the trial period.^16^ The NCABT website recorded how often feedback reports were downloaded.

### Statistical Analysis

#### Sample Size

For each trial, 2 principal comparisons of interest were related to the 2 main effects of the 2 × 2 factorial design: enhanced vs standard content and enhanced vs standard support. We assumed 80% appropriate transfusions at follow-up for each trial,^1^ an intracluster correlation of 0.05,^17^ and cluster sizes varying from 17 to 68 audit cases with a mean (SD) of 45 (6). We required 152 clusters (and 6840 audit cases) to detect a minimally important absolute increase of 6% in appropriate transfusions in the presence of, at most, a small antagonistic statistical interaction^18^ (ie, main effects of 5%) with 80% power using logistic regression models, with a random intercept for cluster and 2-sided 2.5% significance levels for each main effect.

#### Primary Analysis

Data were analyzed from November 1, 2016, to June 1, 2019. We conducted no interim analyses. We undertook primary data summaries and analyses on the intention-to-treat sample, analyzing all clusters as randomized. This was limited only by loss to follow-up of clusters. We anticipated a nontrivial proportion of missing patient data. We therefore explored mechanisms for missing patient data on key variables and imputed data 100 times using multiple imputation with chained equations, assuming patient data were missing at random. Sensitivity analyses assessed whether conclusions were robust across approaches for handling missing data.

We compared primary outcomes separately for each trial using multilevel logistic regression, adjusting for design factors (ie, cluster size, regional transfusion committee, previous allocation) and cluster-level proportion of necessary transfusions at baseline, with effect-coded (−0.5 or +0.5) main effects for enhanced vs standard content, enhanced vs standard support, and their interaction.^13^ We reported patient-level secondary end points of volume of blood transfused (derived from audit data) descriptively, as well as cluster-level volume of transfusions (Blood Stocks Management Scheme data) and adverse events (SHOT data). Components of the primary outcome and core audit standards provided sensitivity analyses for primary analyses. A random intercept model accounted for clustering arising from cluster randomization in all analyses. Model convergence was unreliable with more complex structures. We conducted analyses in SAS, version 9.4 (SAS Institute Inc).

#### Economic Analysis

Our cost-effectiveness analysis adopted an NHS perspective, comparing enhanced vs standard content and enhanced vs standard support for both trials. The time horizon was 1 year. We used resource and outcome data collected in the trials. Costs included feedback interventions, audit costs, additional NHS activity, and transfusions. We obtained unit costs from the trials and from published sources.^19,20,21^ We estimated incremental cost-effectiveness ratios for the proportions of appropriate transfusions, volume of blood transfused, and number of SHOT-reported events. We conducted deterministic and probabilistic sensitivity analyses. A budget impact analysis estimated intervention costs at a national level and the potential to be cost neutral.

## Results

### Surgical Trial

In the surgical trial, we screened 189 NHS trusts and health boards and identified 152 clusters (80.4%) included in the audit. The 2714 patients analyzed across all clusters had a mean (SD) age of 74.9 (14.0) years; 905 were men (33.3%) and 1809 (66.7%) were women. The most common procedures were orthopedic (928 [34.2%]) and fracture repair for the neck of the femur (839 [30.9%]).

**Figure 1.**
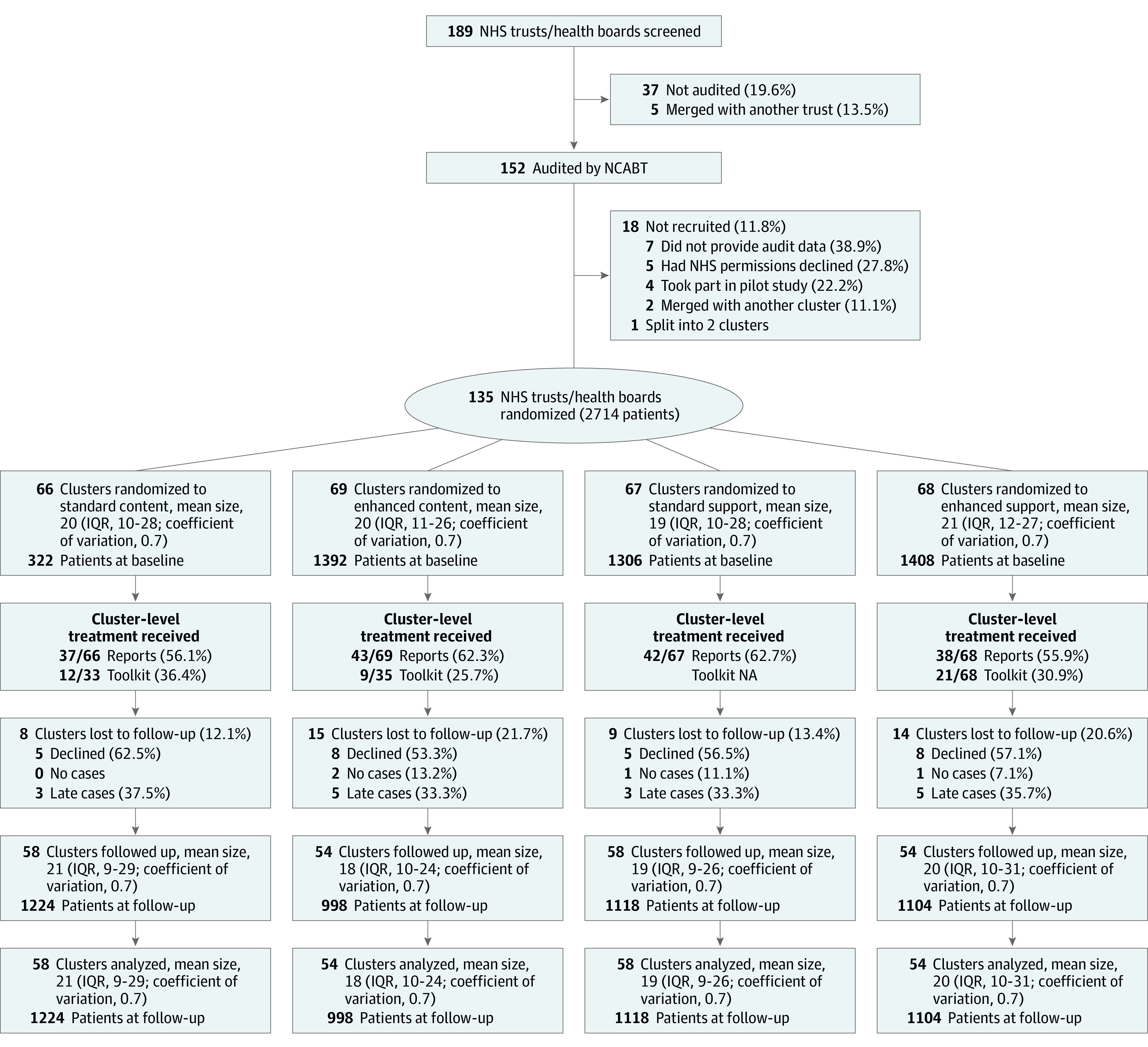
CONSORT Flow Diagram of the Surgical Trial NA indicates not applicable; NCABT, National Comparative Audit of Blood Transfusion; NHS, National Health Service.

Surgical trial baseline data collection was from October 1, 2014, to September 30, 2015, with follow-up data collection from November 1, 2015, to October 31, 2016. Follow-up was completed on October 31, 2016. We randomized 135 of 152 clusters (88.8%) on October 15, 2015, assigning 66 clusters to standard content, 69 to enhanced content, 67 to standard support, and 68 to enhanced support. Mean cluster size was 20 patients, with a coefficient of variation of 0.7 (Figure 1).

All interventions were delivered to clusters as allocated (ie, 100% correct uploads of enhanced vs standard feedback interventions). Fifty-seven of 69 sites (82.6%) randomized to enhanced content downloaded reports at least once compared with 52 of 66 sites (78.8%) randomized to standard reports. Fifty-nine of 68 sites (86.8%) randomized to enhanced support logged onto the toolkit at least once. Twenty-three clusters (17.0%) were lost to follow-up, more in enhanced vs standard content (15 of 69 [21.7%] vs 8 of 66 [12.1%]) and in enhanced vs standard support (14 of 68 [20.6%] vs 9 of 67 [13.4%]). Two of these clusters had no cases to audit, 8 provided audit data too late for trial inclusion, and 13 declined to participate. At follow-up, mean cluster size was also 20 patients, with a coefficient of variation of 0.7.

The baseline and follow-up audits included 2714 and 2222 patients, respectively, with generally well-balanced characteristics. Transfusion timings were similar between baseline and follow-up periods: 249 patients (9.2%) at baseline and 247 patients (11.1%) at follow-up received preoperative transfusions; 363 patients (13.4%) at baseline and 373 patients (16.7%) at follow-up received intraoperative transfusions; and 2560 patients (94.3%) at baseline and 2104 patients (94.7%) at follow-up received postoperative transfusions (Table 1 and Table 2).

**Table 1.** Baseline Patient-Level Characteristics^a^

Characteristic	Intervention				All
	Content		Support		
	Standard	Enhanced	Standard	Enhanced	
**Surgical trial**					
No. of patients	1322	1392	1306	1408	2714
Age, mean (SD), y [No. of patients contributing]	74.7 (13.8) [1318]	75.1 (14.1) [1383]	75.3 (13.8) [1302]	74.6 (14.1) [1399]	74.9 (14.0) [2701]
Sex					
Men	435 (32.9)	470 (33.8)	418 (32.0)	487 (34.6)	905 (33.3)
Women	887 (67.1)	922 (66.2)	888 (68.0)	921 (65.4)	1809 (67.7)
Surgical procedure					
Orthopedic	444 (33.6)	484 (34.8)	435 (33.3)	493 (35.0)	928 (34.2)
Cardiac	233 (17.6)	222 (15.9)	222 (17.0)	233 (16.5)	455 (16.8)
Fractured neck of femur	421 (31.8)	418 (30.0)	410 (31.4)	429 (30.5)	839 (30.9)
Other	222 (16.8)	258 (18.5)	234 (17.9)	246 (17.5)	480 (17.7)
Missing	2 (0.1)	10 (0.7)	5 (0.4)	7 (0.5)	12 (0.4)
Attendance at preoperative clinic	839 (63.5)	922 (66.2)	841 (64.4)	920 (65.3)	1761 (64.9)
Surgery complications	328 (24.8)	381 (27.4)	326 (25.0)	383 (27.2)	709 (26.1)
Patient died	49 (3.7)	63 (4.5)	61 (4.7)	51 (3.6)	112 (4.1)
Transfusion					
Preoperative	120 (9.1)	129 (9.3)	114 (8.7)	135 (9.6)	249 (9.2)
Intraoperative	179 (13.5)	184 (13.2)	171 (13.1)	192 (13.6)	363 (13.4)
Postoperative	1245 (94.2)	1315 (94.5)	1235 (94.6)	1325 (94.1)	2560 (94.3)
No. of preoperative units transfused					
1	26 (21.7)	26 (20.1)	19 (16.7)	33 (24.4)	52 (20.9)
≥2	93 (77.5)	101 (78.3)	94 (82.5)	100 (74.1)	194 (77.9)
Missing	1 (0.8)	2 (1.5)	1 (0.9)	2 (1.5)	3 (1.2)
No. of postoperative units transfused					
1	414 (33.3)	353 (26.8)	355 (28.7)	412 (31.1)	767 (30.0)
≥2	818 (65.7)	940 (71.5)	864 (70.0)	894 (67.5)	1758 (68.7)
Missing	13 (1.0)	22 (1.7)	16 (1.3)	19 (1.4)	35 (1.4)
**Hematological trial**					
No. of patients	2228	2211	2188	2251	4439
Age, median (IQR), y [No. of patients contributing]	73.0 (64.0-80.0) [2227]	72.0 (64.0-80.0) [2208]	72.0 (64.0-80.0) [2187]	72.0 (64.0-80.0) [2248]	72.0 (64.0-80.0) [4435]
Sex					
Men	1306 (58.6)	1335 (60.4)	1301 (59.5)	1340 (59.5)	2641 (59.5)
Women	922 (41.4)	876 (39.6)	887 (40.5)	911 (40.5)	1798 (40.5)
Hematological disorder diagnosis					
Acute leukemia	469 (21.1)	460 (20.8)	435 (19.9)	494 (21.9)	929 (20.9)
Chronic leukemia/lymphoma and myeloma	751 (33.7)	752 (34.0)	754 (34.5)	749 (33.3)	1503 (33.9)
MDS and aplastic anemia	1038 (46.6)	975 (44.1)	1006 (46.0)	1007 (44.7)	2013 (45.3)
Additional treatment for hematological disorder diagnosis	723 (32.5)	615 (27.8)	617 (28.2)	721 (32.0)	1338 (30.1)
Stem cell transplant	128 (5.7)	126 (5.7)	94 (4.3)	160 (7.1)	254 (5.7)
Intensive chemotherapy	574 (25.8)	461 (20.9)	494 (24.5)	541 (24.0)	1035 (23.3)
Participating in clinical study	191 (8.6)	166 (7.5)	195 (8.9)	162 (7.2)	357 (8.0)
Transfusion type					
Red blood cell and platelet	744 (33.4)	643 (29.1)	683 (31.2)	704 (31.3)	1387 (31.2)
Red blood cell only	1360 (61.0)	1421 (64.3)	1377 (62.9)	1404 (62.4)	2781 (62.6)
Platelet only	124 (5.6)	147 (6.6)	128 (5.9)	143 (6.4)	271 (6.1)
Red blood cell transfusions, No.	2104	2064	2060	2108	4168
No. of units transfused, mean (SD) [No. of patients contributing]	1.9 (0.6) [2098]	2.0 (0.6) [2055]	1.9 (0.6) [2051]	2.0 (0.6) [2102]	2.0 (0.68) [4153]
Additional units transfused, median (IQR) [No. of patients contributing]	1.0 (0.0-3.0) [2060]	1.0 (0.0-3.0) [2040]	1.0 (0.0-3.0) [2017]	1.0 (0.0-2.0) [2083]	1.0 (0.0-3.0) [4100]
Platelet transfusions					
No. of patients	868	790	811	847	1658
No. of units transfused, mean (SD) [No. of patients contributing]	1.1 (0.4) [857]	1.1 (0.6) [782]	1.1 (0.4) [800]	1.1 (0.6) [839]	1.1 (0.5) [1639]
Additional units transfused, median (IQR) [No. of patients contributing]	2.0 (0.0-5.0) [854]	2.0 (0.0-5.0) [779]	3.0 (0.0-6.0) [798]	2.0 (0.0-5.0) [835]	2.0 (0.0-5.0) [1633]

Abbreviation: MDS, myelodysplastic syndrome.

^a^ Unless otherwise indicated, data are expressed as number (%) of patients.

**Table 2.** Patient-Level Outcomes at Follow-up

Variable	Intervention^a^				
	Content		Support		All
	Standard	Enhanced	Standard	Enhanced	
**Surgical trial**					
Entire national audit sample, No.	1224	998	1118	1104	2222
Primary outcome					
Appropriate	198 (16.2)	152 (15.2)	176 (15.7)	174 (15.8)	350 (15.8)
Inappropriate	901 (73.6)	726 (72.7)	822 (73.5)	805 (72.9)	1627 (73.2)
Unclassified: hemoglobin level missing	125 (10.2)	120 (12.0)	120 (10.7)	125 (11.3)	245 (11.0)
Secondary outcome					
Total volume of blood transfused, mean (SD) [No. of patients contributing]	2.0 (1.2) [1147]	2.2 (1.7) [921]	2.1 (1.6) [1052]	2.1 (1.3) [1016]	2.1 (1.5) [2068]
**Hematological trial**					
Entire national audit sample, No.	1926	1933	1779	2080	3859
Primary outcome					
Appropriate	1308 (67.9)	1226 (63.4)	1196 (67.2)	1338 (64.3)	2534 (65.7)
Inappropriate	457 (23.7)	507 (26.2)	433 (24.3)	531 (25.5)	964 (25.0)
Unclassified	161 (8.4)	200 (10.3)	150 (8.4)	211 (10.1)	361 (9.4)
Red blood cell transfusions, No.	1815	1832	1674	1973	3647
Secondary outcome					
Volume transfused, median (IQR) [No. of patients contributing]	2.0 (1.0-2.0) [1813]	2.0 (1.0-2.0) [1829]	2.0 (1.0-2.0) [1671]	2.0 (1.0-2.0) [1971]	2.0 (1.0-2.0) [3642]
Platelet transfusions, No.	729	717	633	813	1446
Secondary outcome					
Volume transfused, median (IQR) [No. of patients contributing]	1.0 (1.0-1.0) [716]	1.0 (1.0-1.0) [705]	1.0 (1.0-1.0) [626]	1.0 (1.0-1.0) [795]	1.0 (1.0-1.0) [1421]

^a^ Unless otherwise indicated, data are expressed as number (%) of patients.

For the primary outcome (Table 3), the proportion of patients receiving appropriate transfusions was 0.184 for standard content and 0.176 for enhanced content clusters, with an adjusted odds ratio (OR) of 0.91 (97.5% CI, 0.61-1.36), and was 0.181 for standard support and 0.180 for enhanced support, with an adjusted OR of 1.05 (97.5% CI, 0.68-1.61). We observed no statistically or clinically significant effects for both comparisons and no effects on secondary or other outcomes. The results were robust to assumptions about missing data.

**Table 3.** Primary Trial Analyses^a^

Analysis	No. of patients	Intervention, proportion appropriate		Estimates			*P* value
		Standard	Enhanced	Adjusted risk difference (95% CI)	Adjusted OR (97.5% CI)	Adjusted OR (95% CI)	
Surgical trial							
Content	2222	0.184	0.176	−0.01 (−0.07 to 0.04)	0.91 (0.61 to 1.36)	0.91 (0.64 to 1.30)	.61
Support	2222	0.181	0.180	0.01 (−0.05 to 0.06)	1.05 (0.68 to 1.61)	1.05 (0.72 to 1.52)	.81
Interaction	2222	0.184	0.167	0.05 (−0.08 to 0.13)	1.15 (0.52 to 2.56)	1.15 (0.57 to 2.31)	.70
Hematological trial							
Content	3859	0.744	0.714	−0.04 (−0.10 to 0.01)	0.81 (0.56 to 1.12)	0.81 (0.60 to 1.08)	.15
Support	3859	0.739	0.721	−0.01 (−0.06 to 0.05)	0.96 (0.67 to 1.38)	0.96 (0.71 to 1.32)	.82
Interaction	3859	0.737	0.707	0.03 (−0.08 to 0.14)	1.22 (0.60 to 2.48)	1.22 (0.66 to 2.27)	.52

Abbreviation: OR, odds ratio.

^a^ Performed using multiple imputation (100 imputations), in the full imputation model. Content and support are effect coded (−1 of 2 and +1 of 2), where standard is coded −1 of 2 and enhanced is coded +1 of 2. Therefore, the interaction effect is coded +1 of 4, where content and support are both coded −1 of 2 or both coded +1 of 2 (under enhanced), and −1 of 4 otherwise (under standard). In all cases, standard is the comparator group.

### Hematological Trial

We screened 187 NHS trusts and health boards and identified 141 clusters (75.4%) that were included in the audit. The 4439 patients analyzed across all clusters had a median age of 72.0 (IQR, 64.0-80.0) years; 2641 were men (59.5%) and 1798 were women (40.5%). The most common hematological diseases were myelodysplastic syndrome and aplastic anemia (2013 cases [45.3%]).

**Figure 2.**
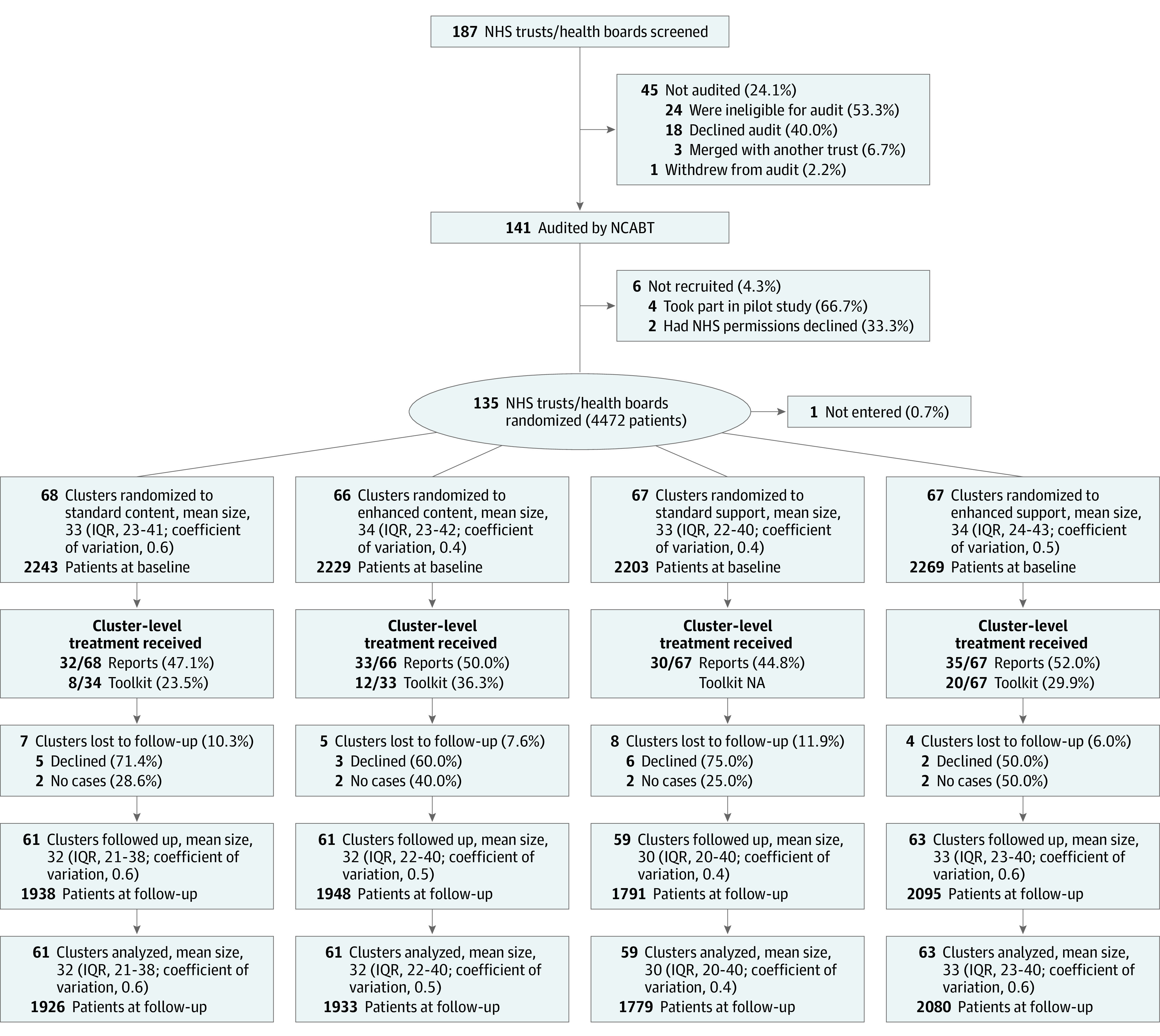
CONSORT Flow Diagram of the Hematological Trial NA indicates not applicable; NCABT, National Comparative Audit of Blood Transfusion; NHS, National Health Service.

Data were collected for trial baseline, including follow-up, was from July 1, 2015, to June 30, 2017. We randomized 135 of 141 (95.7%) clusters on July 6, 2016, including 1 in error, and randomized 68 clusters to standard content, 66 to enhanced content, 67 to standard support, and 67 to enhanced support. The mean cluster size was 33 patients, with a coefficient of variation of 0.5 (Figure 2).

All clusters received interventions as allocated (100%). Fidelity of initial receipt was similarly high; 53 of 66 clusters (80.3%) randomized to enhanced content downloaded the reports at least once compared with 53 of 68 (77.9%) randomized to standard content. Forty-nine of 67 clusters (73.1%) randomized to enhanced support logged into the Toolkit at least once.

Twelve clusters (8.9%) were lost to follow-up (enhanced vs standard content, 5 [7.6%] vs 7 [10.3%]; enhanced vs standard support, 4 [6.0%] vs 8 [11.9%]). Four of these clusters had no cases to audit and 8 declined to participate. At follow-up, mean cluster size was 32 patients, with a coefficient of variation of 0.5.

The baseline and follow-up audits included 4472 patients at baseline and 3859 patients at follow-up. Characteristics were generally well-balanced characteristics in both periods: 1387 (31.7%) at baseline and 1207 (31.3%) at follow-up received both red blood cell and platelet transfusions; 2781 (63.6%) at baseline and 2440 (63.2%) at follow-up received red blood cell transfusions only; and 271 (6.2%) at baseline and 212 (5.5%) at follow-up received platelet transfusions only (Tables 1 and 2).

For the primary outcome (Table 3), the proportion of patients receiving appropriate transfusions was 0.744 for standard content and 0.714 for enhanced content with an adjusted OR of 0.81 (97.5% CI, 0.56-1.12), and 0.739 for standard support and 0.721 for enhanced support with an adjusted OR of 0.96 (97.5% CI, 0.67-1.38). We observed no statistically or clinically significant effects in either comparison and no effects on secondary or other outcomes (eAppendix 3 in Supplement 2). The results were again robust to assumptions about missing data.

### Economic Analysis

In the surgical trial, the additional cost per cluster of feedback was £237 ($345 US) for enhanced content and £32 ($47 US) for enhanced support, with similar costs for the hematological trial (eAppendix 4 in Supplement 2). The difference in cost between enhanced and standard feedback was predominantly due to additional research staff required to produce the enhanced feedback reports (eAppendix 4 [Tables 3 and 4] in Supplement 2). For enhanced content, we observed a 0.8% decrease in appropriate transfusions, an additional 770 (95% CI, 301-1239) red blood cell units transfused, and a reduction of 0.2 SHOT-reported events per cluster. For enhanced support, the corresponding figures were a 0.1% decrease, an additional 312 (95% CI, −137 to 761) red blood cell units transfused, and an increase of 1.3 SHOT-reported events per cluster. When considering all the cost components (including the cost of blood transfused and additional NHS activity generated), the additional cost per cluster in the surgical trial was £135 570 ($197 049 US) for enhanced content and £54 826 ($79 689 US) for enhanced support; these were driven mainly by the costs of the additional blood transfused. Therefore, standard feedback dominated both enhanced interventions. There was considerable uncertainty around the parameters used.

## Discussion

We conducted a rigorous evaluation of 2 empirically and theoretically based interventions embedded within a national audit program. Although both interventions were aimed at enhancing feedback content and supporting local responses in hospitals, neither one increased the numbers of transfusions classified as appropriate compared with standard feedback. Given that both interventions had been systematically designed to strengthen specific aspects of feedback methods, the observed absence of any effects is surprising.

### Limitations

The study’s limitations in local engagement with feedback, audit program design, and statistical power may explain our findings.^15^ We observed incomplete local engagement with the audit program across intervention and standard feedback groups. For example, staff from approximately 1 in 5 clusters did not download feedback reports to receive their comparative feedback, despite having collected audit data, whereas 1 in 10 collected only baseline data. Experience with other large-scale quality improvement programs in hospital settings suggest that they can struggle to reach and engage clinical staff responsible for care delivery^22^ and can take longer and use more resources than envisaged to bring about change.^23^

In contrast to other repeating large-scale audit programs, which often focus on a core, limited set of indicators, the NCABT has a changing program of audit topics each year.^24^ This requires new audit criteria and methods for data collection and analysis and can come at a cost, because clinical engagement may be undermined if the audit criteria have not been fully validated and are not generally accepted over successive audits.^25^

There was a loss of participating clusters at follow-up, compromising statistical power. However, the overall results appeared consistent across both settings and interventions, and the confidence intervals excluded prespecified minimally important clinical effects. The overall number of appropriate transfusions in the surgical trial was less than anticipated, which probably reflects the strict threshold for appropriateness agreed by the independent panel reviewing algorithm for the primary outcome.

## Conclusions

This comparison of 2 cluster randomized trials represents a head-to-head trial comparing different ways of delivering feedback, as advocated by international implementation scientists.^26^ The limitations in broader levels of engagement identified in the context of 1 audit program in transfusion are concerning and may have relevance to other large-scale performance feedback programs, including access to reports and credibility of audit standards, such that their full potential to bring about significant changes in population health care remains underrealized.^25,26,27^ Proponents of auditing still lack answers to key questions about how to optimize feedback to improve patient care, such as which comparators are more likely to motivate change or how much information to include in feedback reports.^27,28,29,30,31,32^ Robust local quality improvement arrangements are integral to delivery of effective responses to audit programs, and further research is needed to determine the best means of facilitating local responses. There is a need to maximize returns from the considerable resources invested in such large-scale programs (including clinician time) by integrating further experimental research within them. Such work could build on the AFFINITIE program by adopting a learning health system involving a series of rapid-cycle randomized evaluations embedded within routine systems of audit.^33,34,35^

## Notes

Supplement 1.
Trial Protocol

Supplement 2. **eAppendix 1.** AFFINITIE TIDIER Checklist for the AFFINITIE Enhanced Content and Enhanced Follow-up Interventions **eAppendix 2.** Sample Reports **eAppendix 3.** Trial Analyses **eAppendix 4.** Economic Analysis

Supplement 3.
Nonauthor Collaborators

Supplement 4.
Data Sharing Statement
