# Supplementary material for: Effectiveness of Enhanced Performance Feedback on Appropriate Use of Blood Transfusions: A Comparison of 2 Cluster Randomized Trials
Source: JAMA Netw Open. 2022 Feb 24;5(2):e220364. doi: 10.1001/jamanetworkopen.2022.0364 (PMC8874348; doi:10.1001/jamanetworkopen.2022.0364)
Supplement: Supplement 2. — eAppendix 1. AFFINITIE TIDIER Checklist for the AFFINITIE Enhanced Content and Enhanced Follow-up Interventions eAppendix 2. Sample Reports eAppendix 3. Trial Analyses eAppendix 4. Economic Analysis [file jamanetwopen-e220364-s002.pdf]

Supplementary Online Content

Stanworth SJ, Walwyn R, Grant-Casey J, et al; AFFINITIE Collaborators. Effectiveness of enhanced performance feedback on appropriate use of blood transfusions: a comparison of 2 cluster randomized trials. *JAMA Netw Open*. 2022;5(2):e220364. doi:10.1001/jamanetworkopen.2022.0364

eAppendix 1. AFFINITIE TIDIER Checklist For the AFFINITIE Enhanced Content and Enhanced Follow-up Interventions

eAppendix 2. Sample Reports

eAppendix 3. Trial Analyses

eAppendix 4. Economic Analysis

This supplementary material has been provided by the authors to give readers additional information about their work.

© 2022 Stanworth SJ et al. *JAMA Network Open*.

eAppendices

eAppendix 1. TIDIER Checklist For the AFFINITIE Enhanced Content and Enhanced Follow-up Interventions
eAppendix 2. Sample Reports
eAppendix 3. Trial
eAppendix 4. Economic Analysis

© 2022 Stanworth SJ et al. *JAMA Network Open*.

eAppendix 1. TIDIER Checklist For the AFFINITIE Enhanced Content and Enhanced Follow-up Interventions

**BRIEF NAME**	
AFFINITIE 'enhanced content': an intervention to develop theory- and evidence-based audit feedback documents	AFFINITIE 'enhanced follow-on' support: a theory- and evidence-based online toolkit to support hospitals' response to audit feedback

WHY Describe any rationale, theory, or goal of the elements essential to the intervention

Intervention theory and rationale

Each intervention drew on: (A) existing evidence on what makes audit and feedback (A&F) more effective, (B) behavioural science, and (C) empirical work.

A) Evidence about what makes A&F more effective

A Cochrane systematic review on the effectiveness of A&F found that it is more effective when feedback: is provided by colleagues or supervisors, is provided in both verbal and written formats, includes explicit targets and action plans to change behaviour, and includes achievable benchmarks/comparators [1].

B) Behavioural science

B.1: Control theory

- Control Theory posits that individuals manage their behaviour by: deciding what they want to do or achieve → trying to do it → monitoring their behaviour → assessing whether they are making progress towards their goal → and adapting behaviour [2]. This process is proposed to occur in a cyclical manner (i.e. a feedback loop) and progress through the loop is facilitated by close correspondence between each step.
- Control Theory has been used to understand how A&F works [3]. As part of an NHS Blood & Transplant (NHSBT) national comparative audit (NCA) cycle, this may involve: NHSBT setting audit standards → hospitals' providing data on clinical practice (i.e. behaviour) → NHSBT auditing practice → NHSBT providing audit findings in feedback documents → hospital staff noting a discrepancy between actual practice and goal performance based on audit feedback (or not) → hospital staff responding to audit feedback (or not).

B.2: Behaviour change techniques (BCTs)

- BCTs are defined as the "observable, replicable and irreducible components of an intervention designed to alter or redirect causal processes that regulate behaviour" (i.e. 'active ingredients' of interventions) [4].
- Each component of the Control Theory loop can be mapped onto one or more BCTs from a comprehensive BCT taxonomy [4].

B.3: Actor, Action, Context, Timeframe, Target (AACTT) framework

- There is evidence that using specific, concrete wording can increase the likelihood of information being understood and remembered [5].
- The AACTT framework provides guidance for increasing behavioural specificity. Where appropriate and feasible, the AACTT framework may be used to specify behaviour in terms of: who (Actor), should do what (Action), where (Context), when (Timeframe) [6].

© 2022 Stanworth SJ et al. *JAMA Network Open*.

C) Empirical work
The AFFINITIE programme aimed to gather evidence to evaluate how to enhance NCAs conducted by NHSBT [7]. This work involved a content analysis and document review of audit feedback from NCA cycles, a multiple-case study based on interviews and observations conducted in four UK hospitals, multidisciplinary consensus panels to select enhancements to include in each intervention, intervention piloting, and feasibility and acceptability interviews with clinical transfusion staff (Gould et al 2014).

Intervention aims

The Enhanced Content intervention aimed to support the audit leads from the NCA of Blood Transfusion to develop feedback reports incorporating theory- and evidence-based feedback characteristics and components. It is in turn intended that these will be delivered to clinical staff involved in blood transfusion and that the enhanced content reports will be easier and less burdensome to read, and will facilitate staff identifying how they are currently performing, areas of discrepancy with standards and comparators, and key recommendations, targets, and actions for change in light of feedback.

The Enhanced Follow on intervention is intended to prompt and support staff to engage in local response to the feedback, including supporting four key behaviours: dissemination of feedback to key stakeholder groups within the hospital, identifying potential targets for change in light of feedback (Goal setting), action planning and problem solving around potential barriers/enablers to implementing change in light of feedback, , and re-monitoring practice locally to assess subsequent progress and improvements in performance.

Both interventions were designed to be **sustainable** beyond the AFFINITIE programme by providing materials and templates for intervention recipients to adapt and/or reuse in future NCAs.

**WHAT** *Materials:* Describe any physical or informational materials used in the intervention, including those provided to participants or used in intervention delivery or in training of intervention providers. Provide information on where the materials can be accessed (e.g. online appendix, URL).	
'Enhanced content' included two components: 1) **training audit teams from the NCA of blood transfusion** to write feedback reports containing recommended enhancements to improve the theory- and evidence-based design of the feedback reports**,** and the 2) the resulting **enhanced feedback reports**.	'Enhanced follow-on' included a main intervention, an online **toolkit**—to support hospitals in planning and responding locally to audit feedback—and a **telephone support** co-intervention to prompt, encourage and support key staff who respond to feedback (i.e. hospital transfusion team) to use the toolkit.

© 2022 Stanworth SJ et al. *JAMA Network Open*.

Training writing teams: Numerous materials were developed to train writing teams to include a number of proposed enhancements in feedback documents: (1) a Powerpoint presentation (2) template/prototype feedback documents, and (3) enhancement guidance documents (brief and full versions) (Appendices 9-10).

The proposed enhancements to feedback content were:

1. In each feedback document, include at least one BCT consistent with each step of Control Theory
2. Ensure audit standards, feedback, recommendations and action plans are behaviourally specific (i.e. based on AACTT)
3. Ensure feedback delivered is clearly related to an audit standard. Take a graded entry approach to feedback reports (see below)).
4. Ensure feedback includes multiple comparators (e.g. national/regional performance, past performance, top 10% of peers)
5. Include a positive message to encourage and recognise good performance
6. Where possible, re-monitor and repeat feedback
7. Improve feedback document presentation (e.g. provide feedback visual format [e.g. graphs], make writing legible, use a consistent layout, personalise feedback)

Feedback documents: The feedback reports included several audit standards (i.e. performance metrics) against which hospitals were audited, graphs to indicate both hospital and national performance for each standard, recommendations for improving practice, and suggestions for staff to disseminate feedback to. The feedback documents were designed to incorporate the above enhancements and to use a graded entry approach, which provided reports of different sizes and levels of detail on the audit findings:

1. <u>Key findings report</u>*:* A short summary (6 pages), briefly highlighting audit results and recommendations for key audit standards
2. <u>Full findings report</u>: A medium-sized document (≈30 pages) reporting the relevance of the audit, who was audited, and the findings and recommendations for all audit standards
3. <u>Supplementary findings report</u>: Large document (≈55 pages), reporting information on clinical context and/or any supporting information (e.g. audit data collection details)

Toolkit: The toolkit included various tools, and associated BCTs, which mapped onto the different stages of Control Theory (Appendices 13-14). The toolkit was divided into several sections (specific tools in italics):

1. <u>Introduction page</u>*:* Provided information about how to use the toolkit and how it was designed
2. <u>Engage clinical staff</u>*:* Included tools to help identify staff involved in transfusion decision-making and staff responsible for disseminating feedback (*Dissemination Cascade*), identify barriers to disseminating feedback and how to address these (*Fishbone Analysis*), and make plans based on goals for disseminating feedback (*Action Plan*)
3. <u>Improve patient care</u>*:* Included tools to identify which of the audit standards to target to improve practice (*Selecting Standards*), identify barriers to achieving goals to improve practice and how to address these (*Fishbone Analysis*), make plans based on goals for improving practice (*Action Plan*), and communicate key messages from the audit and the standards that are being targeted (*Poster*)
4. <u>Monitor practice</u>*:* Included tools to conduct small-scale re-audits (*QuickAudit*) and communicate progress towards goals after using the QuickAudit (*Poster*)
5. <u>Dashboard</u>*:* Provided an overview of progress through the toolkit

The toolkit also included features, such as:

- Interactive click buttons to add, remove, and edit elements of tools
- Click buttons to mark tools as complete and download tools in PDF format
- Editable fields in particular tools to make goals SMART (Specific, Measurable, Achievable, Realistic, Timely) and behaviourally specific (AACTT)

Telephone support: Materials to support training and delivery of telephone support for intervention facilitators included: (1) a flowchart with scripted text aimed at encouraging hospital contacts to log into the toolkit (2) a manual with scripted responses to potential queries for intervention facilitators if/when they were providing clinical transfusion staff an overview of the toolkit while on the phone, and (3) spreadsheets to log details of calls.

© 2022 Stanworth SJ et al. *JAMA Network Open*.

4. Action Plan*:* A document with rows to select standards to target and columns to behaviourally specify plans (based on TACTA)

WHAT *Procedures:* Describe each of the procedures, activities, and/or processes used in the intervention, including any enabling or support activities

Training writing teams: Feedback writing teams were provided training in how to apply enhancements to feedback reports in two phases: prior to and during the production of the feedback documents. Prior to producing the documents, training was provided by members of the AFFINITIE research team during an initial meeting with the audit/feedback writing team. The existing evidence and theory related to A&F was presented, alongside the proposed enhancements and the enhancement guidance materials. Members of the research team also provided ongoing support during the feedback report writing period, by joining teleconferences to discuss progress with the report, and reviewing/commenting on draft reports and providing recommendations on how to further or better incorporate the proposed enhancements.

Feedback documents: Procedures for delivering the resulting feedback reports to hospitals followed standard practice. NHS Trust specific reports were uploaded to each Trust's password protected A&F report library on the NCA of Blood Transfusion's web portal. The assigned contact for that audit at each Trust (typically a transfusion practitioner) was then sent an email to notify that their reports were uploaded and available for download.

Telephone support: All hospitals randomised to receive the toolkit were put on a list with phone numbers for audit contacts. The first part of calls focused on encouraging contacts to log into the toolkit (using the flowchart); if they logged into the toolkit, the second part of calls involved providing an overview of the toolkit and encouraging them to initially engage with using it (using the manual). Details about all calls were logged by intervention facilitators.

Toolkit: For hospitals randomised to receive the Toolkit, these were uploaded to the same we portal as described for the feedback reports. The assigned local audit contact was notified of the Toolkit's availability in the same notification email indicating the feedback reports were available for download.

WHO PROVIDED For each category of intervention provider (e.g. psychologist, nursing assistant), describe their expertise, background and any specific training given

Training writing teams: Intervention facilitators included members of the AFFINITIE research team: postdoctoral Research Fellow with a background in psychology (FL), a Professor of Primary Care (RF), a Consultant Haematologist (SS), and a Senior Medical Statistician (MC).

Feedback documents: NCA staff at NHSBT. (audit manager and coordinator) uploaded feedback documents to the NCA portal.

Telephone support: Intervention facilitators included members of the AFFINITIE research team: postdoctoral Research Fellow (NG), a postdoctoral Research Assistant (CD), and an MSc-level Research Assistant (SMcI). All facilitators had an educational background in psychology. Intervention facilitators were provided three-to-four training sessions prior to delivering telephone support. This involved introducing intervention facilitators to the BCTs delivered in telephone support and using materials in role plays.

Toolkit: NCA staff at NHSBT. (audit manager and coordinator) uploaded the Toolkit to the NCA portal.

HOW Describe the modes of delivery (e.g. face-to-face or by some other mechanism, such as internet or telephone) of the intervention and whether it was provided individually or in a group

© 2022 Stanworth SJ et al. *JAMA Network Open*.

**Training writing teams:** The initial training meeting was conducted in a face-to-face group meeting. Subsequent meetings were conducted face-to-face and by telephone, typically lasting one to two hours. Comments and edits on draft reports were provided via email.<br><br>**Feedback documents:** Feedback documents were uploaded online to the NHSBT webpage and accessible to hospital staff using a password-protected login. An e-mail was sent to listed audit contacts to notify them that the intervention was available.	**Telephone support:** Telephone support was provided individually.<br><br>**Toolkit:** A link to the toolkit was uploaded online to the NHSBT webpage and was accessible to hospital staff using a password-protected login. An e-mail was sent to listed audit contacts to notify them that the intervention was available.
**WHERE** Describe the type(s) of location(s) where the intervention occurred, including any necessary infrastructure or relevant features.	
**Training writing teams**: Face-to-face meetings were held in a private room at blood donor centres in England.<br><br>**Feedback documents:** Hospitals required an internet connection to download the feedback documents.	**Telephone support:** Intervention facilitators used a room with an external phone line located in their university.<br><br>**Toolkit:** Hospitals required an internet connection to use the toolkit.
**WHEN and HOW MUCH** Describe the number of times the intervention was delivered and over what period of time including the number of sessions, their schedule, and their duration, intensity or dose	
**Training writing teams**: The initial training meeting lasted half a day. Subsequent meetings and teleconferences lasted between 1 and 3 hours. Approximately three drafts of the feedback reports were reviewed.<br><br>**Feedback documents:** The intervention was delivered once when the feedback documents were uploaded to the NCA website and participating sites were notified by email.	**Telephone support:** One telephone support call was provided to hospitals over a month period following delivery of the toolkit. Calls lasted approximately 15 minutes on average.<br><br>**Toolkit:** The intervention was delivered once the link to the toolkit was uploaded to the NCA website and participating sites were notified by email. The toolkit was available for the intervention period.
**TAILORING** If the intervention was planned to be personalised, titrated or adapted, then describe what, why, when, and how	
**Training writing teams**: There was no tailoring of the training.<br><br>**Feedback documents:** Feedback documents included recommendations and suggestions for change based on the hospital's performance in relation to each standard (e.g. hospitals in the top third received recognition of good practice and a message of encourage. Hospitals in the bottom third were encouraged to set an intermediate goal of working towards the national average). Recommendations were also tailored based on professional/stakeholder groups (i.e. transfusion laboratory staff vs clinical staff prescribing transfusions)	**Telephone support:** All hospitals received a standardised initial telephone support discussion following a pre-specified flow chart. The telephone support manual also included a number of IF → Then scenarios, representing potential issues an intervention recipient may raise, and providing a suggested response (incorporating behaviour change techniques). This enabled tailoring of telephone support to local needs.<br><br>**Toolkit:** The tools in the Toolkit were not tailored. However, hospitals were encouraged to use the provided tools to locally tailor the recommendations (goals + action plans) in the feedback reports.
**MODIFICATIONS** If the intervention was modified during the course of the study, describe the changes (what, why, when, and how)	

© 2022 Stanworth SJ et al. *JAMA Network Open*.

Training writing teams: n/a

Feedback documents: During Trial 1, some feedback documents were re-issued due to errors in data analysis (e.g. incorrect units of measurement).

Telephone support: In Trial 1, after intervention facilitators contacted hospitals, there was a planned second phase of telephone support where hospitals were provided phone contact details to call us back with queries. As we only had one call from hospitals during Trial 1, this phone contact was replaced with an e-mail contact in Trial 2.

Toolkit: Three additions were made to the toolkit after Trial 1: (1) an undo/redo button, so users could more easily make edits, (2) a reset function, so that the toolkit could be restored to default, and (3) a timeout function of 15 minutes, to increase the validity of visit duration data.

HOW WELL Planned + Actual

Methods for assessing fidelity for both interventions are reported in the AFFINITIE process evaluation protocol [8]. Process evaluation findings are reported in Workstream 3.

Methods for assessing fidelity for both interventions are reported in the AFFINITIE process evaluation protocol [8]. Process evaluation findings are reported in Workstream 3.

References

1. Ivers NM, Jamtvedt G, Flottorp S, Young JM, Odgaard-Jensen J, French SD, et al. Audit and feedback: effects on professional practice and healthcare outcomes. Cochrane Database Syst Rev. 2012;6:CD000259.
2. Carver CS, Scheier MF. Control theory: A useful conceptual framework for personality-social, clinical, and health psychology. Psychological Bulletin. 1982;92(1):111-35.
3. Gardner B, Whittington C, McAteer J, Eccles MP, Michie S. Using theory to synthesise evidence from behaviour change interventions: the example of audit and feedback. Social science & medicine. 2010;70(10):1618-25.
4. Michie S, Richardson M, Johnston M, Abraham C, Francis J, Hardeman W, et al. The behavior change technique taxonomy (v1) of 93 hierarchically clustered techniques: Building an international consensus for the reporting of behavior change interventions. Annals of behavioral medicine : a publication of the Society of Behavioral Medicine. 2013;46(1):81-95.
5. Michie S, Johnston M. Changing clinical behaviour by making guidelines specific. Bmj. 2004;328(7435):343-5.
6. Presseau, McCleary, Lorencatto, Patey, Grimshaw, Francis. (Under Review) Action, Actor, Context, Target, Time (AACTT): A framework for specifying behaviour. Implementation Science.

© 2022 Stanworth SJ et al. *JAMA Network Open*.

7. Gould NJ, Lorencatto F, Stanworth SJ, Michie S, Prior ME, Glidewell L, et al. Application of theory to enhance audit and feedback interventions to increase the uptake of evidence-based transfusion practice: an intervention development protocol. Implement Sci. 2014;9:92.

8. Lorencatto F, Gould NJ, McIntyre SA, During C, Bird J, Walwyn R, et al. A multidimensional approach to assessing intervention fidelity in a process evaluation of audit and feedback interventions to reduce unnecessary blood transfusions: a study protocol. Implementation Science: IS. 2016;11(1):163-.

© 2022 Stanworth SJ et al. *JAMA Network Open*.

eAppendix 2. Sample Reports

a. **PBM2015 Level 1 Key Findings Report**
b. **PBM2015 Level 2 Full Findings Report**

© 2022 Stanworth SJ et al. *JAMA Network Open*.

National Comparative Audit of Blood Transfusion

NHS Blood and Transplant

The Faculty of Intensive Care Medicine

Royal College of Physicians

The Royal College of Anaesthetists

RCS ADVANCING SURGICAL STANDARDS

2015 Audit of Patient Blood Management in adults undergoing elective, scheduled surgery

Hospital Name

KEY FINDINGS REPORT

Our hospital participated in the 2015 audit of PBM in adults undergoing elective,scheduled surgery.

This report provides an overview on how we performed in relation to the keyaudit standards and how we compare to other hospitals nationally.

If you would like **further information** on the findings for these 4 key standards and theother 7 standards, please refer to:

- **'Full Audit Report'**
- **'Supplementary Information Report'**

Who needs to know the results from this audit? Recommended Dissemination List

We should consider sending copies of this feedback report to the following:

- All relevant divisional directors
- All relevant divisional clinical effectiveness leads
- Members of the clinical audit project team (if any)
- Head of nursing
- Clinical audit sponsor
- Medical director
- Clinical audit department (via facilitator for the division)
- Hospital Transfusion Committee and/or Patient Blood Management Committee

Local lead for this audit in your hospital:

How do we compare with other hospitals?

Pre-operative anaemia optimisation (PBM standard 1):

Clinical staff must ensure that patients listed for elective major blood loss surgery have an Hb measured at least 14 days pre-operatively and act upon results*

Our hospital achieved this standard for 29% (6/21) of patients

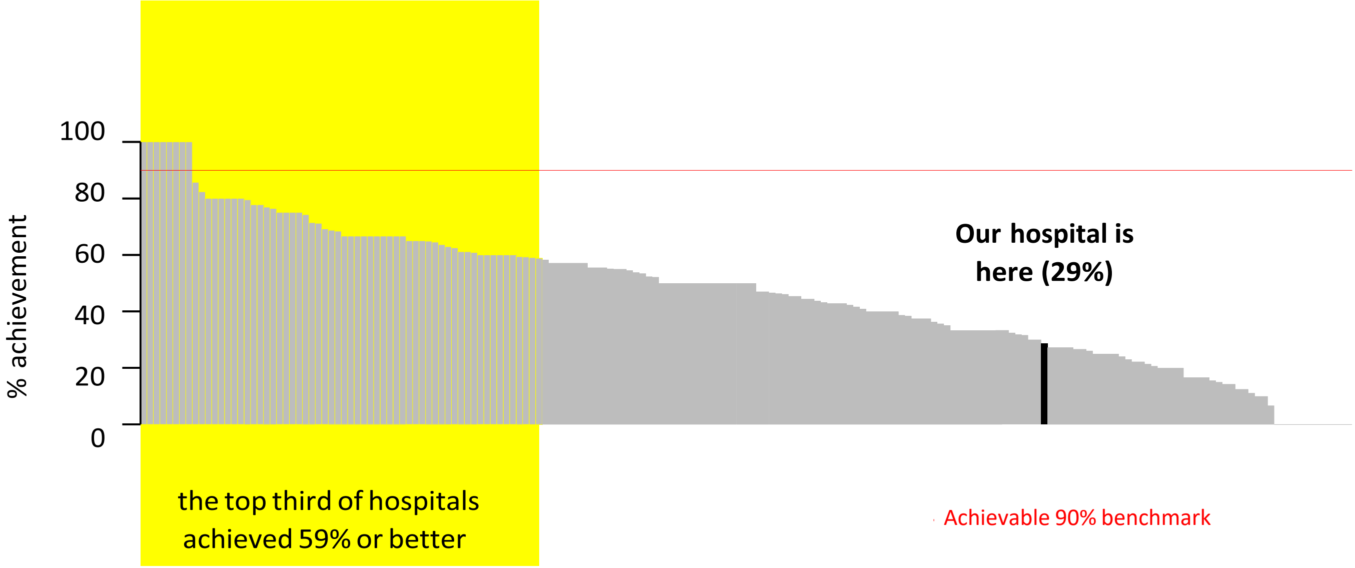

Figure 1: PBM standard 1 hospital comparison. Each chart shows our performance in comparison with the otherparticipating hospitals. The red line illustrates an achievable benchmark of 90%, recognising that a standard of 100% would not be universally attainable.

What should we do next? Recommendations:

For our Hospital	For clinical staff responsible for pre-operative management	For the Hospital Transfusion / Patient Blood Management Committee
• Our performance for this standard was lower than two thirds of the other hospitals nationally. In order to improve the care we provide to our patients, we should prioritise this standard when planning our response to feedback.<br>• We should formulate an action plan to improve performance towards a more feasible short-term goal, such as the national median (i.e. 50%).	• Clinical staff should ensure that **patients are counselled about the relationship between anaemia, morbidity and mortality**, and should be given the **opportunity to defer non-urgent surgery until anaemia** is **investigated** and **treated**.<br>• Clinical staff should **ensure** that **anaemia screening occurs between** the **referral** for **surgery** and **decision to proceed** in order to **allow investigation** and **correction** if appropriate.<br>• Even where surgery is urgent, clinical staff should still **use whatever time is available before operation for anaemia investigation** and **treatment** initiation.	• The Committee should **ensure** that healthcare **pathways** are **structured** to **enable anaemia screening** and **investigation/ correction before surgery**.<br>• The Committee should **work with Commissioners** to **formalise integrated pathways and funding** for the **referral of patients** found to be **anaemic** during surgical workup, if the nature of the anaemia suggests that unexpected significant underlying disease is possible.<br>• The Committee should work with clinicians to **continue monitoring practice in relation to this standard**, by conducting further **local audits** of the number of patients undergoing surgery with anaemia, and **feeding back this information to clinical teams**.

Post-operative transfusion indicated (PBM standard 8):

In patients who do not have active post-operative bleeding, clinical staff should only prescribe a transfusion if the Hb is less than the defined Hb threshold or for transfusion (70g/L in patients without acute coronary ischaemia 80g/L in patients with acute coronary ischaemia).

Our hospital achieved this standard for 27% (6/22) of patients

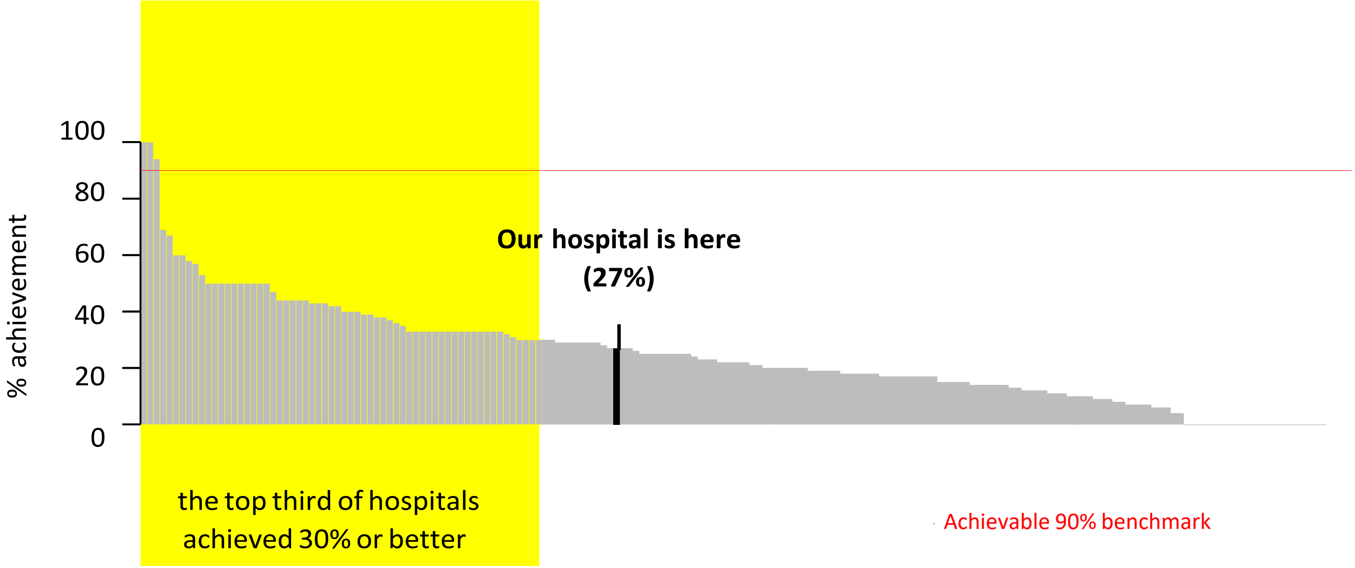

Figure 2: PBM standard 8 hospital comparison

What should we do next? Recommendations:

For our Hospital	For clinical staff making the decision to transfuse	For the Hospital Transfusion / Patient Blood Management team
• Our performance for this standard was in the mid-range when compared with other hospitals nationally, but there is still room for further improvement.<br>• We should formulate an action plan to continue to improve our practice towards achieving this standard.	• Clinical staff should **only prescribe** a **red cell transfusion** in **stable non-bleeding patients** who have a **pre-transfusion Hb** of **less** than **70g/L** or less than **80g/L** in those with **acute coronary syndrome**.<br>• Clinical staff should **record** the **reason** for **transfusion** in the patient's case notes and **record a justification** for transfusion **if** the transfusion was **prescribed for** a patient with **a Hb higher** than the agreed thresholds.	• If a stable non-bleeding patient has a pre-transfusion Hb greater than 80g/L, the transfusion laboratory staff should **query the request prior to issuing blood, with support from Hospital Transfusion / PBM team to do so**.<br>• The team should **work with clinicians to conduct further audits** of the proportions of **patients receiving transfusion outside recommendations**.<br>• The team should consider how best to **work with clinical trainers** to **ensure** that **induction** and **ongoing education programmes** for clinical staff **include randomised trial findings** which compare the patient outcomes of different red cell transfusion strategies.<br>• For hospitals with access to electronic order comms systems, the team should consider how best to **work with** the **IT** department to **design** a **system of decision support** at the time of ordering that **supports best practice**.

Post-operative transfusion – single unit approach (PBM standard 9):

For patients receiving a post-operative transfusion, clinical staff should prescribe one unit of red cells at a time and re-check Hb before prescribing a further unit (unless the patient has active bleeding)

Our hospital achieved this standard for 50% (9/18) of patients

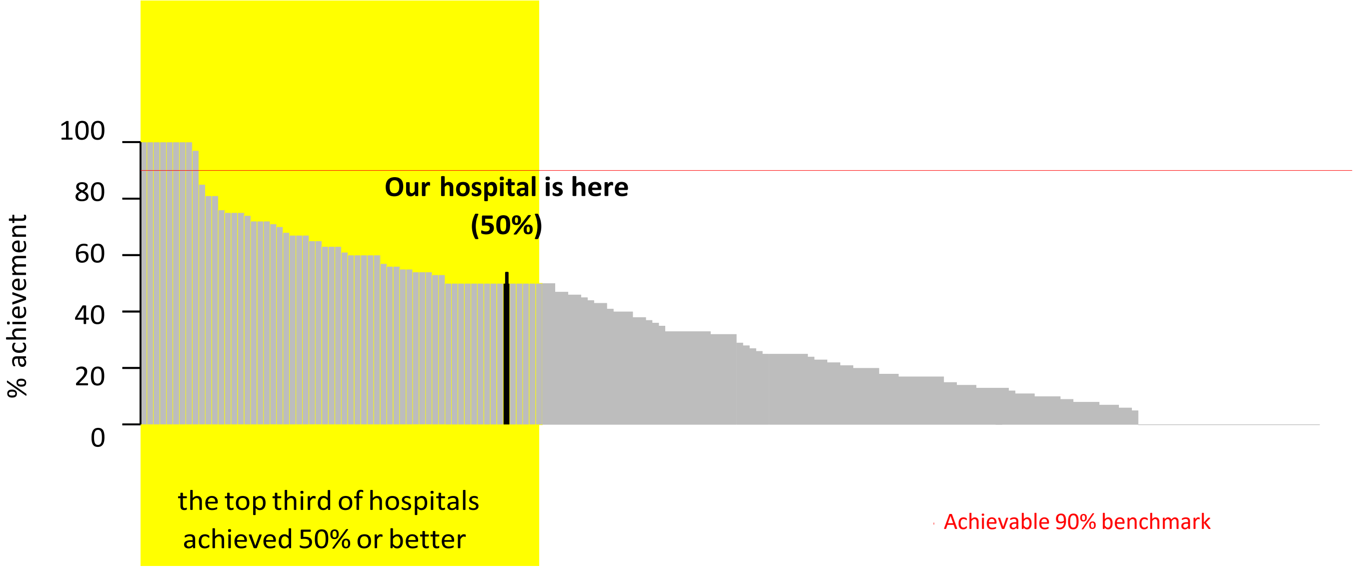

Figure 3: PBM standard 9 hospital comparison

What should we do next? Recommendations:

For our Hospital	For clinical staff making the decision to transfuse	For the Hospital Transfusion / Patient Blood Management team
• Well done. We showed a high level of achievement in this standard. We are performing within the top third of hospitals nationally. This demonstrates strong support for PBM within our hospital. However, there is room to further improve our practice.<br>• We should prepare an action plan that will recognise and build upon our existing good practice to further improve the service that we provide.	• Staff should **recheck Hb after** the **first unit** has been transfused to **see if second** unit can be **avoided**.	• If more than one unit transfusions are being requested for routine pre-operative patients, the laboratory staff should be encouraged to **challenge the request before issuing the blood, with the support of the Hospital Transfusion / PBM team**. This also **strengthens team working** rather than clinicians and lab staff working in "silos".<br>• The Hospital Transfusion / PBM team should work with **clinicians to continue to monitor practice in relation to this standard** by conducting further **local audits** of the proportions of **patients receiving single or more than one unit transfusions**, and **feeding back these findings to clinical teams**.

Patient Blood Management (PBM standard 11):

Clinical staff should attempt all appropriate patient blood management measures in patients who receive a transfusion during major blood loss surgery

Our hospital achieved this standard for 0% (0/15) of patients

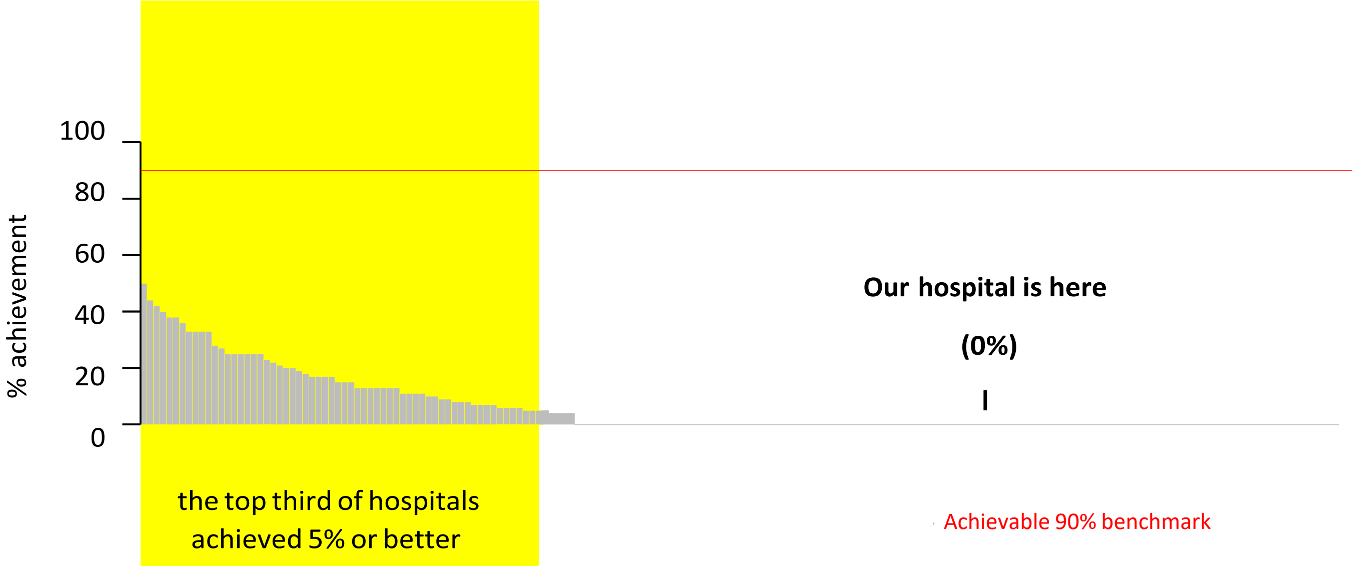

Figure 4: PBM standard 11 hospital comparison

What should we do next? Recommendations:

For our Hospital	For Theatre Staff, Anaesthetists and Surgeons	For the Hospital Transfusion / Patient Blood Management Committee
• Along with most other hospitals, our performance for this standard was lower than expected.<br>• We should formulate an action plan to improve performance towards a more feasible short-term goal, such as 50% working towards 100%.	• The theatre team, anaesthetists and surgeons should ensure that the **PBM measures** identified by the Hospital Transfusion / Patient Blood Management Committee **are implemented as appropriate**.<br>• Where available, **peer data should be applied to compare individual surgeons** and **encourage participation in PBM**.	• The Committee should **ensure that local guidelines exist regarding the use of PBM measures**, including **clear recommendations** on the **individuals** or **teams responsible** for **implementing** these measures.<br>• The Committee should **ensure that the use of tranexamic acid is the** standard of care for **surgical patients** expected to have moderate or more significant blood loss unless contraindicated.<br>• The Committee should **identify the need for intra-operative cell salvage and resource appropriately**; this would normally be used in relevant high blood loss procedures in association with tranexamic acid.

What should we do next? Action Plan

There is evidence that making a specific action plan can facilitate responding to feedback efficiently

- An action plan outlines **what** needs to be done, by **whom**, **where** and **when**.
- We recommend that you pick **two or three recommendations** from the findings report that are important for your hospital to address
- You may find it useful to **complete some or all of this action planning template** when planning your hospital's response to the feedback from this audit

Recommendation from Audit Report	Key Action(s) to be taken	Co-ordinator for Action	Target of Action	Location for Action	Timescale for Action	Indicator of Outcome for Action
Our **selected recommendations(s)** to address	I.e. **WHAT** needs to be done to **address** this recommendation in our hospital?	I.e. **WHO** will be responsible for this action?	I.e. **WHOM** is the action going to affect?	I.e. **WHERE** will this action take place or be discussed?	I.e. **WHEN** will this action be completed (mm/yyyy)?	I.e. **HOW** will the outcome of the action be monitored to ensure it has achieved the desired effect?

Name of individual(s) completing the action plan: Signature: Date:

Name of individual(s) completing the action plan: Signature: Date:

National Comparative Audit of Blood Transfusion

NHS Blood and Transplant

The Faculty of Intensive Care Medicine | Royal College of Physicians | The Royal College of Anaesthetists | RCS Advancing Surgical Standards

2015 Audit of Patient Blood Management in adults undergoing elective, scheduled surgery

Hospital Name

FULL FINDINGS REPORT

- Our hospital participated in the 2015 audit of Patient Blood Management in adults undergoing elective, scheduled surgery.
- Findings from this audit can help us evaluate the quality of our clinical staff's transfusion practice.
- This report **provides full findings on how our hospital performed in relation to the audit standardsand other hospitals nationally**.
- If you would like a summary of the key findings only, please refer to the '***Key Findings***' report.
- If you would like to read additional detail, please refer to the '***Supplementary Information***' report.

Contents

		Page
Section 1	**Why is this audit important?** The rationale for auditing	2–3
Section 2	**Who did we audit?** The characteristics of our audit sample	4–5
Section 3	**How did our hospital perform?** The main findings from the audit in relation to each standard, including a comparison between our hospital's performance and other hospitals nationally and with relevant recommendations for each standard	6–28
Section 4	**What are the Key Priorities?** A summary of the top three priorities identified by the writing group	29
Section 5	**What should we do next?** A suggested action planning template to facilitate our response to feedback	30

Who should we send this report to?

It is recommended that copies of this feedback report may be sent to the following:

- Trust Chief Executive
- Medical Director
- Head of Nursing
- Relevant Divisional Directors
- Divisional Clinical Effectiveness Leads
- Hospital Transfusion Committee and/or Patient Blood Management Committee
- Members of the clinical audit project team
- Clinical Audit Department

Although we recognise that members of the Hospital Transfusion Committee / Patient Blood Management Committee are a key group responsible for implementing change in response to feedback, you may wish to specifically engage with more senior hospital staff in the governance/managerial chain. Senior managementsupport will naturally be important in ensuring sufficient resources are available to implement change.

Local lead for this audit in your hospital:

Section 1: Why is this audit important?

Background

Patient Blood Management (PBM) is evidence-based medicine as applied to transfusion practice, including the treatment and management of pre-operative anaemia, the management of haemostasis andblood conservation. Although effective PBM can lead to more appropriate use of the limited donated blood supply, transfusion avoidance is not the primary goal. Instead, **PBM involves the application of current best evidence to optimise the care and outcomes of all patients who may require transfusion during the course of their care**.[1]

PBM offers the potential for a "win-win" of patient outcome improvements, cost savings, as wellas the public health benefit of reduced demand on donors:[2]

- Transfusion is a life-saving intervention in certain situations where no alternative exists, e.g. exsanguination or marrow failure. However, in most clinical settings, red cell transfusions are administered to patients without active bleeding. Findings from a broad range of randomised controlled trials, including hip fracture surgery, have indicated no evidence of benefit for policies of liberal transfusions (or even in some reports, signals of harm to patients when outcomes are compared to patients receiving restrictive use of blood).[3] **Choosing transfusion as first-line option for treating presumed tissue oxygen deficit in surgical practice may therefore be inappropriate**.
- The donated blood supply is limited and vulnerable to demographic changes as well as the impact of infective pandemics.[4-5] Though the surgical use of blood has decreased over time,[6] **more can and should be done to limit demand on donors**.
- Transfusion is more costly than commonly appreciated,[7] and therefore **minimising use of blood** or **consistent use of alternative management strategies** would be **attractive on financial grounds**.[8-9]
- Transfusion, pre-operative anaemia and acute peri-operative anaemia all carry risk to patients.[10] PBM offers clinicians the opportunity to **prevent their patients getting into situations where decisions must be made as to which of these risks is greater**.

Despite many national[11-12] and international[13-14] recommendations being published in favour of PBM implementation, evidence suggests variability in uptake across the UK. A national survey of organisational arrangements indicated that only the minority of Trusts had adequate time in medical andnursing job plans for PBM, and that many Trusts did not have mechanisms for the reliable use of transfusion alternatives where appropriate.[12]

References

1. *Society for the Advancement of Blood Management. "Professional definition of PBM." Retrieved 28 December, 2014, from www.sabm.org/*
2. *Spahn, D., et al. (2012). "Patient blood management is a win-win: a wake-up call." Br J Anaesth 108: 889-892.*
3. *Murphy, M., et al. (2013). "Transfusing blood safely and appropriately." BMJ, 347: f4304.*
4. *Spahn, D., et al. (2008). "Patient blood management: the pragmatic solution for the problems with blood transfusions." Anesthesiology 109: 951-953.*
5. *Seifried, E., et al. (2011). "How much blood is needed?" Vox Sang 100: 10-21.*
6. *Tinegate, H., et al. (2013). Ten-year pattern of red blood cell use in the North of England.Transfusion, 53: 483-489.*
7. *Abraham, I. and D. Sun (2012). "The cost of blood transfusion in Western Europe as estimated from six studies." Transfusion 52: 1983-1988.*
8. *Spahn, D. (2010). "Anemia and patient blood management in hip and knee surgery: a systematic review of the literature." Anesthesiology 113: 482-495.*
9. *Ejaz, A., et al. (2015). "Potential Economic Impact of Using a Restrictive Transfusion Trigger Among Patients Undergoing Major Abdominal Surgery." JAMA Surg 150: 625-630.*
10. *Kotzé, A., et al. (2015). British Committee for Standards in Haematology Guidelines on the Identification and Management of Pre-Operative Anaemia. British Journal of Haematology. DOI: 10.1111/bjh.13623*
11. *Department of Health. (2007). "Health Service Circular 2007/001." Retrieved 25.3.15 from http://webarchive.nationalarchives.gov.uk/20130107105354/http://www.dh.gov.uk/prod_consum_dh/groups/dh_digitalassets/documents/digitalasset/dh_080803.pdf*
12. *National Blood Transfusion Committee. (2014). "Patient Blood Management: An evidence-based approach to patient care." Retrieved 2.2.15 from http://www.transfusionguidelines.org.uk/uk-transfusion-committees/national-blood-transfusion-committee/patient-blood-management.*
13. *World Health Organisation. (2010). "Sixty-third world health assembly. Agenda Item 11.17: Availability, safety and quality of blood products WHA 63.12." Retrieved 9 September, 2015, from http://apps.who.int/gb/ebwha/pdf_files/WHA63/A63_R12-en.pdf.*
14. *Australian National Blood Authority. (2011). "Patient Blood Management Guidelines Module 2: Peri-operative." Retrieved 9.7.15, from http://www.blood.gov.au/system/files/documents/pbm-module-2.pdf*
15. *Salpeter, S. R., Buckley, J. S., & Chatterjee, S. (2014). Impact of more restrictive blood transfusion strategies on clinical outcomes: a meta-analysis and systematic review. The American journal of medicine, 127(2), 124-131.*
16. *NICE Transfusion guideline in development https://www.nice.org.uk/guidance/indevelopment/gid-cgwave0663/documents accessed 22nd Sept 2015.*

Purpose

This audit was undertaken to document and understand clinical staff's current use of red cell transfusion and PBM approaches in adults undergoing elective, scheduled surgery in relation to eleven audit standards developed by the audit group. The audit is important at a number of levels:

- It **provides national comparative data on PBM practice across the UK**. Surgical blood use has decreased over time as a proportion of total blood use, but no national data is available on the breadth of PBM adoption across the country.
- For hospitals and individual clinicians, it **provides data on how patients are managed along the surgical pathway**. It is nowadays common for multiple clinicians to have input into one patient's care – aggregating data from multiple times (from referral to pre-assessment to surgery and post-operatively) **may inform pathway design and help target improvement programmes**.

PBM measures

The following table illustrates the PBM measures that are appropriate to the index operations.PBM measures are the standard of care for each procedure and ideally all aspects of PBM should have been attempted unless contraindicated or optional.

Table 1: PBM measures appropriate to index operations

**Timing of transfusion**	**Procedure**								
	Primary unilateral / bilateral total hip replacement	Primary unilateral / bilateral and revision total knee replacement	Unilateral revision hip replacement	Surgery for #NOF	Colorectal resection for any indication	Open arterial surgery	• Primary coronary artery bypass graft<br>• Valve replacement +/- CABG	Urological surgery:<br>Cystectomy<br>Nephrectomy	Simple or complex hysterectomy
**Pre-operative**	A	A	A	C	A	A	A	A	A
	B	B	B		B	B	B	B	B
**Intra operative**	A	A	A	D	A	A	A	A	A
	D	D	D		D	D	E	D	D
	G		F			F	F	G	G
**Post-operative**	A	A	A	D	A	A	A	A	A
	D	D	D		D	D	E	D	D
	G	H	F			F	F	G	G
	H		H				H		

PBM Measures Key

A. Pre-operative anaemia optimisation

B. Pre-operative management of patient son anticoagulants and antiplatelet agents

C. Pre-operative management of patients on oral anticoagulants

D. Tranexamic acid

E. Tranexamic acid/aprotinin

F. Intra-operative cell salvage

G. Optional: Intra-operative cell salvage

H. Optional: Post-operative cell salvage

Section 2: Who did we audit?

- This section provides **information on the baseline characteristics of our audited patients,including type of surgery, and key intervals of timing along the surgical pathway**.
- **Characteristics from the national sample of audited patients are presented for comparison purposes**. All of this information can be useful when interpreting our performance in this audit.
- If you would like more detailed information on the clinical and demographic characteristics of our audited patients please refer to the **'*Supplementary Information Report*'**, section '***What are the characteristics of the patients audited?'***

How many patients did we audit?

Our hospital audited 31 adult surgical patients who received a transfusion on one or moreoccasions between 14 days before surgery and up to 7 days post-operatively.

What was the type of surgical procedure audited?

In our hospital, the most common type of surgical procedure was: Arthroplasty for fractured neckof femur, in 32% (10/31) of adult patients (Table 2).

Table 2: Type of surgical procedure audited

	**Our Hospital % N**	**National % N**
Arthroplasty for fractured neck of femur	**(32%) 10**	27% (1044)
Primary unilateral total hip replacement	**(0%) 0**	16% (610)
Primary bilateral total hip replacement	**(0%) 0**	1% (30)
Primary unilateral total knee replacement	**(0%) 0**	9% (341)
Primary bilateral total knee replacement	**(0%) 0**	1% (27)
Unilateral revision hip replacement	**(0%) 0**	7% (258)
Unilateral revision knee replacement	**(0%) 0**	2% (67)
Valve replacement +/− CABG	**(0%) 0**	11% (423)
Primary coronary artery bypass graft	**(0%) 0**	3% (116)
Open arterial surgery	**(16%) 5**	4% (157)
Colorectal resection for any indication	**(13%) 4**	8% (300)
Simple or complex hysterectomy	**(19%) 6**	9% (342)
Nephrectomy	**(10%) 3**	3% (130)
Cystectomy	**(10%) 3**	1% (37)
Not known	**(0%) 0**	0% (15)
**Total**	**31**	100% (3897)

In our hospital, what was the time interval between decision tooperate, the pre-operative assessment and actual surgery?

- This **provides information on our elective pre-operative surgical pathways and helps to identify areas to target** where discrepancies between the audit standards and our current practice are identified.
- If you would like more detailed information on the timings for different surgical procedures please refer to the '***Supplementary Information***' report.

Table 3: Time interval between decision to operate, the pre-operative assessment and actual surgery

	**Our Hospital**	**National**
Patients who had a listing date*	**81% (17/21)**	69% (2679/3897)
Median (IQR) time from listing to surgery in elective patients*	**13 days (4-25), n=17**	42 days (13-93), n=2675
Patients who had a pre-operative assessment visit*	**86% (18/21)**	87% (2446/2818)
Median (IQR) time from pre-operative assessment to surgery*	**14 days (6-20), n=18**	19 days (8-48), n=2420
Patients who had a pre-operative assessment 28 days + before surgery*	**6% (1/18)**	37% (893/2420)

*Excluding patients with fractured neck of femur

Section 3: How did our hospital perform?

Pre-operative anaemia optimisation

PBM standard 1: ***Clinical staff*** *must ensure that patients listed for* ***elective majorblood loss surgery*** *have an* ***Hb measured at least 14 days pre-operatively*** *and****act upon results******

**Anaemia is defined as Hb of less than 130g/L in men less than 120g/L in women*

A pre-operative Hb was taken at least 14 days pre-operatively by clinical staff in 29% (6/21) of patients listed for elective major blood loss surgery compared to in 49% (1386/2838) nationally(patients with fractured neck of femur were excluded).

In relation to pre-operative anaemia optimisation, clinical staff managed patients listed for electivemajor blood loss surgery appropriately in 29% (6/21) of cases compared to in 46% (1305/2836) nationally

- Those with anaemia who have had iron deficiency identified and treated
- Those without anaemia, or those with non-iron deficiency anaemia are not expected to be optimised but meet the standard

How do we compare with other hospitals?

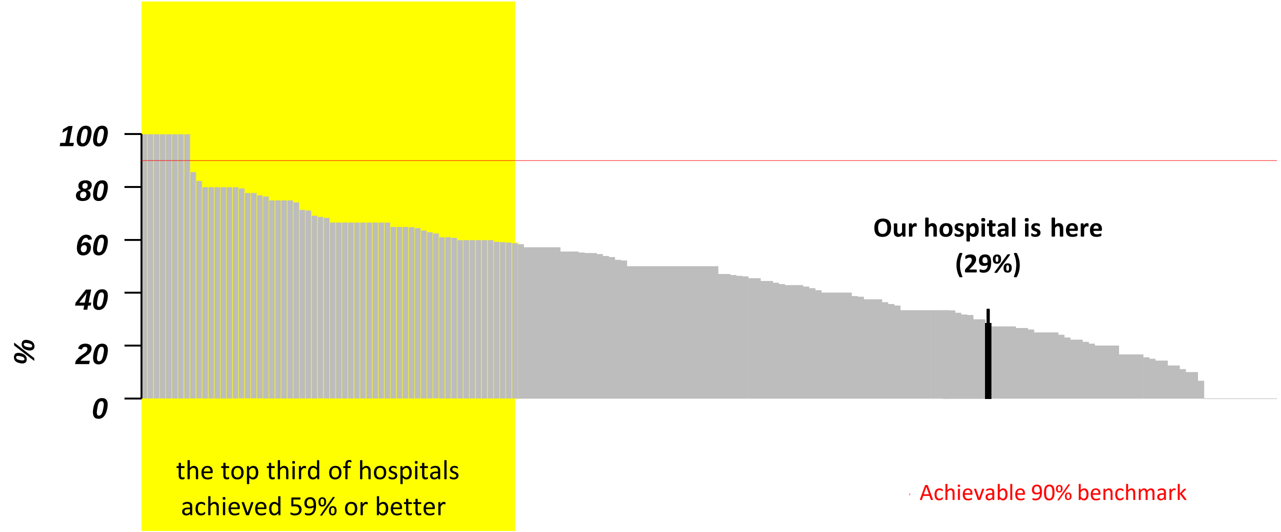

Figure 1: PBM standard 1 hospital comparison. Each chart shows our performance in comparison with the otherparticipating hospitals. The red line illustrates an achievable benchmark of 90%, recognising that a standard of 100% would not be universally attainable.

Why is this standard important?

- The investigation and management of anaemia takes time. Timely Hb testing is thus necessary if patients are not to be:
    - Postponed unnecessarily, at best causing them inconvenience, often distress and sometimes harm OR inappropriately transfused.
- When pre-operative anaemia is discovered during surgical work-up, it should not be seen as simply an abnormal laboratory value. Instead, it should be viewed as:
    - A marker of potential undiagnosed serious disease, for example gastrointestinal cancer or renal failure.
    - A modifiable risk factor for poor surgical outcome.
    - A risk factor to discuss with the patient during the consent process
- Simply proceeding with planned surgery in the face of anaemia is therefore poor medicine.
- On the other hand, if anaemia is only detected close to the time of planned surgery, clinicians caring for the patient peri-operatively are in the invidious situation of having to choose between poor options: proceeding despite the above considerations, or cancelling surgery with its associated waste of resources and burden of morbidity to patients.
- Not detecting anaemia in a timely fashion and/or not managing it appropriately is thus a systemic failure.

Who should we target?

- Figures 2 and table 3 represent the groups of clinical staff responsible for reviewing the pre-operative assessment investigations in our hospital. They should be targeted for PBM standards 1,3 & 5

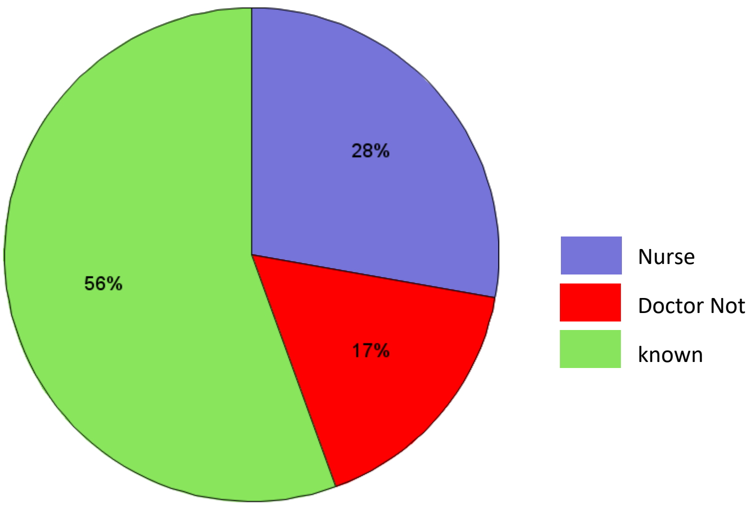

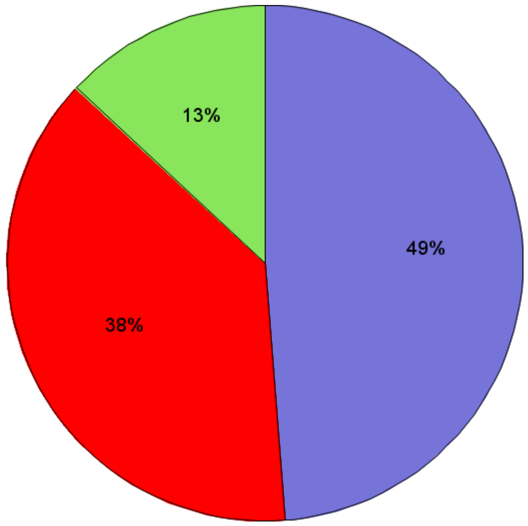

Figure 3: Breakdown between doctors and nurses nationally

	Anaesthetics N	Other specialty N	Specialty not known N
Consultant	0	2	0
Other grade	0	0	1
Speciality not known	0	0	0

Table 4: Grade and specialty of doctor in our hospital

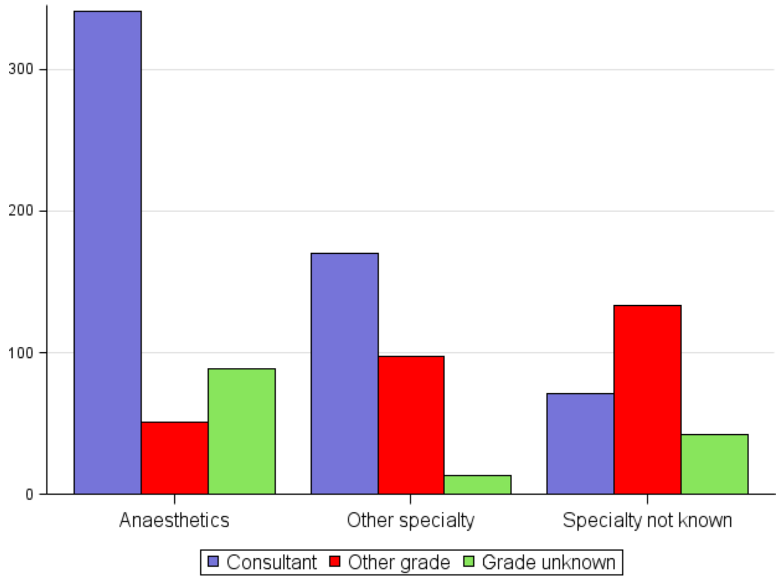

Figure 4: Grade and specialty of doctor nationally

- We should **aim to disseminate feedback to these groups** and to **target these groups for change** where discrepancies between the audit standards and our current practice are identified.

What should we do next? Recommendations:

The following recommendations are in line with the British Committee for Standards in Haematology Guidelines on the Identification and Management of Pre-Operative Anaemia.[10]

For our Hospital	For clinical staff responsible for pre-operative management	For the Hospital Transfusion / Patient Blood Management Committee
• Our performance for this standard was lower than two thirds of the other hospitals nationally. In order to improve the care we provide to our patients, we should prioritise this standard when planning our response to feedback.<br>• We should formulate an action plan to improve performance towards a more feasible short-term goal, such as the national median (i.e. 50%).	• Clinical staff should ensure that **patients are counselled about the relationship between anaemia, morbidity and mortality**, and should be given the **opportunity to defer non-urgent surgery until anaemia** is **investigated** and **treated**.<br>• Clinical staff should **ensure** that **anaemia screening occurs between** the **referral** for **surgery** and **decision to proceed** in order to **allow investigation** and **correction** if appropriate.<br>• Even where surgery is urgent, clinical staff should still **use whatever time is available before operation for anaemia investigation** and **treatment** initiation.	• The Committee should **ensure** that healthcare **pathways** are **structured** to **enable anaemia screening** and **investigation/ correction before surgery**.<br>• The Committee should **work with Commissioners** to **formalise integrated pathways and funding** for the **referral of patients** found to be **anaemic** during surgical workup, if the nature of the anaemia suggests that unexpected significant underlying disease is possible.<br>• The Committee should work with clinicians to **continue monitoring practice in relation to this standard**, by conducting further **local audits** of the number of patients undergoing surgery with anaemia, and **feeding back this information to clinical teams**.

Pre-operative transfusion indicated

PBM standard 2: ***Clinical staff*** *should only* ***prescribe a pre-operative transfusion*** *in* ***patients*** *undergoing* ***elected major blood loss surgery*** *if the* ***Hb is less than thedefined Hb threshold for transfusion (70g/L in patients without acute coronary ischaemia or 80g/L in patients with acute coronary ischaemia)***

- A pre-operative transfusion was prescribed by clinical staff in **10% (3/31)** of our patients compared to 7% (279/3793) nationally.
- The pre-operative transfusion was prescribed when the Hb was less than the defined Hb threshold for transfusion in **100% (2/2)** of our patients compared to 12% (28/242) nationally (patients with fractured neck of femur excluded).

How do we compare with other hospitals?

- Only 28 patients met this standard across all hospitals audited. **The majority of cases did not meet this standard because they were prescribed a pre-operative transfusion when the Hb was more than the defined threshold for transfusion**.
- In our patients, the median (IQR) Hb prior to transfusion was **68 days (68-68), n=3**, compared to 82 days (76-89), n=267 nationally.
- Figure 5 shows the distribution of results for pre-transfusion Hb in pre-operative patients nationally. **This suggests that many patients are being transfused above the recommended threshold**.

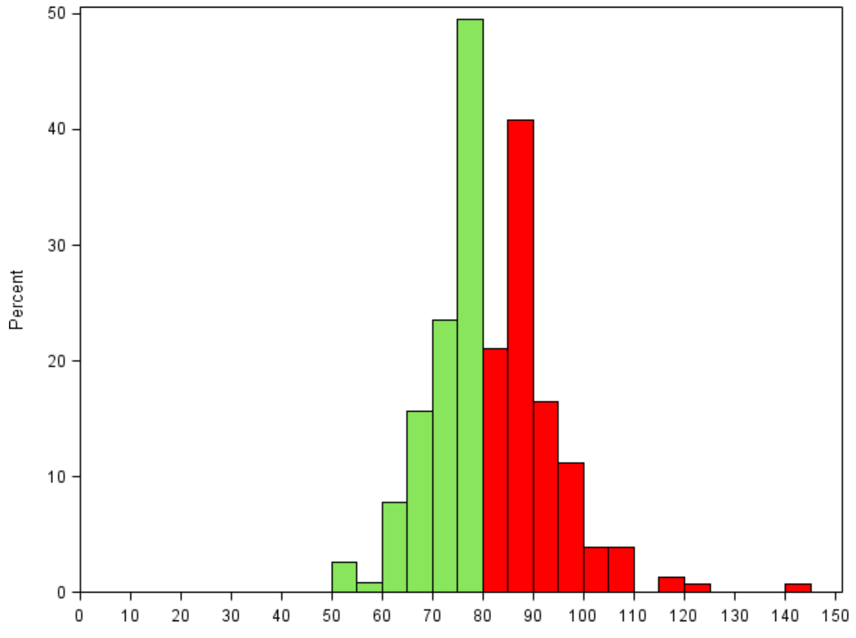

Figure 5: Pre-transfusion Hb in pre-operative patients nationally

Why is this standard important?

- There is an increasing body of evidence from large randomised controlled trials that there is no benefit for transfusing at higher haemoglobin thresholds (liberal practice), and some evidence of harm.[15]
- The use of a restrictive transfusion strategy is therefore recommended. This reduces unnecessary transfusion of red cells, improving outcomes for patients and also reducing costs.
- A higher transfusion threshold of 80g/L is suggested for patients with acute coronary syndrome, given uncertainty about the levels of evidence for this subgroup.

Who should we target?

- Table 5 shows who was involved in the decision to transfuse pre-operatively in our hospital. They should be targeted for standards 1,3 & 4.
- Figure 6 shows breakdown of specialities and grade of doctor nationally (NB there was only 1 nurse in the whole dataset).

	Anaesthetics N	Other specialty N	Specialty not known N
Consultant	0	2	0
Senior Trainee	0	0	0
Junior Trainee	0	0	1
Grade not known	0	0	0

Table 5: Grade and specialty of doctor in our hospital

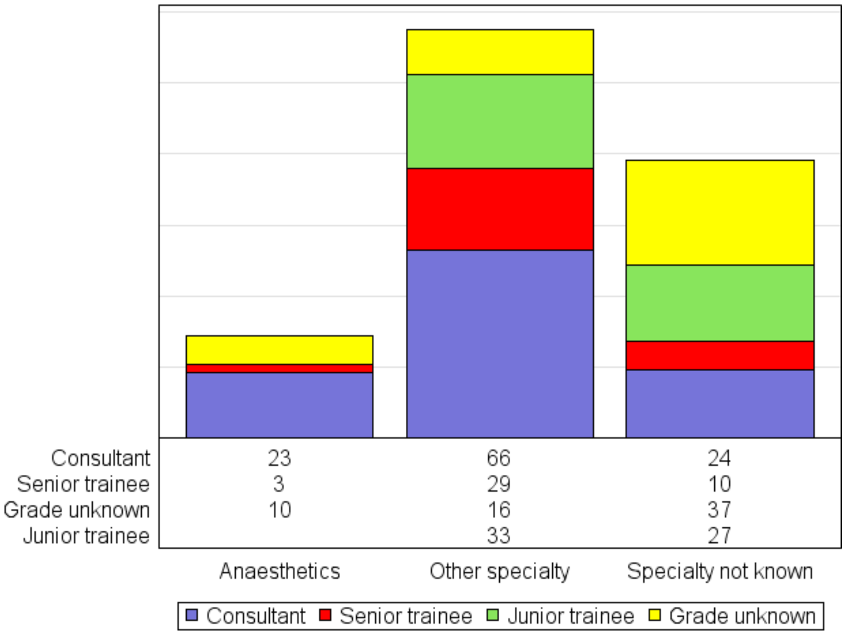

Figure 6: Grade and specialty of doctor nationally

What should we do next? Recommendations:

For our Hospital	For clinical staff responsible for pre-operative management	For the Hospital Transfusion / Patient Blood Management Committee
• Along with most other hospitals, **our performance for this standard was lower than expected**. In order to improve the care we provide to our patients, we **should prioritise this standard** when planning our response to feedback.<br>• We should formulate an action plan to **improve performance towards** a more feasible **short-term goal**, such as 50% working towards 100%.	• Clinical staff should **ensure** that **anaemia screening takes place when referral for surgery is first made** in order to **allow investigation** and **correction** if appropriate.<br>• Even where surgery is urgent, clinical staff should still **use whatever time is available before operation** for **anaemia investigation** and **treatment** initiation.<br>• Clinical staff should ensure that **patients are counselled** about the **relationship** between **anaemia**, **morbidity** and **mortality**, and should be given the **opportunity** to **defer non-urgent surgery** until **anaemia** is **investigated** and **treated** rather than having a blood transfusion to correct the anaemia.<br>• Clinical staff who prescribe blood should be made **aware** of the **findings** from randomised controlled **trials** which compare different strategies of red cell transfusions for patient outcomes and **which indicate a lack of evidence to support any benefit from liberal use of red cells**.<br>• Clinical staff should **only prescribe** a blood **transfusion in stable non-bleeding patients** who have a **pre-transfusion Hb** of **less** than **70g/L**, or less than **80g/L** in those with **acute coronary syndrome**.	• The Committee should ensure that **healthcare pathways** are **structured** to **enable anaemia screening** and **investigation / correction** before surgery.<br>• The Committee should have a **policy** in place to **ensure** that **guidelines promote** that **stable non-bleeding patients are not transfused** with a pre-transfusion **Hb** of **more** than **70g/L** or more than **80g/L** in those with acute **coronary syndrome**.

Pre-operative transfusion indicated only if pre-operative anaemia optimisation has been attempted

PBM standard 3: ***Clinical staff*** *should* ***only prescribe a pre-operative transfusion****in* ***patients*** *undergoing* ***elective major blood loss surgery*** *if the* ***Hb*** *is less than the* ***defined Hb threshold for transfusion*** ***and*** ***pre-operative anaemia optimisation has been attempted***

- The pre-operative transfusion was prescribed when the Hb was less than the defined Hb threshold for transfusion in **0% (0/2)** of our patients in whom pre-operative anaemia optimisation had been attempted compared to 2% (3/132) nationally. *(Patients with fractured neck of femur were excluded from this standard)*.

How do we compare with other hospitals?

- Only 3 patients met the standards across all across all hospitals audited. **The majority of cases failed this standard because they were transfused without an attempt to investigate or correct the anaemia and/or they were transfused above the defined Hb threshold**.

Why is this standard important?

- Transfusion should not be seen as an alternative to good practice as outlined in PBM standard 1 where pre-operative anaemia is proactively managed.
- Transfusion should only be considered in patients where the anaemia is not correctable and the Hb is below the defined threshold.
- There is no evidence from a broad randomised trial literature that liberal use of red cell transfusions is a superior policy for patients
- It thus indicates failed care processes if clinicians have to resort to transfusion because opportunities for investigation and evidence-based anaemia treatment have been missed.

What should we do next? Recommendations:

For our Hospital	For clinical staff responsible for pre-operative management	For the Hospital Transfusion / Patient Blood Management Committee
• Along with most other hospitals, **our performance for this standard was lower than expected**. In order to improve the care we provide to our patients, we **should prioritise this standard** when planning our response to feedback.<br>• We should formulate an action plan to **improve performance towards** a more feasible **short-term goal**, such as 50% working towards 100%.	• Clinical staff should **ensure** that **anaemia screening takes place when referral for surgery is first made** in order to **allow investigation** and **correction** if appropriate.<br>• Even where surgery is urgent, clinical staff should still **use whatever time is available before operation** for **anaemia investigation** and **treatment** initiation.<br>• Clinical staff should ensure that **patients are counselled** about the **relationship** between **anaemia**, **morbidity** and **mortality**, and should be given the **opportunity** to **defer non-urgent surgery** until **anaemia** is **investigated** and **treated** rather than having a blood transfusion to correct the anaemia.<br>• Clinical staff who prescribe blood should be made **aware** of the **findings** from randomised controlled **trials** which compare different strategies of red cell transfusions for patient outcomes and **which indicate a lack of evidence to support any benefit from liberal use of red cells**.<br>• Clinical staff should **only prescribe** a blood **transfusion in stable non-bleeding patients** who have a **pre-transfusion Hb** of **less** than **70g/L**, or less than **80g/L** in those with **acute coronary syndrome**.	• The Committee should ensure that **healthcare pathways** are **structured** to **enable anaemia screening** and **investigation / correction** before surgery.<br>• The Committee should have a **policy** in place to **ensure** that **guidelines promote** that **stable non-bleeding patients are not transfused** with a pre-transfusion **Hb** of **more** than **70g/L** or more than **80g/L** in those with acute **coronary syndrome**.

Pre-operative transfusion – single unit approach

PBM standard 4: *For **patients** receiving a **pre-operative transfusion**, **clinical staff** should **prescribe one unit of red cells at a time** and **re-check Hb before prescribing a further unit***

- The single unit transfusion approach was followed by clinical staff in **0% (0/2)** of our pre-operative patients, compared to 28% (71/253) nationally.

How do we compare with other hospitals?

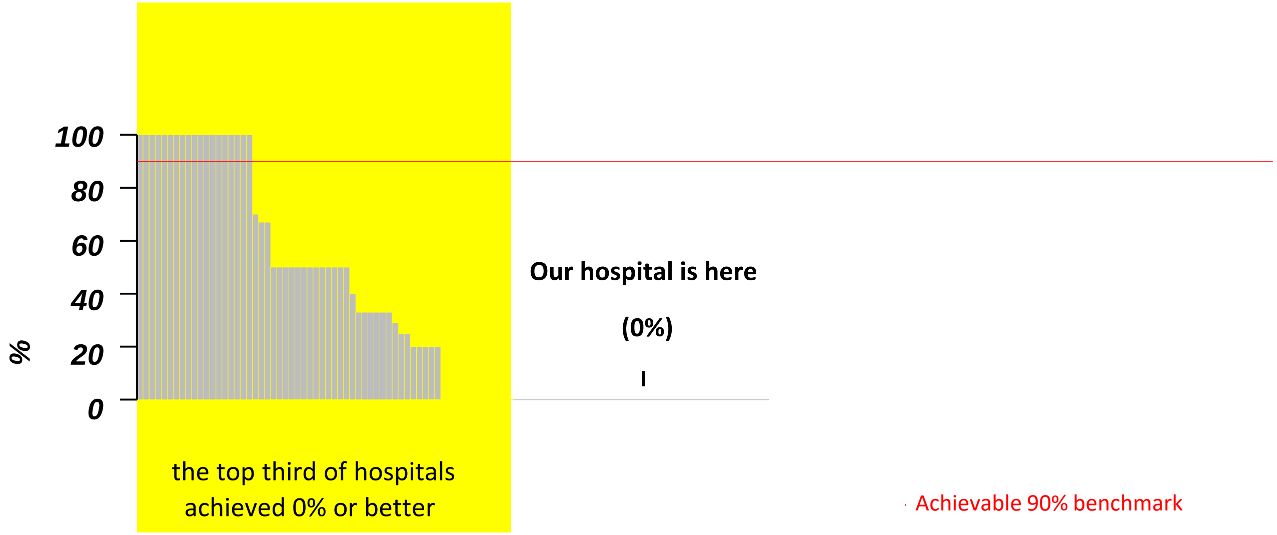

Figure 7: PBM standard 4 hospital comparison

- Nationally 80% (221/277) of patients who were transfused preoperatively received **2 or more units** of blood. In our hospital this figure was **100% (3/3)**.

Why is this standard important?

- A key component of PBM is to avoid the unnecessary transfusion or over-transfusion of blood.
- Good PBM requires all decisions to transfuse to be based upon a recent Hb.
- By checking a patient's Hb after the first unit, it will become evident that in many instances, the second unit is not required.
- Reducing number of units transfused reduces all aspects of transfusion related risk.
- Re-checking Hb after the first unit is likely to save time, effort and money.
- Also reduces risk of cancellation and urgent correction of low Hb at time of surgery.

What should we do next? Recommendations:

For our Hospital	For clinical staff making the decision to transfuse	For the Hospital Transfusion / Patient Blood Management team
• Along with two thirds of other hospitals, our performance for this standard was lower than expected. In order to improve the care we provide to our patients, we should prioritise this standard when planning our response to feedback.<br>• We should formulate an action plan to improve performance towards a more feasible short-term goal, such as 50% working towards 100%.	• Staff should **recheck Hb after** the **first unit** has been transfused to **see if second** unit can be **avoided**.	• If more than one unit transfusions are being requested for routine pre-operative patients, the laboratory staff should be encouraged to **challenge the request before issuing the blood, with the support of the Hospital Transfusion / PBM team**. This also **strengthens team working** rather than clinicians and lab staff working in "silos".<br>• The Hospital Transfusion / PBM team should **work with clinicians to continue to monitor practice in relation to this standard by conducting further local audits** of the proportions of patients receiving single or more than one unit transfusions, and **feeding back these findings to clinical teams**.

Pre-operative anticoagulant and antiplatelet management

PBM standard 5:

- *For **patients** undergoing **elective major blood loss surgery** who are taking **oral anticoagulants and/or antiplatelet agents**, **clinical staff** must **stop** the **oral anticoagulant** and/or **antiplatelet agent(s)** at least **5 days** pre-operatively (unless there are good reasons to continue) and **document the management plan** in the case notes*
- *For patients with **fractured neck of femur** taking **warfarin**, **clinical staff** should aim **for an INR of less than 1.5** on the day before or the day of surgery*

- The algorithm used to assess compliance includes both elements of the standard and is therefore reported as a composite standard in the chart below.
- At our hospital, **10% (3/31)** of patients were on oral anticoagulants or antiplatelet agents pre-operatively, compared to 18% (710/3890) nationally.
- Clinical staff stopped oral anticoagulation at least 5 days pre-operatively in **33% (1/3)** of our patients compared to 87% (315/361) nationally.
- Clinical staff stopped antiplatelet agents at least 5 days pre-operatively (or continued the therapy with documented good reason) in in **0% (0/0)** of our patients compared to 77% (279/363) nationally.
- The INR was less than 1.5 on the day of or the day before surgery in **67% (2/3)** of our patients undergoing surgery for fractured neck of femur compared to 70% (214/306) nationally.
- The algorithm used to assess compliance includes both elements of the standard and is therefore reported as a composite standard in the chart below. At our hospital **50% (1/2)** met this composite standard, compared to 63% (340/541) nationally.

How do we compare with other hospitals?

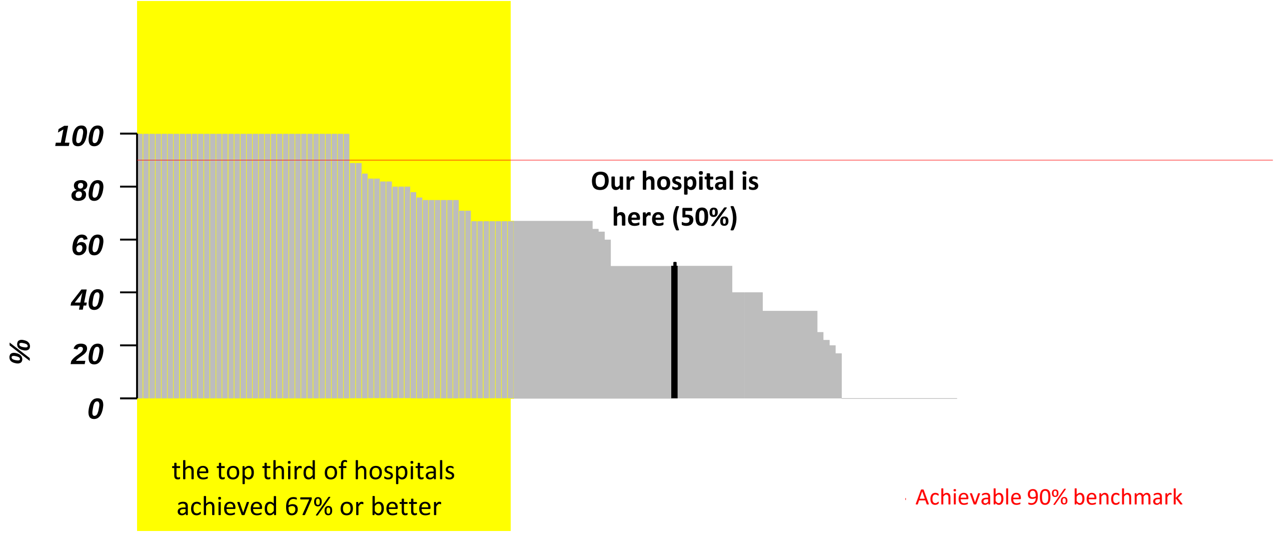

Figure 8: PBM standard 5 hospital comparison

Why is this standard important?

- Patients who do not have their oral anticoagulation or anti platelet medications withheld for at least 5 days before major elective surgery are likely to be at increased risk of bleeding during surgery (does not include aspirin).
- To reduce the risk of bleeding, patients with fractured neck of femur on warfarin should have their anticoagulation actively managed so that their INR is 1.5 or less on the day before or the day of surgery.
- The risk of bleeding versus the risk of thrombosis should be considered for each patient and an individualised management plan should be developed.

What should we do next? Recommendations:

For our Hospital	For clinical staff responsible for pre-operative management	For the hospital thrombosis committee (or equivalent)
• Our performance for this standard was in the mid-range when compared with other hospitals nationally, but there is still room for further improvement.<br>• We should formulate an action plan to continue to improve our practice towards achieving this standard.	• Clinical staff should **prepare an individual management plan** for **each patient undergoing major surgery** who is on **oral anticoagulation** or **antiplatelet therapy** and this should be **documented** in the case notes. For patients with **fractured neck of femur, surgery should not be delayed**.	• The Committee should **prepare** clear **guidelines** for the **perioperative** management of **anticoagulation** and **antiplatelet agents** and ensure that **systems** are in place **to deliver this** standard of care.<br>• The Committee should **regularly audit the management of patients on anticoagulation** or **anti-platelet therapy** to **ensure** that the **guidelines** are being **followed**.

Patient Blood Management in patients who have received anintra-operative transfusion

Clinical staff** should attempt **at least one** **(PBM standard 6)** **or all** **(PBM standard 7)** **appropriate patient blood management measures** in patients who receive a **transfusion** during **major blood loss surgery

- Clinical staff prescribed intra-operative transfusion in **37% (11/30)** of our patients undergoing major blood loss surgery, compared to 25% (982/3851) nationally
- Of those patients who received an intra-operative blood transfusion, clinical staff attempted **at least one** appropriate PBM in **100% (5/5)** of our patients undergoing major blood loss surgery, compared to 83% (661/795) nationally
- Of those patients who received an intra-operative blood transfusion, clinical staff attempted **all** appropriate PBM in **17% (1/6)** of our patients undergoing major blood loss surgery, compared to 16% (133/808) nationally

How do we compare with other hospitals?

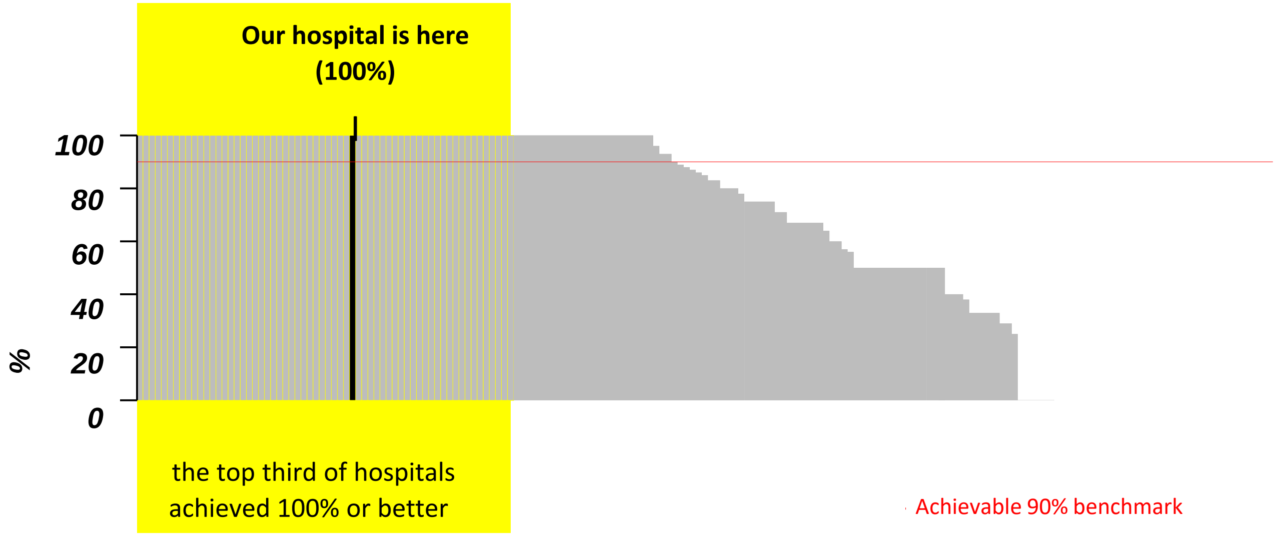

Figure 9: PBM standard 6 hospital comparison

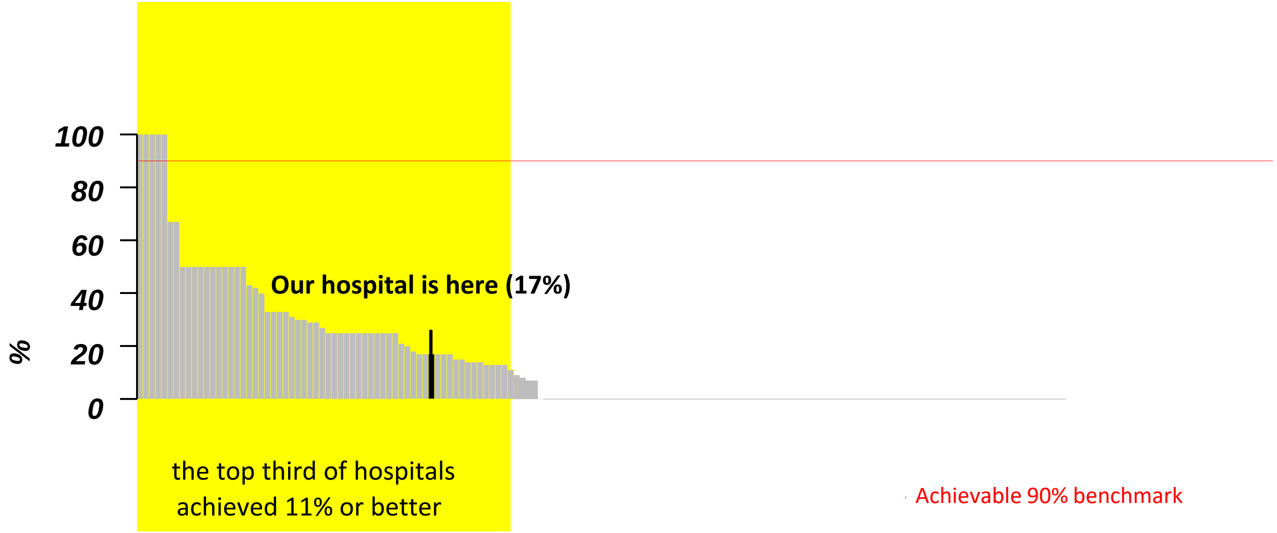

Figure 10: PBM standard 7 hospital comparison

Why is this standard important?

- The over-arching purpose of PBM is to improve outcomes for patients.
- The components of PBM as described in Table 1 combine to minimise the need for blood transfusion.
- PBM can lead to reduced complications, hospital stay and the cost of treating complications.
- PBM Standard 6 relates to at least one PBM intervention being implemented. This is an easy standard to comply with and really corresponds to the basics of good peri-operative practice.
- PBM standard 7 is the most stringent. It reflects the very best practice, where hospitals have processes in place to ensure that every possible PBM intervention has been implemented to the benefit of patients.
- Hospitals should not be satisfied with high achievement in standard 6, but rather, should strive to increase their achievement for standard 7 which represents best PBM practice.

What should we do next? Recommendations:

For our Hospital	For theatre teams	For the Hospital Transfusion / Patient Blood Management team
**For PBM Standard 6:**<br>• Well done. We showed a high level of achievement in this standard. We are performing within the top third of hospitals nationally. This demonstrates strong support for PBM within our hospital. However, there is room to further improve our practice.<br>• We should prepare an action plan that will recognise and build upon our existing good practice to further improve the service that we provide.<br>**For PBM Standard 7:**<br>• Well done. We showed a high level of achievement in this standard. We are performing within the top third of hospitals nationally. This demonstrates strong support for PBM within our hospital. However, there is room to further improve our practice.<br>• We should prepare an action plan that will recognise and build upon our existing good practice to further improve the service that we provide.	• The theatre team, anaesthetists and surgeons should **ensure that the PBM measures** (presented in Table 1) and as identified by the Hospital Transfusion / Patient Blood Management Committee **are implemented as appropriate**.<br>• Where available, **peer data should be applied to compare individual surgeons** and **encourage participation in PBM**.	• The Committee should **ensure that local guidelines exist regarding the use of PBM measures** (as outlined in Table 1), including **clear recommendations** on the **individuals or teams responsible for implementing these measures**.<br>• The Committee should **ensure** that the **use of tranexamic acid is the standard of care for surgical patients** expected to have moderate or more significant blood loss unless contraindicated.<br>• The Committee should **identify the need for intra-operative cell salvage and resource appropriately**; this would normally be used in relevant high blood loss procedures in association with tranexamic acid.

Post-operative transfusion indicated

PBM standard 8: *In* ***patients*** *who* ***do not have active post-operative bleeding***, ***clinical staff*** *should only* ***prescribe a transfusion*** *if the* ***Hb is less than the definedHb threshold or*** *for transfusion (70g/L in patients without acute coronary ischaemia 80g/L in patients with acute coronary ischaemia)*

- At least one post-operative transfusion was prescribed by clinical staff in **71% (22/31)** of our patients compared to 74% (2878/3874) nationally and the analysis has been undertaken on the first post-operative transfusion episode
- The post-operative transfusion was prescribed by clinical staff for documented active bleeding or when the Hb was less than the defined Hb threshold for transfusion in **27% (6/22)** of our patients compared to 24% (669/2757) nationally.

How do we compare with other hospitals?

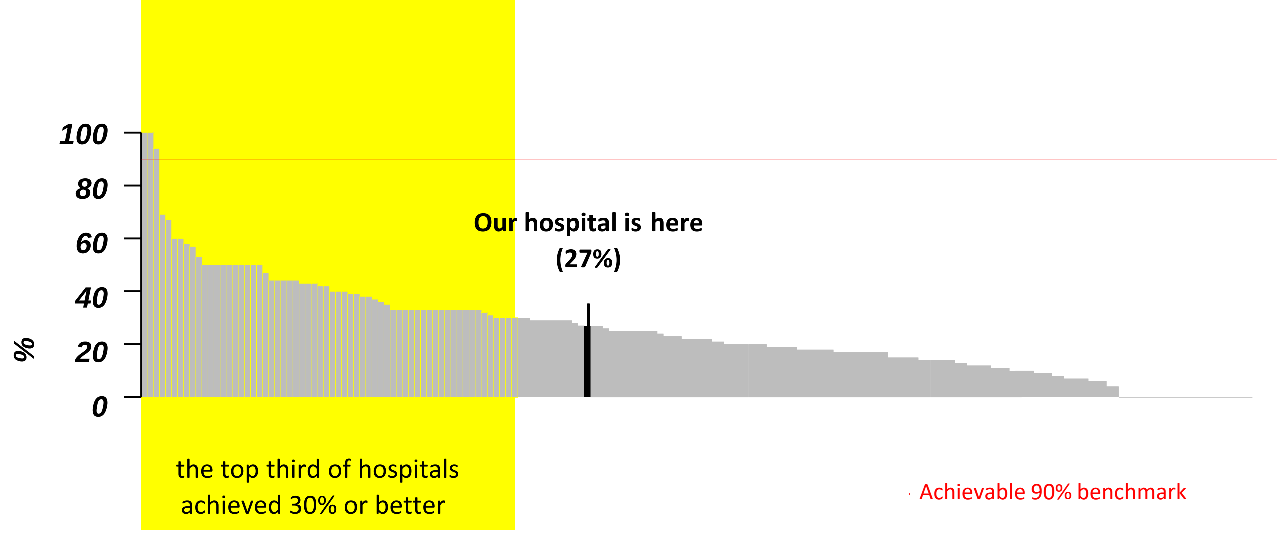

Figure 11: PBM standard 8 hospital comparison

- Table 6 shows the number of transfusion episodes nationally in the first 7 post-operative days. 80% of patients (2307/2878) were transfused on just one occasion.

Table 6: Number of transfusion episodes nationally in first 7 post-operative days

Transfusion	74% (2878/3874)
How many episodes*	
One	80% (2307/2878)
Two	13% (360/2878)
Three	2% (62/2878)
Four	<1% (10/2878)
Five	<0.1% (2/2878)
Not known	5% (137/2878)

* A transfusion episode = any red cells transfused within a 24 hour period

- In our patients, the median (IQR) Hb prior to transfusion was **79 days (72-84), n=22** , compared to 79 days (74-85), n=2717 nationally.

- Figure 12 shows the distribution of Hb results prior to the first post-operative transfusion episode nationally. This suggests that many patients are being transfused above the recommended threshold.

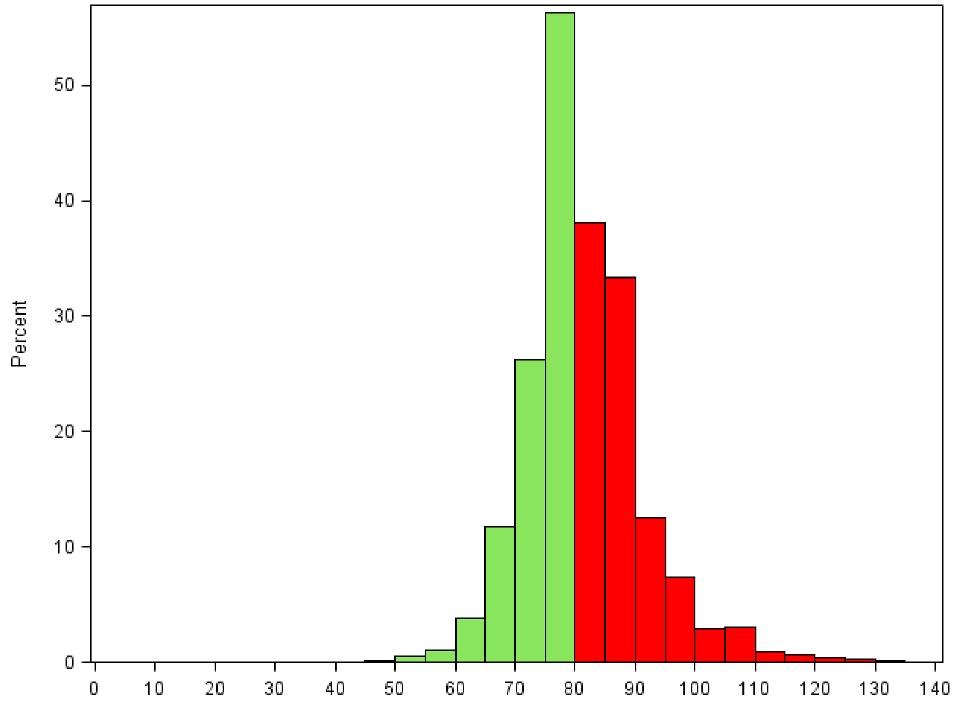

Figure 12: Distribution of Hb results prior to the first post-operative transfusion episode nationally

- Table 7 shows the reasons for the first post-operative transfusion nationally.

Table 7: Reasons for first post-operative transfusion nationally

Active bleeding*	9% (256/2868)
An hb <70 g/L without acute coronary syndrome	11% (304/2868)
An hb < 80 g/L with acute coronary syndrome	5% (134/2868)
Other**	74% (2115/2868)
Low BP or other hemodynamic reason	19% (394/2115)
Hb drop	59% (1242/2115)
Blood loss – any volume recorded	5% (107/2115)
Not known	18% (372/2115)
Not known	2% (59/2868)

* Active post-operative bleeding is defined as bleeding causing systolic Hb <90mmHg, and or heart rate >110bpm, and or return to theatre because of bleeding and or activation of major haemorrhage pathway.

** These categories were formed from free-text stated by auditors

Why is this standard important?

- There is an increasing body of evidence from large randomised controlled trials, that there is no benefit for transfusing at higher haemoglobin thresholds (liberal practice), and some evidence of harm.[15]
- The use of a restrictive transfusion strategy is therefore recommended. This reduces unnecessary transfusion of red cells, improving outcomes for patients and also reducing costs.
- A higher transfusion threshold of 80g/L is recommended for those with acute coronary syndrome, given uncertainty about the levels of evidence for this subgroup.

Who do we target?

- Table 8 shows which clinical staff were involved in the decision to transfuse post-operatively. These staff should also be targeted for standard 9.
- Figure 13 shows the breakdown of specialities and grade of staff nationally.

	Anaesthetics N	Other specialty N	Specialty not known N
Consultant	0	0	1
Senior trainee	0	0	1
Junior trainee	0	0	11
Speciality not known	3	0	6

Table 8: Grade and specialty of doctor in our hospital

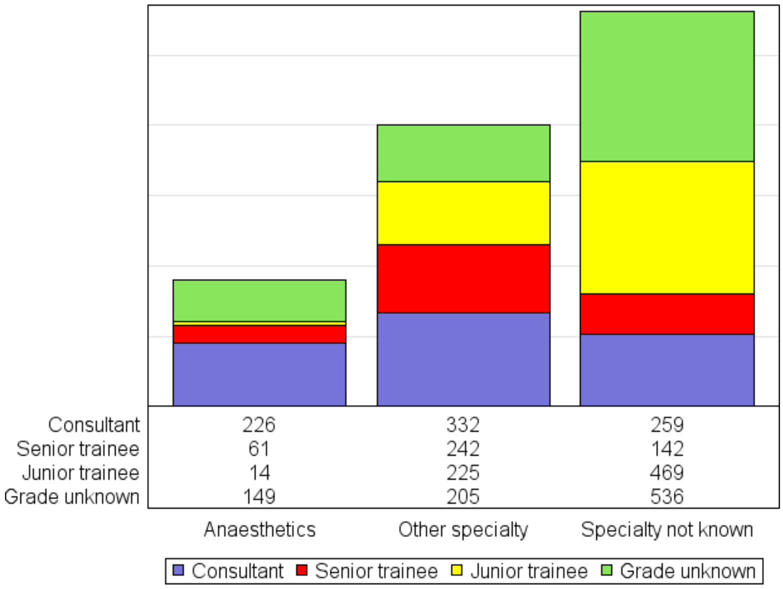

Figure 13: Grade and specialty of doctor nationally

What should we do next? Recommendations:

For our Hospital	For clinical staff making the decision to transfuse	For the Hospital Transfusion / Patient Blood Management team
• Our performance for this standard was in the mid-range when compared with other hospitals nationally, but there is still room for further improvement.<br><br>• We should formulate an action plan to continue to improve our practice towards achieving this standard.	• Clinical staff should **only prescribe** a **red cell transfusion** in **stable non-bleeding patients** who have a **pre-transfusion Hb** of **less** than **70g/L** or less than **80g/L** in those with **acute coronary syndrome**.<br><br>• Clinical staff should **record** the **reason** for **transfusion** in the patient's case notes and **record a justification** for transfusion **if** the transfusion was **prescribed for** a patient with **a Hb higher** than the agreed thresholds.	• If a stable non-bleeding patient has a pre-transfusion Hb greater than 80g/L, the transfusion laboratory staff should **query the request prior to issuing blood, with support from Hospital Transfusion / PBM team to do so**.<br><br>• The team should **work with clinicians to conduct further audits** of the proportions of **patients receiving transfusion outside recommendations**.<br><br>• The team should consider how best to **work with clinical trainers** to **ensure** that **induction** and **ongoing education programmes** for clinical staff **include randomised trial findings** which compare the patient outcomes of different red cell transfusion strategies.<br><br>• For hospitals with access to electronic order comms systems, the team should consider how best to **work with** the **IT** department to **design** a **system of decision support** at the time of ordering that **supports best practice**.

Post-operative transfusion – single unit approach

PBM standard 9: *For **patients** receiving a **post-operative transfusion**, **clinical staff** should **prescribe one unit of red cells at a time** and re-check **Hb before prescribinga further unit (unless the patient has active bleeding)***

- For the first post-operative transfusion episode, the single unit transfusion approach was followed by clinical staff in **50% (9/18)** of our post-operative patients compared to 38% (920/2414) nationally (patients with active bleeding were excluded).

How do we compare with other hospitals?

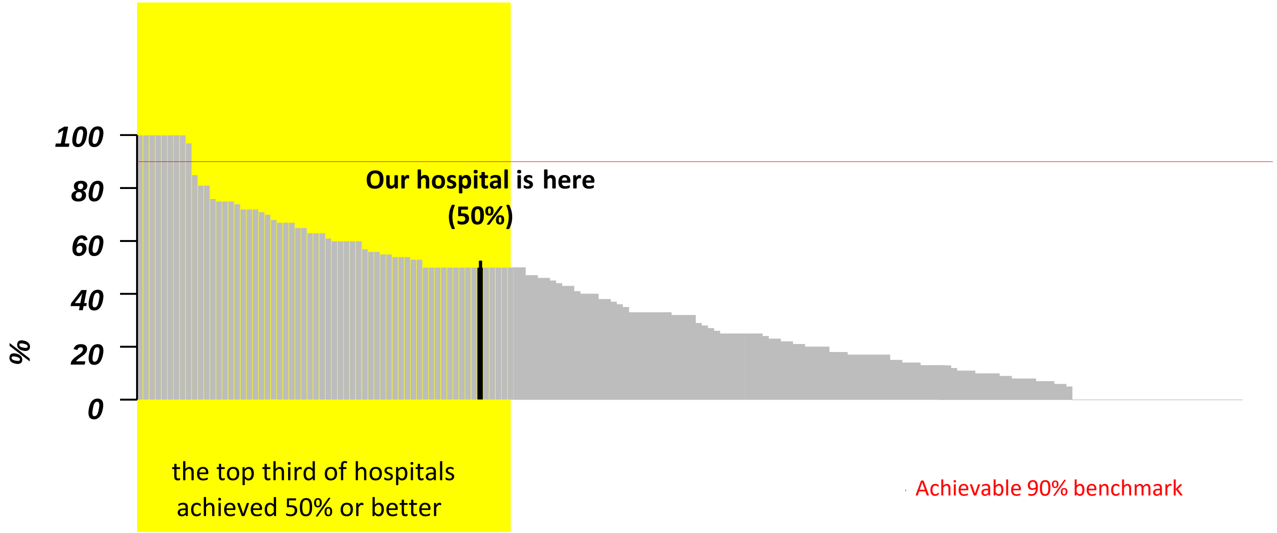

Figure 14: PBM standard 9 hospital comparison

- Nationally 69% (1950/2842) of patients who were transfused post-operatively received **2 or more units** of blood. In our hospital this figure was **59% (13/22)**.

Why is this standard important?

- A key component of PBM is to avoid the unnecessary transfusion or over-transfusion of blood.
- Good PBM requires all decisions to transfuse to be based upon a recent Hb.
- By checking a patient's Hb after the first unit, it will become evident that in many instances, the second unit is not required.
- Reducing number of units transfused reduces all aspects of transfusion related risk.
- Re-checking Hb after the first unit is likely to save time, effort and money.

What should we do next? Recommendations:

For our Hospital	For clinical staff making the decision to transfuse	For the Hospital Transfusion / Patient Blood Management team
• Well done. We showed a high level of achievement in this standard. We are performing within the top third of hospitals nationally. This demonstrates strong support for PBM within our hospital. However, there is room to further improve our practice.<br>• We should prepare an action plan that will recognise and build upon our existing good practice to further improve the service that we provide.	• Staff should **recheck Hb after** the **first unit** has been transfused to **see if second** unit can be **avoided**.	• If more than one unit transfusions are being requested for routine pre-operative patients, the laboratory staff should be encouraged to **challenge the request before issuing the blood, with the support of the Hospital Transfusion / PBM team**. This also **strengthens team working** rather than clinicians and lab staff working in "silos".<br>• The Hospital Transfusion / PBM team should **work with clinicians to continue to monitor practice in relation to this standard** by conducting further **local audits** of the proportions of **patients receiving single or more than one unit transfusions**, and **feeding back these findings to clinical teams**.

Patient Blood Management in patients who have received a post-operative transfusion

Clinical staff** should attempt **at least one** **(PBM standard 10) or all (PBM standard 11)** **appropriate patient blood management measures** in patients who receive a **transfusion** during **major blood loss surgery

- Clinical staff prescribed post-operative transfusion in **71% (22/31)** of our patients undergoing major blood loss surgery compared to 74% (2878/3874) nationally.
- In our patients undergoing major blood loss surgery who received post-operative blood transfusion, clinical staff attempted at **least one** appropriate PBM measure in **93% (14/15)**, compared to 85% (1714/2026) nationally.
- In our patients undergoing major blood loss surgery who received a post-operative blood transfusion, clinical staff attempted **all** appropriate PBM measures in **0% (0/15)**, compared to 8% (1714/2085) nationally.

How do we compare with other hospitals?

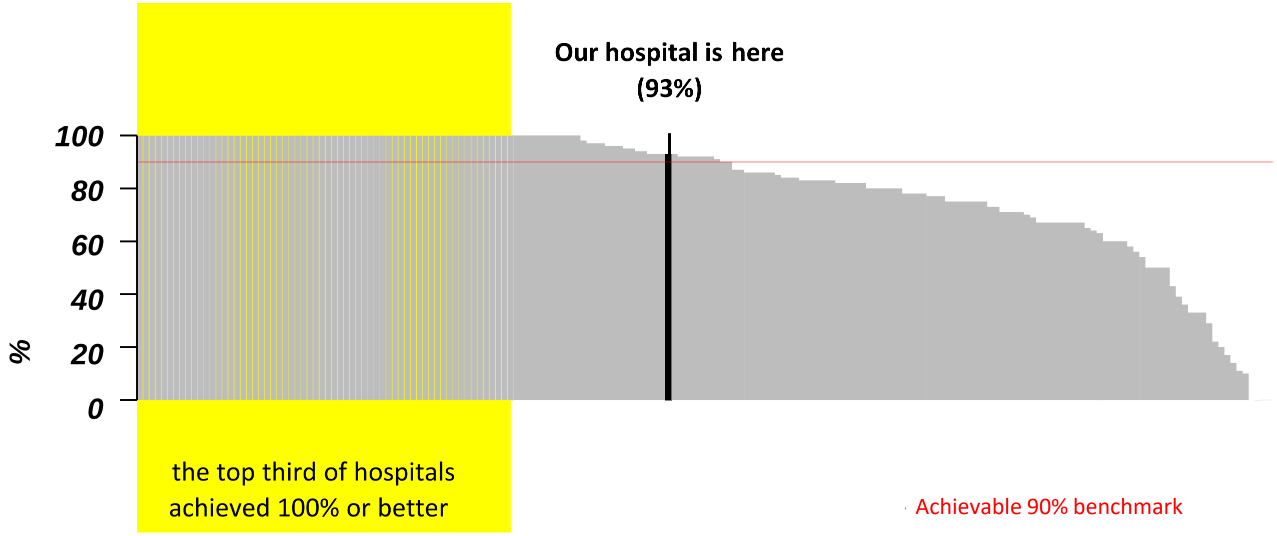

Figure 15: PBM standard 10 hospital comparison

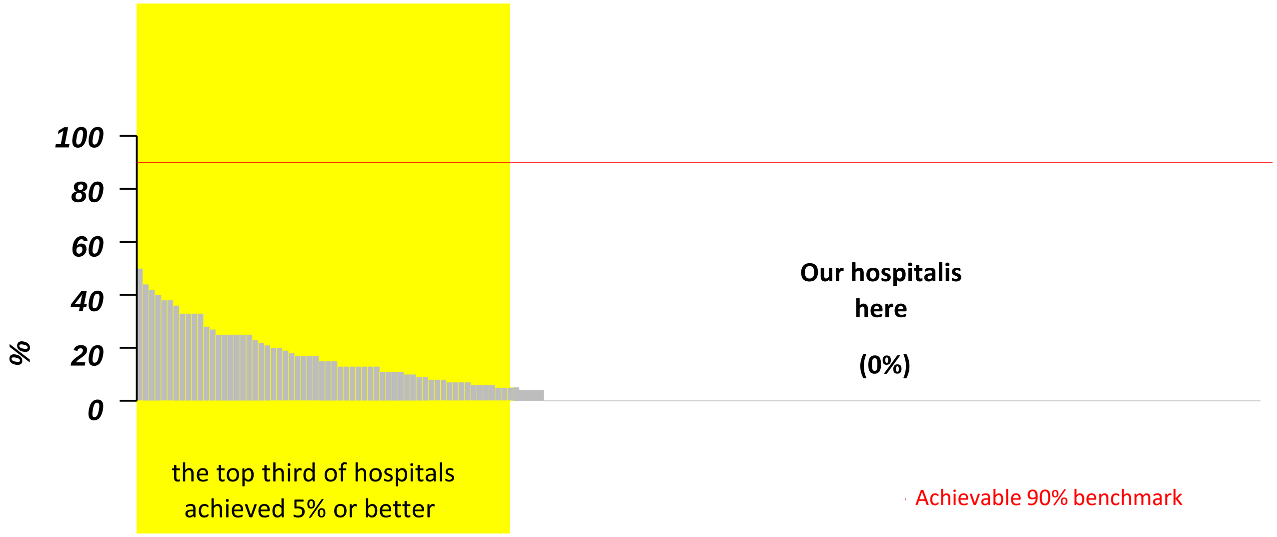

Figure 16: PBM standard 11 hospital comparison

Why is this standard important?

- The over-arching purpose of PBM is to improve outcomes for patients.
- The components of PBM as described in Table 1 combine to minimise the need for blood transfusion.
- PBM can lead to reduced complications, hospital stay and the cost of treating complications.
- PBM Standard 10 relates to at least one PBM intervention being implemented. This is an easy standard to comply with and really corresponds to the basics of good peri-operative practice.
- PBM standard 11 is the most stringent. It reflects the very best practice, where hospitals have processes in place to ensure that every possible PBM intervention has been implemented to the benefit of patients. (Standard 11 is the final common pathway for PBM).
- Hospitals should not be satisfied with high achievement in standard 10, but rather, should strive to increase their achievement for standard 11 which represents best PBM practice.

What should we do next? Recommendations:

For our Hospital	For theatre teams	For the Hospital Transfusion / Patient Blood Management team
**For PBM Standard 10:**<br>• Our performance for this standard was in the mid-range when compared with other hospitals nationally, but there is still room for further improvement.<br>• We should formulate an action plan to continue to improve our practice towards achieving this standard.<br><br>**For PBM Standard 11:**<br>• Along with most other hospitals, our performance for this standard was lower than expected.<br>• We should formulate an action plan to improve performance towards a more feasible short-term goal, such as 50% working towards 100%.	• The theatre team, anaesthetists and surgeons should **ensure that the PBM measures** (presented in Table 1) and as identified by the Hospital Transfusion / Patient Blood Management Committee are **implemented as appropriate**.<br>• Where available, **peer data should be applied to compare individual surgeons** and **encourage participation in PBM**.	• The Committee should **ensure that local guidelines exist regarding the use of PBM measures** (as outlined in Table 1), including **clear recommendations** on the **individuals or teams responsible for implementing these measures**.<br>• The Committee should **ensure that the use of tranexamic acid is the standard of care for surgical patients** expected to have moderate or more significant blood loss unless contraindicated.<br>• The Committee should **identify the need for intra-operative cell salvage and resource appropriately**; this would normally be used in relevant high blood loss procedures in association with tranexamic acid.

Section 4: What are the key priorities?

With the completion of the audit, the writing group discussed a number of key messages. It was recognised that this audit has applied a large number of PBM standards, although a number are interrelated and may represent more 'incremental' standards.

Recognising the need to facilitate the implementation of PBM across many hospitals withdifferent levels of current engagement with PBM, and the desire to maximise impact on reducing patient risk, the audit writing team agreed three top priorities:

1. **Pre-operative anaemia detection and management**: Pathways for (elective) surgery should ensure effective processes exist for the recognition of anaemia, which is followed up and acted upon within a timely period, to allow effective management and investigation.
2. **Post-operative transfusions**: Restrictive use of red cells to only transfuse once the Hb falls below 70g/l should be considered the default indication (unless patients have acute coronary ischaemia).
3. **Broader use of tranexamic acid**: There is accumulating evidence that tranexamic acid is safe and effective to reduce blood loss and minimise transfusion requirements in elective surgery; it is likely to be highly cost-effective.

In the case of all three key priorities, this audit clearly demonstrates considerable opportunities to improve practice, and hence patient outcomes, at many hospitals.

These priorities should be underpinned by local guidelines and educational programmes targeted at those health care professionals responsible for the care of patients in the surgical pathway and reinforced by continuous quality improvement audits overseen by the Hospital Transfusion / Patient Blood Management Committee. The workplan will be supported by the NICE Transfusion guidelines to be published in November 2015 and indevelopment at the time of preparing this report.[16]

Section 5: What should we do next? Action Plan

There is evidence that making a specific action plan can facilitate responding to feedback efficiently

- An action plan outlines **what** needs to be done, by **whom**, **where** and **when**.
- We recommend that you pick **two or three recommendations** from the findings report that are important for your hospital to address
- You may find it useful to **complete some or all of this action planning template** when planning your hospital's response to the feedback from this audit

Recommendation from Audit Report	Key Action(s) to be taken	Co-ordinator for Action	Target of Action	Location for Action	Timescale for Action	Indicator of Outcome for Action
Our **selected recommendations(s)** to address	I.e. **WHAT** needs to be done to **address** this recommendation in our hospital?	I.e. **WHO** will be responsible for this action?	I.e. **WHOM** is the action going to affect?	I.e. **WHERE** will this action take place or be discussed?	I.e. **WHEN** will this action be completed (mm/yyyy)?	I.e. **HOW** will the outcome of the action be monitored to ensure it has achieved the desired effect?

Name of individual(s) completing the action plan: ***Signature:*** ***Date:***

Name of individual(s) completing the action plan: ***Signature:*** ***Date:***

eAppendix 3. Trial Analyses

List of Tables

Trial Results Tables..

Table A1: Surgical Trial - Baseline Patient-Level Characteristics 2

Table A2: Surgical Trial - Follow-Up Patient-Level Characteristics......................... 4

Table A3: Surgical Trial - Patient-Level Outcomes at Baseline 6

Table A4: Surgical Trial - Patient-Level Outcomes at Follow-Up 8

Table A5: Surgical Trial - Primary and Sensitivity Analyses 11

Table A6: Surgical Trial - Patient-Level Supportive Analyses............................... 12

Table A7: Surgical Trial - Cluster-Level SHOT Outcomes at Baseline and Follow-Up 14

Table A8: Surgical Trial: Cluster-Level BSMS Outcomes at Baseline and Follow-Up 16

Table A9: Haematology Trial - Baseline Patient-Level Characteristics 18

Table A10: Haematology Trial - Follow-Up Patient-Level Characteristics............. 21

Table A11: Haematology Trial - Patient-Level Outcomes at Baseline 24

Table A12: Haematology Trial - Patient-Level Outcomes at Follow-Up 26

Table A13: Haematology Trial - Primary and Sensitivity Analyses 28

Table A14: Haematology Trial - Patient-Level Supportive Analysis 29

Table A15: Haematology Trial - Cluster-Level SHOT Outcomes at Baseline and Follow-Up 30

Table A16: Haematology Trial - Cluster-Level BSMS Outcomes at Baseline and Follow-Up 32

© 2022 Stanworth SJ et al. *JAMA Network Open*.

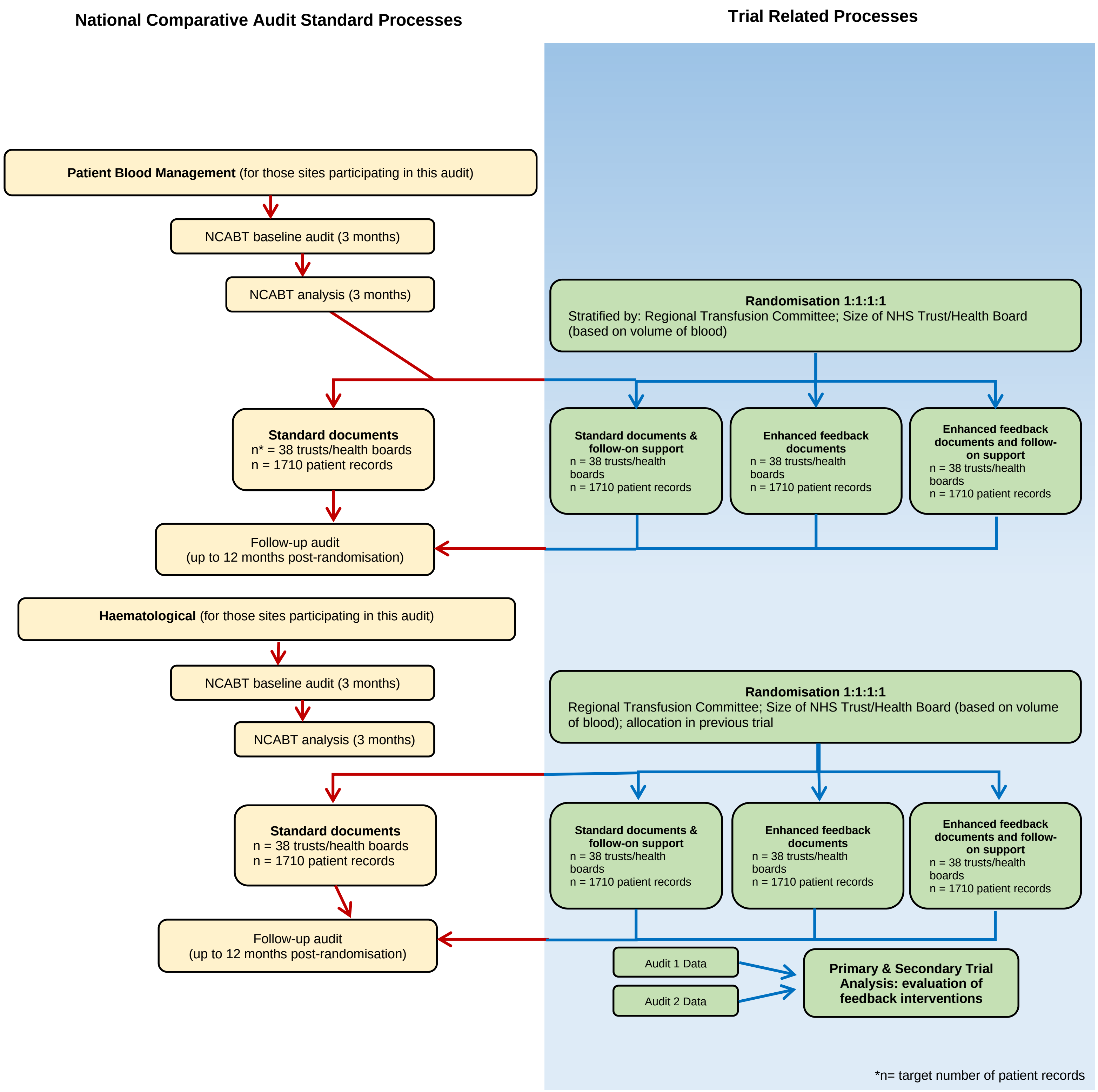

© 2022 Stanworth SJ et al. *JAMA Network Open*.

Table A1: Surgical Trial - Baseline Patient-Level Characteristics

	CONTENT		SUPPORT		
**Variable**	**STANDARD**	**ENHANCED**	**STANDARD**	**ENHANCED**	**Total**
**Entire Sample**	**(n=1322)**	**(n=1392)**	**(n=1306)**	**(n=1408)**	**(n=2714)**
**Age (years),** Mean (SD) N	74.7 (13.80) 1318	75.1 (14.13) 1383	75.3 (13.80) 1302	74.6 (14.12) 1399	74.9 (13.97) 2701
**Gender,** Male N(%)	435 (32.9%)	470 (33.8%)	418 (32.0%)	487 (34.6%)	905 (33.3%)
**Surgical procedure,** N(%)					
Orthopaedic	444 (33.6%)	484 (34.8%)	435 (33.3%)	493 (35.0%)	928 (34.2%)
Cardiac	233 (17.6%)	222 (15.9%)	222 (17.0%)	233 (16.5%)	455 (16.8%)
Fractured neck of femur	421 (31.8%)	418 (30.0%)	410 (31.4%)	429 (30.5%)	839 (30.9%)
Other	222 (16.8%)	258 (18.5%)	234 (17.9%)	246 (17.5%)	480 (17.7%)
Missing	2 (0.2%)	10 (0.7%)	5 (0.4%)	7 (0.5%)	12 (0.4%)
**Attendance at pre-operative clinic,** N(%)					
Yes	839 (63.5%)	922 (66.2%)	841 (64.4%)	920 (65.3%)	1761 (64.9%)
No: Orthopaedic	40 (3.0%)	51 (3.7%)	44 (3.4%)	47 (3.3%)	91 (3.4%)
No: Cardiac	46 (3.5%)	49 (3.5%)	38 (2.9%)	57 (4.0%)	95 (3.5%)
No: Fractured neck of femur	371 (28.1%)	301 (21.6%)	357 (27.3%)	315 (22.4%)	672 (24.8%)
No: Other type of surgery	24 (1.8%)	33 (2.4%)	25 (1.9%)	32 (2.3%)	57 (2.1%)
No: Surgery type missing	0 (0.0%)	1 (0.1%)	0 (0.0%)	1 (0.1%)	1 (0.0%)
Missing	2 (0.2%)	35 (2.5%)	1 (0.1%)	36 (2.6%)	37 (1.4%)
**Hb level at clinic,** Mean (SD) N	122.9 (17.54) 477	121.9 (16.57) 426	122.3 (16.60) 415	122.5 (17.51) 488	122.4 (17.09) 903
**Hb level prior to surgery,** Mean (SD) N	117.4 (17.84) 1289	116.3 (17.18) 1305	117.6 (17.11) 1260	116.1 (17.87) 1334	116.8 (17.52) 2594
**Hb level on Day 1 post surgery,** Mean (SD) N	89.2 (12.59) 1137	90.7 (13.52) 1151	90.6 (12.81) 1105	89.3 (13.30) 1183	90.0 (13.08) 2288
**Prescribed tranexamic acid,** N(%)	454 (34.3%)	440 (31.6%)	425 (32.5%)	469 (33.3%)	894 (32.9%)
**Surgery complications,** N(%)	328 (24.8%)	381 (27.4%)	326 (25.0%)	383 (27.2%)	709 (26.1%)
**Patient died,** N(%)	49 (3.7%)	63 (4.5%)	61 (4.7%)	51 (3.6%)	112 (4.1%)
**Pre-operative transfusion conducted,** N(%)	120 (9.1%)	129 (9.3%)	114 (8.7%)	135 (9.6%)	249 (9.2%)
**Intra-operative transfusion conducted,** N(%)	179 (13.5%)	184 (13.2%)	171 (13.1%)	192 (13.6%)	363 (13.4%)
**Post-operative transfusion conducted,** N(%)	1245 (94.2%)	1315 (94.5%)	1235 (94.6%)	1325 (94.1%)	2560 (94.3%)
**Pre-Operative Blood Transfusions**	**(n=120)**	**(n=129)**	**(n=114)**	**(n=135)**	**(n=249)**
**Hb level,** Mean (SD) N	82.8 (10.62) 115	84.0 (12.75) 123	84.1 (12.42) 110	82.8 (11.18) 128	83.4 (11.76) 238
**Professional making decision to transfuse,** N(%)					
Nurse	1 (0.8%)	0 (0.0%)	1 (0.9%)	0 (0.0%)	1 (0.4%)
Consultant	55 (45.8%)	50 (38.8%)	47 (41.2%)	58 (43.0%)	105 (42.2%)
Other Doctor	51 (42.5%)	64 (49.6%)	50 (43.9%)	65 (48.1%)	115 (46.2%)
Other	7 (5.8%)	1 (0.8%)	5 (4.4%)	3 (2.2%)	8 (3.2%)
Missing	6 (5.0%)	14 (10.9%)	11 (9.6%)	9 (6.7%)	20 (8.0%)
**No. of units transfused,** N(%)					

© 2022 Stanworth SJ et al. *JAMA Network Open*.

	CONTENT		SUPPORT		
**Variable**	**STANDARD**	**ENHANCED**	**STANDARD**	**ENHANCED**	**Total**
Single unit transfusion	26 (21.7%)	26 (20.2%)	19 (16.7%)	33 (24.4%)	52 (20.9%)
Two or more units transfused	93 (77.5%)	101 (78.3%)	94 (82.5%)	100 (74.1%)	194 (77.9%)
Missing	1 (0.8%)	2 (1.6%)	1 (0.9%)	2 (1.5%)	3 (1.2%)
**No. of units transfused,** Mean (SD) N	2.0 (0.74) 119	2.2 (1.04) 127	2.1 (0.80) 113	2.0 (1.00) 133	2.1 (0.91) 246
**Record of acute coronary ischaemia,** N(%)	8 (6.7%)	5 (3.9%)	7 (6.1%)	6 (4.4%)	13 (5.2%)
**Intra-Operative Blood Transfusions**	**(n=179)**	**(n=184)**	**(n=171)**	**(n=192)**	**(n=363)**
**Hb level,** Mean (SD) N	82.5 (16.03) 121	84.5 (14.40) 117	83.0 (14.59) 120	84.0 (15.94) 118	83.5 (15.25) 238
**Professional making decision to transfuse,** N(%)					
Consultant	108 (60.3%)	84 (45.7%)	91 (53.2%)	101 (52.6%)	192 (52.9%)
Other Doctor	55 (30.7%)	57 (31.0%)	59 (34.5%)	53 (27.6%)	112 (30.9%)
Other	2 (1.1%)	12 (6.5%)	7 (4.1%)	7 (3.6%)	14 (3.9%)
Missing	14 (7.8%)	31 (16.8%)	14 (8.2%)	31 (16.2%)	45 (12.4%)
**No. of units transfused,** N(%)					
Single unit transfusion	55 (30.7%)	60 (32.6%)	63 (36.8%)	52 (27.1%)	115 (31.7%)
Two or more units transfused	105 (58.7%)	101 (54.9%)	94 (55.0%)	112 (58.3%)	206 (56.7%)
Missing	19 (10.6%)	23 (12.5%)	14 (8.2%)	28 (14.6%)	42 (11.6%)
**No. of units transfused,** Mean (SD) N	2.1 (1.45) 160	2.0 (1.29) 161	2.0 (1.45) 157	2.1 (1.30) 164	2.1 (1.37) 321
**Post-Operative Blood Transfusions**	**(n=1245)**	**(n=1315)**	**(n=1235)**	**(n=1325)**	**(n=2560)**
**Hb level,** Mean (SD) N	79.7 (9.93) 1192	79.9 (9.24) 1207	80.0 (9.18) 1168	79.6 (9.97) 1231	79.8 (9.59) 2399
**Professional making decision to transfuse,** N(%)					
Nurse	2 (0.2%)	6 (0.5%)	6 (0.5%)	2 (0.2%)	8 (0.3%)
Consultant	288 (23.1%)	376 (28.6%)	289 (23.4%)	375 (28.3%)	664 (25.9%)
Other Doctor	781 (62.7%)	674 (51.3%)	731 (59.2%)	724 (54.6%)	1455 (56.8%)
Other	37 (3.0%)	42 (3.2%)	49 (4.0%)	30 (2.3%)	79 (3.1%)
Missing	137 (11.0%)	217 (16.5%)	160 (12.9%)	194 (14.7%)	354 (13.9%)
**No. of units transfused,** N(%)					
Single unit transfusion	414 (33.3%)	353 (26.8%)	355 (28.7%)	412 (31.1%)	767 (30.0%)
Two or more units transfused	818 (65.7%)	940 (71.5%)	864 (70.0%)	894 (67.5%)	1758 (68.7%)
Missing	13 (1.0%)	22 (1.7%)	16 (1.3%)	19 (1.4%)	35 (1.4%)
**No. of units transfused,** Mean (SD) N	1.8 (0.89) 1232	1.8 (0.69) 1293	1.8 (0.70) 1219	1.8 (0.86) 1306	1.8 (0.79) 2525
**Record of acute coronary ischaemia,** N(%)	95 (7.6%)	59 (4.5%)	79 (6.4%)	75 (5.7%)	154 (6.0%)

© 2022 Stanworth SJ et al. *JAMA Network Open*.

Table A2: Surgical Trial - Follow-Up Patient-Level Characteristics

	CONTENT		SUPPORT		
**Variable**	**STANDARD**	**ENHANCED**	**STANDARD**	**ENHANCED**	**Total**
**Entire Sample**	**(n=1224)**	**(n=998)**	**(n=1118)**	**(n=1104)**	**(n=2222)**
**Age (years),** Mean (SD) N	75.3 (13.26) 1220	73.8 (14.35) 997	74.7 (13.56) 1118	74.6 (14.00) 1099	74.6 (13.78) 2217
**Gender,** Male N(%)	391 (31.9%)	317 (31.8%)	360 (32.2%)	348 (31.5%)	708 (31.9%)
**Surgical procedure,** N(%)					
Orthopaedic	470 (38.4%)	333 (33.4%)	411 (36.8%)	392 (35.5%)	803 (36.1%)
Cardiac	147 (12.0%)	189 (18.9%)	177 (15.8%)	159 (14.4%)	336 (15.1%)
Fractured neck of femur	424 (34.6%)	281 (28.2%)	346 (30.9%)	359 (32.5%)	705 (31.7%)
Other	182 (14.9%)	194 (19.4%)	184 (16.5%)	192 (17.4%)	376 (16.9%)
Missing	1 (0.1%)	1 (0.1%)	0 (0.0%)	2 (0.2%)	2 (0.1%)
**Attendance at pre-operative clinic,** N(%)					
Yes	736 (60.1%)	657 (65.8%)	735 (65.7%)	658 (59.6%)	1393 (62.7%)
No: Orthopaedic	52 (4.2%)	36 (3.6%)	26 (2.3%)	62 (5.6%)	88 (4.0%)
No: Cardiac	15 (1.2%)	36 (3.6%)	25 (2.2%)	26 (2.4%)	51 (2.3%)
No: Fractured neck of femur	385 (31.5%)	246 (24.6%)	306 (27.4%)	325 (29.4%)	631 (28.4%)
No: Other type of surgery	35 (2.9%)	23 (2.3%)	26 (2.3%)	32 (2.9%)	58 (2.6%)
No: Surgery type missing	1 (0.1%)	0 (0.0%)	0 (0.0%)	1 (0.1%)	1 (0.0%)
**Hb level at clinic,** Mean (SD) N	121.8 (15.69) 482	119.4 (16.51) 372	121.1 (15.89) 435	120.4 (16.30) 419	120.8 (16.08) 854
**Hb level prior to surgery,** Mean (SD) N	115.8 (16.86) 1189	115.9 (17.41) 972	116.8 (16.72) 1097	114.9 (17.45) 1064	115.9 (17.10) 2161
**Hb level on Day 1 post surgery,** Mean (SD) N	89.8 (13.78) 1069	91.1 (13.92) 879	90.9 (13.53) 989	89.8 (14.16) 959	90.4 (13.85) 1948
**Prescribed tranexamic acid,** N(%)	464 (37.9%)	426 (42.7%)	491 (43.9%)	399 (36.1%)	890 (40.1%)
**Patient died,** N(%)	32 (2.6%)	39 (3.9%)	35 (3.1%)	36 (3.3%)	71 (3.2%)
**Pre-operative transfusion conducted,** N(%)	120 (9.8%)	127 (12.7%)	104 (9.3%)	143 (13.0%)	247 (11.1%)
**Intra-operative transfusion conducted,** N(%)	188 (15.4%)	185 (18.5%)	186 (16.6%)	187 (16.9%)	373 (16.8%)
**Post-operative transfusion conducted,** N(%)	1166 (95.3%)	938 (94.0%)	1059 (94.7%)	1045 (94.7%)	2104 (94.7%)
**Pre-Operative Blood Transfusions**	**(n=120)**	**(n=127)**	**(n=104)**	**(n=143)**	**(n=247)**
**Hb level,** Mean (SD) N	82.1 (13.52) 93	85.0 (15.21) 85	83.4 (15.05) 77	83.6 (13.94) 101	83.5 (14.39) 178
**No. of units transfused,** N(%)					
Single unit transfusion	24 (20.0%)	22 (17.3%)	17 (16.3%)	29 (20.3%)	46 (18.6%)
Two or more units transfused	61 (50.8%)	62 (48.8%)	60 (57.7%)	63 (44.1%)	123 (49.8%)
Missing	35 (29.2%)	43 (33.9%)	27 (26.0%)	51 (35.7%)	78 (31.6%)
**No. of units transfused,** Mean (SD) N	2.1 (1.16) 85	2.2 (2.12) 84	2.2 (2.19) 77	2.0 (1.15) 92	2.1 (1.71) 169
**Record of acute coronary ischaemia,** N(%)	8 (6.7%)	10 (7.9%)	6 (5.8%)	12 (8.4%)	18 (7.3%)

© 2022 Stanworth SJ et al. *JAMA Network Open*.

	CONTENT		SUPPORT		
**Variable**	**STANDARD**	**ENHANCED**	**STANDARD**	**ENHANCED**	**Total**
**Intra-Operative Blood Transfusions**	**(n=188)**	**(n=185)**	**(n=186)**	**(n=187)**	**(n=373)**
**Hb level,** Mean (SD) N	83.3 (12.60) 112	83.0 (16.47) 105	83.0 (14.43) 112	83.2 (14.78) 105	83.1 (14.57) 217
**No. of units transfused,** N(%)					
Single unit transfusion	72 (38.3%)	73 (39.5%)	71 (38.2%)	74 (39.6%)	145 (38.9%)
Two or more units transfused	116 (61.7%)	110 (59.5%)	114 (61.3%)	112 (59.9%)	226 (60.6%)
Missing	0 (0.0%)	2 (1.1%)	1 (0.5%)	1 (0.5%)	2 (0.5%)
**No. of units transfused,** Mean (SD) N	2.1 (1.35) 188	2.1 (1.61) 183	2.2 (1.71) 185	2.0 (1.21) 186	2.1 (1.48) 371
**Post-Operative Blood Transfusions**	**(n=1166)**	**(n=938)**	**(n=1059)**	**(n=1045)**	**(n=2104)**
**Hb level,** Mean (SD) N	79.5 (9.92) 1036	78.7 (9.57) 823	79.3 (9.78) 946	78.9 (9.77) 913	79.1 (9.77) 1859
**No. of units transfused,** N(%)					
Single unit transfusion	481 (41.3%)	369 (39.3%)	421 (39.8%)	429 (41.1%)	850 (40.4%)
Two or more units transfused	645 (55.3%)	536 (57.1%)	602 (56.8%)	579 (55.4%)	1181 (56.1%)
Missing	40 (3.4%)	33 (3.5%)	36 (3.4%)	37 (3.5%)	73 (3.5%)
**No. of units transfused,** Mean (SD) N	1.6 (0.66) 1126	1.7 (0.95) 905	1.7 (0.91) 1023	1.7 (0.68) 1008	1.7 (0.80) 2031
**Record of acute coronary ischaemia,** N(%)	61 (5.2%)	54 (5.8%)	54 (5.1%)	61 (5.8%)	115 (5.5%)

© 2022 Stanworth SJ et al. *JAMA Network Open*.

Table A3: Surgical Trial - Patient-Level Outcomes at Baseline

	CONTENT		SUPPORT		
**Variable**	**STANDARD**	**ENHANCED**	**STANDARD**	**ENHANCED**	**Total**
**Entire NCA Audit Sample**	**(n=1322)**	**(n=1392)**	**(n=1306)**	**(n=1408)**	**(n=2714)**
**Primary Outcome,** N(%)					
Appropriate	209 (15.8%)	174 (12.5%)	177 (13.6%)	206 (14.6%)	383 (14.1%)
Outside Guidelines	1036 (78.4%)	1097 (78.8%)	1050 (80.4%)	1083 (76.9%)	2133 (78.6%)
Unclassified: ACI status unknown, Hb 70-80g/L	26 (2.0%)	15 (1.1%)	13 (1.0%)	28 (2.0%)	41 (1.5%)
Unclassified: Hb level missing	51 (3.9%)	106 (7.6%)	66 (5.1%)	91 (6.5%)	157 (5.8%)
**Secondary Outcome,** Mean (SD) N					
Total Volume of Blood Transfused	2.1 (1.40) 1287	2.2 (1.16) 1344	2.1 (1.22) 1275	2.2 (1.34) 1356	2.2 (1.28) 2631
**Supportive Outcomes[1],** N(%)					
**PBM Standard 1**					
Meets standard	215 (16.3%)	199 (14.3%)	181 (13.9%)	233 (16.5%)	414 (15.3%)
Does not meet standard	363 (27.5%)	345 (24.8%)	331 (25.3%)	377 (26.8%)	708 (26.1%)
Insufficient information	323 (24.4%)	430 (30.9%)	384 (29.4%)	369 (26.2%)	753 (27.7%)
Excluded	421 (31.8%)	418 (30.0%)	410 (31.4%)	429 (30.5%)	839 (30.9%)
**PBM Standard 2**					
Meets standard	8 (0.6%)	15 (1.1%)	11 (0.8%)	12 (0.9%)	23 (0.8%)
Does not meet standard	99 (7.5%)	96 (6.9%)	86 (6.6%)	109 (7.7%)	195 (7.2%)
Insufficient information	4 (0.3%)	13 (0.9%)	9 (0.7%)	8 (0.6%)	17 (0.6%)
Excluded	1211 (91.6%)	1268 (91.1%)	1200 (91.9%)	1279 (90.8%)	2479 (91.3%)
**PBM Standard 3**					
Meets standard	2 (0.2%)	1 (0.1%)	3 (0.2%)	0 (0.0%)	3 (0.1%)
Does not meet standard	25 (1.9%)	34 (2.4%)	24 (1.8%)	35 (2.5%)	59 (2.2%)
Insufficient information	27 (2.0%)	35 (2.5%)	29 (2.2%)	33 (2.3%)	62 (2.3%)
Excluded	1268 (95.9%)	1322 (95.0%)	1250 (95.7%)	1340 (95.2%)	2590 (95.4%)
**PBM Standard 4**					
Meets standard	30 (2.3%)	34 (2.4%)	21 (1.6%)	43 (3.1%)	64 (2.4%)
Does not meet standard	78 (5.9%)	82 (5.9%)	79 (6.0%)	81 (5.8%)	160 (5.9%)
Insufficient information	3 (0.2%)	8 (0.6%)	6 (0.5%)	5 (0.4%)	11 (0.4%)
Excluded	1211 (91.6%)	1268 (91.1%)	1200 (91.9%)	1279 (90.8%)	2479 (91.3%)
**PBM Standard 8**					
Meets standard	57 (4.3%)	38 (2.7%)	47 (3.6%)	48 (3.4%)	95 (3.5%)
Does not meet standard	827 (62.6%)	906 (65.1%)	888 (68.0%)	845 (60.0%)	1733 (63.9%)
Insufficient information	361 (27.3%)	371 (26.7%)	300 (23.0%)	432 (30.7%)	732 (27.0%)
Excluded	77 (5.8%)	77 (5.5%)	71 (5.4%)	83 (5.9%)	154 (5.7%)
**Intermediate Outcomes,** N(%)					
Planned surgery date equals actual surgery date	642 (48.6%)	651 (46.8%)	629 (48.2%)	664 (47.2%)	1293 (47.6%)
Attendance at pre-operative assessment clinic	839 (63.5%)	922 (66.2%)	841 (64.4%)	920 (65.3%)	1761 (64.9%)

© 2022 Stanworth SJ et al. *JAMA Network Open*.

	CONTENT		SUPPORT		
**Variable**	**STANDARD**	**ENHANCED**	**STANDARD**	**ENHANCED**	**Total**
Ferritin checked	68 (5.1%)	79 (5.7%)	52 (4.0%)	95 (6.7%)	147 (5.4%)
Oral iron before operation	135 (10.2%)	152 (10.9%)	141 (10.8%)	146 (10.4%)	287 (10.6%)
IV iron before operation	12 (0.9%)	8 (0.6%)	10 (0.8%)	10 (0.7%)	20 (0.7%)
Prescribed tranexamic acid	454 (34.3%)	440 (31.6%)	425 (32.5%)	469 (33.3%)	894 (32.9%)
Collection for IOCS commenced	162 (12.3%)	139 (10.0%)	166 (12.7%)	135 (9.6%)	301 (11.1%)
Post-operative cell salvage used	24 (1.8%)	12 (0.9%)	19 (1.5%)	17 (1.2%)	36 (1.3%)
Patient given post-operative iron	195 (14.8%)	251 (18.0%)	239 (18.3%)	207 (14.7%)	446 (16.4%)
Length of post-operative hospital stay**,** Mean (SD) N	12.7 (11.63) 1299	13.1 (11.72) 1359	13.1 (12.48) 1288	12.7 (10.87) 1370	12.9 (11.67) 2658
**Pre-Operative Blood Transfusions**	**(n=120)**	**(n=129)**	**(n=114)**	**(n=135)**	**(n=249)**
**Supportive Outcomes,** N(%)					
**Pre-operative component of primary outcome**					
Appropriate	11 (9.2%)	18 (14.0%)	13 (11.4%)	16 (11.9%)	29 (11.6%)
Outside Guidelines	102 (85.0%)	102 (79.1%)	96 (84.2%)	108 (80.0%)	204 (81.9%)
Unclassified: ACI status unknown, Hb 70-80g/L	2 (1.7%)	3 (2.3%)	1 (0.9%)	4 (3.0%)	5 (2.0%)
Unclassified: Hb level missing	5 (4.2%)	6 (4.7%)	4 (3.5%)	7 (5.2%)	11 (4.4%)
**No. of units transfused pre-Operatively**					
Single unit transfusion	26 (21.7%)	26 (20.2%)	19 (16.7%)	33 (24.4%)	52 (20.9%)
Two or more units transfused	93 (77.5%)	101 (78.3%)	94 (82.5%)	100 (74.1%)	194 (77.9%)
Missing	1 (0.8%)	2 (1.6%)	1 (0.9%)	2 (1.5%)	3 (1.2%)
**Post-Operative Blood Transfusions**	**(n=1245)**	**(n=1315)**	**(n=1235)**	**(n=1325)**	**(n=2560)**
**Supportive Outcomes,** N(%)					
**Post-operative component of primary outcome**					
Appropriate	205 (16.5%)	167 (12.7%)	173 (14.0%)	199 (15.0%)	372 (14.5%)
Outside Guidelines	964 (77.4%)	1027 (78.1%)	985 (79.8%)	1006 (75.9%)	1991 (77.8%)
Unclassified: ACI status unknown, Hb 70-80g/L	25 (2.0%)	16 (1.2%)	13 (1.1%)	28 (2.1%)	41 (1.6%)
Unclassified: Hb level missing	51 (4.1%)	105 (8.0%)	64 (5.2%)	92 (6.9%)	156 (6.1%)
**No. of units transfused post-Operatively**					
Single unit transfusion	414 (33.3%)	353 (26.8%)	355 (28.7%)	412 (31.1%)	767 (30.0%)
Two or more units transfused	818 (65.7%)	940 (71.5%)	864 (70.0%)	894 (67.5%)	1758 (68.7%)
Missing	13 (1.0%)	22 (1.7%)	16 (1.3%)	19 (1.4%)	35 (1.4%)

[1]Audit Standards, Patient Blood Management Standards, 1,2,3,4 and 8

Standard 1: Clinical staff must ensure that patients listed for elective major blood loss surgery have an Hb measured at least 14 days pre-operatively and act upon results

Standard 2: Clinical staff should only prescribe a pre-operative transfusion in patients undergoing elected major blood loss surgery if the Hb is less than the defined Hb threshold for transfusion (70g/L in patients without acute coronary ischaemia or 80g/L in patients with acute coronary ischaemia)

© 2022 Stanworth SJ et al. *JAMA Network Open*.

Standard 3: Clinical staff should only prescribe a pre-operative transfusion in patients undergoing elective major blood loss surgery if the Hb is less than the defined Hb threshold for transfusion and pre-operative anaemia optimisation has been attempted

Standard 4: For patients receiving a pre-operative transfusion, clinical staff should prescribe one unit of red cells at a time and re-check Hb before prescribing a further unit

Standard 8: In patients who do not have active post-operative bleeding, clinical staff should only prescribe a transfusion if the Hb is less than the defined Hb threshold or for transfusion (70g/L in patients without acute coronary ischaemia 80g/L in patients with acute coronary ischaemia).

Table A4: Surgical Trial - Patient-Level Outcomes at Follow-Up

	CONTENT		SUPPORT		
**Variable**	**STANDARD**	**ENHANCED**	**STANDARD**	**ENHANCED**	**Total**
**Entire NCA Audit Sample**	**(n=1224)**	**(n=998)**	**(n=1118)**	**(n=1104)**	**(n=2222)**
**Primary Outcome,** N(%)					
Appropriate	198 (16.2%)	152 (15.2%)	176 (15.7%)	174 (15.8%)	350 (15.8%)
Outside Guidelines	901 (73.6%)	726 (72.7%)	822 (73.5%)	805 (72.9%)	1627 (73.2%)
Unclassified: ACI status unknown, Hb 70-80g/L	1 (0.1%)	2 (0.2%)	3 (0.3%)	0 (0.0%)	3 (0.1%)
Unclassified: Hb level missing	124 (10.1%)	118 (11.8%)	117 (10.5%)	125 (11.3%)	242 (10.9%)
**Secondary Outcome,** Mean (SD) N					
Total Volume of Blood Transfused	2.0 (1.22) 1147	2.2 (1.71) 921	2.1 (1.62) 1052	2.1 (1.28) 1016	2.1 (1.46) 2068
**Supportive Outcomes[1],** N(%)					
**PBM Standard 1**					
Meets standard	240 (19.6%)	191 (19.1%)	218 (19.5%)	213 (19.3%)	431 (19.4%)
Does not meet standard	332 (27.1%)	265 (26.6%)	285 (25.5%)	312 (28.3%)	597 (26.9%)
Insufficient information	228 (18.6%)	261 (26.2%)	269 (24.1%)	220 (19.9%)	489 (22.0%)
Excluded	424 (34.6%)	281 (28.2%)	346 (30.9%)	359 (32.5%)	705 (31.7%)
**PBM Standard 2**					
Meets standard	14 (1.1%)	9 (0.9%)	12 (1.1%)	11 (1.0%)	23 (1.0%)
Does not meet standard	67 (5.5%)	68 (6.8%)	63 (5.6%)	72 (6.5%)	135 (6.1%)
Insufficient information	35 (2.9%)	46 (4.6%)	28 (2.5%)	53 (4.8%)	81 (3.6%)
Excluded	1108 (90.5%)	875 (87.7%)	1015 (90.8%)	968 (87.7%)	1983 (89.2%)
**PBM Standard 3**					
Meets standard	2 (0.2%)	1 (0.1%)	0 (0.0%)	3 (0.3%)	3 (0.1%)
Does not meet standard	24 (2.0%)	23 (2.3%)	26 (2.3%)	21 (1.9%)	47 (2.1%)
Insufficient information	48 (3.9%)	57 (5.7%)	39 (3.5%)	66 (6.0%)	105 (4.7%)
Excluded	1150 (94.0%)	917 (91.9%)	1053 (94.2%)	1014 (91.8%)	2067 (93.0%)
**PBM Standard 4**					
Meets standard	29 (2.4%)	26 (2.6%)	21 (1.9%)	34 (3.1%)	55 (2.5%)
Does not meet standard	51 (4.2%)	48 (4.8%)	52 (4.7%)	47 (4.3%)	99 (4.5%)
Insufficient information	36 (2.9%)	49 (4.9%)	30 (2.7%)	55 (5.0%)	85 (3.8%)
Excluded	1108 (90.5%)	875 (87.7%)	1015 (90.8%)	968 (87.7%)	1983 (89.2%)
**PBM Standard 8**					
Meets standard	68 (5.6%)	32 (3.2%)	48 (4.3%)	52 (4.7%)	100 (4.5%)
Does not meet standard	717 (58.6%)	546 (54.7%)	621 (55.5%)	642 (58.2%)	1263 (56.8%)
Insufficient information	381 (31.1%)	360 (36.1%)	390 (34.9%)	351 (31.8%)	741 (33.3%)

© 2022 Stanworth SJ et al. *JAMA Network Open*.

	CONTENT		SUPPORT		
Variable	STANDARD	ENHANCED	STANDARD	ENHANCED	Total
Excluded	58 (4.7%)	60 (6.0%)	59 (5.3%)	59 (5.3%)	118 (5.3%)
**Intermediate Outcomes,** N(%)					
Planned surgery date equals actual surgery date	813 (66.4%)	547 (54.8%)	743 (66.5%)	617 (55.9%)	1360 (61.2%)
Attendance at pre-operative assessment clinic	736 (60.1%)	657 (65.8%)	735 (65.7%)	658 (59.6%)	1393 (62.7%)
Ferritin checked	71 (5.8%)	76 (7.6%)	68 (6.1%)	79 (7.2%)	147 (6.6%)
Oral iron before operation	144 (11.8%)	106 (10.6%)	131 (11.7%)	119 (10.8%)	250 (11.3%)
IV iron before operation	15 (1.2%)	12 (1.2%)	9 (0.8%)	18 (1.6%)	27 (1.2%)
Prescribed tranexamic acid	464 (37.9%)	426 (42.7%)	491 (43.9%)	399 (36.1%)	890 (40.1%)
Collection for IOCS commenced	127 (10.4%)	125 (12.5%)	132 (11.8%)	120 (10.9%)	252 (11.3%)
Post-operative cell salvage used	15 (1.2%)	19 (1.9%)	20 (1.8%)	14 (1.3%)	34 (1.5%)
Patient given post-operative iron	185 (15.1%)	169 (16.9%)	193 (17.3%)	161 (14.6%)	354 (15.9%)
Length of post-operative hospital stay, Mean (SD) N	12.5 (10.41) 1195	12.8 (12.15) 979	12.2 (10.88) 1106	13.1 (11.56) 1068	12.7 (11.22) 2174
**Pre-Operative Blood Transfusions**	**(n=120)**	**(n=127)**	**(n=104)**	**(n=143)**	**(n=247)**
**Supportive Outcomes,** N(%)					
**Pre-operative component of primary outcome**					
Appropriate	16 (13.3%)	10 (7.9%)	12 (11.5%)	14 (9.8%)	26 (10.5%)
Outside Guidelines	77 (64.2%)	75 (59.1%)	65 (62.5%)	87 (60.8%)	152 (61.5%)
Unclassified: Hb level missing	27 (22.5%)	42 (33.1%)	27 (26.0%)	42 (29.4%)	69 (27.9%)
**No. of units transfused pre-operatively**					
Single unit transfusion	24 (20.0%)	22 (17.3%)	17 (16.3%)	29 (20.3%)	46 (18.6%)
Two or more units transfused	61 (50.8%)	62 (48.8%)	60 (57.7%)	63 (44.1%)	123 (49.8%)
Missing	35 (29.2%)	43 (33.9%)	27 (26.0%)	51 (35.7%)	78 (31.6%)
**Post-Operative Blood Transfusions**	**(n=1166)**	**(n=938)**	**(n=1059)**	**(n=1045)**	**(n=2104)**
**Supportive Outcomes,** N(%)					
**Post-operative component of primary outcome**					
Appropriate	198 (17.0%)	155 (16.5%)	174 (16.4%)	179 (17.1%)	353 (16.8%)
Outside Guidelines	844 (72.4%)	671 (71.5%)	774 (73.1%)	741 (70.9%)	1515 (72.0%)
Unclassified: ACI status unknown, Hb 70-80g/L	1 (0.1%)	2 (0.2%)	3 (0.3%)	0 (0.0%)	3 (0.1%)
Unclassified: Hb level missing	123 (10.5%)	110 (11.7%)	108 (10.2%)	125 (12.0%)	233 (11.1%)
**No. of units transfused post-operatively**					
Single unit transfusion	481 (41.3%)	369 (39.3%)	421 (39.8%)	429 (41.1%)	850 (40.4%)
Two or more units transfused	645 (55.3%)	536 (57.1%)	602 (56.8%)	579 (55.4%)	1181 (56.1%)
Missing	40 (3.4%)	33 (3.5%)	36 (3.4%)	37 (3.5%)	73 (3.5%)

[1]Audit Standards, Patient Blood Management Standards, 1,2,3,4 and 8

Standard 1: Clinical staff must ensure that patients listed for elective major blood loss surgery have an Hb measured at least 14 days pre-operatively and act upon results

© 2022 Stanworth SJ et al. *JAMA Network Open*.

Standard 2: Clinical staff should only prescribe a pre-operative transfusion in patients undergoing elected major blood loss surgery if the Hb is less than the defined Hb threshold for transfusion (70g/L in patients without acute coronary ischaemia or 80g/L in patients with acute coronary ischaemia)

Standard 3: Clinical staff should only prescribe a pre-operative transfusion in patients undergoing elective major blood loss surgery if the Hb is less than the defined Hb threshold for transfusion and pre-operative anaemia optimisation has been attempted

Standard 4: For patients receiving a pre-operative transfusion, clinical staff should prescribe one unit of red cells at a time and re-check Hb before prescribing a further unit

Standard 8: In patients who do not have active post-operative bleeding, clinical staff should only prescribe a transfusion if the Hb is less than the defined Hb threshold or for transfusion (70g/L in patients without acute coronary ischaemia 80g/L in patients with acute coronary ischaemia).

© 2022 Stanworth SJ et al. *JAMA Network Open*.

Table A5: Surgical Trial - Primary and Sensitivity Analyses

Analysis	Unadjusted Proportion Appropriate STANDARD	Unadjusted Proportion Appropriate ENHANCED	Estimated Adjusted Risk Difference (95% CI)	Estimated Adjusted Odds Ratio (97.5% CI)	Estimated Adjusted Odds Ratio (95% CI)	p-value	N
**Primary Analysis (multiple imputation, 100 imputations, full imputation model)**							
Content	0.184	0.176	-0.01 (-0.07 to 0.04)	0.91 (0.61 to 1.36)	0.91 (0.64 to 1.30)	0.605	2222
Support	0.181	0.180	0.01 (-0.05 to 0.06)	1.05 (0.68 to 1.61)	1.05 (0.72 to 1.52)	0.807	2222
Interaction	0.184	0.167	0.05 (-0.08 to 0.13)	1.15 (0.52 to 2.56)	1.15 (0.57 to 2.31)	0.696	2222
**Sensitivity Analyses**							
**Complete Case Analysis**							
Content	0.180	0.173	-0.01 (-0.06 to 0.04)	0.93 (0.62 to 1.40)	0.93 (0.65 to 1.33)	0.694	1977
Support	0.176	0.178	0.00 (-0.05 to 0.06)	1.02 (0.68 to 1.53)	1.02 (0.71 to 1.45)	0.934	1977
Interaction	0.181	0.163	0.00 (-0.10 to 0.10)	0.98 (0.45 to 2.13)	0.98 (0.50 to 1.93)	0.947	1977

© 2022 Stanworth SJ et al. *JAMA Network Open*.

Table A6: Surgical Trial - Patient-Level Supportive Analyses

Analysis	Unadjusted Proportion Appropriate STANDARD	Unadjusted Proportion Appropriate ENHANCED	Estimated Adjusted Odds Ratio (95% CI)	p-value	N
**Pre-Operative Component of the Primary Outcome***					
Content	0.154	0.117	0.68 (0.28 to 1.67)	0.398	247
Support	0.145	0.128	0.85 (0.36 to 2.00)	0.712	247
Interaction	0.139	0.122	1.92 (0.35 to 10.67)	0.456	247
**Post-Operative Component of the Primary Outcome**					
Content	0.195	0.191	0.94 (0.66 t0 1.34)	0.734	2104
Support	0.190	0.196	1.09 (0.74 to 1.59)	0.673	2104
Interaction	0.195	0.185	1.12 (0.55 to 2.27)	0.764	2104
**NCA Audit Standard 1*** [1]					
Content	0.390	0.364	0.96 (0.69 to 1.35)	0.828	1517
Support	0.382	0.374	0.96 (0.68 to 1.34)	0.793	1517
Interaction	0.379	0.375	1.18 (0.61 to 2.29)	0.625	1517
**NCA Audit Standard 2***					
Content	0.129	0.138	1.01 (0.33 to 3.14)	0.983	239
Support	0.179	0.098	0.54 (0.22 to 1.31)	0.174	239
Interaction	0.146	0.097	1.03 (0.17 to 6.18)	0.972	239
**NCA Audit Standard 4***					
Content	0.515	0.580	1.86 (0.89 to 3.86)	0.098	239
Support	0.476	0.604	1.66 (0.82 to 3.37)	0.159	239
Interaction	0.531	0.600	0.42 (0.10 to 1.71)	0.226	239
**NCA Audit Standard 8***					
Content	0.114	0.078	0.55 (0.23 to 1.30)	0.170	2104
Support	0.099	0.097	0.86 (0.35 to 2.11)	0.746	2104
Interaction	0.108	0.060	0.51 (0.11 to 2.41)	0.396	2104

NOTE: multiple imputation was used (100 imputations, full imputation model) for all analyses. * Reduced analysis model to facilitate model convergence, design factors omitted as cluster-level covariates

* Reduced model to facilitate model convergence, design factors omitted as cluster-level covariates in the analysis;

[1]Audit Standards, Patient Blood Management Standards, 1,2,3,4 and 8

Standard 1: Clinical staff must ensure that patients listed for elective major blood loss surgery have an Hb measured at least 14 days pre-operatively and act upon results

Standard 2: Clinical staff should only prescribe a pre-operative transfusion in patients undergoing elected major blood loss surgery if the Hb is less than the defined Hb threshold for transfusion (70g/L in patients without acute coronary ischaemia or 80g/L in patients with acute coronary ischaemia)

Standard 3: Clinical staff should only prescribe a pre-operative transfusion in patients undergoing elective major blood loss surgery if the Hb is less than the defined Hb threshold for transfusion and pre-operative anaemia optimisation has been attempted

© 2022 Stanworth SJ et al. *JAMA Network Open*.

Standard 4: For patients receiving a pre-operative transfusion, clinical staff should prescribe one unit of red cells at a time and re-check Hb before prescribing a further unit

Standard 8: In patients who do not have active post-operative bleeding, clinical staff should only prescribe a transfusion if the Hb is less than the defined Hb threshold or for transfusion (70g/L in patients without acute coronary ischaemia 80g/L in patients with acute coronary ischaemia).

© 2022 Stanworth SJ et al. *JAMA Network Open*.

Table A7: Surgical Trial - Cluster-Level SHOT Outcomes at Baseline and Follow-Up

	CONTENT		SUPPORT		
**Variable**	**STANDARD**	**ENHANCED**	**STANDARD**	**ENHANCED**	**Total**
**Entire SHOT Sample (N)**	**(n=66)**	**(n=69)**	**(n=67)**	**(n=68)**	**(n=135)**
**Total Number of Incidents**, Median (IQR) (Range)					
Baseline	14.5 (5, 22) (0, 154)	13 (9, 24) (3, 77)	13 (8, 22) (0, 77)	16 (7, 24) (0, 154)	14 (8, 23) (0, 154)
Follow-Up	13 (6, 21) (1, 158)	13 (7, 22) (0, 68)	12 (5, 21) (0, 68)	13 (7.5, 23) (1, 158)	13 (6, 22) (0, 158)
Oct-Dec 2014 (Baseline)	3 (1, 6) (0, 27)	3 (1, 6) (0, 21)	3 (1, 6) (0, 21)	3 (1, 6) (0, 27)	3 (1, 6) (0, 27)
Jan-Mar 2015 (Baseline)	4 (1, 7) (0, 40)	4 (2, 8) (0, 28)	3 (2, 7) (0, 28)	4 (1, 7.5) (0, 40)	4 (1, 7) (0, 40)
Apr-Jun 2015 (Baseline)	3 (1, 6) (0, 40)	4 (2, 5) (0, 21)	3 (2, 5) (0, 26)	4 (2, 7) (0, 40)	3 (2, 6) (0, 40)
Jul-Sept 2015 (Baseline)	4 (1, 7) (0, 47)	4 (2, 6) (0, 24)	3 (1, 5) (0, 24)	4 (1.5, 7) (0, 47)	4 (1, 7) (0, 47)
Nov-Jan 2016 (Follow-Up)	3 (1, 6) (0, 49)	3 (1, 5) (0, 18)	3 (1, 6) (0, 18)	3 (1, 5) (0, 49)	3 (1, 6) (0, 49)
Feb-Apr 2016 (Follow-Up)	3 (1, 6) (0, 37)	3 (1, 5) (0, 15)	3 (1, 5) (0, 15)	3 (2, 6.5) (0, 37)	3 (1, 6) (0, 37)
May-Jul 2016 (Follow-Up)	3 (1, 6) (0, 30)	3 (2, 6) (0, 24)	3 (1, 6) (0, 24)	3.5 (1, 6) (0, 30)	3 (1, 6) (0, 30)
Aug-Oct 2016 (Follow-Up)	3 (1, 6) (0, 42)	3 (1, 7) (0, 25)	3 (1, 6) (0, 19)	3 (1.5, 7) (0, 42)	3 (1, 7) (0, 42)
**Number of Relevant Errors**, Median (IQR) (Range)					
Baseline	0 (0, 1) (0, 6)	0 (0, 1) (0, 5)	0 (0, 1) (0, 4)	0 (0, 1) (0, 6)	0 (0, 1) (0, 6)
Follow-Up	0 (0, 1) (0, 9)	0 (0, 1) (0, 6)	0 (0, 1) (0, 6)	0 (0, 1) (0, 9)	0 (0, 1) (0, 9)
Oct-Dec 2014 (Baseline)	0 (0, 0) (0, 1)	0 (0, 0) (0, 3)	0 (0, 0) (0, 3)	0 (0, 0) (0, 2)	0 (0, 0) (0, 3)
Jan-Mar 2015 (Baseline)	0 (0, 0) (0, 2)	0 (0, 0) (0, 3)	0 (0, 0) (0, 2)	0 (0, 0) (0, 3)	0 (0, 0) (0, 3)
Apr-Jun 2015 (Baseline)	0 (0, 0) (0, 3)	0 (0, 0) (0, 2)	0 (0, 0) (0, 2)	0 (0, 0) (0, 3)	0 (0, 0) (0, 3)
Jul-Sept 2015 (Baseline)	0 (0, 0) (0, 2)	0 (0, 0) (0, 2)	0 (0, 0) (0, 2)	0 (0, 0) (0, 1)	0 (0, 0) (0, 2)
Nov-Jan 2016 (Follow-Up)	0 (0, 0) (0, 2)	0 (0, 0) (0, 2)	0 (0, 0) (0, 2)	0 (0, 0) (0, 2)	0 (0, 0) (0, 2)
Feb-Apr 2016 (Follow-Up)	0 (0, 0) (0, 3)	0 (0, 0) (0, 2)	0 (0, 0) (0, 2)	0 (0, 0) (0, 3)	0 (0, 0) (0, 3)
May-Jul 2016 (Follow-Up)	0 (0, 0) (0, 2)	0 (0, 0) (0, 2)	0 (0, 0) (0, 2)	0 (0, 0) (0, 2)	0 (0, 0) (0, 2)
Aug-Oct 2016 (Follow-Up)	0 (0, 0) (0, 2)	0 (0, 0) (0, 2)	0 (0, 0) (0, 2)	0 (0, 0) (0, 2)	0 (0, 0) (0, 2)
**Number of Relevant Near Miss or Right-Blood Right-Patient Incidents**, Median (IQR) (Range)					
Baseline	0 (0, 1) (0, 2)	0 (0, 0) (0, 2)	0 (0, 1) (0, 2)	0 (0, 0) (0, 1)	0 (0, 0) (0, 2)
Follow-Up	0 (0, 1) (0, 9)	0 (0, 0) (0, 4)	0 (0, 1) (0, 6)	0 (0, 0) (0, 9)	0 (0, 0) (0, 9)
Oct-Dec 2014 (Baseline)	0 (0, 0) (0, 1)	0 (0, 0) (0, 1)	0 (0, 0) (0, 1)	0 (0, 0) (0, 1)	0 (0, 0) (0, 1)
Jan-Mar 2015 (Baseline)	0 (0, 0) (0, 1)	0 (0, 0) (0, 1)	0 (0, 0) (0, 1)	0 (0, 0) (0, 1)	0 (0, 0) (0, 1)
Apr-Jun 2015 (Baseline)	0 (0, 0) (0, 1)	0 (0, 0) (0, 1)	0 (0, 0) (0, 1)	0 (0, 0) (0, 1)	0 (0, 0) (0, 1)
Jul-Sept 2015 (Baseline)	0 (0, 0) (0, 1)	0 (0, 0) (0, 2)	0 (0, 0) (0, 2)	0 (0, 0) (0, 1)	0 (0, 0) (0, 2)
Nov-Jan 2016 (Follow-Up)	0 (0, 0) (0, 3)	0 (0, 0) (0, 2)	0 (0, 0) (0, 2)	0 (0, 0) (0, 3)	0 (0, 0) (0, 3)
Feb-Apr 2016 (Follow-Up)	0 (0, 0) (0, 3)	0 (0, 0) (0, 1)	0 (0, 0) (0, 3)	0 (0, 0) (0, 2)	0 (0, 0) (0, 3)
May-Jul 2016 (Follow-Up)	0 (0, 0) (0, 1)	0 (0, 0) (0, 1)	0 (0, 0) (0, 1)	0 (0, 0) (0, 1)	0 (0, 0) (0, 1)
Aug-Oct 2016 (Follow-Up)	0 (0, 0) (0, 3)	0 (0, 0) (0, 1)	0 (0, 0) (0, 2)	0 (0, 0) (0, 3)	0 (0, 0) (0, 3)
**Total Number of Unpredictable Incidents**, Median (IQR) (Range)					
Baseline	1 (0, 3) (0, 10)	1 (0, 2) (0, 15)	1 (0, 3) (0, 15)	1 (0, 2) (0, 14)	1 (0, 3) (0, 15)
Follow-Up	1 (0, 2) (0, 8)	1 (0, 2) (0, 8)	1 (0, 2) (0, 7)	1 (0, 2) (0, 8)	1 (0, 2) (0, 8)
Oct-Dec 2014 (Baseline)	0 (0, 1) (0, 5)	0 (0, 0) (0, 4)	0 (0, 1) (0, 5)	0 (0, 1) (0, 4)	0 (0, 1) (0, 5)
Jan-Mar 2015 (Baseline)	0 (0, 1) (0, 4)	0 (0, 1) (0, 5)	0 (0, 1) (0, 5)	0 (0, 1) (0, 4)	0 (0, 1) (0, 5)

© 2022 Stanworth SJ et al. *JAMA Network Open*.

	CONTENT		SUPPORT		
**Variable**	**STANDARD**	**ENHANCED**	**STANDARD**	**ENHANCED**	**Total**
Apr-Jun 2015 (Baseline)	0 (0, 1) (0, 4)	0 (0, 1) (0, 5)	0 (0, 1) (0, 5)	0 (0, 1) (0, 5)	0 (0, 1) (0, 5)
Jul-Sept 2015 (Baseline)	0 (0, 0) (0, 5)	0 (0, 1) (0, 5)	0 (0, 1) (0, 4)	0 (0, 1) (0, 5)	0 (0, 1) (0, 5)
Nov-Jan 2016 (Follow-Up)	0 (0, 1) (0, 3)	0 (0, 1) (0, 3)	0 (0, 1) (0, 3)	0 (0, 1) (0, 2)	0 (0, 1) (0, 3)
Feb-Apr 2016 (Follow-Up)	0 (0, 0) (0, 3)	0 (0, 0) (0, 5)	0 (0, 1) (0, 2)	0 (0, 0) (0, 5)	0 (0, 0) (0, 5)
May-Jul 2016 (Follow-Up)	0 (0, 0) (0, 4)	0 (0, 1) (0, 4)	0 (0, 0) (0, 3)	0 (0, 1) (0, 4)	0 (0, 0) (0, 4)
Aug-Oct 2016 (Follow-Up)	0 (0, 1) (0, 3)	0 (0, 0) (0, 4)	0 (0, 1) (0, 3)	0 (0, 1) (0, 4)	0 (0, 1) (0, 4)
**Total Number of Possibly Preventable Incidents**, Median (IQR) (Range)					
Baseline	0 (0, 2) (0, 72)	1 (0, 2) (0, 9)	1 (0, 2) (0, 9)	1 (0, 2) (0, 72)	1 (0, 2) (0, 72)
Follow-Up	0 (0, 1) (0, 17)	0 (0, 1) (0, 7)	0 (0, 1) (0, 7)	0 (0, 1) (0, 17)	0 (0, 1) (0, 17)
Oct-Dec 2014 (Baseline)	0 (0, 0) (0, 11)	0 (0, 1) (0, 3)	0 (0, 1) (0, 3)	0 (0, 0) (0, 11)	0 (0, 1) (0, 11)
Jan-Mar 2015 (Baseline)	0 (0, 0) (0, 22)	0 (0, 0) (0, 4)	0 (0, 0) (0, 5)	0 (0, 1) (0, 22)	0 (0, 0) (0, 22)
Apr-Jun 2015 (Baseline)	0 (0, 1) (0, 21)	0 (0, 1) (0, 4)	0 (0, 1) (0, 4)	0 (0, 1) (0, 21)	0 (0, 1) (0, 21)
Jul-Sept 2015 (Baseline)	0 (0, 0) (0, 18)	0 (0, 0) (0, 5)	0 (0, 0) (0, 4)	0 (0, 0) (0, 18)	0 (0, 0) (0, 18)
Nov-Jan 2016 (Follow-Up)	0 (0, 0) (0, 12)	0 (0, 0) (0, 3)	0 (0, 0) (0, 6)	0 (0, 0) (0, 12)	0 (0, 0) (0, 12)
Feb-Apr 2016 (Follow-Up)	0 (0, 0) (0, 1)	0 (0, 0) (0, 2)	0 (0, 0) (0, 2)	0 (0, 0) (0, 1)	0 (0, 0) (0, 2)
May-Jul 2016 (Follow-Up)	0 (0, 0) (0, 4)	0 (0, 0) (0, 3)	0 (0, 0) (0, 3)	0 (0, 0) (0, 4)	0 (0, 0) (0, 4)
Aug-Oct 2016 (Follow-Up)	0 (0, 0) (0, 2)	0 (0, 0) (0, 4)	0 (0, 0) (0, 4)	0 (0, 0) (0, 2)	0 (0, 0) (0, 4)

© 2022 Stanworth SJ et al. *JAMA Network Open*.

Table A8: Surgical Trial: Cluster-Level BSMS Outcomes at Baseline and Follow-Up

	CONTENT		SUPPORT		
**Variable**	**STANDARD**	**ENHANCED**	**STANDARD**	**ENHANCED**	**Total**
**Entire BSMS Sample**	**(n=66)**	**(n=69)**	**(n=67)**	**(n=68)**	**(n=135)**
**Total Volume of RBC Transfused**, Median (IQR) (Range) N					
Baseline	8048.5 (5595, 10746) (138, 31311) 62	8172 (5175, 11863) (548, 39006) 66	7346.5 (5160.5, 11373.5) (548, 31311) 64	8194 (5917.5, 11228.5) (138, 39006) 64	8147 (5372, 11245.5) (138, 39006) 128
Follow-Up	6736.5 (4920, 10316) (565, 30230) 62	7506 (5072, 12756) (649, 32670) 67	6670 (4920, 10534) (2240, 29528) 63	7438 (5085, 11059) (565, 32670) 66	7012 (4970, 10927) (565, 32670) 129
Oct-Dec 2014 (Baseline)	2040 (1427, 2802) (235, 7749) 61	2037 (1340, 3059) (574, 9132) 65	1909 (1222, 2996) (594, 8008) 63	2082 (1476, 2853) (235, 9132) 63	2039 (1340, 2946) (235, 9132) 126
Jan-Mar 2015 (Baseline)	1936 (1421, 2798) (168, 7909) 61	1895 (1322, 3158) (509, 9284) 65	1798 (1240, 3002) (648, 7909) 63	1936 (1466, 2798) (168, 9284) 63	1922 (1341, 2905) (168, 9284) 126
Apr-Jun 2015 (Baseline)	2009 (1342, 2762) (195, 7998) 61	1989.5 (1298, 2924) (413, 9986) 66	1756.5 (1247.5, 2801) (488, 7998) 64	2109 (1427, 2858) (195, 9986) 63	2009 (1298, 2813) (195, 9986) 127
Jul-Sept 2015 (Baseline)	1993 (1359, 2576) (138, 7655) 61	1917 (1371, 2878) (433, 10604) 65	1921 (1359, 2771) (712, 7655) 62	1955 (1378.5, 2867.5) (138, 10604) 64	1943 (1367, 2793) (138, 10604) 126
Nov-Jan 2016 (Follow-Up)	1828.5 (1278.5, 2578) (154, 7815) 60	2083 (1252, 3004) (400, 9488) 66	1798.5 (1243, 2813) (400, 7815) 62	2027 (1330, 2751) (154, 9488) 64	1910 (1252, 2755) (154, 9488) 126
Feb-Apr 2016 (Follow-Up)	1710.5 (1246.5, 2506.5) (129, 7458) 60	1999.5 (1231, 3080) (644, 9990) 66	1772 (1208, 2610) (724, 7458) 63	1896 (1281, 2779) (129, 9990) 63	1811 (1231, 2710) (129, 9990) 126
May-Jul 2016 (Follow-Up)	1663 (1155, 2471) (158, 8124) 61	2077 (1299, 3535) (400, 7684) 65	1713 (1235, 2838) (400, 7684) 62	2051.5 (1269, 3015) (158, 8124) 64	1836.5 (1246, 2993) (158, 8124) 126
Aug-Oct 2016 (Follow-Up)	1644.5 (1090, 2716) (100, 7792) 62	1820 (1176, 3200) (571, 6819) 66	1648 (1120, 2725) (415, 7066) 63	1887 (1233, 2975) (100, 7792) 65	1718 (1147.5, 2897.5) (100, 7792) 128
**Total Gross Volume of RBC Issued**, Median (IQR) (Range) N					
Baseline	7991 (5101, 11185) (659, 30843) 63	8302 (5296, 13375.5) (3126, 41681) 68	7809 (5070, 11460) (3102, 31085) 65	8221.5 (5439, 12100) (659, 41681) 66	8113 (5215, 11525) (659, 41681) 131
Follow-Up	8678 (5640, 11365) (828, 31827) 63	8743.5 (5456, 13959.5) (3296, 41827) 68	8678 (5455, 11828) (3407, 32605) 65	8709 (6048, 11711) (828, 41827) 66	8687 (5551, 11828) (828, 41827) 131
Oct-Dec 2014 (Baseline)	2209 (1459, 2865) (248, 7876) 63	2144 (1366.5, 3513) (786, 10176) 68	2209 (1380, 2955) (806, 8222) 65	2159 (1493, 3156) (248, 10176) 66	2209 (1400, 2980) (248, 10176) 131
Jan-Mar 2015 (Baseline)	2139 (1399, 2775) (190, 8022) 63	2132.5 (1402, 3346) (748, 9798) 68	2131 (1365, 3012) (920, 8509) 65	2153.5(1491, 2887) (190, 9798) 66	2134 (1399, 2987) (190, 9798) 131
Apr-Jun 2015 (Baseline)	2062 (1343, 2884) (213, 8118) 63	2112.5 (1374, 3274) (907, 10840) 68	2019 (1332, 2946) (792, 8118) 65	2189 (1411, 2939) (213, 10840) 66	2081 (1355, 2946) (213, 10840) 131
Jul-Sept 2015 (Baseline)	2134 (1400, 2869) (177, 7811) 63	2205.5 (1408.5, 3532.5) (738, 11013) 68	2126 (1399, 2875) (738, 8126) 65	2182 (1465, 2987) (177, 11013) 66	2138 (1401, 2965) (177, 11013) 131
Nov-Jan 2016 (Follow-Up)	2018 (1346, 2775) (188, 7954) 63	2122.5 (1327, 3256) (778, 9868) 68	2012 (1276, 2921) (756, 7954) 65	2140 (1432, 2888) (188, 9868) 66	2097 (1327, 2921) (188, 9868) 131
Feb-Apr 2016 (Follow-Up)	1902 (1368, 2698) (154, 7589) 63	2107 (1337.5, 3302) (741, 10277) 68	1876 (1272, 2745) (753, 7819) 65	2043.5 (1370, 3050) (154, 10277) 66	2043 (1357, 2971) (154, 10277) 131

© 2022 Stanworth SJ et al. *JAMA Network Open*.

	CONTENT		SUPPORT		
**Variable**	**STANDARD**	**ENHANCED**	**STANDARD**	**ENHANCED**	**Total**
May-Jul 2016 (Follow-Up)	2018 (1223, 2723) (173, 8286) 63	2130 (1298.5, 3460.5) (765, 10506) 68	1996 (1278, 2821) (749, 7956) 65	2118 (1426, 3028) (173, 10506) 66	2030 (1288, 3008) (173, 10506) 131
Aug-Oct 2016 (Follow-Up)	2008 (1192, 2699) (144, 7948) 63	2014 (1266, 3360.5) (780, 11030) 68	1828 (1194, 2747) (723, 7583) 65	2021.5 (1350, 2986) (144, 11030) 66	2008 (1240, 2852) (144, 11030) 131
**Total Volume of RBC Wasted**, Median (IQR) (Range) N					
Baseline	162.5 (107, 265) (2, 1171) 62	165 (98, 294) (22, 1978) 66	161 (99.5, 256) (21, 1565) 64	169.5 (105, 292) (2, 1978) 64	163 (100.5, 279) (2, 1978) 128
Follow-Up	157 (94, 280) (3, 1212) 62	182 (88, 357) (23, 1326) 67	155 (88, 282) (34, 1282) 63	206 (94, 337) (3, 1326) 66	162 (91, 325) (3, 1326) 129
Oct-Dec 2014 (Baseline)	41 (26, 71) (3, 303) 61	39 (21, 78) (4, 655) 65	41 (21, 71) (3, 415) 63	39 (26, 78) (4, 655) 63	40 (23, 76) (3, 655) 126
Jan-Mar 2015 (Baseline)	36 (25, 61) (2, 266) 61	43 (23, 80) (5, 514) 65	37 (25, 66) (2, 442) 63	43 (22, 79) (7, 514) 63	37 (23, 67) (2, 514) 126
Apr-Jun 2015 (Baseline)	40 (25, 64) (3, 269) 61	42 (25, 80) (4, 421) 66	38.5 (22, 71) (3, 406) 64	42 (25, 81) (4, 421) 63	42 (25, 73) (3, 421) 127
Jul-Sept 2015 (Baseline)	44 (28, 70) (2, 333) 61	40 (28, 75) (2, 409) 65	38 (27, 64) (7, 333) 62	44 (28, 79) (2, 409) 64	43 (28, 75) (2, 409) 126
Nov-Jan 2016 (Follow-Up)	36 (22.5, 74.5) (1, 236) 60	47 (24, 76) (2, 380) 66	35 (22, 68) (2, 308) 62	46.5 (23.5, 80.5) (1, 380) 64	42 (23, 75) (1, 380) 126
Feb-Apr 2016 (Follow-Up)	45 (27, 75.5) (0, 254) 60	46.5 (25, 99) (6, 354) 66	39 (26, 78) (6, 254) 63	53 (25, 99) (0, 354) 63	46 (26, 81) (0, 354) 126
May-Jul 2016 (Follow-Up)	41 (21, 71) (1, 544) 61	47 (23, 88) (9, 389) 65	38.5 (23, 79) (9, 389) 62	49.5 (23, 79.5) (1, 544) 64	43 (23, 79) (1, 544) 126
Aug-Oct 2016 (Follow-Up)	37 (20, 75) (1, 371) 62	47.5 (20, 92) (4, 351) 66	36 (20, 75) (4, 365) 63	48 (20, 82) (1, 371) 65	42.5 (20, 82) (1, 371) 128

© 2022 Stanworth SJ et al. *JAMA Network Open*.

Table A9: Haematology Trial - Baseline Patient-Level Characteristics

	CONTENT		SUPPORT		
**Variable**	**STANDARD**	**ENHANCED**	**STANDARD**	**ENHANCED**	**Total**
**Entire Sample**	**(n=2228)**	**(n=2211)**	**(n=2188)**	**(n=2251)**	**(n=4439)**
**Age (years),** Median (IQR) N	73.0 (64.0, 80.0) 2227	72.0 (64.0, 80.0) 2208	72.0 (64.0, 80.0) 2187	72.0 (64.0, 80.0) 2248	72.0 (64.0, 80.0) 4435
**Gender,** Male N(%)	1306 (58.6%)	1335 (60.4%)	1301 (59.5%)	1340 (59.5%)	2641 (59.5%)
**Weight (kg)** Median (IQR) N	71.0 (61.0, 82.0) 1594	72.0 (62.5, 83.9) 1565	72.0 (62.0, 83.0) 1507	71.0 (62.0, 82.8) 1652	71.5 (62.0, 83.0) 3159
**Haematological Diagnosis,** N(%)					
Acute Leukaemia	469 (21.1%)	460 (20.8%)	435 (19.9%)	494 (21.9%)	929 (20.9%)
Chronic leukaemia/ lymphoma and myeloma	751 (33.7%)	752 (34.0%)	754 (34.5%)	749 (33.3%)	1503 (33.9%)
MDS and aplastic anaemia	1038 (46.6%)	975 (44.1%)	1006 (46.0%)	1007 (44.7%)	2013 (45.3%)
**Additional treatment for haematological diagnosis,** N(%)	723 (32.5%)	615 (27.8%)	617 (28.2%)	721 (32.0%)	1338 (30.1%)
Stem cell transplant	128 (5.7%)	126 (5.7%)	94 (4.3%)	160 (7.1%)	254 (5.7%)
Intensive chemotherapy	574 (79.4%)	461 (75.0%)	494 (80.1%)	541 (75.0%)	1035 (77.4%)
Participating in clinical study	191 (8.6%)	166 (7.5%)	195 (8.9%)	162 (7.2%)	357 (8.0%)
**Receive a red blood cell transfusion,** N(%)	2104 (94.4%)	2064 (93.4%)	2060 (94.1%)	2108 (93.6%)	4168 (93.9%)
**Receive a platelet transfusion,** N(%)	868 (39.0%)	790 (35.7%)	811 (37.1%)	847 (37.6%)	1658 (37.4%)
**Transfusion Type,** N(%)					
Red cell and platelet	744 (33.4%)	643 (29.1%)	683 (31.2%)	704 (31.3%)	1387 (31.2%)
Red cell only	1360 (61.0%)	1421 (64.3%)	1377 (62.9%)	1404 (62.4%)	2781 (62.6%)
Platelet only	124 (5.6%)	147 (6.6%)	128 (5.9%)	143 (6.4%)	271 (6.1%)
**Red Cell Transfusions**	**(n=2104)**	**(n=2064)**	**(n=2060)**	**(n=2108)**	**(n=4168)**
**Inpatient,** N(%)	697 (33.1%)	656 (31.8%)	698 (33.9%)	655 (31.1%)	1353 (32.5%)
**Symptoms:**					
**Symptomatic anaemia,** N(%)	1079 (51.3%)	962 (46.6%)	1064 (51.7%)	977 (46.3%)	2041 (49.0%)
**Severity grade,** N(%)					
Mild	434 (40.2%)	395 (41.1%)	409 (38.4%)	420 (43.0%)	829 (40.6%)
Moderate	515 (47.7%)	443 (46.0%)	527 (49.5%)	431 (44.1%)	958 (46.9%)
Severe	87 (8.1%)	90 (9.4%)	99 (9.3%)	78 (8.0%)	177 (8.7%)
Unspecified	43 (4.0%)	34 (3.5%)	29 (2.8%)	48 (4.9%)	77 (3.8%)
**Hb level less than local threshold,** N(%)	581 (27.6%)	563 (27.3%)	557 (27.0%)	587 (27.8%)	1144 (27.4%)
**Chronic transfusion programme,** N(%)	532 (25.3%)	516 (25.0%)	539 (26.2%)	509 (24.1%)	1048 (25.1%)
**Cannot determine,** N(%)	41 (1.9%)	74 (3.6%)	42 (2.0%)	73 (3.5%)	115 (2.8%)
**Clinical indication**, N(%)					
Acute blood loss	38 (1.8%)	47 (2.3%)	43 (2.1%)	42 (2.0%)	85 (2.0%)
Medical anaemia	860 (38.6%)	706 (31.9%)	731 (33.4%)	835 (37.1%)	1566 (35.3%)

© 2022 Stanworth SJ et al. *JAMA Network Open*.

	CONTENT		SUPPORT		
**Variable**	**STANDARD**	**ENHANCED**	**STANDARD**	**ENHANCED**	**Total**
Medical anaemia in patients with cardiovascular disease	63 (3.0%)	58 (2.8%)	62 (3.0%)	59 (2.8%)	121 (2.9%)
Medical anaemia with Sepsis/CNS complications	112 (5.3%)	103 (5.0%)	118 (5.7%)	97 (4.6%)	215 (5.2%)
Medical anaemia when receiving radiotherapy	8 (0.4%)	6 (0.3%)	7 (0.3%)	7 (0.3%)	14 (0.3%)
Chronic anaemia	1376 (65.4%)	1354 (65.6%)	1414 (68.6%)	1316 (62.4%)	2730 (65.5%)
Other	20 (1.0%)	34 (1.6%)	26 (1.3%)	28 (1.3%)	54 (1.3%)
**Number of units transfused,** Mean (SD) N	1.9 (0.56) 2098	2.0 (0.59) 2055	1.9 (0.58) 2051	2.0 (0.58) 2102	2.0 (0.58) 4153
**Pre-transfusion Hb count performed at an appropriate time?** N(%)	1961 (93.2%)	1936 (93.8%)	1905 (92.5%)	1992 (94.5%)	3897 (93.5%)
**Pre-transfusion Hb count,** Mean (SD) N	78.9 (10.50) 1958	79.1 (11.26) 1930	79.3 (10.84) 1904	78.7 (10.92) 1984	79.0 (10.88) 3888
**Hb measured after each unit transfused,** N(%)	63 (3.6%)	56 (3.3%)	70 (4.2%)	49 (2.8%)	119 (3.5%)
**Post-transfusion Hb count performed at an appropriate time?** N(%)	674 (32.0%)	648 (31.4%)	679 (33.0%)	643 (30.5%)	1322 (31.7%)
**Additional units transfused,** Median (IQR) N	1.0 (0.0, 3.0) 2060	1.0 (0.0, 3.0) 2040	1.0 (0.0, 3.0) 2017	1.0 (0.0, 2.0) 2083	1.0 (0.0, 3.0) 4100
**Platelet Transfusions**	**(n=868)**	**(n=790)**	**(n=811)**	**(n=847)**	**(n=1658)**
**Inpatient,** N(%)	497 (57.3%)	472 (59.7%)	475 (58.6%)	494 (58.3%)	969 (58.4%)
**Reason for platelet transfusion,** N(%)					
a. prophylactic to prevent bleeding and not having a procedure modified who bleeding grade 0 or 1	677 (78.0%)	611 (77.3%)	629 (77.6%)	659 (77.8%)	1288 (77.7%)
b. pre procedure modified who bleeding grade 0 or 1 as defined above	74 (8.5%)	72 (9.1%)	75 (9.2%)	71 (8.4%)	146 (8.8%)
**Clinical indication,** N(%)					
Prophylactic	426 (49.1%)	422 (53.4%)	398 (49.1%)	450 (53.1%)	848 (51.1%)
Prophylactic in the presence of currently existing risk factors for bleeding	394 (45.4%)	315 (39.9%)	381 (47.0%)	328 (38.7%)	709 (42.8%)
Pre-procedure	79 (9.1%)	75 (9.5%)	79 (9.7%)	75 (8.9%)	154 (9.3%)
Therapeutic	89 (10.3%)	65 (8.2%)	73 (9.0%)	81 (9.6%)	154 (9.3%)
**Number of units transfused,** Mean (SD) N	1.1 (0.39) 857	1.1 (0.58) 782	1.1 (0.41) 800	1.1 (0.56) 839	1.1 (0.49) 1639
**Platelets HLA matched,** N(%)	66 (7.6%)	51 (6.5%)	75 (9.2%)	42 (5.0%)	117 (7.1%)
**Pre-transfusion platelet count performed at an appropriate time?** N(%)	819 (94.4%)	737 (93.3%)	762 (94.0%)	794 (93.7%)	1556 (93.8%)

© 2022 Stanworth SJ et al. *JAMA Network Open*.

	CONTENT		SUPPORT		
**Variable**	**STANDARD**	**ENHANCED**	**STANDARD**	**ENHANCED**	**Total**
**Pre-transfusion platelet count,** Median (IQR) N	11.0 (8.0, 18.0) 804	11.0 (8.0, 18.0) 698	12.0 (8.0, 18.0) 745	11.0 (8.0, 18.0) 757	11.0 (8.0, 18.0) 1502
**Platelet count above threshold stated in local guidelines,** N(%)	161 (19.7%)	187 (25.4%)	184 (24.1%)	164 (20.7%)	348 (22.4%)
**Platelet count measured after each unit transfused,** N(%)	29 (30.9%)	17 (19.8%)	28 (28.3%)	18 (22.2%)	46 (25.6%)
**Post-transfusion platelet count performed at an appropriate time?** N(%)	531 (61.2%)	462 (58.5%)	488 (60.2%)	505 (59.6%)	993 (59.9%)
**Additional units transfused,** Median (IQR) N	2.0 (0.0, 5.0) 854	2.0 (0.0, 5.0) 779	3.0 (0.0, 6.0) 798	2.0 (0.0, 5.0) 835	2.0 (0.0, 5.0) 1633

Mild *(Chronic fatigue, loss of energy);*
Moderate *(Palpitations; Shortness of breath on exertion etc.);*
Severe *(Shortness of breath at rest; symptoms of ischaemic, heart disease, such as chest pain; hypotension or tachycardia, unresponsive to fluid resuscitation; cardiac failure);* **Unspecified.**
Clinical Indication is based on the National Blood Transfusion Indication Codes (June 2016). **Reason for transfusion** described why the patient needed a transfusion at this particular time, rather than asking what the underlying clinical condition was.
Platelet transfusion: a & b) The WHO grade 0 or 1 defines who should be included in a), so it is not both. See modified WHO grades below:
Modified World Health Organisation bleeding grade 0 or 1
Type of bleeding included:

- No evidence of bleeding
- Mild/moderate petechiae, purpura

Mild/moderate oropharyngeal bleeding, epistaxis <30 minutes in duration

© 2022 Stanworth SJ et al. *JAMA Network Open*.

Table A10: Haematology Trial - Follow-Up Patient-Level Characteristics

	CONTENT		SUPPORT		
**Variable**	**STANDARD**	**ENHANCED**	**STANDARD**	**ENHANCED**	**Total**
**Entire Sample**	**(n=1926)**	**(n=1933)**	**(n=1779)**	**(n=2080)**	**(n=3859)**
**Age (years),** Median (IQR) N	73.0 (64.0, 81.0) 1926	73. (63.0, 81.0) 1933	73.0 (63.0, 81.0) 1779	73.0 (63.0, 81.0) 2080	73.0 (63.0, 81.0) 3859
**Gender,** Male N(%)	1130 (58.7%)	1166 (60.3%)	719 (40.4%)	844 (40.6%)	2296 (59.5%)
**Weight (kg)** Median (IQR) N	72.0 (63.1, 82.5) 1447	72.4 (63.0, 83.0) 1267	72.2 (62.7, 83.0) 1222	72.0 (63.4, 82.0) 1492	72.0 (63.0, 83.0) 2714
**Haematological Diagnosis,** N(%)					
Acute Leukaemia	430 (22.3%)	381 (19.7%)	348 (19.6%)	463 (22.3%)	811 (21.0%)
Chronic leukaemia/ lymphomas and myeloma	625 (32.5%)	603 (31.2%)	591 (33.2%)	637 (30.6%)	1228 (31.8%)
MDS and aplastic anaemia	874 (45.4%)	894 (46.2%)	827 (46.5%)	941 (45.2%)	1768 (45.8%)
**Additional treatment for haematological diagnosis,** N(%)	584 (30.3%)	539 (27.9%)	508 (28.6%)	615 (29.6%)	1123 (29.1%)
Stem cell transplant	93 (4.8%)	94 (4.9%)	93 (5.2%)	94 (4.5%)	187 (4.8%)
Intensive chemotherapy	449 (76.9%)	407 (75.5%)	379 (74.6%)	477 (77.6%)	856 (76.2%)
Participating in clinical study	170 (8.8%)	150 (7.8%)	181 (10.2%)	139 (6.7%)	320 (8.3%)
**Receive a red blood cell transfusion,** N(%)	1815 (94.2%)	1832 (94.8%)	1674 (94.1%)	1973 (94.9%)	3647 (94.5%)
**Receive a platelet transfusion,** N(%)	717 (37.2%)	702 (36.3%)	621 (34.9%)	798 (38.4%)	1419 (36.8%)
**Transfusion Type,** N(%)					
Red cell and platelet	606 (31.5%)	601 (31.1%)	516 (29.0%)	691 (33.2%)	1207 (31.3%)
Red cell only	1209 (62.8%)	1231 (63.7%)	1158 (65.1%)	1282 (61.6%)	2440 (63.2%)
Platelet only	111 (5.8%)	101 (5.2%)	105 (5.9%)	107 (5.1%)	212 (5.5%)
**Red Cell Transfusions**	**(n=1815)**	**(n=1832)**	**(n=1674)**	**(n=1973)**	**(n=3647)**
**Inpatient,** N(%)	564 (31.1%)	547 (29.9%)	528 (31.5%)	583 (29.5%)	1111 (30.5%)
**Symptoms:**					
**Symptomatic anaemia,** N(%)	821 (45.2%)	830 (45.3%)	789 (47.1%)	862 (43.7%)	1651 (45.3%)
**Severity grade,** N(%)					
Mild	332 (40.4%)	346 (41.7%)	337 (42.7%)	341 (39.6%)	678 (41.1%)
Moderate	375 (45.7%)	359 (43.3%)	341 (43.2%)	393 (45.6%)	734 (44.5%)
Severe	73 (8.9%)	68 (8.2%)	67 (8.5%)	74 (8.6%)	141 (8.5%)
Unspecified	41 (5.0%)	57 (6.8%)	44 (5.6%)	54 (6.2%)	98 (5.9%)
**Hb level less than local threshold,** N(%)	505 (27.8%)	487 (26.6%)	467 (27.9%)	525 (26.6%)	992 (27.2%)
**Chronic transfusion programme,** N(%)	607 (33.4%)	578 (31.6%)	535 (32.0%)	650 (32.9%)	1185 (32.5%)
**Cannot determine,** N(%)	54 (3.0%)	80 (4.4%)	52 (3.1%)	82 (4.2%)	134 (3.7%)

© 2022 Stanworth SJ et al. *JAMA Network Open*.

	CONTENT		SUPPORT		
**Variable**	**STANDARD**	**ENHANCED**	**STANDARD**	**ENHANCED**	**Total**
**Clinical indication,** N(%)					
Acute blood loss	29 (1.6%)	40 (2.2%)	30 (1.8%)	39 (2.0%)	69 (1.9%)
Medical anaemia	776 (40.3%)	733 (37.9%)	662 (37.2%)	847 (40.7%)	1509 (39.1%)
Medical anaemia in patients with cardiovascular disease	76 (4.2%)	59 (3.2%)	69 (4.1%)	66 (3.3%)	135 (3.7%)
Medical anaemia with Sepsis/CNS complications	100 (5.5%)	103 (5.6%)	109 (6.5%)	94 (4.8%)	203 (5.6%)
Medical anaemia when receiving radiotherapy	24 (1.3%)	5 (0.3%)	5 (0.3%)	24 (1.2%)	29 (0.8%)
Chronic anaemia	1172 (64.6%)	1126 (61.5%)	1083 (64.7%)	1215 (61.6%)	2298 (63.0%)
Other	51 (2.8%)	66 (3.6%)	74 (4.4%)	43 (2.2%)	117 (3.2%)
**Number of units transfused,** Mean (SD) N	1.8 (0.65) 1813	1.8 (0.60) 1829	1.8 (0.62) 1671	1.8 (0.63) 1971	1.8 (0.62) 3642
**Pre-transfusion Hb count performed at an appropriate time?** N(%)	1713 (94.4%)	1706 (93.1%)	1571 (93.8%)	1848 (93.7%)	3419 (93.7%)
**Pre-transfusion Hb count,** Mean (SD) N	77.2 (9.88) 1713	78.0 (10.11) 1705	77.3 (9.84) 1571	77.8 (10.14) 1847	77.6 (10.01) 3418
**Hb measured after each unit transfused,** N(%)	63 (4.9%)	39 (3.1%)	50 (4.3%)	52 (3.7%)	102 (4.0%)
**Post-transfusion Hb count performed at an appropriate time?** N(%)	600 (33.1%)	568 (31.0%)	557 (33.3%)	611 (31.0%)	1168 (32.0%)
**Additional units transfused,** Median (IQR) N	1.0 (0.0, 3.0) 1772	1.0 (0.0, 3.0) 1770	1.0 (0.0, 2.0) 1635	1.0 (0.0, 3.0) 1907	1.0 (0.0, 3.0) 3542
**Platelet Transfusions**	**(n=717)**	**(n=702)**	**(n=621)**	**(n=798)**	**(n=1419)**
**Inpatient,** N(%)	399 (55.6%)	378 (53.8%)	352 (56.7%)	425 (53.3%)	777 (54.8%)
**Reason for platelet transfusion:**					
a. prophylactic to prevent bleeding and not having a procedure modified who bleeding grade 0 or 1, N(%)	562 (78.4%)	557 (79.3%)	506 (81.5%)	613 (76.8%)	1119 (78.9%)
b. pre procedure modified who bleeding grade 0 or 1 as defined above, N(%)	68 (9.5%)	60 (8.5%)	49 (7.9%)	79 (9.9%)	128 (9.0%)
**Clinical indication,** N(%)					
Prophylactic	329 (45.9%)	328 (46.7%)	282 (45.4%)	375 (47.0%)	657 (46.3%)
Prophylactic in the presence of currently existing risk factors for bleeding	289 (40.3%)	295 (42.0%)	270 (43.5%)	314 (39.3%)	584 (41.2%)
Pre-procedure	70 (9.8%)	65 (9.3%)	53 (8.5%)	82 (10.3%)	135 (9.5%)
Therapeutic	59 (8.2%)	55 (7.8%)	50 (8.1%)	64 (8.0%)	114 (8.0%)
**Number of units transfused,** Mean (SD) N	1.1 (0.37) 704	1.1 (0.60) 690	1.1 (0.37) 614	1.1 (0.58) 780	1.1 (0.50) 1394

© 2022 Stanworth SJ et al. *JAMA Network Open*.

	CONTENT		SUPPORT		
**Variable**	**STANDARD**	**ENHANCED**	**STANDARD**	**ENHANCED**	**Total**
**Platelets HLA matched,** N(%)	37 (5.2%)	61 (8.7%)	50 (8.1%)	48 (6.0%)	98 (6.9%)
**Pre-transfusion platelet count performed at an appropriate time?** N(%)	692 (96.5%)	675 (96.2%)	601 (96.8%)	766 (96.0%)	1367 (96.3%)
**Pre-transfusion platelet count,** Median (IQR) N	11.0 (7.0, 17.0) 688	11.0 (8.0, 18.0) 667	11.0 (7.0, 18.0) 597	11.0 (7.0, 18.0) 758	11.0 (7.0, 18.0) 1355
**Platelet count above threshold stated in local guidelines,** N(%)	154 (22.3%)	173 (25.6%)	166 (27.6%)	161 (21.0%)	327 (23.9%)
**Platelet count measured after each unit transfused,** N(%)	30 (45.5%)	11 (21.6%)	20 (39.2%)	21 (31.8%)	41 (35.0%)
**Post-transfusion platelet count performed at an appropriate time?** N(%)	448 (62.5%)	414 (59.0%)	392 (63.1%)	470 (58.9%)	862 (60.7%)
**Additional units transfused,** Median (IQR) N	2.0 (1.0, 5.0) 700	2.0 (1.0, 5.0) 673	2.0 (1.0, 5.0) 607	2.0 (1.0, 5.0) 766	2.0 (1.0, 5.0) 1373

© 2022 Stanworth SJ et al. *JAMA Network Open*.

Table A11: Haematology Trial - Patient-Level Outcomes at Baseline

	CONTENT		SUPPORT		
**Variable**	**STANDARD**	**ENHANCED**	**STANDARD**	**ENHANCED**	**Total**
**Entire NCA Audit Sample**	**(n=2228)**	**(n=2211)**	**(n=2188)**	**(n=2251)**	**(n=4439)**
**Primary Outcome,** N(%)					
Appropriate	1355 (60.8%)	1396 (63.1%)	1393 (63.7%)	1358 (60.3%)	2751 (62.0%)
Outside Guidelines	617 (27.7%)	524 (23.7%)	544 (24.9%)	597 (26.5%)	1141 (25.7%)
Unclassified	256 (11.5%)	291 (13.2%)	251 (11.5%)	296 (13.1%)	547 (12.3%)
**Supportive Outcomes,**[1] N(%)					
**Standard 1**					
Meets standard	1961 (93.4%)	1936 (94.0%)	1905 (92.7%)	1992 (94.6%)	3897 (93.7%)
Does not meet standard	139 (6.6%)	124 (6.0%)	149 (7.3%)	114 (5.4%)	263 (6.3%)
**Standard 2**					
Meets standard	148 (25.8%)	140 (25.3%)	142 (24.9%)	146 (26.2%)	288 (25.6%)
Does not meet standard	415 (72.4%)	405 (73.1%)	422 (74.0%)	398 (71.5%)	820 (72.8%)
Insufficient information	10 (1.7%)	9 (1.6%)	6 (1.1%)	13 (2.3%)	19 (1.7%)
**Standard 3**					
Meets standard	9 (40.9%)	11 (68.8%)	13 (54.2%)	7 (50.0%)	20 (52.6%)
Does not meet standard	13 (59.1%)	5 (31.3%)	11 (45.8%)	7 (50.0%)	18 (47.4%)
**Standard 6**					
Meets standard	120 (53.6%)	99 (49.3%)	102 (52.8%)	117 (50.4%)	219 (51.5%)
Does not meet standard	95 (42.4%)	82 (40.8%)	82 (42.5%)	95 (40.9%)	177 (41.6%)
Insufficient information	9 (4.0%)	20 (10.0%)	9 (4.7%)	20 (8.6%)	29 (6.8%)
**Standard 7**					
Meets standard	405 (59.8%)	391 (64.0%)	373 (59.3%)	423 (64.2%)	796 (61.8%)
Does not meet standard	272 (40.2%)	220 (36.0%)	256 (40.7%)	236 (35.8%)	492 (38.2%)
**Red Cell Transfusions**	**(n=2104)**	**(n=2064)**	**(n=2060)**	**(n=2108)**	**(n=4168)**
**Secondary Outcome,** Median (IQR) N					
Volume of Red Cells Transfused	2.0 (2.0, 2.0) 2098	2.0 (2.0, 2.0) 2055	2.0 (2.0, 2.0) 2051	2.0 (2.0, 2.0) 2102	2.0 (2.0, 2.0) 4153
**Supportive Outcomes,** N(%)					
**Red cell component of primary outcome**					
Appropriate	1627 (77.3%)	1605 (77.8%)	1651 (80.1%)	1581 (75.0%)	3232 (77.5%)
Outside Guidelines	262 (12.5%)	213 (10.3%)	198 (9.6%)	277 (13.1%)	475 (11.4%)
Unclassified	215 (10.2%)	246 (11.9%)	211 (10.2%)	250 (11.9%)	461 (11.1%)
**Intermediate Outcomes,** N(%)					
**Hb measured after each unit transfused**	63 (3.6%)	56 (3.3%)	70 (4.2%)	49 (2.8%)	119 (3.5%)
**Platelet Transfusions**	**(n=883)**	**(n=808)**	**(n=826)**	**(n=865)**	**(n=1691)**
**Secondary Outcome,** Median (IQR) N					

© 2022 Stanworth SJ et al. *JAMA Network Open*.

	CONTENT		SUPPORT		
**Variable**	**STANDARD**	**ENHANCED**	**STANDARD**	**ENHANCED**	**Total**
Volume of Platelets Transfused	1.0 (1.0, 1.0) 872	1.0 (1.0, 1.0) 800	1.0 (1.0, 1.0) 815	1.0 (1.0, 1.0) 857	1.0 (1.0, 1.0) 1672
**Supportive Outcomes,** N(%)					
**Platelet component of primary outcome**					
Appropriate	278 (31.5%)	256 (31.7%)	257 (31.1%)	277 (32.0%)	534 (31.6%)
Outside Guidelines	406 (46.0%)	346 (42.8%)	384 (46.5%)	368 (42.5%)	752 (44.5%)
Unclassified	199 (22.5%)	206 (25.5%)	185 (22.4%)	220 (25.4%)	405 (24.0%)
**Intermediate Outcomes,** N(%)					
**Platelet count measured after each unit transfused**	29 (30.2%)	18 (20.7%)	29 (28.4%)	18 (22.2%)	47 (25.7%)

[1]Audit Standards, Haematology Standards 1, 2, 3,6 and 7

Standard 1: Clinical staff should measure Hb prior to transfusion of red blood cells in haematology patients

Standard 2: Clinical staff should only transfuse red blood cells in normovolaemic asymptomatic haematology inpatients without additional risk factors (cardiovascular disease or signs or symptoms of cardiovascular compromise, severe sepsis or acute cerebral ischaemia) if their pre-transfusion Hb is less than 70g/L

Standard 3: Clinical staff should only transfuse red blood cells in haematology inpatients with cardiovascular disease or signs or symptoms of cardiovascular compromise if their pre-transfusion Hb is less than 80g/L

Standard 6: Clinical staff should only transfuse prophylactic platelets in patients with a reversible cause for bone marrow failure and no other risk factors for bleeding if their pre-transfusion platelet count is below 10 x 10^9/L

Standard 7: Clinical staff should avoid routinely prescribing prophylactic platelet transfusions to patients with irreversible chronic bone marrow failure

© 2022 Stanworth SJ et al. *JAMA Network Open*.

Table A12: Haematology Trial - Patient-Level Outcomes at Follow-Up

	CONTENT		SUPPORT		
**Variable**	**STANDARD**	**ENHANCED**	**STANDARD**	**ENHANCED**	**Total**
**Entire NCA Audit Sample**	**(n=1926)**	**(n=1933)**	**(n=1779)**	**(n=2080)**	**(n=3859)**
**Primary Outcome,** N(%)					
Appropriate	1308 (67.9%)	1226 (63.4%)	1196 (67.2%)	1338 (64.3%)	2534 (65.7%)
Outside Guidelines	457 (23.7%)	507 (26.2%)	433 (24.3%)	531 (25.5%)	964 (25.0%)
Unclassified	161 (8.4%)	200 (10.3%)	150 (8.4%)	211 (10.1%)	361 (9.4%)
**Supportive Outcomes,** N(%)					
**Standard 1**					
Meets standard	1713 (94.5%)	1706 (93.2%)	1571 (94.0%)	1848 (93.7%)	3419 (93.9%)
Does not meet standard	100 (5.5%)	124 (6.8%)	100 (6.0%)	124 (6.3%)	224 (6.1%)
**Standard 2**					
Meets standard	138 (30.0%)	102 (23.4%)	118 (28.5%)	122 (25.3%)	240 (26.8%)
Does not meet standard	318 (69.1%)	325 (74.5%)	291 (70.3%)	352 (73.0%)	643 (71.8%)
Insufficient information	4 (0.9%)	9 (2.1%)	5 (1.2%)	8 (1.7%)	13 (1.5%)
**Standard 3**					
Meets standard	8 (50.0%)	15 (83.3%)	8 (53.3%)	15 (78.9%)	23 (67.6%)
Does not meet standard	6 (37.5%)	1 (5.6%)	5 (33.3%)	2 (10.5%)	7 (20.6%)
Insufficient information	2 (12.5%)	2 (11.1%)	2 (13.3%)	2 (10.5%)	4 (11.8%)
**Standard 6**					
Meets standard	107 (59.1%)	105 (53.8%)	85 (57.0%)	127 (55.9%)	212 (56.4%)
Does not meet standard	72 (39.8%)	87 (44.6%)	62 (41.6%)	97 (42.7%)	159 (42.3%)
Insufficient information	2 (1.1%)	3 (1.5%)	2 (1.3%)	3 (1.3%)	5 (1.3%)
**Standard 7**					
Meets standard	333 (59.3%)	381 (68.4%)	311 (61.5%)	403 (65.7%)	714 (63.8%)
Does not meet standard	229 (40.7%)	176 (31.6%)	195 (38.5%)	210 (34.3%)	405 (36.2%)
**Red Cell Transfusions**	**(n=1815)**	**(n=1832)**	**(n=1674)**	**(n=1973)**	**(n=3647)**
**Secondary Outcome,** Median (IQR) N					
Volume of Red Cells Transfused	2.0 (1.0, 2.0) 1813	2.0 (1.0, 2.0) 1829	2.0 (1.0, 2.0) 1671	2.0 (1.0, 2.0) 1971	2.0 (1.0, 2.0) 3642
**Supportive Outcomes,**[1] N(%)					
**Red cell component of primary outcome**					
Appropriate	1497 (82.5%)	1438 (78.5%)	1368 (81.7%)	1567 (79.4%)	2935 (80.5%)
Outside Guidelines	173 (9.5%)	209 (11.4%)	169 (10.1%)	213 (10.8%)	382 (10.5%)
Unclassified	145 (8.0%)	185 (10.1%)	137 (8.2%)	193 (9.8%)	330 (9.0%)
**Intermediate Outcomes,** N(%)					
**Hb measured after each unit transfused**	63 (4.9%)	39 (3.1%)	50 (4.3%)	52 (3.7%)	102 (4.0%)
**Platelet Transfusions**	**(n=729)**	**(n=717)**	**(n=633)**	**(n=813)**	**(n=1446)**

© 2022 Stanworth SJ et al. *JAMA Network Open*.

	CONTENT		SUPPORT		
**Variable**	**STANDARD**	**ENHANCED**	**STANDARD**	**ENHANCED**	**Total**
**Secondary Outcome,** Median (IQR) N					
Volume of Platelets Transfused	1.0 (1.0, 1.0) 716	1.0 (1.0, 1.0) 705	1.0 (1.0, 1.0) 626	1.0 (1.0, 1.0) 795	1.0 (1.0, 1.0) 1421
**Supportive Outcomes,** N(%)					
**Platelet component of primary outcome**					
Appropriate	279 (38.3%)	241 (33.6%)	227 (35.9%)	293 (36.0%)	520 (36.0%)
Outside Guidelines	315 (43.2%)	337 (47.0%)	299 (47.2%)	353 (43.4%)	652 (45.1%)
Unclassified	135 (18.5%)	139 (19.3%)	107 (16.9%)	167 (20.6%)	174 (19.0%)
**Intermediate Outcomes,** N(%)					
**Platelet count measured after each unit transfused**	30 (45.5%)	11 (21.6%)	20 (39.2%)	21 (31.8%)	41 (35.0%)

[1]Audit Standards, Haematology Standards 1, 2, 3,6 and 7

Standard 1: Clinical staff should measure Hb prior to transfusion of red blood cells in haematology patients

Standard 2: Clinical staff should only transfuse red blood cells in normovolaemic asymptomatic haematology inpatients without additional risk factors (cardiovascular disease or signs or symptoms of cardiovascular compromise, severe sepsis or acute cerebral ischaemia) if their pre-transfusion Hb is less than 70g/L

Standard 3: Clinical staff should only transfuse red blood cells in haematology inpatients with cardiovascular disease or signs or symptoms of cardiovascular compromise if their pre-transfusion Hb is less than 80g/L

Standard 6: Clinical staff should only transfuse prophylactic platelets in patients with a reversible cause for bone marrow failure and no other risk factors for bleeding if their pre-transfusion platelet count is below 10 x 10^9/L

Standard 7: Clinical staff should avoid routinely prescribing prophylactic platelet transfusions to patients with irreversible chronic bone marrow failure

© 2022 Stanworth SJ et al. *JAMA Network Open*.

Table A13: Haematology Trial - Primary and Sensitivity Analyses

Analysis	Unadjusted Proportion Appropriate STANDARD	Unadjusted Proportion Appropriate ENHANCED	Estimated Adjusted Risk Difference (95% CI)	Estimated Adjusted Odds Ratio (97.5% CI)	Estimated Adjusted Odds Ratio (95% CI)	p-value	N
**Primary Analysis (multiple imputation, 100 imputations, full imputation model)**							
Content	0.744	0.714	-0.04 (-0.09 to 0.02)	0.82 (0.59 to 1.15)	0.82 (0.61 to 1.10)	0.193	3859
Support	0.739	0.721	-0.01 (-0.06 to 0.05)	0.96 (0.67 to 1.38)	0.96 (0.70 to 1.32)	0.811	3859
Interaction	0.737	0.707	0.02 (-0.10 to 0.13)	1.15 (0.56 to 2.34)	1.15 (0.61 to 2.14)	0.668	3859
**Sensitivity Analyses**							
**Complete Case Analysis**							
Content	0.741	0.707	-0.04 (-0.09 to 0.02)	0.82 (0.59 to 1.15)	0.82 (0.61 to 1.10)	0.193	3498
Support	0.734	0.716	-0.01 (-0.06 to 0.05)	0.96 (0.67 to 1.38)	0.96 (0.70 to 1.32)	0.811	3498
Interaction	0.734	0.699	0.02 (-0.10 to 0.13)	1.15 (0.56 to 2.34)	1.15 (0.61 to 2.14)	0.668	3498

© 2022 Stanworth SJ et al. *JAMA Network Open*.

Table A14: Haematology Trial - Patient-Level Supportive Analysis

Analysis	Unadjusted Proportion Appropriate STANDARD	Unadjusted Proportion Appropriate ENHANCED	Estimated Adjusted Odds Ratio (95% CI)	p-value	N
**Red cell Component of the Primary Outcome[1]**					
Content	0.889	0.869	0.73 (0.45 to 1.18)	0.204	3647
Support	0.887	0.873	0.92 (0.52 to 1.61)	0.767	3647
Interaction	0.886	0.862	1.30 (0.46 to 3.69)	0.623	3647
**Platelet Component of the Primary Outcome[1]***					
Content	0.476	0.422	0.77 (0.54 to 1.11)	0.167	1247
Support	0.437	0.459	1.11 (0.78 to 1.60)	0.558	1247
Interaction	0.445	0.459	1.90 (0.92 to 3.91)	0.083	1247
**NCA Audit Standard 1[2]*[3]**					
Content	0.944	0.932	0.71 (0.43 to 1.19)	0.194	3643
Support	0.940	0.937	0.97 (0.58 to 1.61)	0.897	3643
Interaction	0.942	0.930	0.97 (0.35 to 2.70)	0.957	3643
**NCA Audit Standard 2[1]***					
Content	0.304	0.237	0.66 (0.44 to 0.98)	0.041	896
Support	0.287	0.257	1.01 (0.68 to 1.52)	0.947	896
Interaction	0.279	0.250	1.89 (0.84 to 4.25)	0.121	896
**NCA Audit Standard 6[1]***					
Content	0.598	0.548	0.71 (0.40 to 1.25)	0.235	376
Support	0.579	0.567	1.07 (0.61 to 1.90)	0.809	376
Interaction	0.560	0.598	3.59 (1.25 to 11.31)	0.029	376
**NCA Audit Standard 7[2]***					
Content	0.593	0.684	1.37 (0.89 to 2.10)	0.146	1119
Support	0.615	0.657	0.94 (0.62 to 1.45)	0.792	1119
Interaction	0.612	0.706	1.29 (0.55 to 3.04)	0.554	1119

NOTE: [1] multiple imputation was used (100 imputations, full imputation model). [2] complete case analysis as no missing data. * Reduced analysis model to facilitate model convergence, design factors omitted as cluster-level covariates

[3]Audit Standards, Haematology Standards 1, 2, 3,6 and 7

Standard 1: Clinical staff should measure Hb prior to transfusion of red blood cells in haematology patients

Standard 2: Clinical staff should only transfuse red blood cells in normovolaemic asymptomatic haematology inpatients without additional risk factors (cardiovascular disease or signs or symptoms of cardiovascular compromise, severe sepsis or acute cerebral ischaemia) if their pre-transfusion Hb is less than 70g/L

Standard 3: Clinical staff should only transfuse red blood cells in haematology inpatients with cardiovascular disease or signs or symptoms of cardiovascular compromise if their pre-transfusion Hb is less than 80g/L

Standard 6: Clinical staff should only transfuse prophylactic platelets in patients with a reversible cause for bone marrow failure and no other risk factors for bleeding if their pre-transfusion platelet count is below 10 x 10^9/L

Standard 7: Clinical staff should avoid routinely prescribing prophylactic platelet transfusions to patients with irreversible chronic bone marrow failure

© 2022 Stanworth SJ et al. *JAMA Network Open*.

Table A15: Haematology Trial - Cluster-Level SHOT Outcomes at Baseline and Follow-Up

	CONTENT		SUPPORT		
**Variable**	**STANDARD**	**ENHANCED**	**STANDARD**	**ENHANCED**	**Total**
**Entire SHOT Sample**	**(n=68)**	**(n=66)**	**(n=67)**	**(n=67)**	**(n=134)**
**Total Number of Incidents**, Median (IQR) (Range)					
Baseline	12 (6.5, 25) (0, 189)	14 (7, 21) (0, 71)	15 (9, 25) (0, 56)	12 (6, 22) (0, 189)	13 (7, 24) (0, 189)
Follow-Up	15.5 (7, 25) (0, 175)	15 (8, 26) (0, 74)	16 (8, 26) (0, 74)	13 (6, 25) (0, 175)	15 (8, 25) (0, 175)
Jul-Sep 2015 (Baseline)	3 (2, 6) (0, 47)	4.5 (2, 7) (0, 24)	4 (3, 7) (0, 16)	3 (1, 7) (0, 47)	4 (2, 7) (0, 47)
Oct-Dec 2015 (Baseline)	3 (1, 6) (0, 71)	3 (1, 6) (0, 18)	3 (1, 6) (0, 32)	3 (1, 6) (0, 71)	3 (1, 6) (0, 71)
Jan-Mar 2016 (Baseline)	3.5 (2, 6) (0, 39)	3 (1, 6) (0, 14)	3 (2, 6) (0, 13)	3 (1, 6) (0, 39)	3 (2, 6) (0, 39)
Apr-Jun 2016 (Baseline)	3 (1, 6) (0, 32)	3 (1, 5) (0, 23)	3 (2, 6) (0, 16)	2 (1, 6) (0, 32)	3 (1, 6) (0, 32)
Jul-Sep 2016 (Follow-Up)	4 (2, 6) (0, 33)	3.5 (2, 7) (0, 18)	4 (2, 6) (0, 17)	4 (2, 7) (0, 33)	4 (2, 7) (0, 33)
Oct-Dec 2016 (Follow-Up)	3 (1, 6.5) (0, 55)	3.5 (2, 8) (0, 26)	4 (2, 8) (0, 26)	2 (1, 7) (0, 55)	3 (1, 7) (0, 55)
Jan-Mar 2017 (Follow-Up)	4 (1.5, 7) (0, 47)	3 (2, 6) (0, 22)	4 (2, 7) (0, 18)	3 (1, 6) (0, 47)	4 (2, 6) (0, 47)
Apr-Jun 2017 (Follow-Up)	3 (1, 6) (0, 43)	3 (2, 7) (0, 18)	3 (2, 6) (0, 18)	3 (1, 7) (0, 43)	3 (1, 6) (0, 43)
**Number of Relevant Errors**, Median (IQR) (Range)					
Baseline	1 (0, 1.5) (0, 11)	1 (0, 1) (0, 11)	1 (0, 2) (0, 11)	0 (0, 1) (0, 11)	1 (0, 1) (0, 11)
Follow-Up	0 (0, 1) (0, 8)	0 (0, 1) (0, 21)	0 (0, 1) (0, 4)	0 (0, 1) (0, 21)	0 (0, 1) (0, 21)
Jul-Sep 2015 (Baseline)	0 (0, 0) (0, 2)	0 (0, 1) (0, 4)	0 (0, 1) (0, 4)	0 (0, 0) (0, 2)	0 (0, 0) (0, 4)
Oct-Dec 2015 (Baseline)	0 (0, 0.5) (0, 4)	0 (0, 0) (0, 11)	0 (0, 0) (0, 11)	0 (0, 0) (0, 4)	0 (0, 0) (0, 11)
Jan-Mar 2016 (Baseline)	0 (0, 0) (0, 4)	0 (0, 0) (0, 3)	0 (0, 0) (0, 3)	0 (0, 0) (0, 4)	0 (0, 0) (0, 4)
Apr-Jun 2016 (Baseline)	0 (0, 0) (0, 3)	0 (0, 0) (0, 4)	0 (0, 0) (0, 4)	0 (0, 0) (0, 4)	0 (0, 0) (0, 4)
Jul-Sep 2016 (Follow-Up)	0 (0, 0) (0, 3)	0 (0, 0) (0, 2)	0 (0, 0) (0, 3)	0 (0, 0) (0, 2)	0 (0, 0) (0, 3)
Oct-Dec 2016 (Follow-Up)	0 (0, 0) (0, 3)	0 (0, 0) (0, 2)	0 (0, 0) (0, 3)	0 (0, 0) (0, 3)	0 (0, 0) (0, 3)
Jan-Mar 2017 (Follow-Up)	0 (0, 0) (0, 2)	0 (0, 0) (0, 21)	0 (0, 0) (0, 1)	0 (0, 0) (0, 21)	0 (0, 0) (0, 21)
Apr-Jun 2017 (Follow-Up)	0 (0, 0) (0, 2)	0 (0, 0) (0, 3)	0 (0, 0) (0, 3)	0 (0, 0) (0, 2)	0 (0, 0) (0, 3)
**Number of Relevant Near Miss for Right-Blood Right-Patient Incidents**, Median (IQR) (Range)					
Baseline	0 (0, 1) (0, 3)	0 (0, 0) (0, 3)	0 (0, 1) (0, 1)	0 (0, 0) (0, 3)	0 (0, 0) (0, 3)
Follow-Up	0 (0, 0) (0, 2)	0 (0, 0) (0, 2)	0 (0, 0) (0, 1)	0 (0, 0) (0, 2)	0 (0, 0) (0, 2)
Jul-Sep 2015 (Baseline)	0 (0, 0) (0, 1)	0 (0, 0) (0, 1)	0 (0, 0) (0, 1)	0 (0, 0) (0, 1)	0 (0, 0) (0, 1)
Oct-Dec 2015 (Baseline)	0 (0, 0) (0, 1)	0 (0, 0) (0, 1)	0 (0, 0) (0, 1)	0 (0, 0) (0, 1)	0 (0, 0) (0, 1)
Jan-Mar 2016 (Baseline)	0 (0, 0) (0, 2)	0 (0, 0) (0, 1)	0 (0, 0) (0, 1)	0 (0, 0) (0, 2)	0 (0, 0) (0, 2)
Apr-Jun 2016 (Baseline)	0 (0, 0) (0, 2)	0 (0, 0) (0, 2)	0 (0, 0) (0, 1)	0 (0, 0) (0, 2)	0 (0, 0) (0, 2)
Jul-Sep 2016 (Follow-Up)	0 (0, 0) (0, 1)	0 (0, 0) (0, 1)	0 (0, 0) (0, 1)	0 (0, 0) (0, 1)	0 (0, 0) (0, 1)
Oct-Dec 2016 (Follow-Up)	0 (0, 0) (0, 1)	0 (0, 0) (0, 1)	0 (0, 0) (0, 1)	0 (0, 0) (0, 1)	0 (0, 0) (0, 1)
Jan-Mar 2017 (Follow-Up)	0 (0, 0) (0, 1)	0 (0, 0) (0, 2)	0 (0, 0) (0, 1)	0 (0, 0) (0, 2)	0 (0, 0) (0, 2)

© 2022 Stanworth SJ et al. *JAMA Network Open*.

	CONTENT		SUPPORT		
**Variable**	**STANDARD**	**ENHANCED**	**STANDARD**	**ENHANCED**	**Total**
Apr-Jun 2017 (Follow-Up)	0 (0, 0) (0, 1)	0 (0, 0) (0, 0)	0 (0, 0) (0, 0)	0 (0, 0) (0, 1)	0 (0, 0) (0, 1)
**Total Number of Unpredictable Incidents**, Median (IQR) (Range)					
Baseline	1 (0, 2.5) (0, 11)	1 (0, 2) (0, 15)	1 (0, 3) (0, 7)	1 (0, 2) (0, 15)	1 (0, 2) (0, 15)
Follow-Up	1 (0, 2.5) (0, 9)	1 (0, 3) (0, 16)	1 (0, 3) (0, 16)	1 (0, 2) (0, 9)	1 (0, 3) (0, 16)
Jul-Sep 2015 (Baseline)	0 (0, 1) (0, 5)	0 (0, 1) (0, 5)	0 (0, 1) (0, 3)	0 (0, 0) (0, 5)	0 (0, 1) (0, 5)
Oct-Dec 2015 (Baseline)	0 (0, 1) (0, 4)	0 (0, 1) (0, 2)	0 (0, 0) (0, 4)	0 (0, 1) (0, 2)	0 (0, 1) (0, 4)
Jan-Mar 2016 (Baseline)	0 (0, 0) (0, 4)	0 (0, 1) (0, 3)	0 (0, 1) (0, 4)	0 (0, 0) (0, 3)	0 (0, 1) (0, 4)
Apr-Jun 2016 (Baseline)	0 (0, 1) (0, 3)	0 (0, 0) (0, 6)	0 (0, 1) (0, 3)	0 (0, 1) (0, 6)	0 (0, 1) (0, 6)
Jul-Sep 2016 (Follow-Up)	0 (0, 1) (0, 3)	0 (0, 1) (0, 3)	0 (0, 1) (0, 3)	0 (0, 0) (0, 3)	0 (0, 1) (0, 3)
Oct-Dec 2016 (Follow-Up)	0 (0, 0.5) (0, 4)	0 (0, 1) (0, 6)	0 (0, 0) (0, 6)	0 (0, 1) (0, 4)	0 (0, 1) (0, 6)
Jan-Mar 2017 (Follow-Up)	0 (0, 0) (0, 3)	0 (0, 1) (0, 8)	0 (0, 1) (0, 8)	0 (0, 0) (0, 4)	0 (0, 1) (0, 8)
Apr-Jun 2017 (Follow-Up)	0 (0, 1) (0, 3)	0 (0, 1) (0, 3)	0 (0, 1) (0, 3)	0 (0, 0) (0, 3)	0 (0, 1) (0, 3)
**Total Number of Possibly Preventable Incidents**, Median (IQR) (Range)					
Baseline	0 (0, 2) (0, 48)	0 (0, 1) (0, 6)	1 (0, 1) (0, 7)	0 (0, 2) (0, 48)	0 (0, 2) (0, 48)
Follow-Up	0 (0, 1.5) (0, 13)	0 (0, 1) (0, 8)	0 (0, 1) (0, 6)	0 (0, 1) (0, 13)	0 (0, 1) (0, 13)
Jul-Sep 2015 (Baseline)	0 (0, 0.5) (0, 18)	0 (0, 0) (0, 5)	0 (0, 1) (0, 5)	0 (0, 0) (0, 18)	0 (0, 0) (0, 18)
Oct-Dec 2015 (Baseline)	0 (0, 0) (0, 28)	0 (0, 1) (0, 4)	0 (0, 0) (0, 6)	0 (0, 0) (0, 28)	0 (0, 0) (0, 28)
Jan-Mar 2016 (Baseline)	0 (0, 0) (0, 2)	0 (0, 0) (0, 3)	0 (0, 0) (0, 1)	0 (0, 0) (0, 3)	0 (0, 0) (0, 3)
Apr-Jun 2016 (Baseline)	0 (0, 0) (0, 3)	0 (0, 0) (0, 1)	0 (0, 0) (0, 3)	0 (0, 0) (0, 2)	0 (0, 0) (0, 3)
Jul-Sep 2016 (Follow-Up)	0 (0, 0) (0, 3)	0 (0, 0) (0, 7)	0 (0, 0) (0, 3)	0 (0, 0) (0, 7)	0 (0, 0) (0, 7)
Oct-Dec 2016 (Follow-Up)	0 (0, 0) (0, 4)	0 (0, 0) (0, 2)	0 (0, 0) (0, 2)	0 (0, 0) (0, 4)	0 (0, 0) (0, 4)
Jan-Mar 2017 (Follow-Up)	0 (0, 0) (0, 3)	0 (0, 1) (0, 2)	0 (0, 1) (0, 3)	0 (0, 0) (0, 3)	0 (0, 0) (0, 3)
Apr-Jun 2017 (Follow-Up)	0 (0, 0) (0, 4)	0 (0, 0) (0, 2)	0 (0, 0) (0, 2)	0 (0, 0) (0, 4)	0 (0, 0) (0, 4)

© 2022 Stanworth SJ et al. *JAMA Network Open*.

Table A16: Haematology Trial - Cluster-Level BSMS Outcomes at Baseline and Follow-Up

	CONTENT		SUPPORT		
**Variable**	**STANDARD**	**ENHANCED**	**STANDARD**	**ENHANCED**	**Total**
**Entire BSMS Sample**	**(n=68)**	**(n=66)**	**(n=67)**	**(n=67)**	**(n=134)**
**Total Volume of RBC Transfused**, Median (IQR) (Range) N					
Baseline	7724.5 (5298, 11183) (1024, 30449) 66	7662 (5243, 11838) (1764, 38434) 65	6747 (4730.5, 11247.5) (1727, 38434) 64	8532 (5316, 11838) (1024, 30449) 67	7662 (5243, 11514) (1024, 38434) 131
Follow-Up	6793 (4843, 11481) (1216, 29892) 65	7560 (4677, 11483) (1263, 29014) 66	6480 (4479.5, 11514.5) (1263, 29892) 64	7769 (4843, 10977) (1216, 29014) 67	7107 (4677, 11483) (1216, 29892) 131
Jul-Sep 2015 (Baseline)	2026.5 (1406.5, 2782) (433, 7655) 64	2037.5 (1345, 2907) (712, 10604) 62	1896 (1278, 2771) (712, 10604) 61	2137 (1418, 2946) (433, 7655) 65	2026.5 (1375, 2878) (433, 10604) 126
Oct-Dec 2015 (Baseline)	1994 (1370, 2883) (687, 7897) 63	2154 (1370, 2917) (368, 9865) 63	1889 (1188, 2858) (368, 9865) 61	2198 (1439, 2917) (687, 7897) 65	2025 (1370, 2883) (368, 9865) 126
Jan-Mar 2016 (Baseline)	2060.5 (1269, 2713.5) (648, 7655) 64	2003.5 (1224.5, 2999.5) (535, 9841) 64	1826.5 (1143, 2689) (648, 9841) 62	2101.5 (1274, 2929) (535, 8020) 66	2030 (1247.5, 2822.5) (535, 9841) 128
Apr-Jun 2016 (Baseline)	1987 (1293, 2665) (275, 7637) 65	1946.5 (1276.5, 3023) (671, 8124) 64	1937 (1246, 3169) (671, 8124) 63	2105 (1293, 3001) (275, 7242) 66	1965 (1288, 3001) (275, 8124) 129
Jul-Sep 2016 (Follow-Up)	1818.5 (1257.5, 2979.5) (352, 7912) 64	2088 (1176, 3114) (618, 7680) 63	1818.5 (1153, 2984) (618, 7912) 62	2088 (1329, 2836) (352, 7164) 65	2019 (1214, 2984) (352, 7912) 127
Oct-Dec 2016 (Follow-Up)	1791 (1222, 2722) (441, 7453) 65	1953 (1274, 2926) (384, 7618) 63	1850 (1097, 2859) (384, 7453) 62	1939 (1300, 2722) (441, 7618) 66	1927 (1231, 2843) (384, 7618) 128
Jan-Mar 2017 (Follow-Up)	1898 (1275, 2908) (323, 7145) 63	1930 (1145, 2940) (659, 7683) 63	1811.5 (1094, 2913) (627, 7145) 62	1955.5 (1300.5, 3025) (323, 7683) 64	1918.5 (1197, 2913) (323, 7683) 126
Apr-Jun 2017 (Follow-Up)	1733 (1240.5, 2861.5) (268, 7382) 64	1794 (1152, 2995.5) (473, 7786) 64	1558 (1141, 2861.5) (473, 7382) 64	1886.5 (1273, 3071) (268, 7786) 64	1769.5 (1194.5, 2869.5) (268, 7786) 128
**Total Gross Volume of RBC Issued**, Median (IQR) (Range) N					
Baseline	8489 (5456, 11667) (2823, 31009) 67	8483.5 (5599, 11925) (3135, 41792) 66	8203.5 (5483, 11667) (2968, 41792) 66	8760 (5535, 11925) (2823, 32230) 67	8489 (5520, 11784) (2823, 41792) 133
Follow-Up	7996 (4958, 11480) (2900, 30850) 67	8213 (5096, 11800) (2801, 43714) 66	7816 (4958, 11678) (2801, 43714) 66	8129 (5108, 11746) (2900, 32254) 67	8050 (5096, 11678) (2801, 43714) 133
Jul-Sep 2015 (Baseline)	2220 (1432, 2933) (704, 7811) 67	2139 (1416, 2987) (738, 11013) 66	2099.5 (1416, 2956) (738, 11013) 66	2202 (1455, 3025) (704, 8126) 67	2144 (1423, 2965) (704, 11013) 133
Oct-Dec 2015 (Baseline)	2064 (1348, 2891) (713, 8029) 67	2167.5 (1433, 3026) (782, 10292) 66	2038 (1315, 2970) (780, 10292) 66	2225 (1433, 2930) (713, 8029) 67	2118 (1425, 2948) (713, 10292) 133
Jan-Mar 2016 (Baseline)	2159 (1353, 2838) (681, 7782) 67	2038 (1428, 2889) (782, 10187) 66	2080 (1381, 2835) (681, 10187) 66	2159 (1392, 3060) (694, 8283) 67	2115 (1392, 2838) (681, 10187) 133
Apr-Jun 2016 (Baseline)	2122 (1316, 2850) (712, 7812) 67	2119 (1435, 3050) (774, 10300) 66	2039.5 (1316, 2937) (732, 10300) 66	2220 (1393, 3021) (712, 8221) 67	2122 (1351, 2945) (712, 10300) 133
Jul-Sep 2016 (Follow-Up)	1998 (1282, 2916) (745, 8099) 67	2115.5 (1340, 3003) (647, 10927) 66	1926 (1282, 3003) (647, 10927) 66	2192 (1296, 2916) (745, 8053) 67	2104 (1296, 2954) (647, 10927) 133
Oct-Dec 2016 (Follow-Up)	2013 (1288, 2870) (742, 7646) 67	2041.5 (1301, 2966) (680, 11042) 66	2010.5 (1253, 2960) (680, 11042) 66	2038 (1301, 2966) (742, 8392) 67	2037 (1301, 2960) (680, 11042) 133

© 2022 Stanworth SJ et al. *JAMA Network Open*.

	CONTENT		SUPPORT		
**Variable**	**STANDARD**	**ENHANCED**	**STANDARD**	**ENHANCED**	**Total**
Jan-Mar 2017 (Follow-Up)	1940 (1291, 2873) (672, 7439) 67	2031 (1266, 2962) (765, 11127) 66	1967 (1241, 2939) (672, 11127) 66	1984 (1318, 2928) (715, 8351) 67	1984 (1276, 2928) (672, 11127) 133
Apr-Jun 2017 (Follow-Up)	2034 (1279, 2820) (618, 7666) 67	1968 (1299, 3043) (709, 10618) 66	1934 (1279, 2924) (708, 10618) 66	1979 (1327, 3043) (618, 8032) 67	1979 (1299, 2924) (618, 10618) 133
**Total Volume of RBC Wasted**, Median (IQR) (Range) N					
Baseline	198 (106, 279) (26, 1406) 66	154 (103, 314) (31, 1450) 65	190 (111.5, 269) (32, 1450) 64	168 (101, 315) (26, 1406) 67	184 (103, 313) (26, 1450) 131
Follow-Up	189 (104, 300) (19, 1713) 65	155.5 (79, 309) (6, 1665) 66	187 (85, 296) (6, 1713) 64	178 (89, 324) (17, 1355) 67	180 (86, 309) (6, 1713) 131
Jul-Sep 2015 (Baseline)	46.5 (30, 75) (2, 392) 64	37 (25, 72) (7, 409) 62	47 (31, 81) (7, 409) 61	36 (25, 70) (2, 392) 65	40.5 (28, 72) (2, 409) 126
Oct-Dec 2015 (Baseline)	49 (24, 73) (6, 316) 63	35 (23, 79) (2, 427) 63	43 (24, 73) (2, 427) 61	46 (23, 72) (6, 305) 65	44.5 (24, 73) (2, 427) 126
Jan-Mar 2016 (Baseline)	48.5 (24.5, 75.5) (6, 378) 64	46.5 (27.5, 90) (3, 346) 64	48.5 (27, 70) (3, 346) 62	45.5 (27, 84) (5, 378) 66	47 (27, 82.5) (3, 378) 128
Apr-Jun 2016 (Baseline)	54 (23, 79) (4, 442) 65	43.5 (23.5, 84.5) (5, 268) 64	46 (23, 75) (5, 442) 63	49.5 (23, 82) (4, 331) 66	47 (23, 80) (4, 442) 129
Jul-Sep 2016 (Follow-Up)	50 (25.5, 79) (2, 458) 64	43 (24, 89) (8, 374) 63	43.5 (24, 84) (4, 458) 62	48 (25, 87) (2, 340) 65	44 (24, 85) (2, 458) 127
Oct-Dec 2016 (Follow-Up)	42 (25, 68) (4, 323) 65	40 (18, 69) (6, 438) 63	41.5 (20, 64) (4, 438) 62	40 (20, 70) (5, 295) 66	40.5 (20, 68.5) (4, 438) 128
Jan-Mar 2017 (Follow-Up)	45 (23, 77) (2, 541) 63	43 (20, 75) (4, 424) 63	47 (23, 69) (4, 541) 62	42 (21, 83.5) (2, 276) 64	43.5 (21, 75) (2, 541) 126
Apr-Jun 2017 (Follow-Up)	49 (28, 78) (1, 510) 64	37 (23, 70.5) (2, 541) 64	45 (25, 69) (2, 510) 64	45.5 (26, 87.5) (1, 541) 64	45 (25.5, 76) (1, 541) 128

© 2022 Stanworth SJ et al. *JAMA Network Open*.

eAppendix 4. Economic Analysis

This economic analysis was performed using 2017 UK GBP, using unit costs relevant to a UK setting. To convert the figures presented to USD, we recommend dividing the GBP values by 0.685, which is the Organisation for Economic Co-operation and Development (OECD) Purchasing Power Parity rate for GBP/USD for 2017.

Contents

Methodology – supplemental 35
- Design overview and model 35
- Costing exercise methodology 35
  - Parameters 1: Cost of collecting audit data 36
  - Parameters 2: Cost of developing and delivering audit reports 36
  - Parameters 3: Cost of delivering follow-on-support programmes 37
  - Parameters 4: Cost of additional activity by NHS staff as a result of receiving audit report 37
  - Parameters 5: Cost of volume of blood transfused 38
- Unit costs 38
- Outcome parameters 38
- Cost-Effectiveness analysis methodology 38
- Sensitivity Analysis methodology 39
- Budget impact and intervention cost-neutral analyses 39

Results - supplemental 39
- Intervention costs and budget impact 39
- Cost-neutral analysis 39
- Cost-Effectiveness Analysis Results – supplemental 40
  - Trial 1 – Secondary Outcomes: Volume of blood transfused and number of SHOT events 40
  - Trial 2 - Primary outcome: Percentage of transfusions acceptable 40
  - Trial 2 – Secondary Outcomes: Volume of blood transfused and number of SHOT events 40
- Sensitivity Analysis– Trial 1 41
  - Percentage of transfusions acceptable (Enhanced vs Standard Content) 41

© 2022 Stanworth SJ et al. *JAMA Network Open*.

Percentage of transfusions acceptable (Enhanced vs Standard Follow-on Support) 59
Volume of blood transfused (Enhanced vs Standard Content) 59
Volume of blood transfused (Enhanced vs Standard Follow-on Support) 59
Number of SHOT events (Enhanced vs Standard Content) 60
Trial 1 – Number of SHOT events (Enhanced vs Standard Follow-on Support) 60
Sensitivity Analysis Results – Trial 2 60
Percentage of transfusions acceptable (Enhanced vs Standard Content) 60
Percentage of transfusions acceptable (Enhanced vs Standard Follow-on Support) 61
Volume of blood transfused (Enhanced vs Standard Content) 61
Trial 2 – Volume of blood transfused (Enhanced vs Standard Follow-on Support) 61
Trial 2 – Number of SHOT events (Enhanced vs Standard Content) 62
Trial 2 – Number of SHOT events (Enhanced vs Standard Follow-on Support) 62
Supplementary Figures and Tables 63

Methodology – supplemental

Design overview and model

The analysis was conducted using decision analytic modelling from the perspective of the NHS. For each of Trials 1 and 2 we compared the costs and the outcomes of the 'enhanced content' vs 'usual content' arms, and the 'enhanced follow-on support' vs 'usual follow-on support' arms (Figure 1). We explored uncertainty around the parameters used in the model using sensitivity analysis.

The primary outcome of the trials was the proportion of transfusions given that were acceptable. For ease of understanding the Incremental Cost-Effectiveness Ratios (ICERs) presented in this analysis we multiplied those proportions by 100 so that they were percentages. Therefore, the primary outcome of this analysis was the percentage of acceptable transfusions. Secondary outcomes comprised the volume of blood transfused and the number of SHOT-reportable incidents. The time horizon for this analysis was one year, during which two rounds of audits took place for each trial. We applied no discounting.

Costing exercise methodology

The costs of the intervention were collected from a combination of top-down (gross-costing) and bottom-up (micro-costing) methods, depending on the type and quality of data available. Top-down involved for example, dividing an overall cost by the number of participating staff or sites; bottom-

© 2022 Stanworth SJ et al. *JAMA Network Open*.

up involved for example, calculating an average cost-per-participating-staff-member-per-site and multiplying up by the number of sites. All costs are expressed in 2017/18 UK pounds. All salary costs used were taken at the approximated midpoint of the NHS 2017/18 salary band. For the purposes of this analysis there were 185 sites in Trial 1 and 194 sites in Trial 2.

The costs given are broken down into:

- Cost of collecting audit data
    - The costs of collecting audit data were divided into cost of time by NHS staff completing audit booklets, and time inputting this data into NHSBT's online system. The inputting data was further broken down into NHS and NHSBT staff time inputting data.
- Cost of developing and delivering the audit reports
    - (intervention report vs usual report).
- Cost of delivering the Follow-on Support interventions
    - (intervention support vs usual support/no support).
- Cost of extra activity conducted by sites as a result of participating audit.
- Cost of volume of blood transfused.

For each of the analyses (Figure 1), these costs have been aggregated for ease of understanding into:

- The costs of audit components: consisting of the costs of NHS and NHSBT staff time in recording audit data in audit booklets and uploading them on the NHSBT system;
- The costs of the feedback components: consisting of the telephone intervention (both research staff and NHS staff); and the online toolkit (running costs and NHS staff time spent using the toolkit);
- The costs of writing and delivering audit reports: consisting of paid staff time in developing the content of both the standard and enhanced reports and the 'production' costs of delivering the audit to all participating sites.

The unit costs presented here are given at site level, and we have attempted to be as clear as possible regarding whether a particular cost applies to a single audit round, or both audit rounds of a given Trial. All costs presented have be rounded to the nearest whole number (where appropriate).

Parameters 1: Cost of collecting audit data

The total cost of collecting audit data for Trial 1 was £93,841; and for Trial 2 was £110,507. These costs were divided equally between comparison arms in the cost-effectiveness analysis and the uncertainty around these estimates were explored in the probabilistic sensitivity analysis. These estimates were based on data obtained from NHSBT. The data on audit-book completion time was self-reported by clinicians as they completed the booklets. The data on inputting online was drawn from system logs. See Table 1 and Table 2.

Parameters 2: Cost of developing and delivering audit reports

© 2022 Stanworth SJ et al. *JAMA Network Open*.

The costs presented in Table 3 and Table 4 represent 2 audit rounds. No data was available on estimating the variance around each of these values. The estimates are based on a combination of minutes from report-writing meetings and training sessions, as well as conversations with AFFINITIE and NHSBT staff on their roles and time spent working on standard and enhanced reports.

Parameters 3: Cost of delivering follow-on-support programmes

The costs of the telephone support service (TSS) and the online toolkit are both presented in Table 5 and Table 6. The estimates for the TSS are based on call logs recorded by the telephone system, and on shift rotas for the research staff responsible for making and receiving calls to hospital staff. No information was available about who the calls were made to, so we have assumed that it was always a Band 7 (mid-point) nurse transfusions practitioner (TP) who was making/answer calls. TSS records contain data on shifts that were either 3 hours in the morning or 2.5hours in the afternoon for November 2015.

The usage costs of the online toolkit are based on system logs, and the development costs are based on consultation with the AFFINITIE and NHSBT teams. The development costs are not included in the economic evaluation as they are one-off costs and not relevant to the ongoing use of the toolkit. The toolkit was made available for both standard and enhanced arms of the trial, and records were kept on the usage level of the toolkit for both arms. We had no data on who was using the toolkit, so we have assumed that the NHS staff member using the online toolkit is always a mid-point Band 7 Nurse Transfusion Practitioner.

Parameters 4: Cost of additional activity by NHS staff as a result of receiving audit report

The data on additional activity that NHS staff undertook as a result of receiving the audit reports and interventions was measured as part of an online questionnaire. The questionnaire was primarily qualitative in focus, but included two questions in particular that were relevant to the economic evaluation. These were:

- Q17b. Please estimate approximately how many hours you spent planning your response to feedback. (free text)
- Q20b. Please specify approximately how many meetings the feedback materials were shared/ discussed at. (free text)

The level of missing data was problematic for this analysis, with only a fraction of the 76 sites in each arm responding to the questionnaire after cleaning (standard content arm = 26; enhanced content arm = 22; standard FoS arm = 22; enhanced FoS arm = 23). Furthermore, 18% of Q17b responses were missing and 10% of Q20b. The data for both questions was analysed in Stata15 using generalized linear modelling (using a gamma distribution).

For Question 17b we assumed the respondent and therefore hours worked was performed by a Nurse TP (mid-point band 7). Regarding question 20b, the composition of these meetings was unknown. However the majority of respondents noted that the meeting the report was shared at

© 2022 Stanworth SJ et al. *JAMA Network Open*.

was a “Haematology Transfusion Committee” (HTC) or a derivation thereof. Consultation with AFFINITIE team suggested that an HTC would be comprised of a minimum of 3 senior consultants (10 years+), 2 nurse specialists (Band 7), 2 managers (Band 8a) and 2 executive-level managers (Band 8d). We therefore estimated the cost of an HTC meeting would be £284 per hour.

For the purposes of the analysis, we assumed that the costs calculated would apply to all sites (for each trial), since all NHS trusts receive the reports and should have some discussion in the form of an HTC which would likely require some work done on the part of a relatively junior member of staff to prepare and present at this HTC. See Table 7 and Table 8.

Parameters 5: Cost of volume of blood transfused

Data on the volume of blood transfused is presented in Tables 10 and 11. This data was collected at cluster level, and was converted to site level in for this analysis by dividing the volume of blood transfused in each arm by the number of clusters in each arm and multiplying by the number of sites in each arm. For Trial 1 data was only available on the volume of red blood cells (RBC) transfused; for Trial 2 data was available for platelets as well as RBC. The unit cost of RBC and platelets are given “Unit costs” section of the main report. The number of sites is given in the “Budget impact and intervention cost-neutral analysis” section of the main report.

Unit costs

The cost used for purchasing a unit of blood was £128.99 and £185.86 for RBC and platelets respectively.[21] We applied a cost of £51.32 for transfusing a unit of blood. This was obtained using the value given for subsequent units of blood transfused in the costing statement of the NICE guidelines for blood transfusion and management,[22] and inflating the difference for 2017/18.

Therefore, the unit cost for one unit of RBC and at NHS pay scale 2017/18 was £175.78 and £229.51 respectively. Unit costs for staff time were drawn from the NHS pay scale 2017/18,[23] the BMA pay scales for junior doctors 2017/18,[24] and the BMA pay scales for consultants 2017/18.[25] Non-clinical staff costs were drawn from the healthcare or research organisation employing the relevant staff member (NHSBT, University of Leeds, or City University, London).

Outcome parameters

Outcome data were obtained from the outputs of the trial analyses. Where necessary, these outcomes were converted from mean-per-cluster to mean-per-site, to conform to the unit of analysis for this study. Table 10 presents the parameters used in the analysis for the primary and secondary outcomes for Trial 1, and Table 11 describes those for Trial 2.

Cost-Effectiveness analysis methodology

We calculated Incremental Cost-Effectiveness Ratios (ICERs) to measure the cost-effectiveness for each comparative analysis for Trials 1 & 2. The ICER is interpreted as the additional cost required to

© 2022 Stanworth SJ et al. *JAMA Network Open*.

produce one unit of benefit. For the primary outcome, benefit is defined as one additional percentage of blood transfused acceptably. For SHOT it is one less SHOT reportable event. For the volume of blood, we have taken the view that fewer units of blood transfused are preferred to more, so the ICER shows the additional cost per 1-unit reduction in the volume of blood transfused.

The ICER calculation shown in Table 12 and Table 13 demonstrates how the difference cost components were included in calculating the ICER, using the primary outcome as an example.

Sensitivity Analysis methodology

To explore the uncertainty around the parameters used in our model, we conducted probabilistic sensitivity analysis (PSA). We varied the costs of collecting audit data; the costs of the feedback interventions, the cost of additional NHS activity, the percentage of transfusions given that were acceptable, the volume of blood transfused, and the number of transfusion-related adverse events.

We presented our PSA results in terms of mean incremental difference and associated Uncertainty Intervals (UI) on a cost-effectiveness plane, and a cost-effectiveness acceptability curve (CEAC) was generated for each analysis.

Budget impact and intervention cost-neutral analyses

We calculated the impact of each intervention on all 185 participating sites in Trial 1, and 194 sites in Trial 2 in order to estimate the impact on the NHS budget at a national level. We then adopted a cost-neutral framework by asking ‘how many units of blood would need to be prevented from being transfused in order to be cost-neutral (i.e. to break even)?’ For this analysis we evaluated the cost of each intervention in terms of the equivalent cost in units of RBC. We did this by calculating the incremental costs of each pair of interventions (Enhanced minus Standard) for each Trial, and dividing the result by the cost of a unit of RBC.

Results - supplemental

Intervention costs and budget impact

Table 14 and Table 15 present the cost of each arm for each Trial respectively. The largest costs across all arms were the costs of the blood transfused. The smallest costs were those of the feedback interventions.

Excluding the cost of blood transfusions, For Trial 1 the incremental cost of the enhanced vs standard content intervention was £219 per site; and was £18 per site for the enhanced vs standard follow-on support intervention. For Trial 2 these figures were £248 and -£198. Due to the cost of additional NHS activity, enhanced follow-on support was cheaper per site than standard.

Cost-neutral analysis

© 2022 Stanworth SJ et al. *JAMA Network Open*.

For Trial 1, the mean incremental cost of the enhanced vs standard content intervention was £219 per site, and was £18 per site for enhanced vs standard follow-on support. Hence, the interventions would need to reduce the volume of unacceptable blood transfusions by 1.2, and 0.1 units of RBC per site respectively in order to be cost neutral.

For Trial 2, the mean incremental cost of the enhanced vs standard content intervention was £248 per site, and was -£198 for enhanced vs standard follow-on support. Hence, the interventions would need to reduce the volume of unacceptable blood transfusions by 1.4, and 0.0 units of RBC per site respectively in order to be cost neutral. The incremental costs and equivalent units of RBC are presented in Table 16. The results for trial 2 are shown in Table 17.

Cost-Effectiveness Analysis Results – supplemental

Trial 1 – Secondary Outcomes: Volume of blood transfused and number of SHOT events

The enhanced content intervention transfused 770 additional units of RBC per site vs standard content. Given the intervention costs in the main paper, and taking the view that fewer units of RBC are preferred to more, enhanced content is dominated by the standard intervention (it was more costly and less effective). Comparable results were found for enhanced follow-on support, which transfused 312 additional units of RBC per site vs standard follow-on support, and was therefore also dominated by the standard intervention.

Enhanced content saw a reduction in SHOT events of 0.2 per site vs standard content, generating an ICER of £735,927 per SHOT event prevented. Enhanced follow-on support saw an increase in SHOT events of 1.3 per site vs standard follow-on support, meaning the standard intervention was dominant.

Trial 2 - Primary outcome: Percentage of transfusions acceptable

The cost-per-site of the standard content and enhanced content was £674,603 and £689,303 respectively. Enhanced content cost more than the standard content by £14,700 per site, and saw a decrease of 3.0% in acceptable transfusions given. The cost-per-site of standard follow-on support and enhanced follow-on support was £582,236 and £616,514 respectively. Enhanced follow-on support cost more than standard follow-on support by £34,278 per site, and saw a decrease of 1.8% in acceptable transfusions given.

Therefore, the enhanced content intervention was dominated by the standard intervention, and the enhanced follow-on support intervention was also dominated by the standard intervention.

Trial 2 – Secondary Outcomes: Volume of blood transfused and number of SHOT events

© 2022 Stanworth SJ et al. *JAMA Network Open*.

The enhanced content intervention transfused 75 additional units of RBC per site vs standard content. Given the intervention costs in the main paper, and taking the view that fewer units of RBC are preferred to more, enhanced content is dominated by the standard intervention (it was more costly and less effective). Comparable results were found for enhanced follow-on support, which transfused 192 additional units of RBC per site vs standard follow-on support, and was therefore also dominated by the standard intervention.

Enhanced content saw a reduction in SHOT events of 0.4 per site vs standard content, generating an ICER of £40,612 per SHOT event prevented. Enhanced follow-on support saw an increase in SHOT events of 1.0 per site vs standard follow-on support, meaning the standard intervention was dominant.

Sensitivity Analysis– Trial 1

Percentage of transfusions acceptable (Enhanced vs Standard Content)

The results of this PSA are presented in the main text.

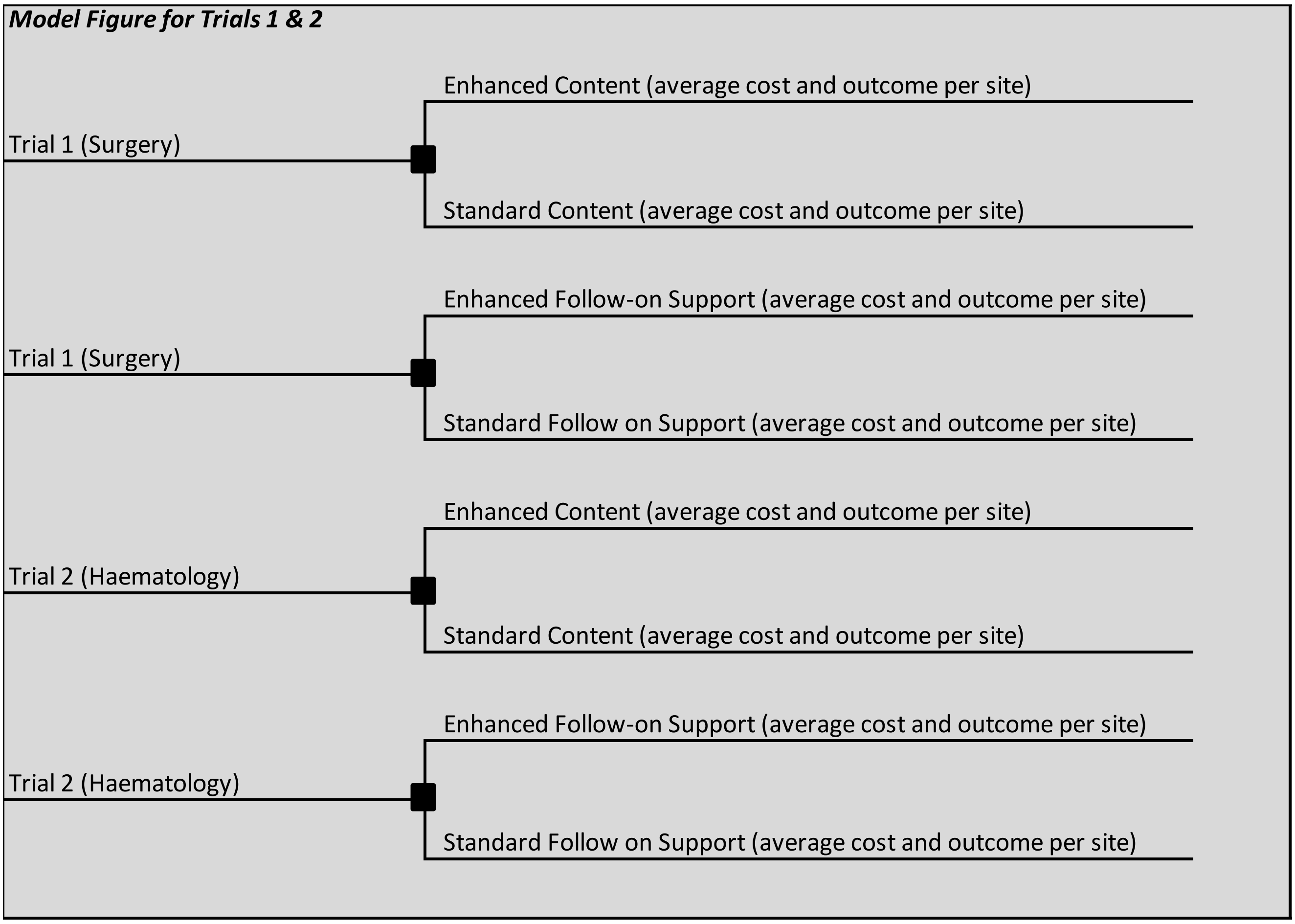

Figure 1: Cost-effectiveness analysis model for Trials 1 and 2

© 2022 Stanworth SJ et al. *JAMA Network Open*.

Table 1: Breakdown of costs of collecting audit data for Trial 1

	Value	Notes
Time and cost of completing audit booklets (Trial 1)		
Mean time take to complete audit booklets	28.4 minutes (SD = 55.5)	Median time was 27 minutes (IQR 18;37)
Weighted average salary for NHS staff	£38,387 per annum<br>£0.31 per minute	Based on description of 217 staff who completed booklet
*Number of patients audited in T1R1**	4035	
Total cost of time spent completing audit booklets T1R1	£35,211 (SD = £24,450)	
Number of patients audited in T1R2	3266	
Total cost of time spent completing audit booklets T1R2	£28,500 (SD = £19,790)	
Total cost of both audit rounds	£63,711 (SD = £44,240)	
Cost per site	£418 (SD = £290)	
Time and cost of inputting audit booklet data online (Trial 1)		
Mean time taken to enter data online	25.7 mins for NHS staff (SD = 55.5)<br>6.8 minutes for NHSBT staff (SD = 3.5)	Median time for NHS and NHSBT was 10 and 6 minutes respectively
Weighted average salary for NHSBT staff	£38,726 per annum	Based on records of staff tasked with inputting data

© 2022 Stanworth SJ et al. *JAMA Network Open*.

	£0.31 per minute	
Number of NHS entries	3769	These are system logs of data being entered, therefore not necessarily 1 entry per booklet
Cost to NHS of time spent inputting data	£29,840 (SD = £64,283)	
Number of NHSBT entries	138	These are audit booklets returned to NHSBT with data that need inputting online. Postage costs not included.
Cost to NHSBT of time spent inputting data	£257 (SD = £423)	
Total cost of inputting data online	£30,130 (SD = £64,434)	
Cost per site	£198 (SD = £422)	

Table 2: Breakdown of costs of collecting audit data for Trial 2

	**Value**	**Notes**
<u>Time and cost of completing audit booklets (Trial 2)</u>		
Mean time take to complete booklet	27 minutes (SD = 31)	Median time was 19 minutes (IQR 11;27)
Weighted average salary for NHS staff	£39,748 per annum £0.32 per minute	Based on description of 163 staff who completed booklet
Number of patients audited in T2R1	4098	
Total cost of time spent completing audit booklets T2R1	£35,240 (SD = £40,461)	

© 2022 Stanworth SJ et al. *JAMA Network Open*.

Number of patients audited in T2R2	4098	
Total cost of time spent completing audit booklets T2R2	£35,240 (SD = £40,461)	
Total cost of both audit rounds	£70,481 (SD = £80,922)	
Cost per site	£464 (SD = £532)	

<u>*Time and cost of inputting audit booklet data online (Trial 2)*</u>

Mean time taken to enter data online	25.7 minutes for NHS staff 6.8 minutes for NHSBT staff	No data available for Trial 2. Therefore Trial 1 values used
Number of NHS entries	4692	
Cost to NHS of inputting data	£38,465 (SD = £82,864)	
Number of NHSBT entries	744	These are audit booklets returned to NHSBT with data that need inputting online. Postage costs not included.
Cost to NHSBT of inputting data	£1,560 (SD = £814)	
Total	£40,026 (SD = 83,678)	
Cost per site	£263 (SD = 551)	

© 2022 Stanworth SJ et al. *JAMA Network Open*.

Table 3: cost breakdown for developing and delivering the Standard report

	Value	Notes
Writing Group costs		
Writing Groups meetings	£415	800 cumulative minutes. 8 members of group (not all in attendance at every meeting).
Additional work done by writing group members	£3,758	Est. 12 hours per member for 5 paid members of writing group.
Additional work done by audit lead	£1,148	Est. 50 hours (From consultation with AFFINITIE team)
Production Costs		
NHSBT Programme director time	£2,295	Est 82 hours (Time working on audit feedback for 2 rounds)
NHSBT analyst time	£5,356	0.5 FTE, 1 year
Building audit on NHSBT system	£2,800	For 2 audit rounds
Total	£15,773	
Cost per site	£104	(76 sites)

© 2022 Stanworth SJ et al. *JAMA Network Open*.

Table 4: cost breakdown for developing and delivering the Enhanced report

	Value	Notes
Enhanced Writing Group costs		
Training writers of audit report	£1,274	4-6 members per meeting. 25 cumulative hours
Cost of time spent at writing groups meetings	£3026	11 members of group (not all in attendance at every meeting). 146 cumulative hours
Additional work done by writing group members	£2,565	For 7 paid members of writing group. Est. 12 hours per member
Additional work done by audit lead	£2,213	Est. 50 hours (From consultation with AFFINITIE team)
Cost of time of AFFINITIE Researcher	£18,512	1 years at 0.4 FTE, not including time in training and meetings.
Production Costs		
NHSBT Programme director time	£2,295	Est 82 hours Time working on audit feedback
NHSBT analyst time	£5,356	0.5 FTE, 1 year
Building audit on NHSBT system	£2,800	For 2 audit rounds
Graphic design consultant time	£4000	
AFFINITIE statistician	£14,980	.25 FTE medical statistician for 16months. (Does not include time at meetings)
Algorithm development	£2,615	
Total	£59,637	
Cost per site	£392	(76 sites)

© 2022 Stanworth SJ et al. *JAMA Network Open*.

Table 5: cost breakdown for the Telephone Support Service

	Value	Notes
TSS shifts (Trial 1)		
Total time on shifts	£2364	123 hours of shifts by 3 different researchers, including 24 cumulative hours of training sessions
Call logs (Trial 1)		
NHS staff time on phone	£371 (SD = £268)	1,264 minutes (SD = 912)
Phone line cost	£12.60	0.01p per minute (sourced from City University who provided the service)
Total	£2,747 (SD = £2,644)	
Cost per site	£36 (SD = £35)	
TSS shifts (Trial 2)		
Total time on shifts	£2,951	157 hours of shifts by 3 different researchers, including 24 cumulative hours of training sessions
Call logs (Trial 2)		
NHS staff time on phone	£289 (SD = 221)	986 minutes (SD = 752)
Phone line cost	£9.86	0.01p per minute
Total	£3,273 (SD = £3,179)	
Cost per site	£43 (SD = £42)	

**All costs rounded to nearest whole number (where appropriate)*

© 2022 Stanworth SJ et al. *JAMA Network Open*.

Table 6: cost breakdown for the Online Toolkit

	Value	Notes
Development costs		
Lead Researcher at 0.4 FTE for 2 years	£37,539	
Computer Science staff	£70,238	
Server hire (annual)	£667	
Total Development cost	£108,444	
running cost	£667	server hire cost (annual)
Running cost per site	£4.40	For each of Trials 1 and 2
NHS staff usage – Trial 1	**Mean (SD)**	
Standard arm average duration of use	116 minutes (159)	2670 minutes logged on across 23 sites
cost per site	£14.60 (£18.50)	including running costs
Enhanced arm average duration of use	35 minutes (£51)	909 minutes logged on across 26 sites
Cost per site	£7.90 (£9.50)	including running costs
NHS staff usage – Trial 2		
Standard arm average duration of use	10 minutes (14)	220 minutes logged on across 24 sites
cost per site	£5.30 (£5.70)	including running costs
Enhanced arm average duration of use	19 minutes (26)	233 minutes logged on across 15 sites
Cost per site	£5.50 (£5.90)	including running costs

© 2022 Stanworth SJ et al. *JAMA Network Open*.

Table 7: cost breakdown for additional NHS staff activity – Trial 1

	**Mean (SE)**	**Hours spent (SE)**
Cost of time Nurse TP spent on planning their response to audit report (per site)		
Standard content	£144.56 (£30.36)	8.2 hours (1.7)
Enhanced content	£166.32 (£35.45)	9.4 hours (2.0)
Standard FoS	£132.70 (£30.36)	7.5 hours (1.7)
Enhanced FoS	£178.98 (£35.45)	10.2 hours (2.0)
Cost of time at meetings the report was discussed at (per site)		
Standard content	£1091.47 (£174.43)	3.8 hours (0.61)
Enhanced content	£1051.13 (£173.86)	3.7 hours (0.61)
Standard FoS	£1101.41 (£175.85)	3.9 hours (0.62)
Enhanced FoS	£1040.62 (£172.16)	3.7 hours (0.61)

SE = standard error of the mean

© 2022 Stanworth SJ et al. *JAMA Network Open*.

Table 8: cost breakdown for additional NHS staff activity – Trial 2

	Mean (SE)	Hours spent (SE)
Cost of time Nurse TP spent on planning their response to audit report (per site)		
Standard content	£100.10 (£20.05)	5.7 (1.1)
Enhanced content	£89.77 (£18.15)	5.1 (1.0)
Standard FoS	£109.24 (£22.94)	6.2 (1.3)
Enhanced FoS	£83.17 (£15.95)	4.7 (0.9)
Cost of time at meetings the report was discussed at (per site)		
Standard content	£819.88 (£109.09)	2.9 (0.38)
Enhanced content	£852.27 (£114.49)	3.0 (0.4)
Standard FoS	£956.90 (£134.66)	3.4 (0.47)
Enhanced FoS	£726.98 (£92.90)	2.6 (0.33)

SE = standard error of the mean

© 2022 Stanworth SJ et al. *JAMA Network Open*.

Table 9: cost breakdown for volume of blood transfused

	Cost for all sites	SD		Mean per site	SD
Trial 1					
Standard Content	£90,961,967	£65,350,799		£492,619	£353,711
Enhanced Content	£116,022,150	£83,362,731		£628,189	£451,178
Standard FoS	£93,619,300	£63,271,664		£506,982	£342,474
Enhanced FoS	£103,757,875	£79,280,997		£561,808	£429,036
Trial 2					
Standard Content	£130,914,365	£102,720,804		£675,173	£529,887
Enhanced Content	£133,779,463	£110,394,066		£689,873	£569,421
Standard FoS	£112,903,993	£88,467,971		£582,805	£456,528
Enhanced FoS	£119,570,002	£88,812,870		£617,084	£458,331

© 2022 Stanworth SJ et al. *JAMA Network Open*.

Table 10: Trial 1 outcome parameters. Percentage of transfusions acceptable (primary outcome), volume of blood transfused (secondary outcome), and number of SHOT events (secondary outcome).

	Percentage of transfusions acceptable	Volume of blood transfused per arm	Mean per site	Number of SHOT events	Mean per site
Standard content	18.4	516,499 (SD = 371,291)	2,792 (SD = 2,007)	1193 (SD = 1441)	6.5 (SD = 7.8)
Enhanced content	17.6	658,951 (SD = 473,650)	3,562 (SD = 2,560)	1159 (SD = 967)	6.3 (SD =) 5.2
Standard FoS	18.1	531,618 (SD = 359,462)	2,874 (SD = 1,943)	1059 (SD = 938)	5.7 (SD = 5.1)
Enhanced FoS	18.0	589,301 (SD = 450,541)	3,185 (SD = 2,435)	1293 (SD = 1466)	7.0 (SD = 7.9)

Table 11: Trial 2 outcome parameters. Percentage of transfusions acceptable (primary outcome), volume of blood transfused (secondary outcome), and number of SHOT events (secondary outcome).

	Percentage of transfusions acceptable	Volume of RBC transfused	Mean RBC per site	Volume of platelets transfused	Mean platelets per site	Number of SHOT events	Mean per site
Standard content	74.4	625,753 (SD = 437,078)	3,226 (SD = 2,253)	90,122 (SD = 112,250)	465 (SD = 579)	1355 (SD = 1674)	7.0 (SD = 8.6)
Enhanced content	71.4	635,726 (SD = 456,894)	3,277 (SD = 2,355)	94,699 (SD = 130,297)	488 (SD = 672)	1285 (SD = 1061)	6.6 (SD = 5.5)
Standard FoS	73.9	584,232 (SD = 423,981)	3,012 (SD = 2,185)	43,714 (SD = 60,247)	225 (SD = 311)	1256 (SD = 914)	6.5 (SD = 4.7)
Enhanced FoS	72.1	618,725 (SD = 428,452)	3,189 (SD = 2,209)	46,438 (SD = 58,331)	239 (SD = 301)	1384 (SD = 1750)	7.1 (SD = 9.0)

© 2022 Stanworth SJ et al. *JAMA Network Open*.

Table 12: Standard vs Enhanced Content analyses, ICER

For the Standard vs Enhanced Content analyses, the ICER can be given as:

**Standard content audit report (Control)**	**Enhanced content audit report (Intervention)**
**Costs$_{\text{(Standard Content)}}$ =**<br>Cost of collecting audit data and delivering standard audit report<br>+<br>Cost of activity sites conducted as a result of receiving audit report<br>+<br>Cost of blood units transfused	**Costs$_{\text{(Enhanced Content)}}$ =**<br>Cost of collecting audit data and delivering enhanced audit report<br>+<br>Cost of activity sites conducted as a result of receiving audit report<br>+<br>Cost of blood units transfused
**Benefits$_{\text{(Standard Content)}}$ =**<br>The percentage of transfusions given that were acceptable	**Benefits$_{\text{(Enhanced Content)}}$ =**<br>The percentage of transfusions given that were acceptable
**Incremental Cost-Effectiveness Ratio =**<br>**(Costs$_{\text{(Enhanced Content)}}$ - Costs$_{\text{(Standard Content)}}$) / (Benefits$_{\text{(Enhanced Content)}}$ - Benefits$_{\text{(Standard Content)}}$)**	

© 2022 Stanworth SJ et al. *JAMA Network Open*.

Table 13: Standard vs Enhanced Follow-on Support analyses, ICER

For the Standard vs Enhanced Follow-on Support (FoS) analyses, the ICER can be given as:

**Standard FoS (Control)**	**Enhanced FoS (Intervention)**
**Costs**$_{\textbf{(Standard FoS)}}$ =<br>Cost of collecting audit data and delivering standard audit report<br>+<br>Cost of delivering standard FoS<br>+<br>Cost of activity sites conducted as a result of receiving audit report<br>+<br>Cost of blood units transfused	**Costs**$_{\textbf{(Enhanced FoS)}}$ =<br>Cost of collecting audit data and delivering standard audit report<br>+<br>Cost of delivering enhanced FoS<br>+<br>Cost of activity sites conducted as a result of receiving audit report<br>+<br>Cost of blood units transfused
**Benefits**$_{\textbf{(Standard FoS)}}$ =<br>The percentage of transfusions given that were acceptable	**Benefits**$_{\textbf{(Enhanced FoS)}}$ =<br>The percentage of transfusions given that were acceptable
**Incremental Cost-Effectiveness Ratio =**<br>**(Costs**$_{\textbf{(Enhanced FoS)}}$ **- Costs**$_{\textbf{(Standard FoS)}}$ **) / (Benefits**$_{\textbf{(Enhanced FoS)}}$ **- Benefits**$_{\textbf{(Standard FoS)}}$ **)**	

© 2022 Stanworth SJ et al. *JAMA Network Open*.

Table 14: cost components of the interventions for Trial 1

Trial 1

	Standard Content		Enhanced Content		Difference (i.e. Additional cost of Enhanced)	
	**Cost for all 185 sites**	**Cost per site**	**Cost for all 185 sites**	**Cost per site**	**All sites**	**Per site**
Audit data collection	£93,841	£507	£93,841	£507	£0	£0
Feedback interventions	£22,739	£123	£66,604	£360	£43,864	£237
Additional NHS activity	£228,666	£1,236	£225,228	£1,217	-£3,438	-£19
Blood transfused	£90,789,344	£490,753	£115,829,314	£626,104	£25,039,970	£135,351
Total	£91,134,590	£492,619	£116,214,986	£628,165	£25,080,396	£135,570

	Standard Follow-on Support		Enhanced Follow-on Support		Difference (i.e. Additional cost of Enhanced)	
	**Cost for all 185 sites**	**Cost per site**	**Cost for all 185 sites**	**All sites**	**All sites**	**Per site**
Audit data collection	£93,841	£507	£93,841	£507	£0	£0
Feedback interventions	£22,739	£123	£28,721	£155	£5,982	£32
Additional NHS activity	£228,311	£1,234	£225,625	£1,220	-£2,686	-£15
Blood transfused	£93,446,854	£505,118	£103,586,378	£559,926	£10,139,524	£54,808
Total	£93,791,745	£506,982	£103,934,565	£561,808	£10,142,820	£54,826

© 2022 Stanworth SJ et al. *JAMA Network Open*.

Table 15: cost components of the interventions for Trial 2

Trial 2	Standard Content		Enhanced Content		Difference (i.e. Additional cost of Enhanced)	
	**Cost for all 194 sites**	**Cost per site**	**Cost for all 194 sites**	**All sites**	**All sites**	**Per site**
Audit data collection	£110,507	£570	£110,507	£570	£0	£0
Feedback interventions	£17,069	£88	£60,933	£314	£43,864	£226
Additional NHS activity	£178,476	£920	£182,755	£942	£4,278	£22
Blood transfused	£130,677,492	£673,595	£133,481,117	£688,047	£2,803,625	£14,452
Total	£130,983,544	£675,173	£133,835,311	£689,873	£2,851,768	£14,700
	**Standard follow-on support**		**Enhanced follow-on support**		**Difference (i.e. Additional cost of Enhanced)**	
	**Cost for all 194 sites**	**Cost per site**	**Cost for all 194 sites**	**Cost per site**	**All sites**	**Per site**
Audit data collection	£110,507	£570	£110,507	£570	£0	£0
Feedback interventions	£17,069	£88	£30,141	£155	£13,073	£67
Additional NHS activity	£208,577	£1,075	£157,169	£810	-£51,407	-£265
Blood transfused	£130,677,492	£673,595	£119,416,399	£615,548	-£11,261,092	-£58,047
Total	£113,064,213	£582,805	£119,714,217	£617,084	£6,650,004	£34,278

© 2022 Stanworth SJ et al. *JAMA Network Open*.

Table 16: Breakdown of calculation for cost-neutral analysis

**Trial 1**	**Cost per site**	**units of RBC equivalent needed to be cost-neutral**
Enhanced vs Standard Content		
Incremental mean	£219	1.2
Incremental Standard Deviation	£210	1.2
95%UI upper bound	£630	3.6
95%UI lower bound	-£193	0
Enhanced vs Standard Follow-on Support		
Incremental mean	£18	0.1
Incremental Standard Deviation	£24	0.1
95%UI upper bound	£66	0.4
95%UI lower bound	-£30	0

© 2022 Stanworth SJ et al. *JAMA Network Open*.

Table 17: Breakdown of calculation for cost-neutral analysis

Trial 2	Cost per site	units of RBC equivalent
Enhanced vs Standard Content		
Incremental mean	£248	1.4
Incremental Standard Deviation	£230	1.3
95%UI upper bound	£698	4.0
95%UI lower bound	-£202	0
Enhanced vs Standard Follow-on Support		
Incremental mean	-£198	0
Incremental Standard Deviation	£19	0.1
95%UI upper bound	-£161	0
95%UI lower bound	-£234	0

© 2022 Stanworth SJ et al. *JAMA Network Open*.

Figure 2 shows the simulated outputs shown on a cost-effectiveness plane (CEP). Figure 3 shows the probability of the enhanced intervention being cost effective at increasing WTP thresholds. This suggests a 55.3% chance that the enhanced intervention is cost-effective at a WTP threshold of £1,000 per percentage increase in acceptable transfusions.

Percentage of transfusions acceptable (Enhanced vs Standard Follow-on Support)

The PSA results suggest that the mean incremental cost of the enhanced vs standard intervention was £64,319 per site (95% UI, -£1,003,034 to £1,131,673). The mean incremental change in percentage of acceptable transfusions was -0.11 (95% UI, -2.38 to 2.16). Therefore, the enhanced intervention was more costly and less effective than the standard. Figure 4 shows the simulated outputs on a CEP. Figure 5 shows the CEAC, suggesting a 50.3% chance that the enhanced intervention is cost-effective at a WTP threshold of £1,000.

Volume of blood transfused (Enhanced vs Standard Content)

The PSA results suggest that the mean incremental cost of the Enhanced vs Standard intervention was £122,911 per site (95% UI, -£1,033,345 to £1,279,168). The mean incremental change in volume of blood transfused was 698 (95% UI, -5,409 to 6,806). This indicates that the Enhanced intervention was more costly and less effective at reducing the volume of blood transfused than the Standard, but there is considerable uncertainty in these estimates, indicated by the wide confidence intervals that include zero. Figure 6 shows the simulated outputs are shown on a cost-effectiveness plan (CEP) below.

Figure 7 shows the probability of the Enhanced intervention being cost effective at increasing willingness-to-pay (WTP) thresholds, also known as the cost-effectiveness acceptability curve (CEAC). The PSA suggests there is a 94% chance that the Enhanced Intervention is cost-effective at a WTP threshold of £1,000 per unit reduction in the volume of blood transfused.

Volume of blood transfused (Enhanced vs Standard Follow-on Support)

The PSA results suggest that the mean incremental cost of the Enhanced vs Standard intervention was £92,536 per site (95% UI, -£1,000,577 to £1,185,649). The mean incremental change in volume of blood transfused was 518 (95% UI, -6,989 to 5,953). This indicates that the Enhanced intervention was more costly and less effective at reducing the volume of blood transfused than the Standard, but there is considerable uncertainty in these estimates, indicated by the wide confidence intervals that include zero. Figure 8 shows the simulated outputs are shown on a cost-effectiveness plan (CEP) below.

Figure 9 shows the probability of the Enhanced intervention being cost effective at increasing willingness-to-pay (WTP) thresholds, also known as the cost-effectiveness acceptability curve (CEAC).

© 2022 Stanworth SJ et al. *JAMA Network Open*.

The PSA suggests there is a 98.9% chance that the Enhanced Intervention is cost-effective at a WTP threshold of £1,000 per unit reduction in the volume of blood transfused.

Number of SHOT events (Enhanced vs Standard Content)

The PSA results suggest that the mean incremental cost of the Enhanced vs Standard intervention was £151,769 per site (95% UI, -£,991,730 to £1,296,268). The mean incremental change in SHOT events was -0.19 (95% UI, -17.5 to 17.9). This indicates that the Enhanced intervention was more costly and more effective at reducing the number of SHOT events than the Standard, but there is considerable uncertainty in these estimates, indicated by the wide confidence intervals that include zero. Figure 10 shows the simulated outputs are shown on a cost-effectiveness plan (CEP) below.

Figure 11 shows the probability of the Enhanced intervention being cost effective at increasing willingness-to-pay (WTP) thresholds, also known as the cost-effectiveness acceptability curve (CEAC). The PSA suggests there is a 49.5% chance that the Enhanced Intervention is cost-effective at a WTP threshold of £1,000 per 1-unit reduction in the number of SHOT-reportable events.

Trial 1 – Number of SHOT events (Enhanced vs Standard Follow-on Support)

The PSA results suggest that the mean incremental cost of the Enhanced vs Standard intervention was £35,469 per site (95% UI, -£1,030,945 to £1,101,883). The mean incremental change in SHOT events was -0.79 (95% UI, -17.7 to 16.2). This indicates that the Enhanced intervention was more costly and less effective at reducing the number of SHOT events than the Standard, but there is considerable uncertainty in these estimates, indicated by the wide confidence intervals that include zero. Figure 12 shows the simulated outputs are shown on a cost-effectiveness plan (CEP) below.

Figure 13 shows the probability of the Enhanced intervention being cost effective at increasing willingness-to-pay (WTP) thresholds, also known as the cost-effectiveness acceptability curve (CEAC). The PSA suggests there is a 50.5% chance that the Enhanced Intervention is cost-effective at a WTP threshold of £1,000 per 1-unit reduction in the number of SHOT-reportable events.

Sensitivity Analysis Results – Trial 2

Percentage of transfusions acceptable (Enhanced vs Standard Content)

The PSA results suggest that the mean incremental cost of the enhanced vs standard intervention was £23,869 per site (95% UI, -£851,107 to £898,846). The mean incremental change in percentage of acceptable transfusions was -3.04 (95% UI, -5.04 to -1.03). Therefore, the enhanced intervention was more costly and less effective than the standard. Figure 14 shows the simulated outputs are shown on a CEP. Figure 15 shows the CEAC, suggesting a 43.1% chance that the enhanced intervention is cost-effective at a WTP threshold of £1,000.

© 2022 Stanworth SJ et al. *JAMA Network Open*.

Percentage of transfusions acceptable (Enhanced vs Standard Follow-on Support)

The PSA results suggest that the mean incremental cost of the enhanced vs standard intervention was £36,201 per site (95% UI, -£1,045,641 to £1,118,042). The mean incremental change in percentage of acceptable transfusions was -1.8 (95% UI, -3.8 to 0.2). Therefore, the enhanced intervention was more costly and less effective than the standard. Figure 16 shows the simulated outputs on a CEP. Figure 17 shows the CEAC, suggesting a 53.8% chance that the enhanced intervention is cost-effective at a WTP threshold of £1,000.

Volume of blood transfused (Enhanced vs Standard Content)

The PSA results suggest that the mean incremental cost of the Enhanced vs Standard intervention was £12,633 per site (95% UI, -£862,404 to £887,669). The mean incremental change in volume of blood transfused was 28.5 (95% UI, -6,923 to 6,866). This indicates that the Enhanced intervention was more costly and less effective at reducing the volume of blood transfused than the Standard, but there is considerable uncertainty in these estimates, indicated by the wide confidence intervals that include zero. Figure 18 shows the simulated outputs are shown on a cost-effectiveness plan (CEP) below.

Figure 19 shows the probability of the Enhanced intervention being cost effective at increasing willingness-to-pay (WTP) thresholds, also known as the cost-effectiveness acceptability curve (CEAC). The PSA suggests there is a 95.6% chance that the Enhanced Intervention is cost-effective at a WTP threshold of £1,000 per unit reduction in the volume of blood transfused.

Trial 2 – Volume of blood transfused (Enhanced vs Standard Follow-on Support)

The PSA results suggest that the mean incremental cost of the Enhanced vs Standard intervention was £2,690 per site (95% UI, -£1,092,638 to £1,087,257). The mean incremental change in volume of blood transfused was 204 (95% UI, -6,215 to 5,807). This indicates that the Enhanced intervention was more costly and less effective at reducing the volume of blood transfused than the Standard, but there is considerable uncertainty in these estimates, indicated by the wide confidence intervals that include zero. Figure 20 shows the simulated outputs are shown on a cost-effectiveness plan (CEP) below.

Figure 21 shows the probability of the Enhanced intervention being cost effective at increasing willingness-to-pay (WTP) thresholds, also known as the cost-effectiveness acceptability curve (CEAC). The PSA suggests there is a 93.6% chance that the Enhanced Intervention is cost-effective at a WTP threshold of £1,000 per unit reduction in the volume of blood transfused.

© 2022 Stanworth SJ et al. *JAMA Network Open*.

Trial 2 – Number of SHOT events (Enhanced vs Standard Content)

The PSA results suggest that the mean incremental cost of the Enhanced vs Standard intervention was £471 per site (95% UI, -£833,316 to £834,257). The mean incremental decrease in SHOT events was 0.35 (95% UI, -19.8 to 20.5). This indicates that the Enhanced intervention was more costly and more effective at reducing the number of SHOT events than the Standard, but there is considerable uncertainty in these estimates, indicated by the wide confidence intervals that include zero. Figure 22 shows the simulated outputs are shown on a cost-effectiveness plan (CEP) below.

Figure 23 shows the probability of the Enhanced intervention being cost effective at increasing willingness-to-pay (WTP) thresholds, also known as the cost-effectiveness acceptability curve (CEAC). The PSA suggests there is a 49.2% chance that the Enhanced Intervention is cost-effective at a WTP threshold of £1,000 per 1-unit reduction in the number of SHOT-reportable events.

Trial 2 – Number of SHOT events (Enhanced vs Standard Follow-on Support)

The PSA results suggest that the mean incremental cost of the Enhanced vs Standard intervention was £37,691 per site (95% UI, -£1,030,523 to £1,105,905). The mean incremental increase in SHOT events was 0.5 (95% UI, -19.9 to 19.0). This indicates that the Enhanced intervention was more costly and less effective at reducing the number of SHOT events than the Standard, but there is considerable uncertainty in these estimates, indicated by the wide confidence intervals that include zero. Figure 24 shows the simulated outputs are shown on a cost-effectiveness plan (CEP) below.

Figure 25 shows the probability of the Enhanced intervention being cost effective at increasing willingness-to-pay (WTP) thresholds, also known as the cost-effectiveness acceptability curve (CEAC). The PSA suggests there is a 50.7% chance that the Enhanced Intervention is cost-effective at a WTP threshold of £1,000 per 1-unit reduction in the number of SHOT-reportable events.

© 2022 Stanworth SJ et al. *JAMA Network Open*.

Supplementary Figures and Tables

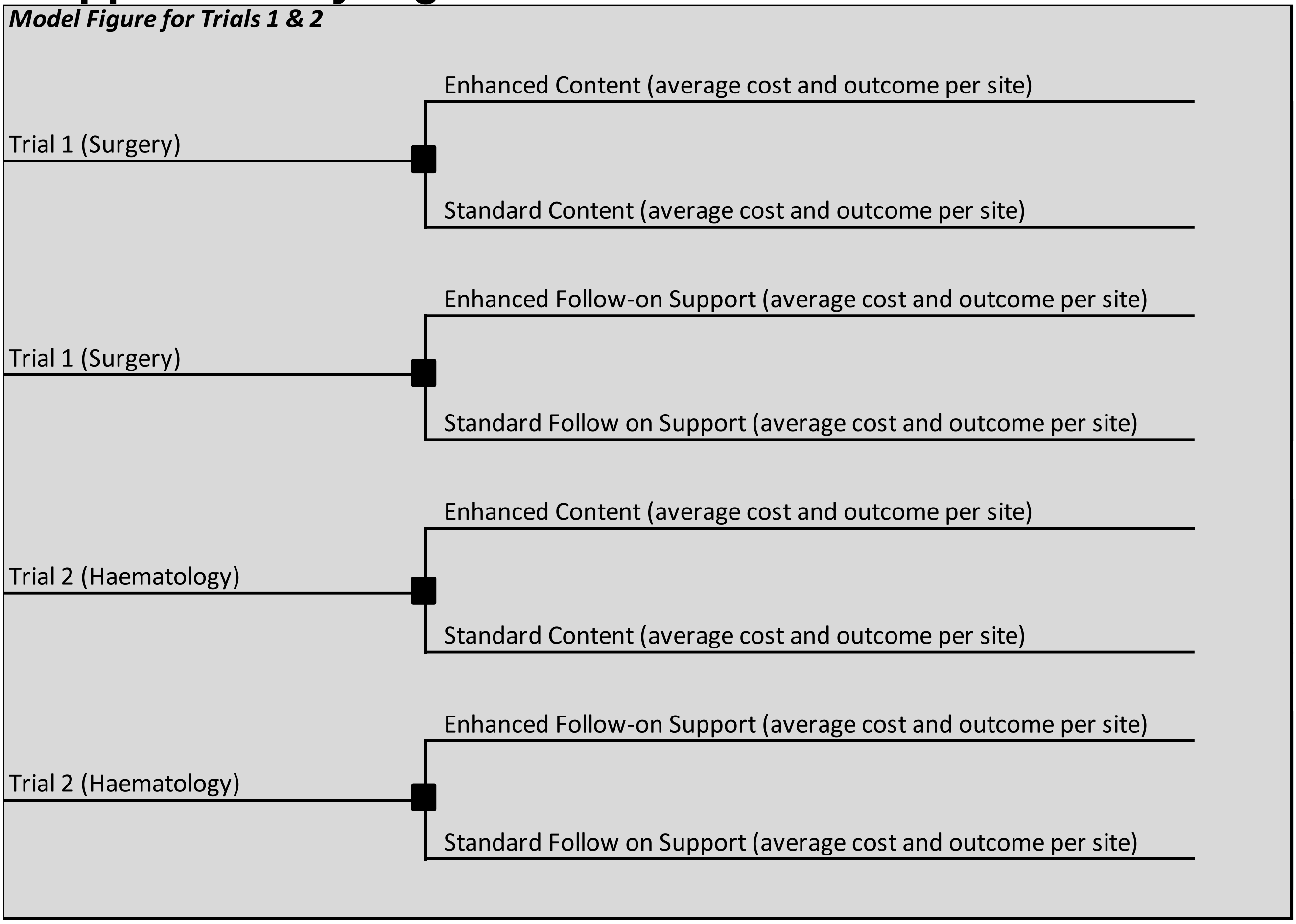

Figure 1: Cost-effectiveness analysis model for Trials 1 and 2

© 2022 Stanworth SJ et al. *JAMA Network Open*.

Table 1: Breakdown of costs of collecting audit data for Trial 1

	Value	Notes
Time and cost of completing audit booklets (Trial 1)		
Mean time take to complete audit booklets	28.4 minutes (SD = 55.5)	Median time was 27 minutes (IQR 18;37)
Weighted average salary for NHS staff	£38,387 per annum<br>£0.31 per minute	Based on description of 217 staff who completed booklet
*Number of patients audited in T1R1**	4035	
Total cost of time spent completing audit booklets T1R1	£35,211 (SD = £24,450)	
Number of patients audited in T1R2	3266	
Total cost of time spent completing audit booklets T1R2	£28,500 (SD = £19,790)	
Total cost of both audit rounds	£63,711 (SD = £44,240)	
Cost per site	£418 (SD = £290)	
Time and cost of inputting audit booklet data online (Trial 1)		
Mean time taken to enter data online	25.7 mins for NHS staff (SD = 55.5)<br>6.8 minutes for NHSBT staff (SD = 3.5)	Median time for NHS and NHSBT was 10 and 6 minutes respectively
Weighted average salary for NHSBT staff	£38,726 per annum	Based on records of staff tasked with inputting data

© 2022 Stanworth SJ et al. *JAMA Network Open*.

	£0.31 per minute	
Number of NHS entries	3769	These are system logs of data being entered, therefore not necessarily 1 entry per booklet
Cost to NHS of time spent inputting data	£29,840 (SD = £64,283)	
Number of NHSBT entries	138	These are audit booklets returned to NHSBT with data that need inputting online. Postage costs not included.
Cost to NHSBT of time spent inputting data	£257 (SD = £423)	
Total cost of inputting data online	£30,130 (SD = £64,434)	
Cost per site	£198 (SD = £422)	

Table 2: Breakdown of costs of collecting audit data for Trial 2

	**Value**	**Notes**
Time and cost of completing audit booklets (Trial 2)		
Mean time take to complete booklet	27 minutes (SD = 31)	Median time was 19 minutes (IQR 11;27)
Weighted average salary for NHS staff	£39,748 per annum £0.32 per minute	Based on description of 163 staff who completed booklet
Number of patients audited in T2R1	4098	
Total cost of time spent completing audit booklets T2R1	£35,240 (SD = £40,461)	

© 2022 Stanworth SJ et al. *JAMA Network Open*.

Number of patients audited in T2R2	4098	
Total cost of time spent completing audit booklets T2R2	£35,240 (SD = £40,461)	
Total cost of both audit rounds	£70,481 (SD = £80,922)	
Cost per site	£464 (SD = £532)	

<u>*Time and cost of inputting audit booklet data online (Trial 2)*</u>

Mean time taken to enter data online	25.7 minutes for NHS staff 6.8 minutes for NHSBT staff	No data available for Trial 2. Therefore Trial 1 values used
Number of NHS entries	4692	
Cost to NHS of inputting data	£38,465 (SD = £82,864)	
Number of NHSBT entries	744	These are audit booklets returned to NHSBT with data that need inputting online. Postage costs not included.
Cost to NHSBT of inputting data	£1,560 (SD = £814)	
Total	£40,026 (SD = 83,678)	
Cost per site	£263 (SD = 551)	

© 2022 Stanworth SJ et al. *JAMA Network Open*.

Table 3: cost breakdown for developing and delivering the Standard report

	Value	Notes
Writing Group costs		
Writing Groups meetings	£415	800 cumulative minutes. 8 members of group (not all in attendance at every meeting).
Additional work done by writing group members	£3,758	Est. 12 hours per member for 5 paid members of writing group.
Additional work done by audit lead	£1,148	Est. 50 hours (From consultation with AFFINITIE team)
Production Costs		
NHSBT Programme director time	£2,295	Est 82 hours (Time working on audit feedback for 2 rounds)
NHSBT analyst time	£5,356	0.5 FTE, 1 year
Building audit on NHSBT system	£2,800	For 2 audit rounds
Total	£15,773	
Cost per site	£104	(76 sites)

© 2022 Stanworth SJ et al. *JAMA Network Open*.

Table 4: cost breakdown for developing and delivering the Enhanced report

	Value	Notes
Enhanced Writing Group costs		
Training writers of audit report	£1,274	4-6 members per meeting. 25 cumulative hours
Cost of time spent at writing groups meetings	£3026	11 members of group (not all in attendance at every meeting). 146 cumulative hours
Additional work done by writing group members	£2,565	For 7 paid members of writing group. Est. 12 hours per member
Additional work done by audit lead	£2,213	Est. 50 hours (From consultation with AFFINITIE team)
Cost of time of AFFINITIE Researcher	£18,512	1 years at 0.4 FTE, not including time in training and meetings.
Production Costs		
NHSBT Programme director time	£2,295	Est 82 hours Time working on audit feedback
NHSBT analyst time	£5,356	0.5 FTE, 1 year
Building audit on NHSBT system	£2,800	For 2 audit rounds
Graphic design consultant time	£4000	
AFFINITIE statistician	£14,980	.25 FTE medical statistician for 16months. (Does not include time at meetings)
Algorithm development	£2,615	
Total	£59,637	
Cost per site	£392	(76 sites)

© 2022 Stanworth SJ et al. *JAMA Network Open*.

Table 5: cost breakdown for the Telephone Support Service

	Value	Notes
TSS shifts (Trial 1)		
Total time on shifts	£2364	123 hours of shifts by 3 different researchers, including 24 cumulative hours of training sessions
Call logs (Trial 1)		
NHS staff time on phone	£371 (SD = £268)	1,264 minutes (SD = 912)
Phone line cost	£12.60	0.01p per minute (sourced from City University who provided the service)
Total	£2,747 (SD = £2,644)	
Cost per site	£36 (SD = £35)	
TSS shifts (Trial 2)		
Total time on shifts	£2,951	157 hours of shifts by 3 different researchers, including 24 cumulative hours of training sessions
Call logs (Trial 2)		
NHS staff time on phone	£289 (SD = 221)	986 minutes (SD = 752)
Phone line cost	£9.86	0.01p per minute
Total	£3,273 (SD = £3,179)	
Cost per site	£43 (SD = £42)	

**All costs rounded to nearest whole number (where appropriate)*

© 2022 Stanworth SJ et al. *JAMA Network Open*.

Table 6: cost breakdown for the Online Toolkit

	Value	Notes
Development costs		
Lead Researcher at 0.4 FTE for 2 years	£37,539	
Computer Science staff	£70,238	
Server hire (annual)	£667	
Total Development cost	£108,444	
running cost	£667	server hire cost (annual)
Running cost per site	£4.40	For each of Trials 1 and 2
NHS staff usage – Trial 1	**Mean (SD)**	
Standard arm average duration of use	116 minutes (159)	2670 minutes logged on across 23 sites
cost per site	£14.60 (£18.50)	including running costs
Enhanced arm average duration of use	35 minutes (£51)	909 minutes logged on across 26 sites
Cost per site	£7.90 (£9.50)	including running costs
NHS staff usage – Trial 2		
Standard arm average duration of use	10 minutes (14)	220 minutes logged on across 24 sites
cost per site	£5.30 (£5.70)	including running costs
Enhanced arm average duration of use	19 minutes (26)	233 minutes logged on across 15 sites
Cost per site	£5.50 (£5.90)	including running costs

© 2022 Stanworth SJ et al. *JAMA Network Open*.

Table 7: cost breakdown for additional NHS staff activity – Trial 1

	Mean (SE)	Hours spent (SE)
Cost of time Nurse TP spent on planning their response to audit report (per site)		
Standard content	£144.56 (£30.36)	8.2 hours (1.7)
Enhanced content	£166.32 (£35.45)	9.4 hours (2.0)
Standard FoS	£132.70 (£30.36)	7.5 hours (1.7)
Enhanced FoS	£178.98 (£35.45)	10.2 hours (2.0)
Cost of time at meetings the report was discussed at (per site)		
Standard content	£1091.47 (£174.43)	3.8 hours (0.61)
Enhanced content	£1051.13 (£173.86)	3.7 hours (0.61)
Standard FoS	£1101.41 (£175.85)	3.9 hours (0.62)
Enhanced FoS	£1040.62 (£172.16)	3.7 hours (0.61)

SE = standard error of the mean

© 2022 Stanworth SJ et al. *JAMA Network Open*.

Table 8: cost breakdown for additional NHS staff activity – Trial 2

	Mean (SE)	Hours spent (SE)
Cost of time Nurse TP spent on planning their response to audit report (per site)		
Standard content	£100.10 (£20.05)	5.7 (1.1)
Enhanced content	£89.77 (£18.15)	5.1 (1.0)
Standard FoS	£109.24 (£22.94)	6.2 (1.3)
Enhanced FoS	£83.17 (£15.95)	4.7 (0.9)
Cost of time at meetings the report was discussed at (per site)		
Standard content	£819.88 (£109.09)	2.9 (0.38)
Enhanced content	£852.27 (£114.49)	3.0 (0.4)
Standard FoS	£956.90 (£134.66)	3.4 (0.47)
Enhanced FoS	£726.98 (£92.90)	2.6 (0.33)

SE = standard error of the mean

© 2022 Stanworth SJ et al. *JAMA Network Open*.

Table 9: cost breakdown for volume of blood transfused

	Cost for all sites	SD	Mean per site	SD
Trial 1				
Standard Content	£90,961,967	£65,350,799	£492,619	£353,711
Enhanced Content	£116,022,150	£83,362,731	£628,189	£451,178
Standard FoS	£93,619,300	£63,271,664	£506,982	£342,474
Enhanced FoS	£103,757,875	£79,280,997	£561,808	£429,036
Trial 2				
Standard Content	£130,914,365	£102,720,804	£675,173	£529,887
Enhanced Content	£133,779,463	£110,394,066	£689,873	£569,421
Standard FoS	£112,903,993	£88,467,971	£582,805	£456,528
Enhanced FoS	£119,570,002	£88,812,870	£617,084	£458,331

© 2022 Stanworth SJ et al. *JAMA Network Open*.

Table 10: Trial 1 outcome parameters. Percentage of transfusions acceptable (primary outcome), volume of blood transfused (secondary outcome), and number of SHOT events (secondary outcome).

	**Percentage of transfusions acceptable**	**Volume of blood transfused per arm**	**Mean per site**	**Number of SHOT events**	**Mean per site**
Standard content	18.4	516,499 (SD = 371,291)	2,792 (SD = 2,007)	1193 (SD = 1441)	6.5 (SD = 7.8)
Enhanced content	17.6	658,951 (SD = 473,650)	3,562 (SD = 2,560)	1159 (SD = 967)	6.3 (SD =) 5.2
Standard FoS	18.1	531,618 (SD = 359,462)	2,874 (SD = 1,943)	1059 (SD = 938)	5.7 (SD = 5.1)
Enhanced FoS	18.0	589,301 (SD = 450,541)	3,185 (SD = 2,435)	1293 (SD = 1466)	7.0 (SD = 7.9)

Table 11: Trial 2 outcome parameters. Percentage of transfusions acceptable (primary outcome), volume of blood transfused (secondary outcome), and number of SHOT events (secondary outcome).

	**Percentage of transfusions acceptable**	**Volume of RBC transfused**	**Mean RBC per site**	**Volume of platelets transfused**	**Mean platelets per site**	**Number of SHOT events**	**Mean per site**
Standard content	74.4	625,753 (SD = 437,078)	3,226 (SD = 2,253)	90,122 (SD = 112,250)	465 (SD = 579)	1355 (SD = 1674)	7.0 (SD = 8.6)
Enhanced content	71.4	635,726 (SD = 456,894)	3,277 (SD = 2,355)	94,699 (SD = 130,297)	488 (SD = 672)	1285 (SD = 1061)	6.6 (SD = 5.5)
Standard FoS	73.9	584,232 (SD = 423,981)	3,012 (SD = 2,185)	43,714 (SD = 60,247)	225 (SD = 311)	1256 (SD = 914)	6.5 (SD = 4.7)
Enhanced FoS	72.1	618,725 (SD = 428,452)	3,189 (SD = 2,209)	46,438 (SD = 58,331)	239 (SD = 301)	1384 (SD = 1750)	7.1 (SD = 9.0)

© 2022 Stanworth SJ et al. *JAMA Network Open*.

Table 12: Standard vs Enhanced Content analyses, ICER

For the Standard vs Enhanced Content analyses, the ICER can be given as:

**Standard content audit report (Control)**	**Enhanced content audit report (Intervention)**
**Costs$_{\text{(Standard Content)}}$ =**<br>Cost of collecting audit data and delivering standard audit report<br>+<br>Cost of activity sites conducted as a result of receiving audit report<br>+<br>Cost of blood units transfused	**Costs$_{\text{(Enhanced Content)}}$ =**<br>Cost of collecting audit data and delivering enhanced audit report<br>+<br>Cost of activity sites conducted as a result of receiving audit report<br>+<br>Cost of blood units transfused
**Benefits$_{\text{(Standard Content)}}$ =**<br>The percentage of transfusions given that were acceptable	**Benefits$_{\text{(Enhanced Content)}}$ =**<br>The percentage of transfusions given that were acceptable
**Incremental Cost-Effectiveness Ratio =**<br>**(Costs$_{\text{(Enhanced Content)}}$ - Costs$_{\text{(Standard Content)}}$) / (Benefits$_{\text{(Enhanced Content)}}$ - Benefits$_{\text{(Standard Content)}}$)**	

© 2022 Stanworth SJ et al. *JAMA Network Open*.

Table 13: Standard vs Enhanced Follow-on Support analyses, ICER

For the Standard vs Enhanced Follow-on Support (FoS) analyses, the ICER can be given as:

**Standard FoS (Control)**	**Enhanced FoS (Intervention)**
**Costs$_{(Standard\ FoS)}$ =**<br>Cost of collecting audit data and delivering standard audit report<br>+<br>Cost of delivering standard FoS<br>+<br>Cost of activity sites conducted as a result of receiving audit report<br>+<br>Cost of blood units transfused	**Costs$_{(Enhanced\ FoS)}$ =**<br>Cost of collecting audit data and delivering standard audit report<br>+<br>Cost of delivering enhanced FoS<br>+<br>Cost of activity sites conducted as a result of receiving audit report<br>+<br>Cost of blood units transfused
**Benefits$_{(Standard\ FoS)}$ =**<br>The percentage of transfusions given that were acceptable	**Benefits$_{(Enhanced\ FoS)}$ =**<br>The percentage of transfusions given that were acceptable
**Incremental Cost-Effectiveness Ratio =**<br>**(Costs$_{(Enhanced\ FoS)}$ - Costs$_{(Standard\ FoS)}$) / (Benefits$_{(Enhanced\ FoS)}$ - Benefits$_{(Standard\ FoS)}$)**	

© 2022 Stanworth SJ et al. *JAMA Network Open*.

Table 14: cost components of the interventions for Trial 1

Trial 1

	**Standard Content**		**Enhanced Content**		**Difference (i.e. Additional cost of Enhanced)**	
	**Cost for all 185 sites**	**Cost per site**	**Cost for all 185 sites**	**Cost per site**	**All sites**	**Per site**
Audit data collection	£93,841	£507	£93,841	£507	£0	£0
Feedback interventions	£22,739	£123	£66,604	£360	£43,864	£237
Additional NHS activity	£228,666	£1,236	£225,228	£1,217	-£3,438	-£19
Blood transfused	£90,789,344	£490,753	£115,829,314	£626,104	£25,039,970	£135,351
Total	£91,134,590	£492,619	£116,214,986	£628,165	£25,080,396	£135,570

	**Standard Follow-on Support**		**Enhanced Follow-on Support**		**Difference (i.e. Additional cost of Enhanced)**	
	**Cost for all 185 sites**	**Cost per site**	**Cost for all 185 sites**	**All sites**	**All sites**	**Per site**
Audit data collection	£93,841	£507	£93,841	£507	£0	£0
Feedback interventions	£22,739	£123	£28,721	£155	£5,982	£32
Additional NHS activity	£228,311	£1,234	£225,625	£1,220	-£2,686	-£15
Blood transfused	£93,446,854	£505,118	£103,586,378	£559,926	£10,139,524	£54,808
Total	£93,791,745	£506,982	£103,934,565	£561,808	£10,142,820	£54,826

© 2022 Stanworth SJ et al. *JAMA Network Open*.

Table 15: cost components of the interventions for Trial 2

Trial 2	Standard Content		Enhanced Content		Difference (i.e. Additional cost of Enhanced)	
	Cost for all 194 sites	Cost per site	Cost for all 194 sites	All sites	All sites	Per site
Audit data collection	£110,507	£570	£110,507	£570	£0	£0
Feedback interventions	£17,069	£88	£60,933	£314	£43,864	£226
Additional NHS activity	£178,476	£920	£182,755	£942	£4,278	£22
Blood transfused	£130,677,492	£673,595	£133,481,117	£688,047	£2,803,625	£14,452
Total	£130,983,544	£675,173	£133,835,311	£689,873	£2,851,768	£14,700

	Standard follow-on support		Enhanced follow-on support		Difference (i.e. Additional cost of Enhanced)	
	Cost for all 194 sites	Cost per site	Cost for all 194 sites	Cost per site	All sites	Per site
Audit data collection	£110,507	£570	£110,507	£570	£0	£0
Feedback interventions	£17,069	£88	£30,141	£155	£13,073	£67
Additional NHS activity	£208,577	£1,075	£157,169	£810	-£51,407	-£265
Blood transfused	£130,677,492	£673,595	£119,416,399	£615,548	-£11,261,092	-£58,047
Total	£113,064,213	£582,805	£119,714,217	£617,084	£6,650,004	£34,278

© 2022 Stanworth SJ et al. *JAMA Network Open*.

Table 16: Breakdown of calculation for cost-neutral analysis

**Trial 1**	**Cost per site**	**units of RBC equivalent needed to be cost-neutral**
Enhanced vs Standard Content		
Incremental mean	£219	1.2
Incremental Standard Deviation	£210	1.2
95%UI upper bound	£630	3.6
95%UI lower bound	-£193	0
Enhanced vs Standard Follow-on Support		
Incremental mean	£18	0.1
Incremental Standard Deviation	£24	0.1
95%UI upper bound	£66	0.4
95%UI lower bound	-£30	0

© 2022 Stanworth SJ et al. *JAMA Network Open*.

Table 17: Breakdown of calculation for cost-neutral analysis

Trial 2	Cost per site	units of RBC equivalent
Enhanced vs Standard Content		
Incremental mean	£248	1.4
Incremental Standard Deviation	£230	1.3
95%UI upper bound	£698	4.0
95%UI lower bound	-£202	0
Enhanced vs Standard Follow-on Support		
Incremental mean	-£198	0
Incremental Standard Deviation	£19	0.1
95%UI upper bound	-£161	0
95%UI lower bound	-£234	0

© 2022 Stanworth SJ et al. *JAMA Network Open*.

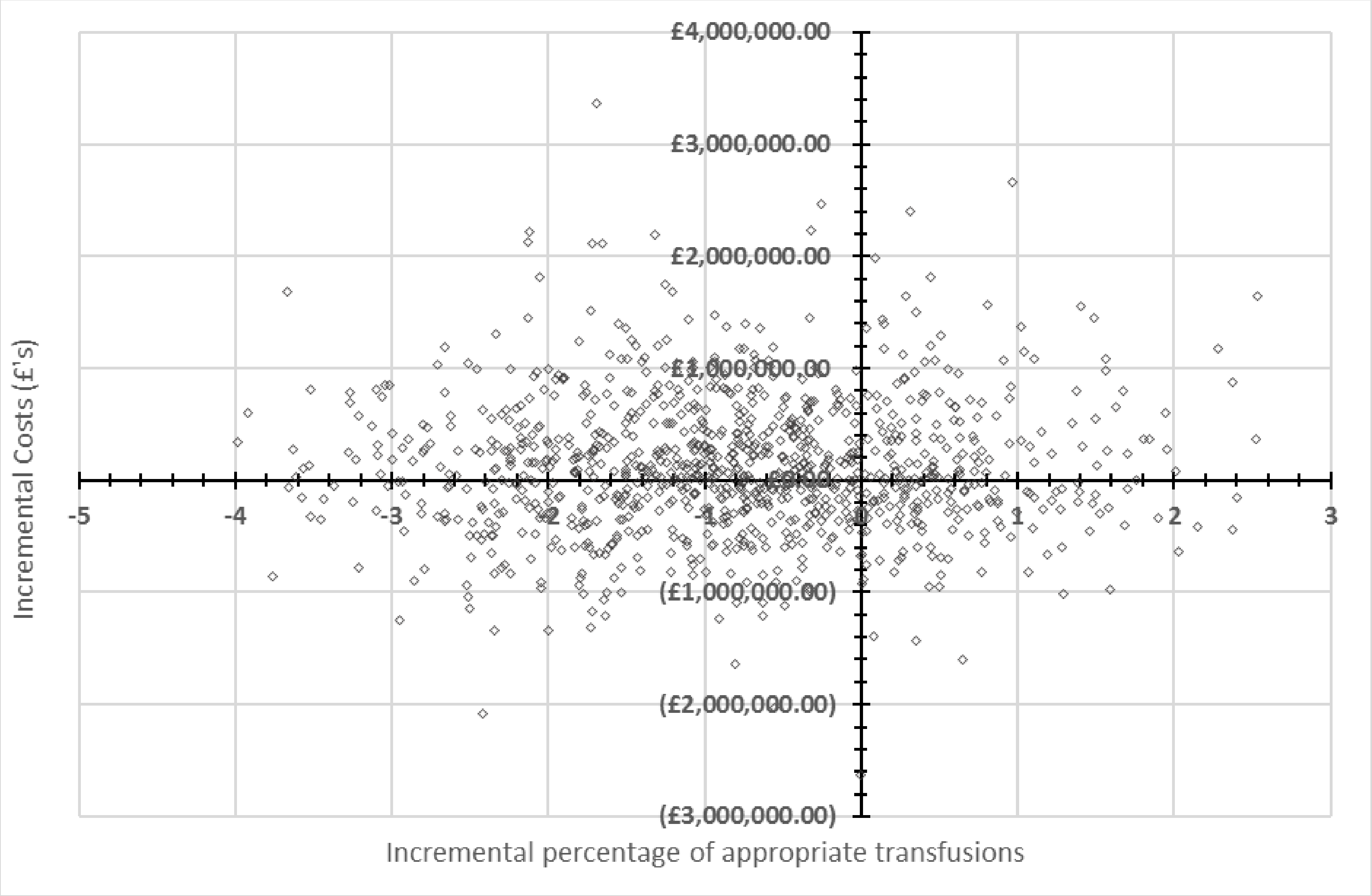

Figure 2: Simulated outputs on the cost-effectiveness plane (CEP) for Trial 1 enhanced vs standard content for the primary outcome. Each grey diamonds represents one individual simulated output of the model.

© 2022 Stanworth SJ et al. *JAMA Network Open*.

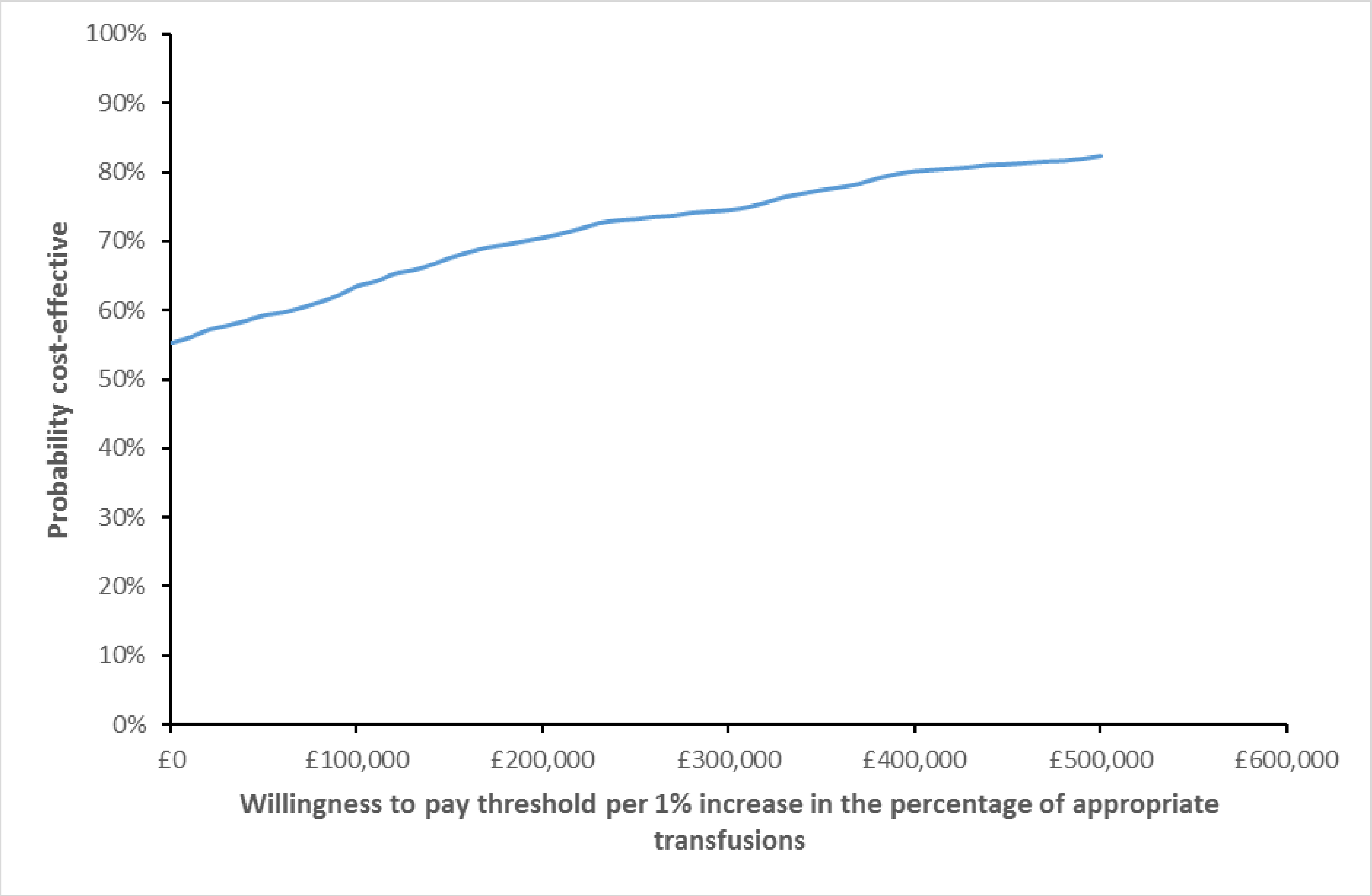

Figure 3: Cost-effectiveness acceptability curve (CEAC) for Trial 1 enhanced vs standard content for the primary outcome. The blue line represents the probability that the intervention is cost-effective at ascending WTP thresholds.

© 2022 Stanworth SJ et al. *JAMA Network Open*.

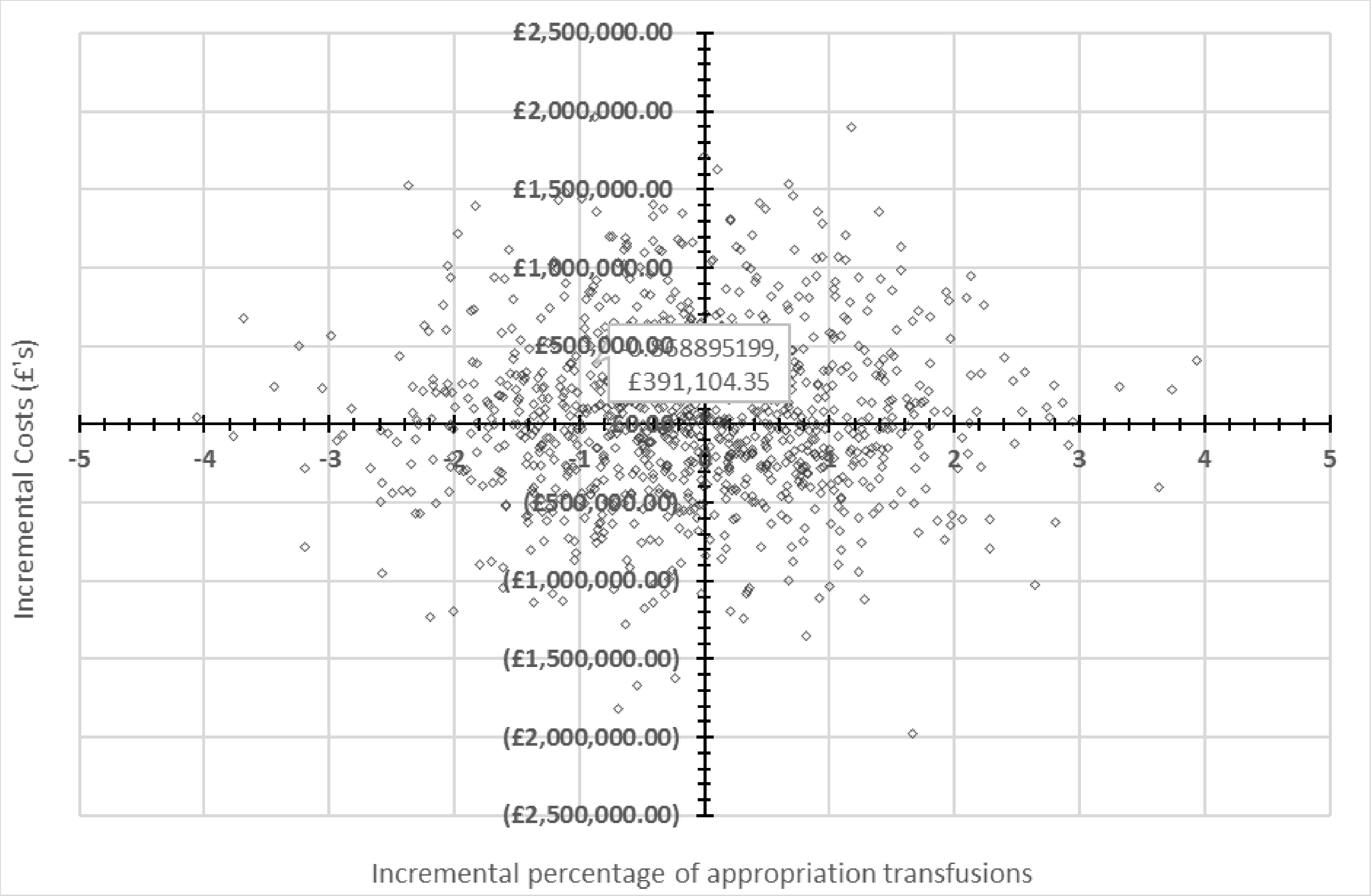

Figure 4: CEP for enhanced vs standard follow-on support for percentage of acceptable transfusions

© 2022 Stanworth SJ et al. *JAMA Network Open*.

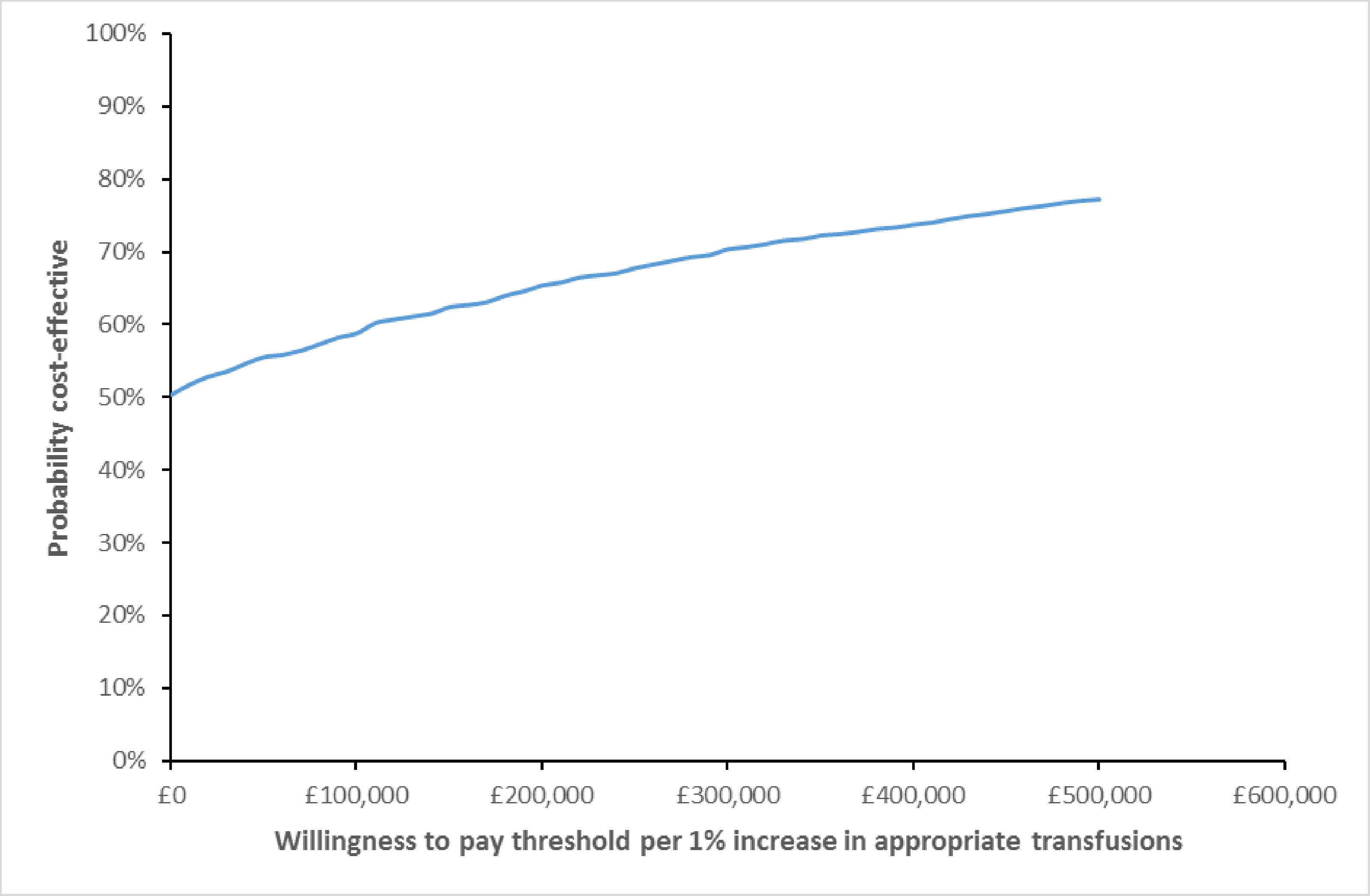

Figure 5: CEAC for enhanced vs standard follow-on support for percentage of acceptable transfusions

© 2022 Stanworth SJ et al. *JAMA Network Open*.

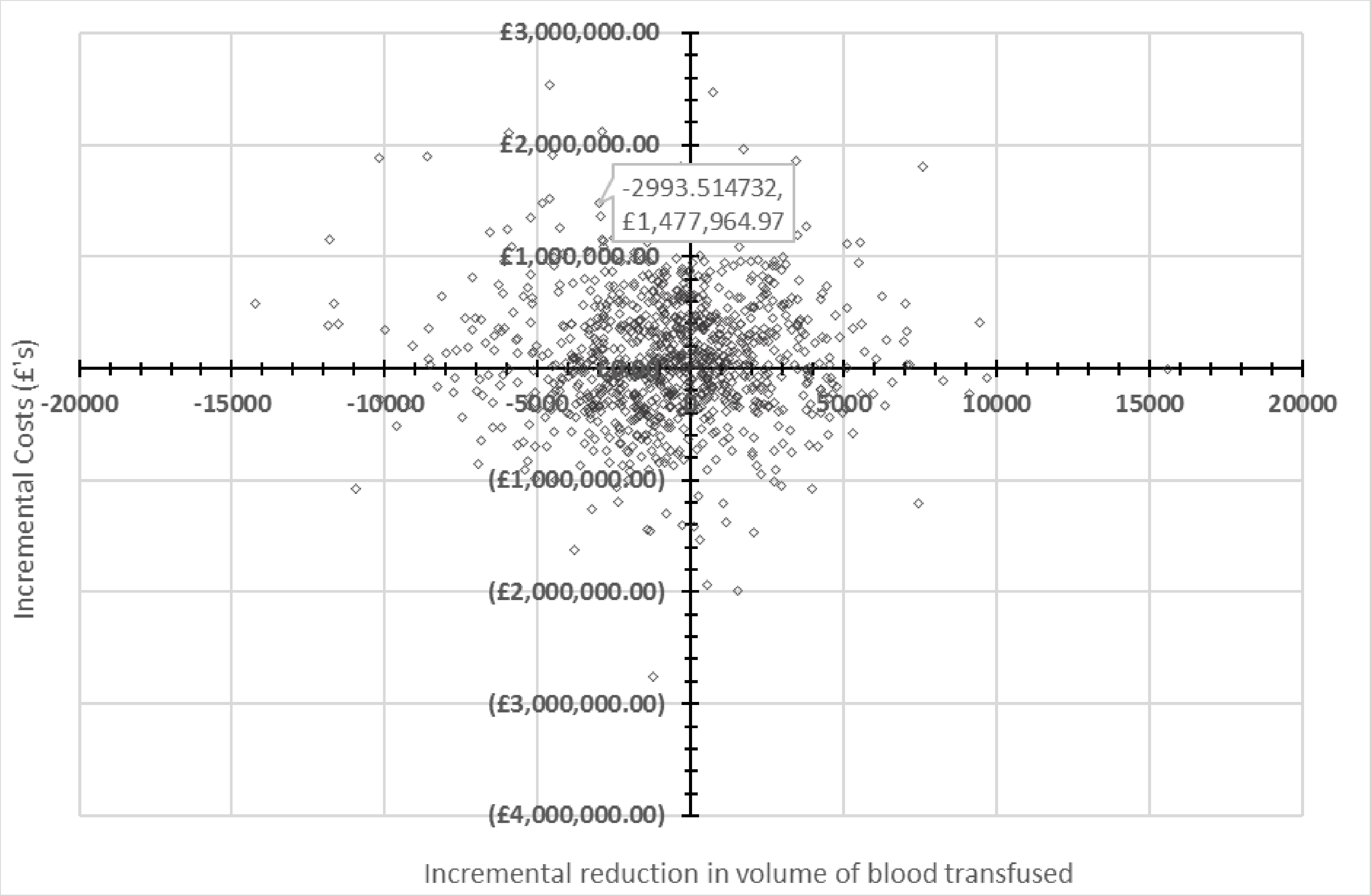

Figure 6: CEP for Enhanced vs Standard Content for Trial 1 volume of blood transfused

© 2022 Stanworth SJ et al. *JAMA Network Open*.

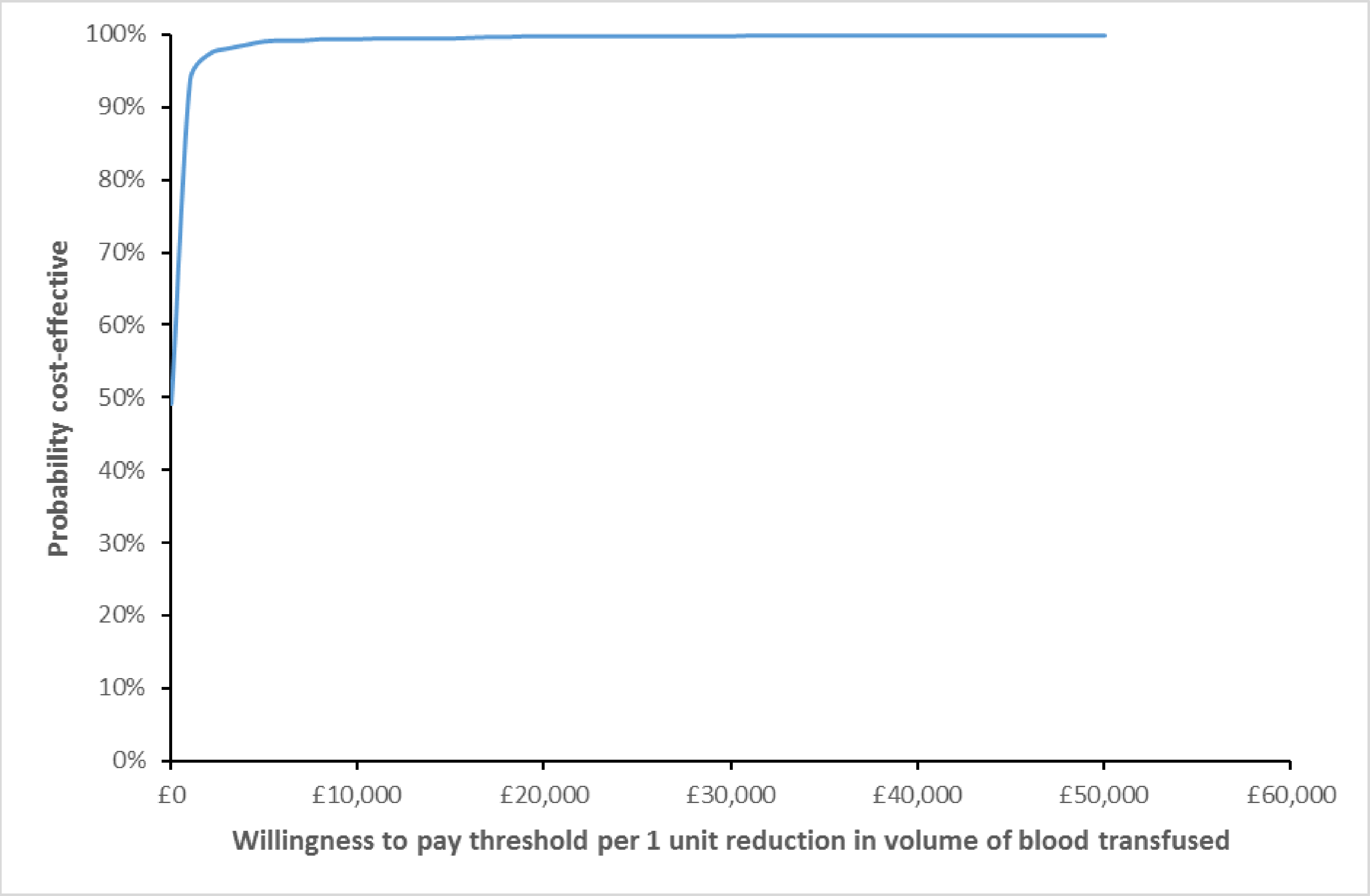

Figure 7: CEAC for Enhanced vs Standard Content for Trial 1 volume of blood transfused

© 2022 Stanworth SJ et al. *JAMA Network Open*.

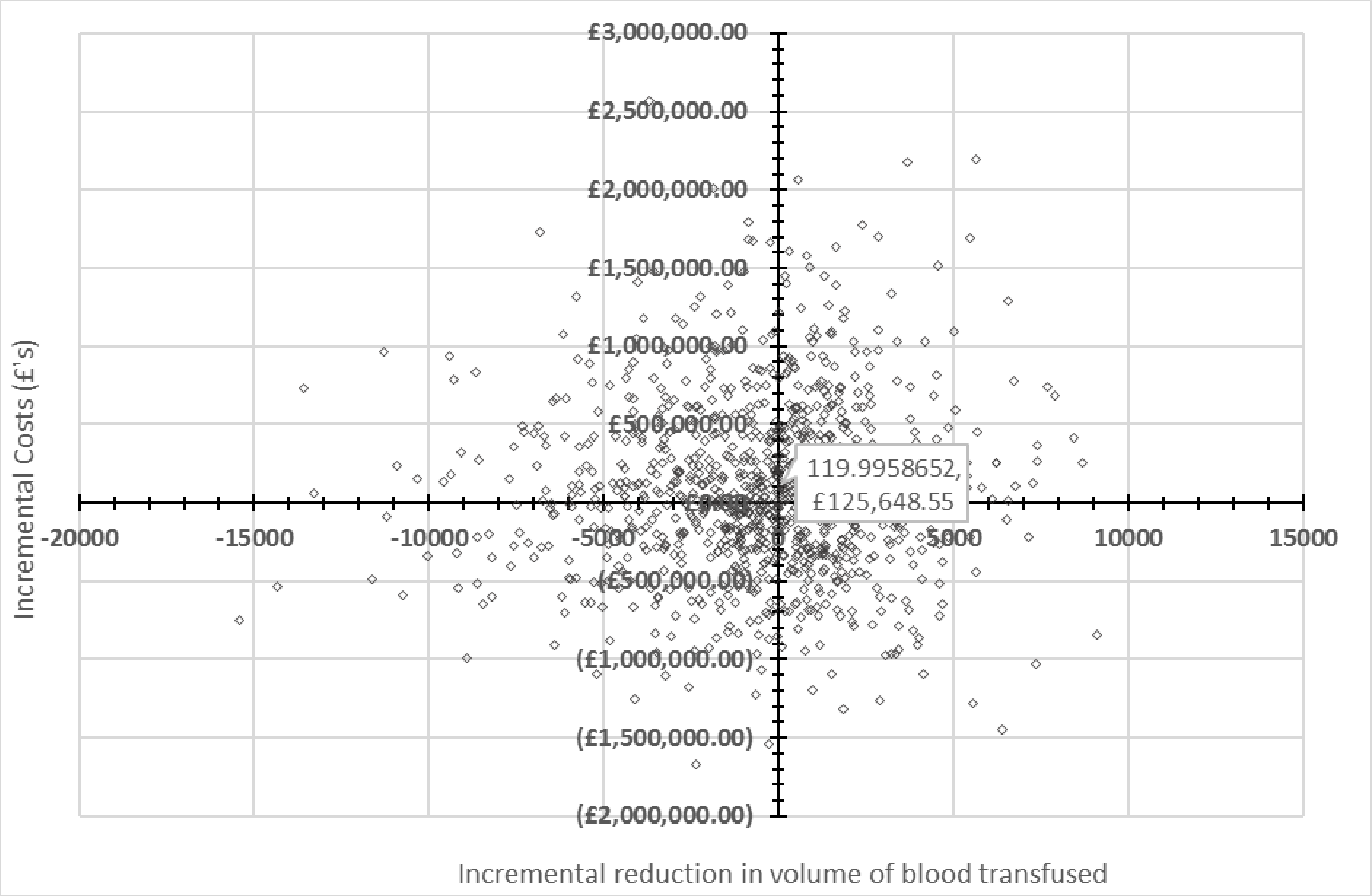

Figure 8: CEP for Enhanced vs Standard Follow-on Support for Trial 1 volume of blood transfused

© 2022 Stanworth SJ et al. *JAMA Network Open*.

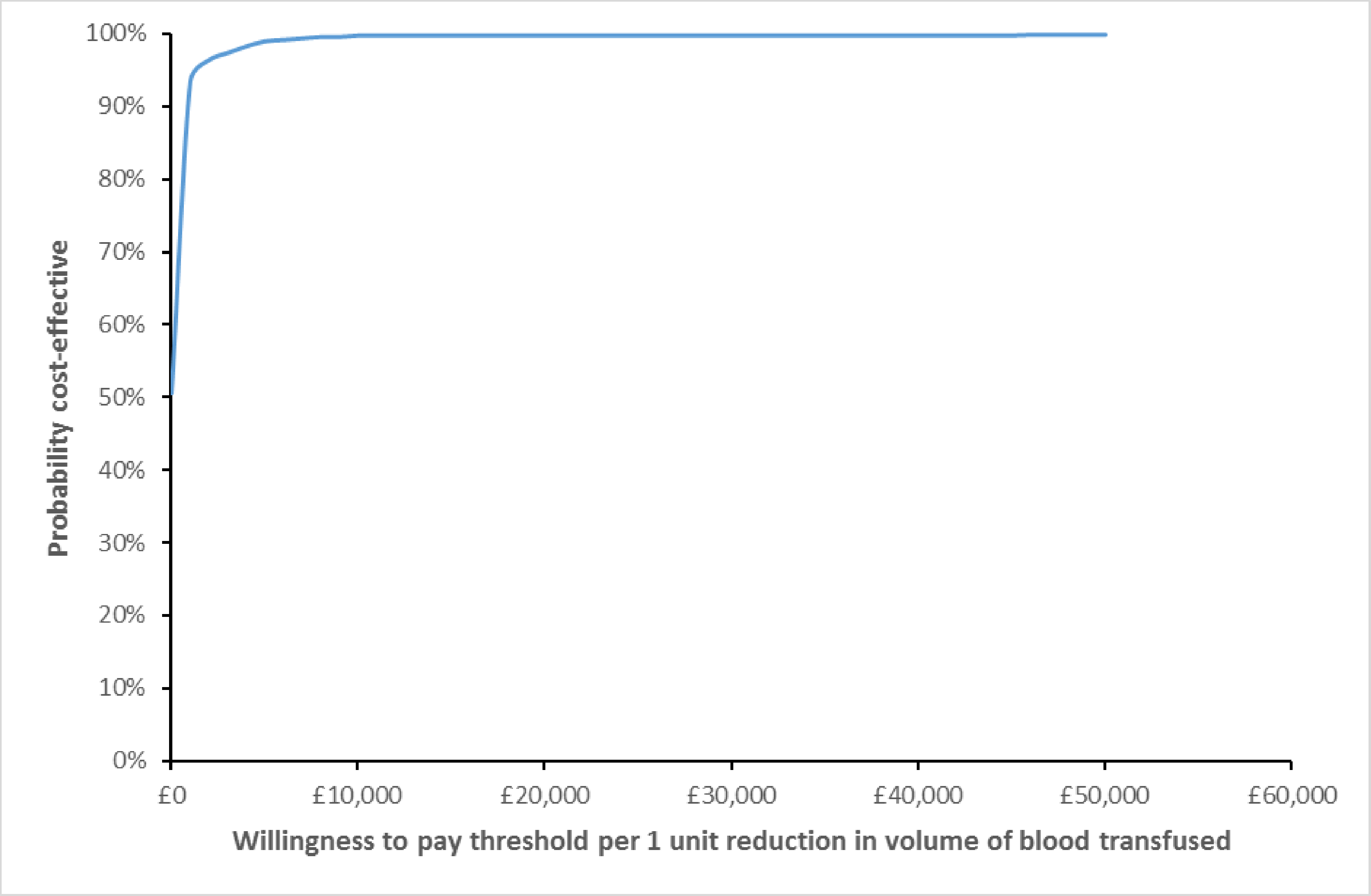

Figure 9: CEAC for Enhanced vs Standard Follow-on Support for Trial 1 volume of blood transfused

© 2022 Stanworth SJ et al. *JAMA Network Open*.

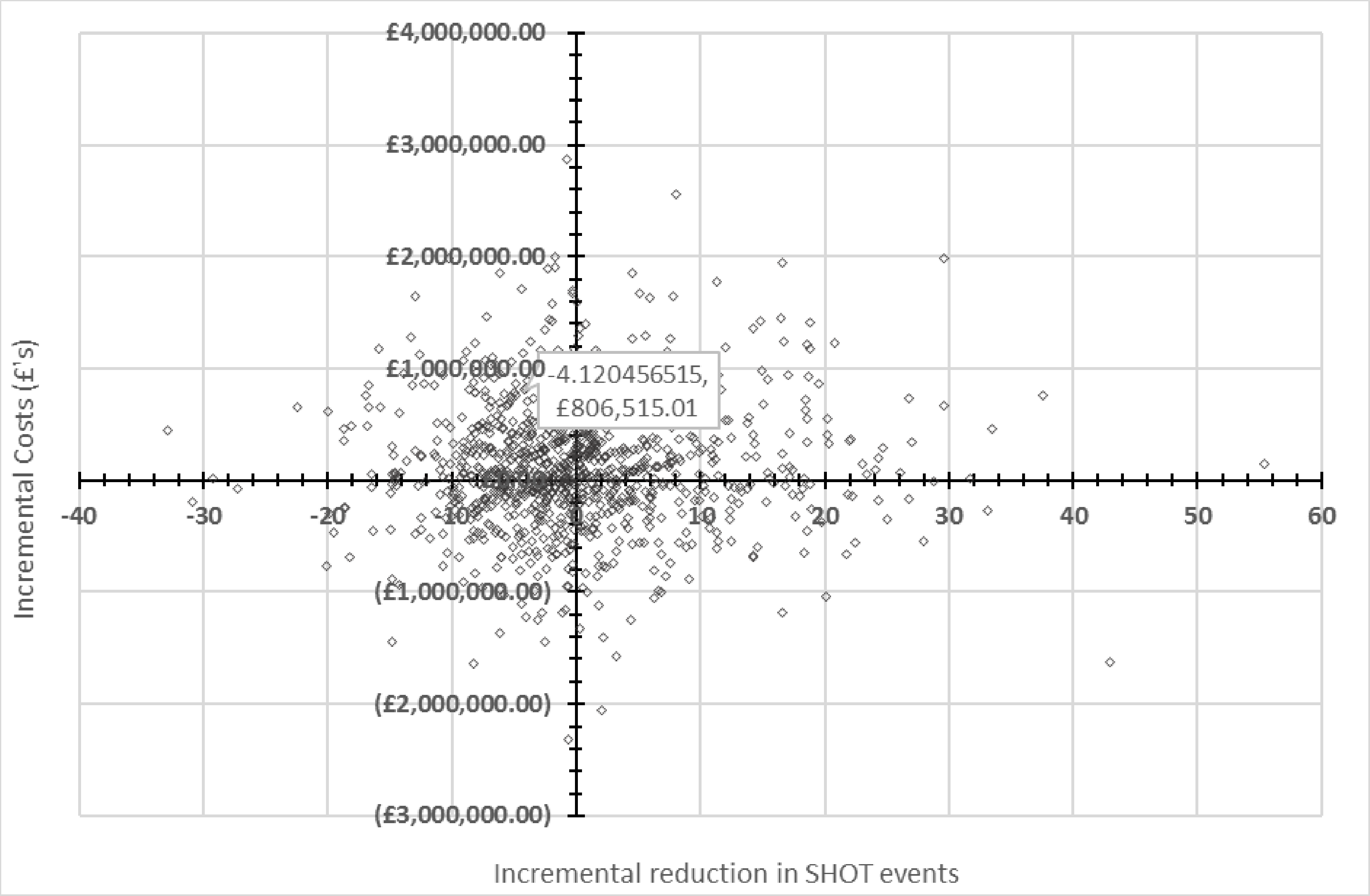

Figure 10: CEP for Enhanced vs Standard Content for Trial 1 SHOT events

© 2022 Stanworth SJ et al. *JAMA Network Open*.

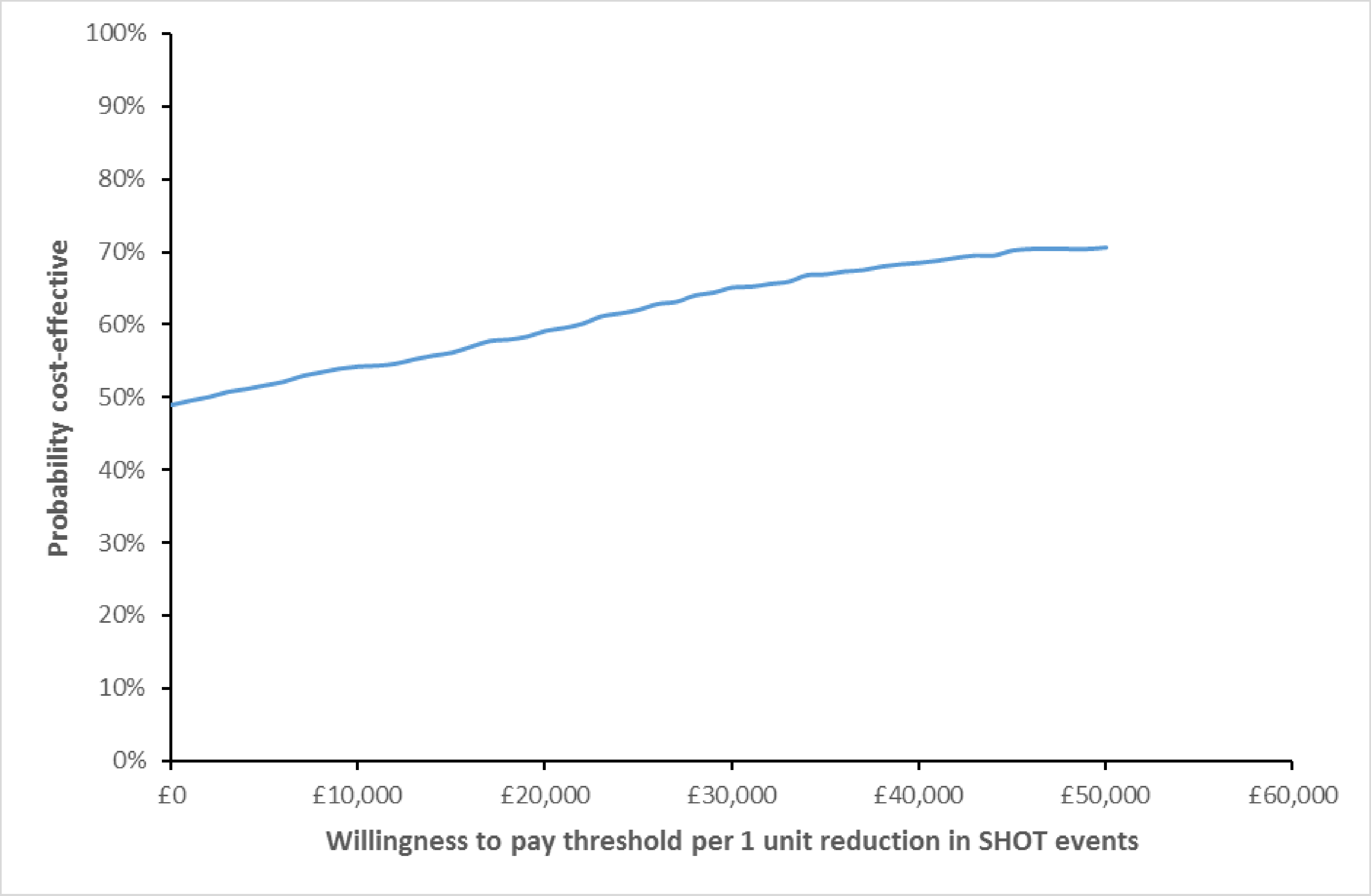

Figure 11: CEAC for Enhanced vs Standard Content for Trial 1 SHOT events

© 2022 Stanworth SJ et al. *JAMA Network Open*.

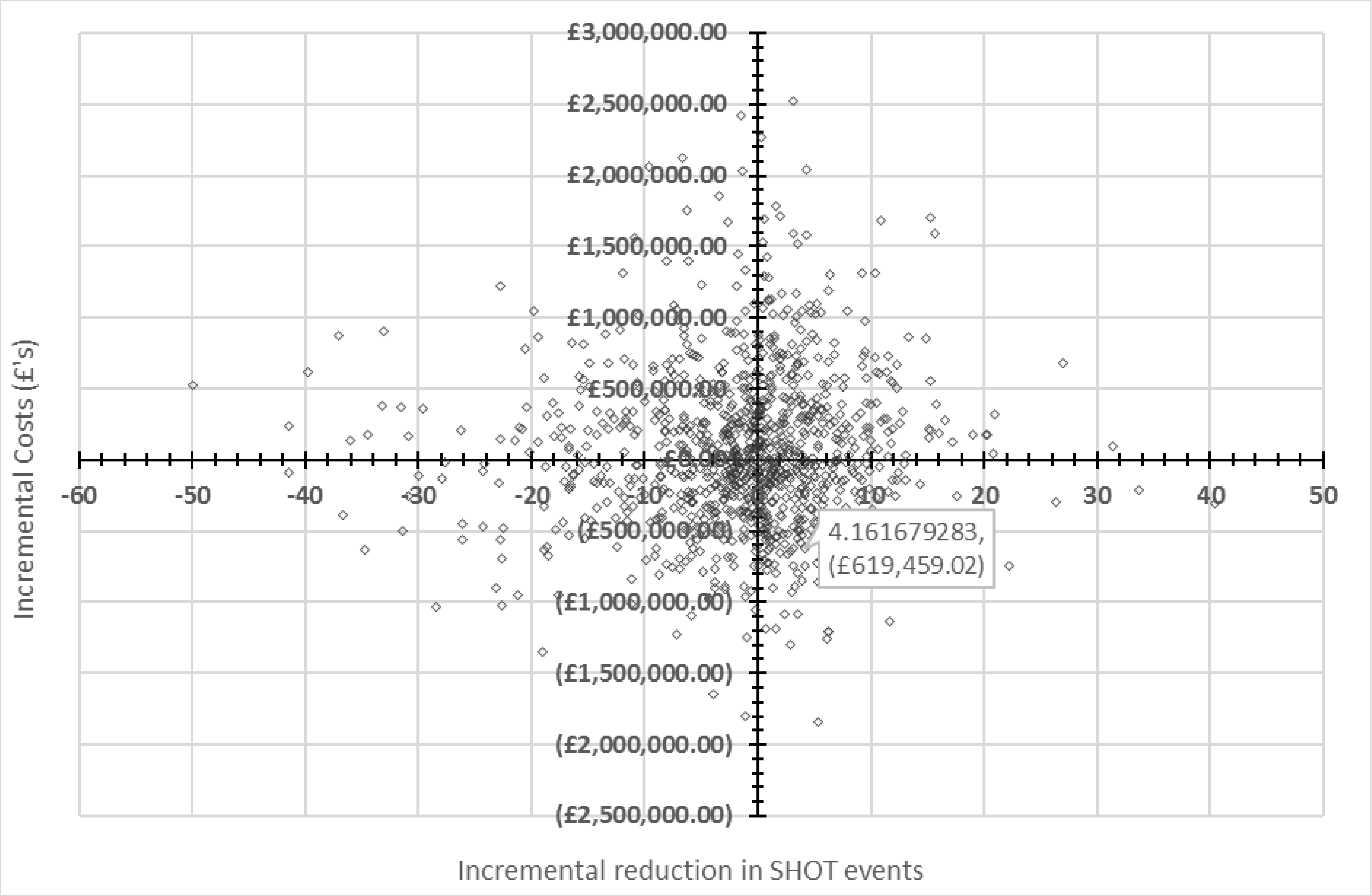

Figure 12: CEP for Enhanced vs Standard Follow-on Support for Trial 1 SHOT events

© 2022 Stanworth SJ et al. *JAMA Network Open*.

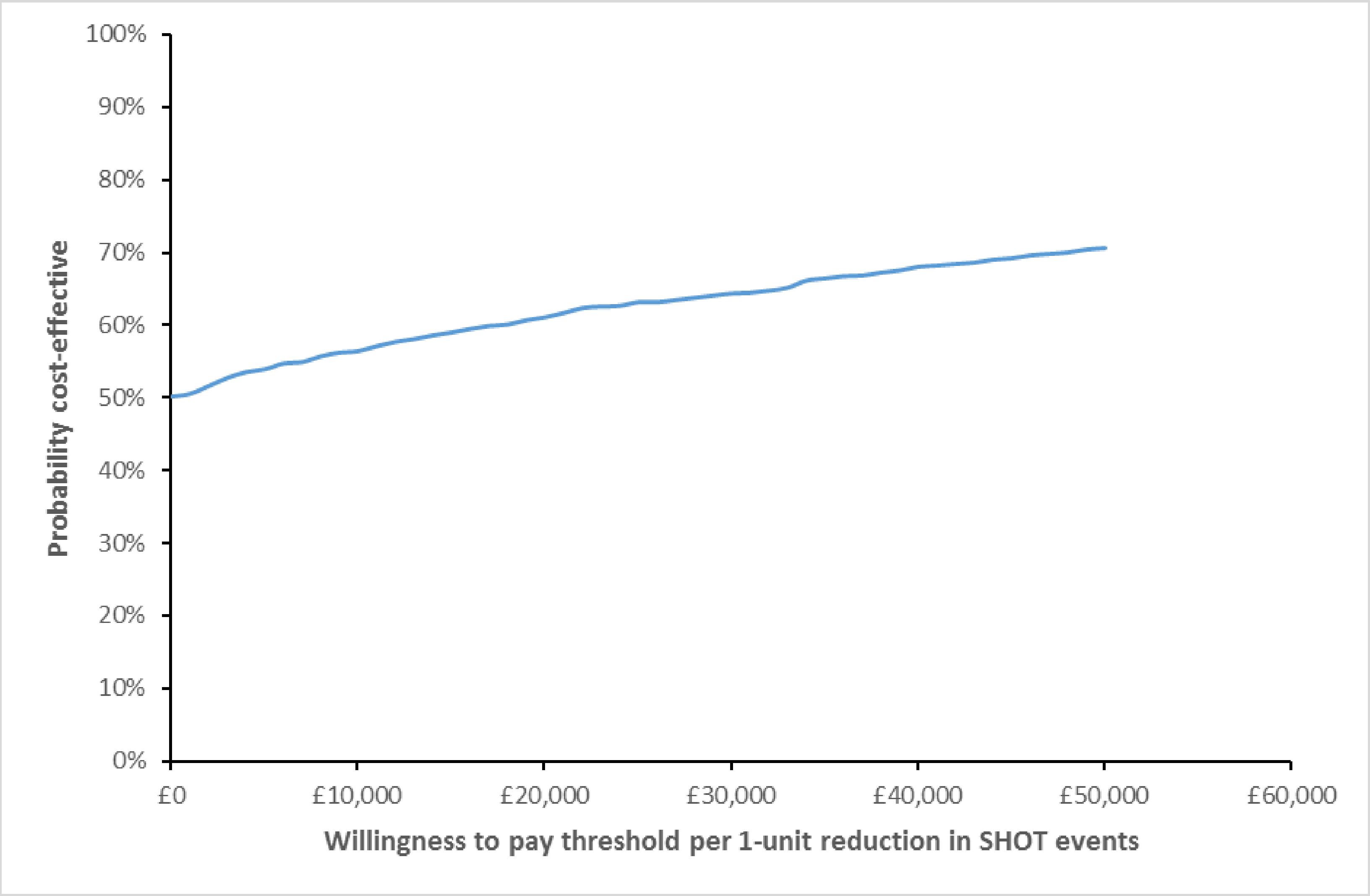

Figure 13: CEAC for Enhanced vs Standard Follow-on Support for Trial 1 SHOT events

© 2022 Stanworth SJ et al. *JAMA Network Open*.

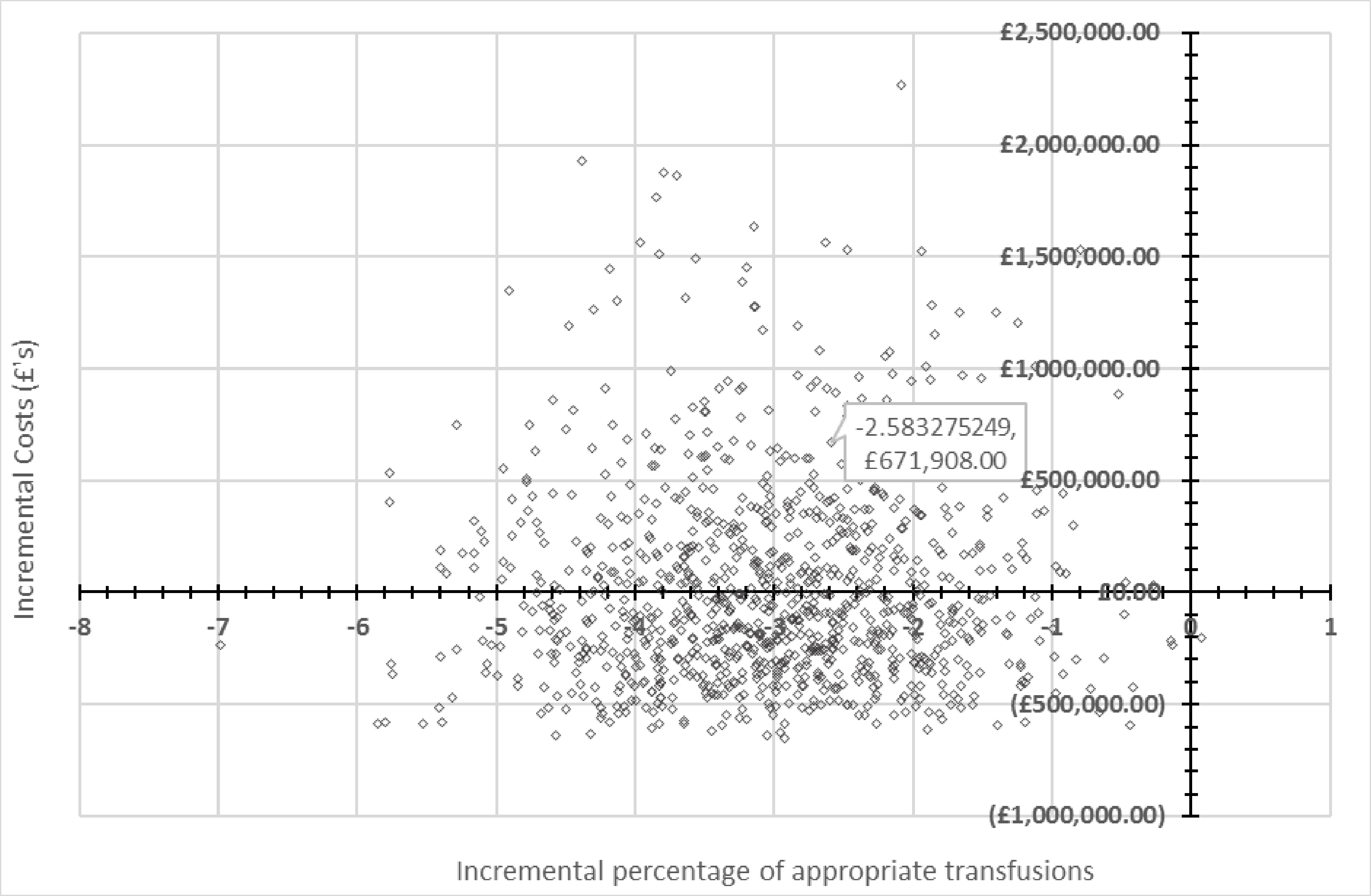

Figure 14: CEP for Enhanced vs Standard Content for Trial 2 Percentage of Transfusions Acceptable

© 2022 Stanworth SJ et al. *JAMA Network Open*.

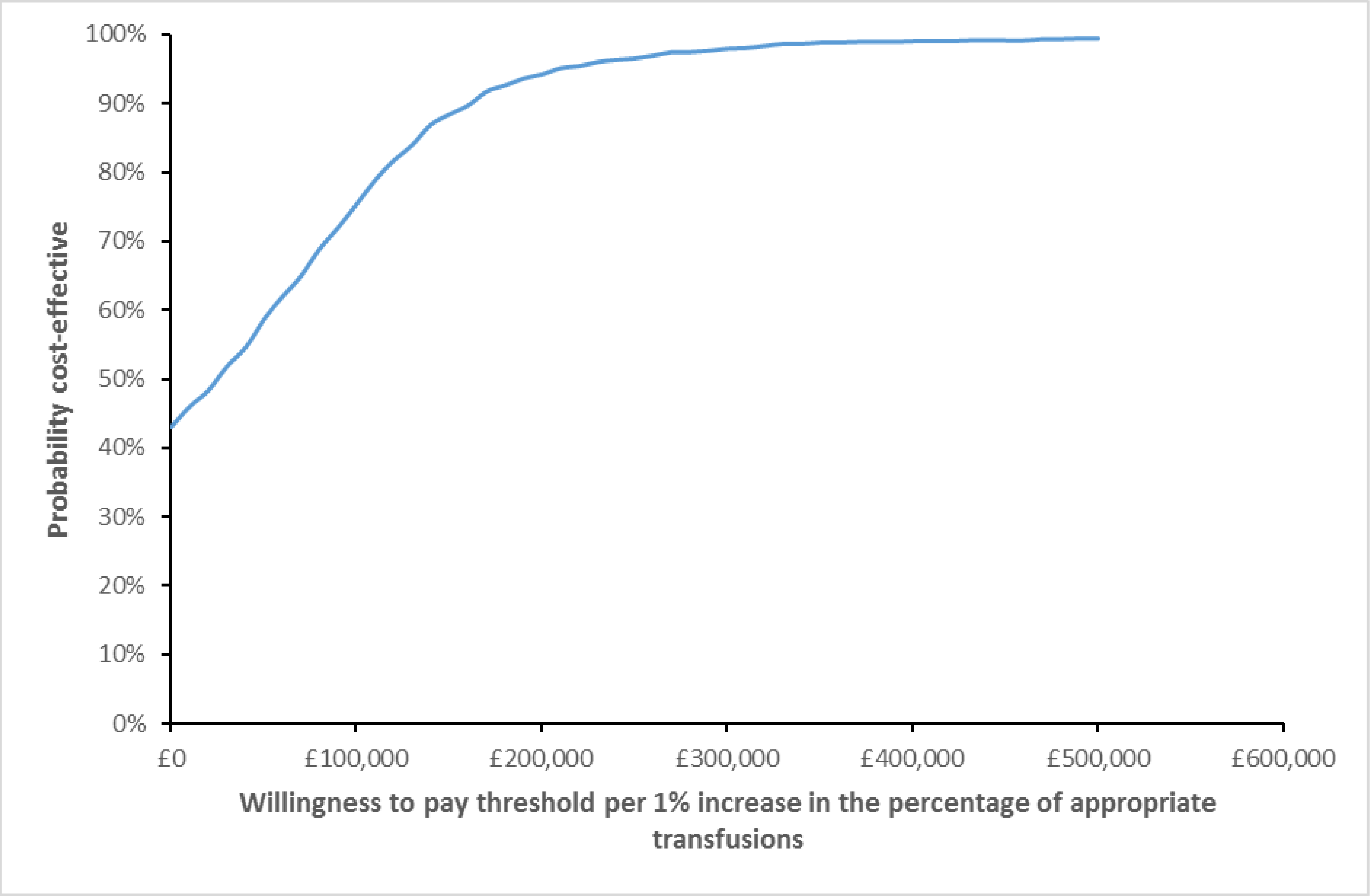

Figure 15: CEAC for Enhanced vs Standard Content for Trial 2 Percentage of Transfusions Acceptable

© 2022 Stanworth SJ et al. *JAMA Network Open*.

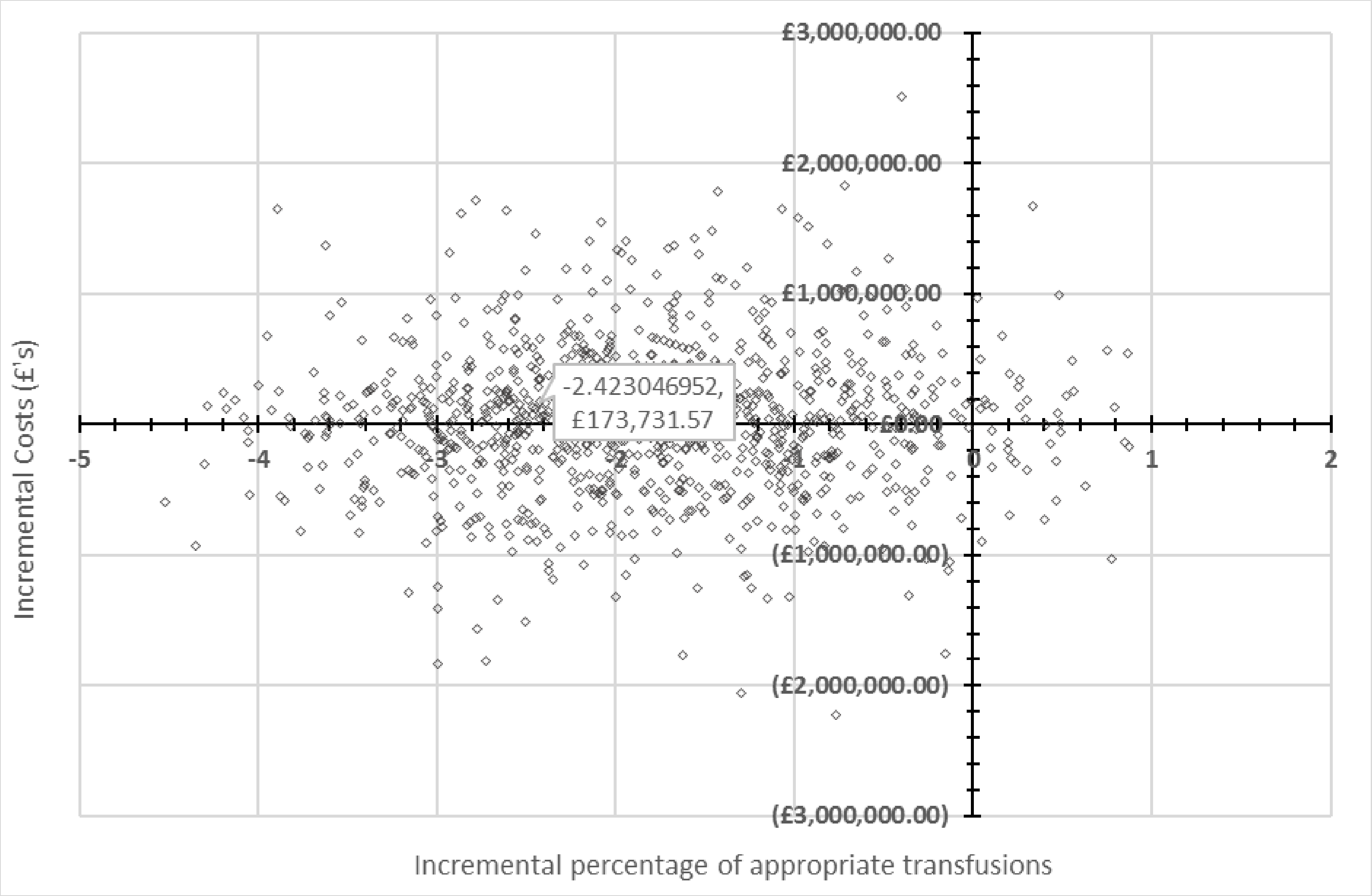

Figure 16: CEP for Enhanced vs Standard Follow-on Support for Trial 2 Percentage of Transfusions Acceptable

© 2022 Stanworth SJ et al. *JAMA Network Open*.

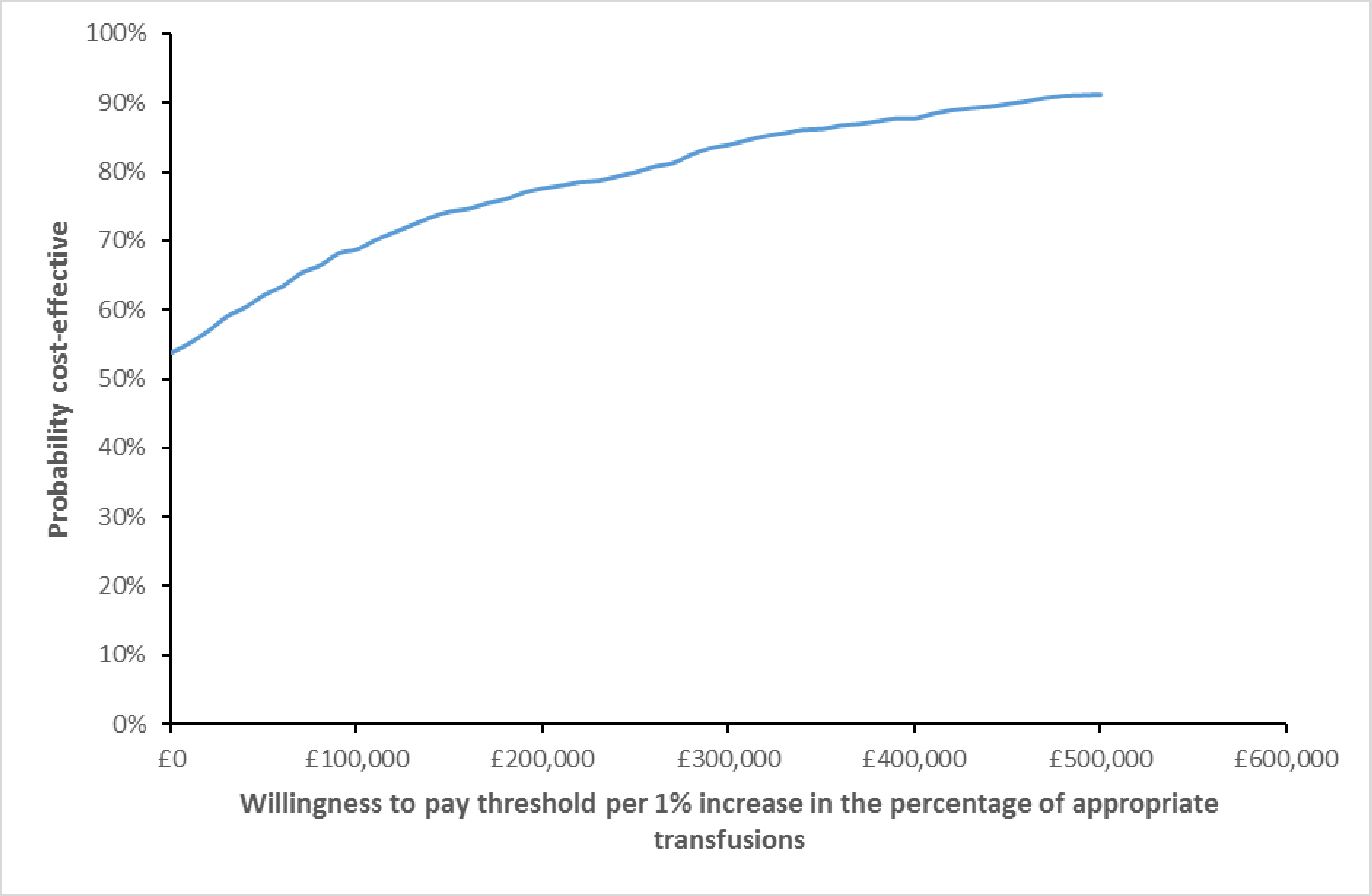

Figure 17: CEAC for enhanced vs standard follow-on support for percentage of acceptable transfusions

© 2022 Stanworth SJ et al. *JAMA Network Open*.

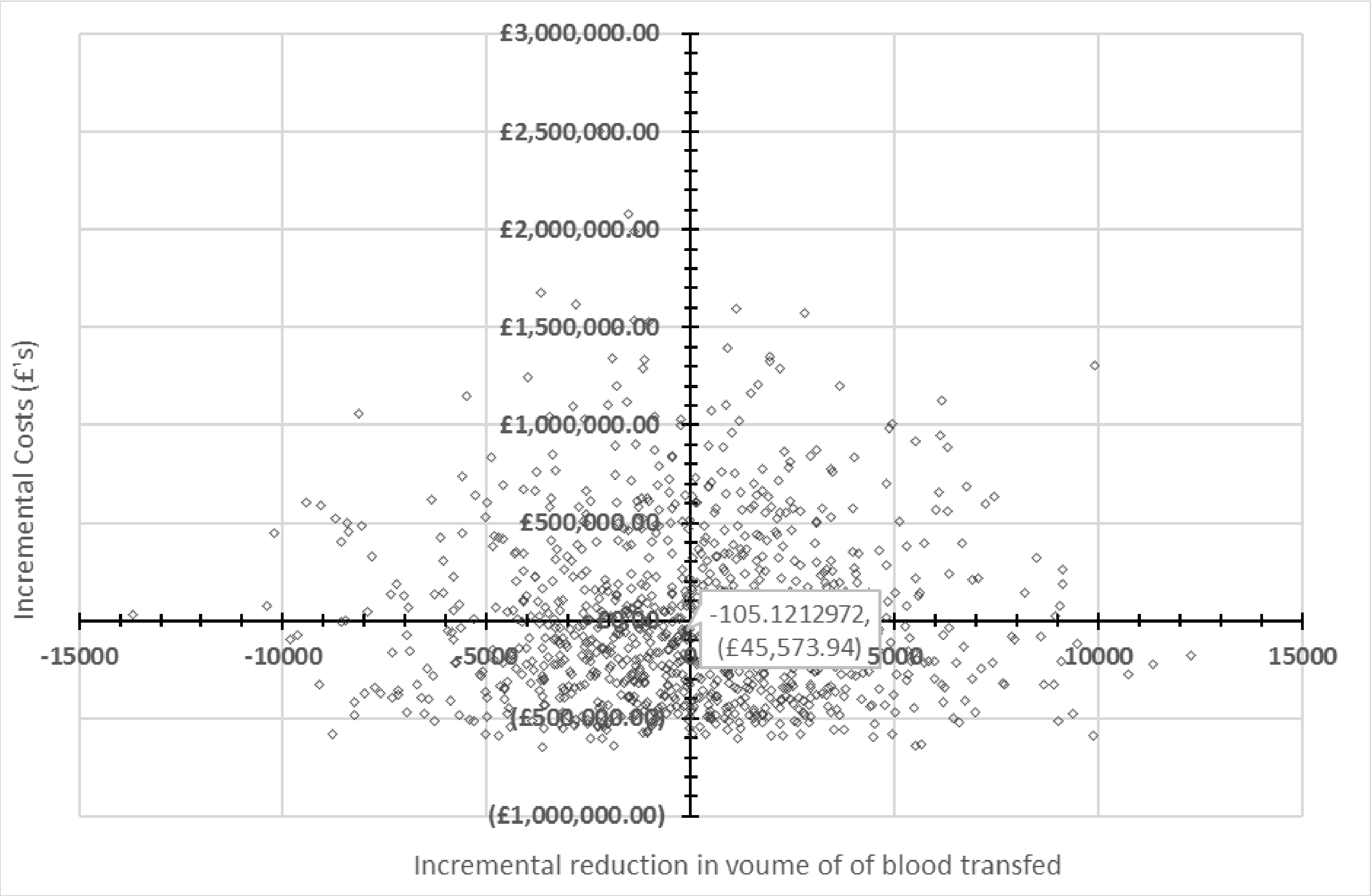

Figure 18: CEP for Enhanced vs Standard Content for Trial 2 volume of blood transfused

© 2022 Stanworth SJ et al. *JAMA Network Open*.

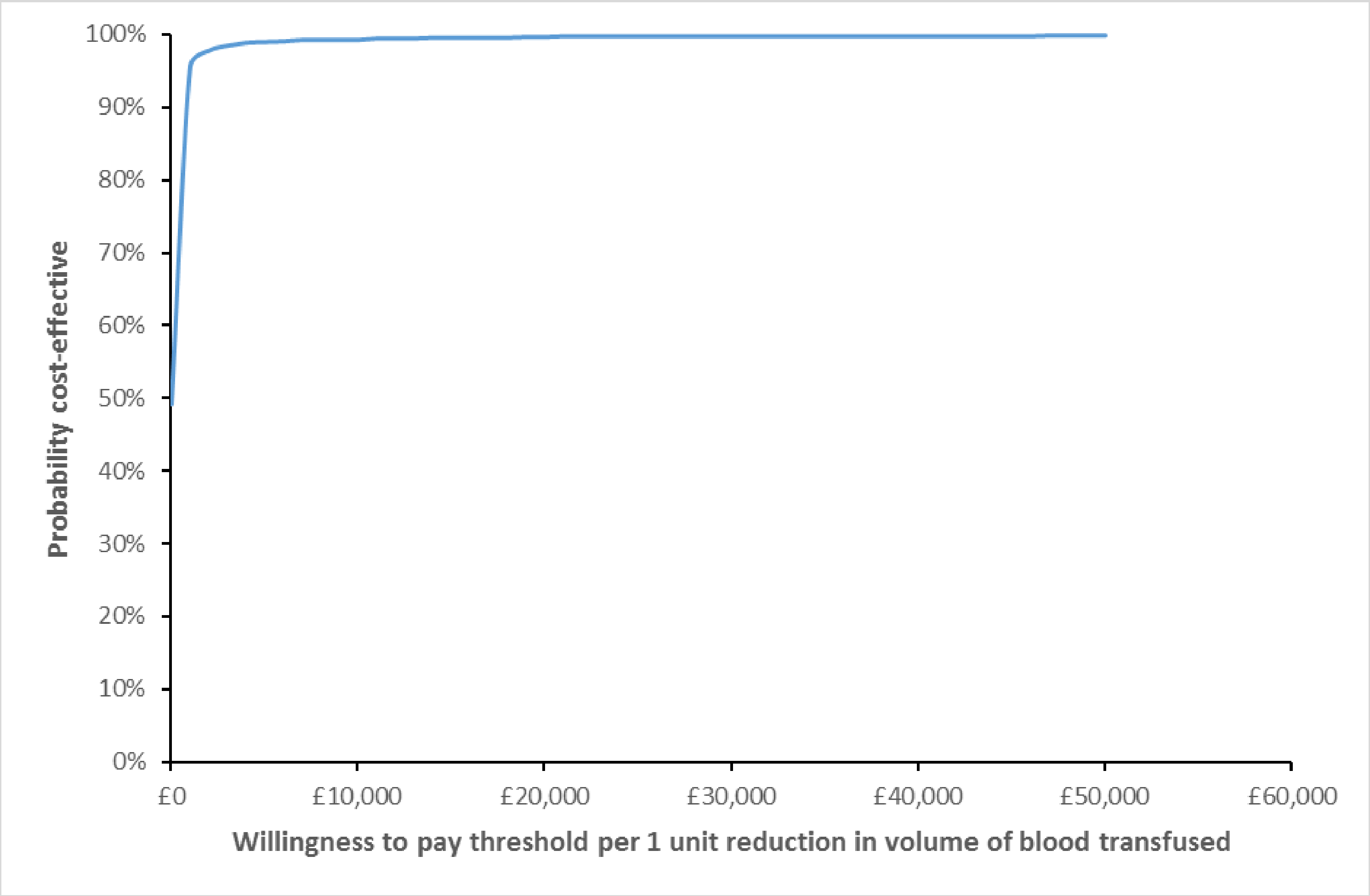

Figure 19: CEAC for Enhanced vs Standard Content for Trial 2 volume of blood Transfused

© 2022 Stanworth SJ et al. *JAMA Network Open*.

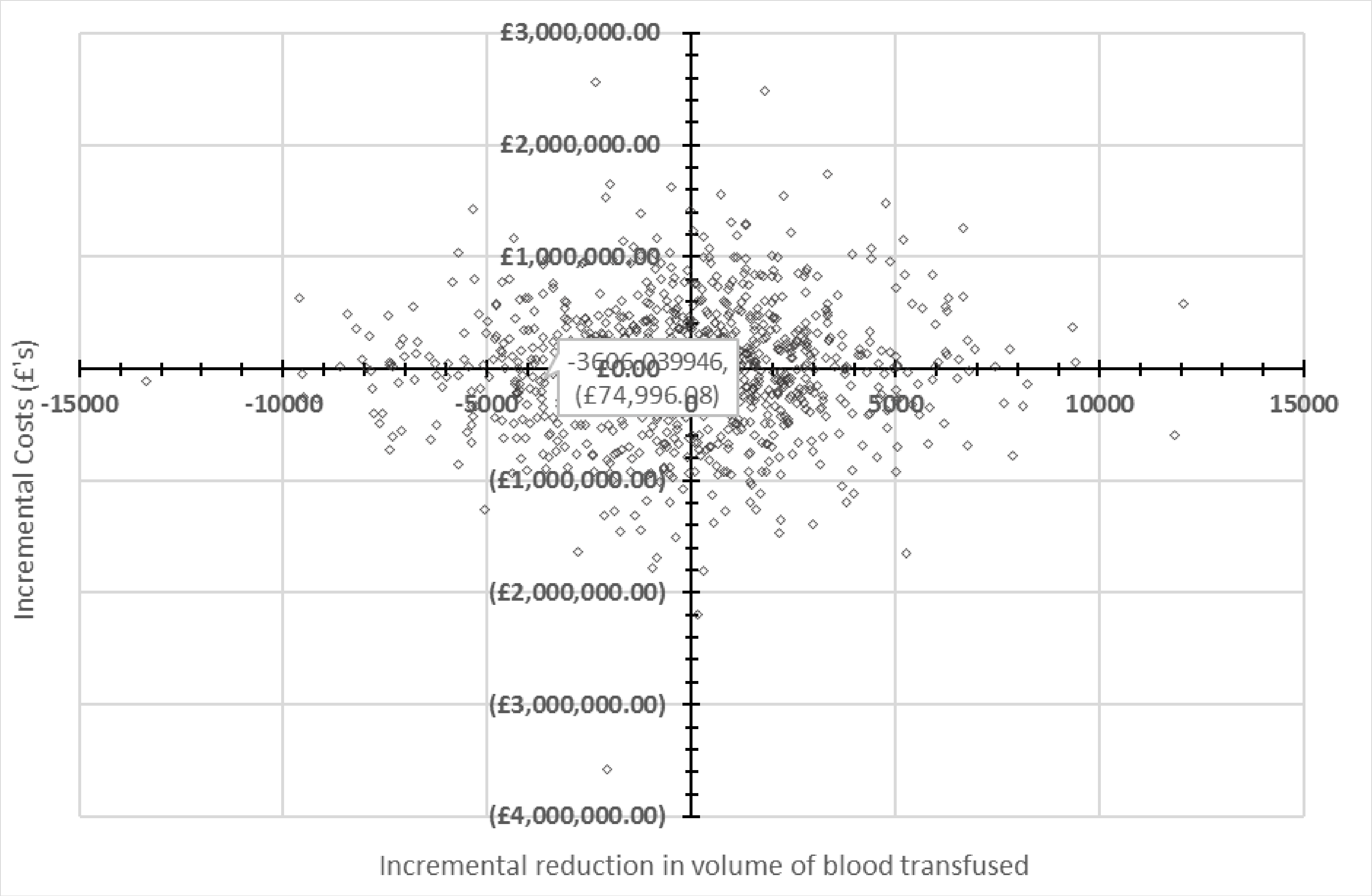

Figure 20: CEP for Enhanced vs Standard Follow-on Support for Trial 2 volume of blood transfused

© 2022 Stanworth SJ et al. *JAMA Network Open*.

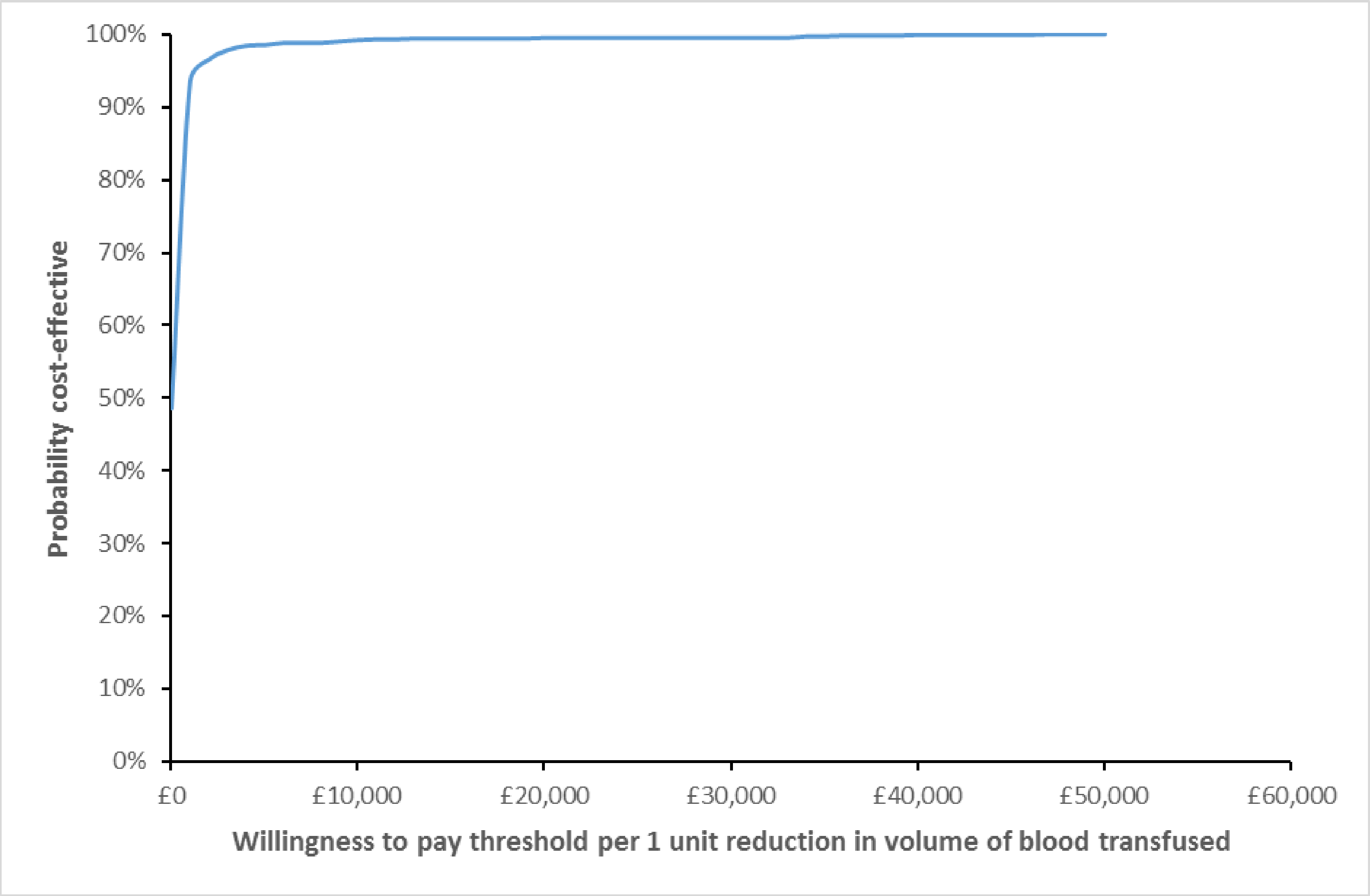

Figure 21: CEAC for Enhanced vs Standard Follow-on Support for Trial 2 volume of blood transfused

© 2022 Stanworth SJ et al. *JAMA Network Open*.

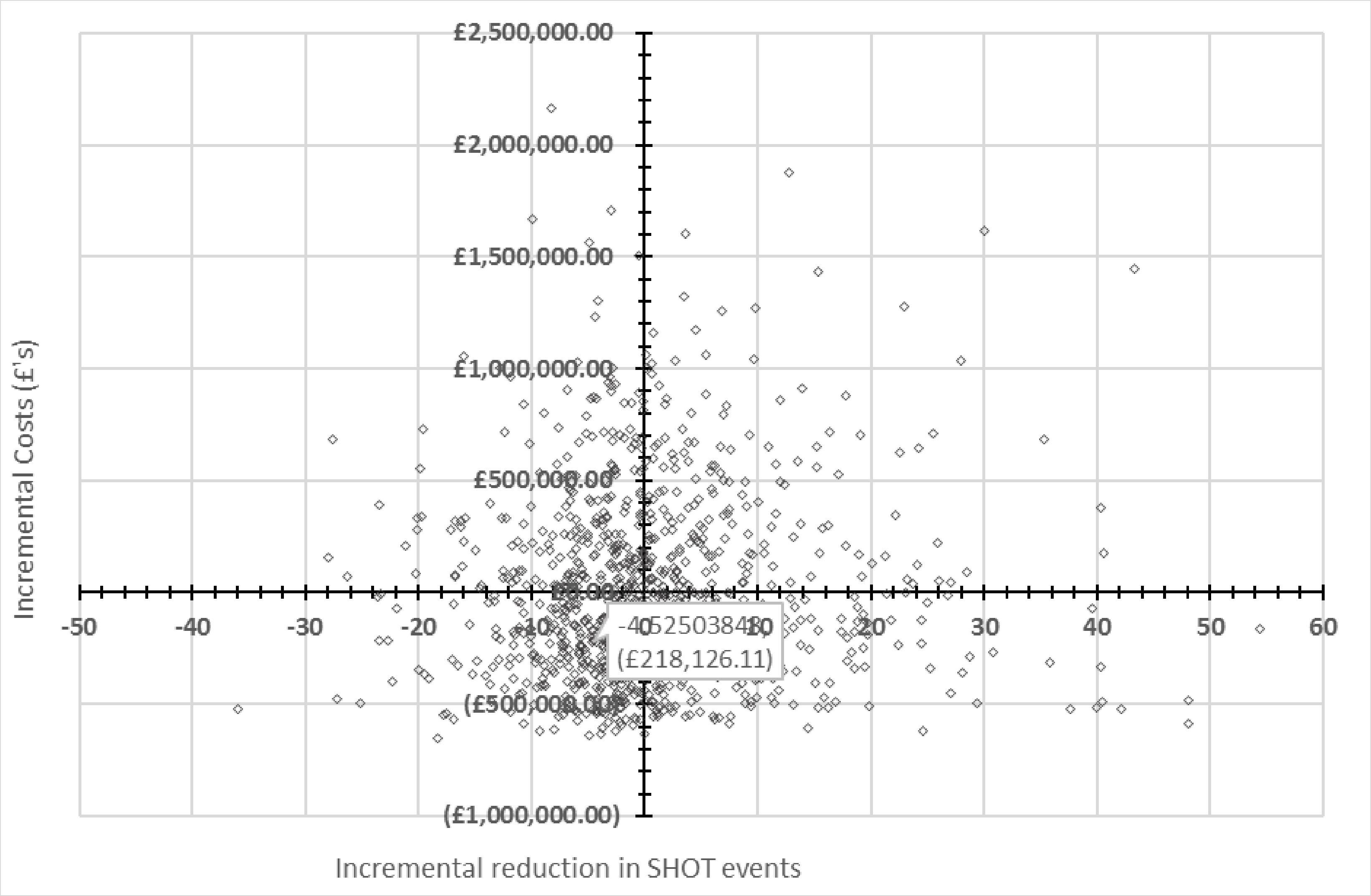

Figure 22: CEP for Enhanced vs Standard Content for Trial 2 SHOT events

© 2022 Stanworth SJ et al. *JAMA Network Open*.

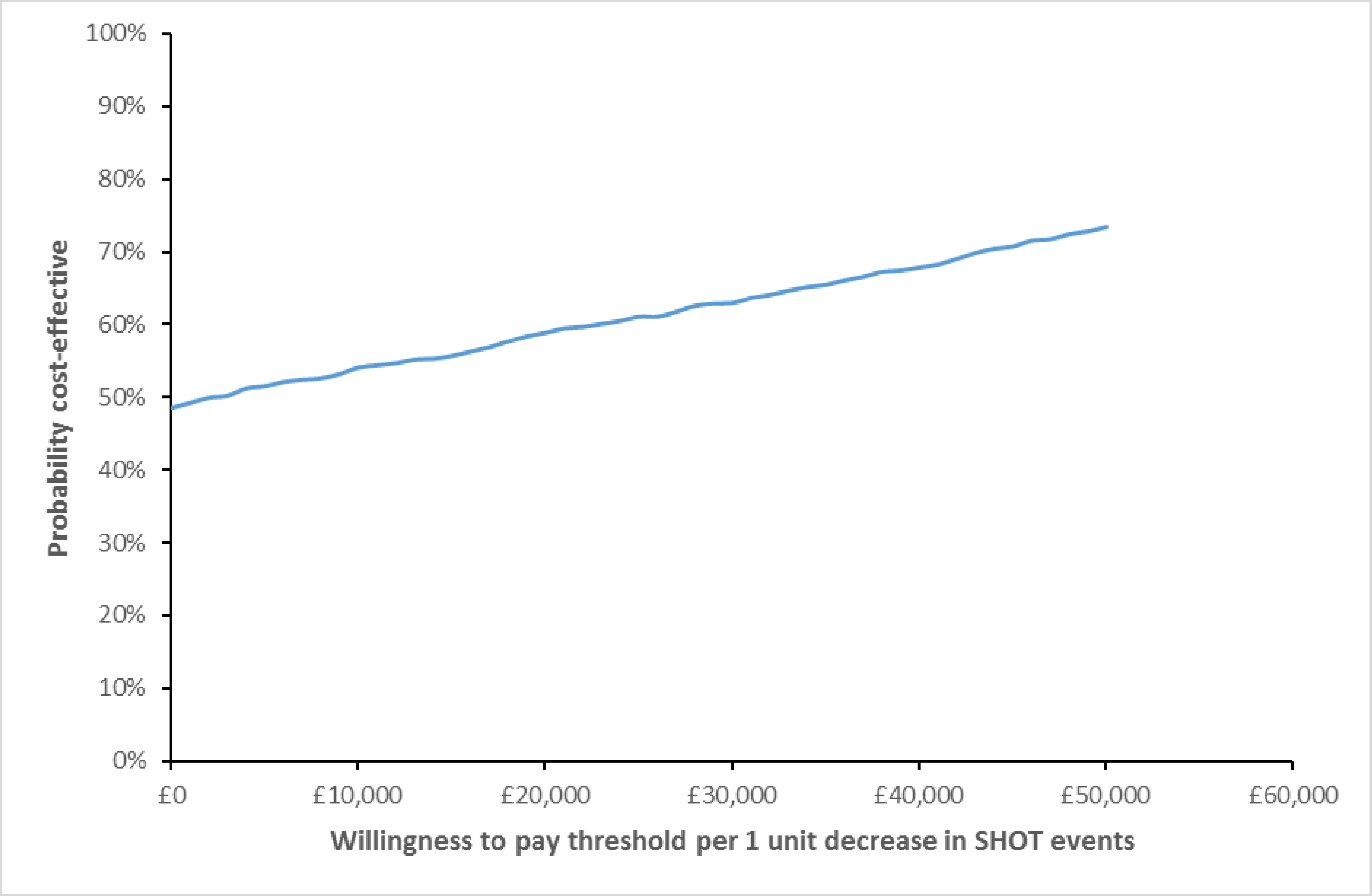

Figure 23: CEAC for Enhanced vs Standard Content for Trial 2 SHOT events

© 2022 Stanworth SJ et al. *JAMA Network Open*.

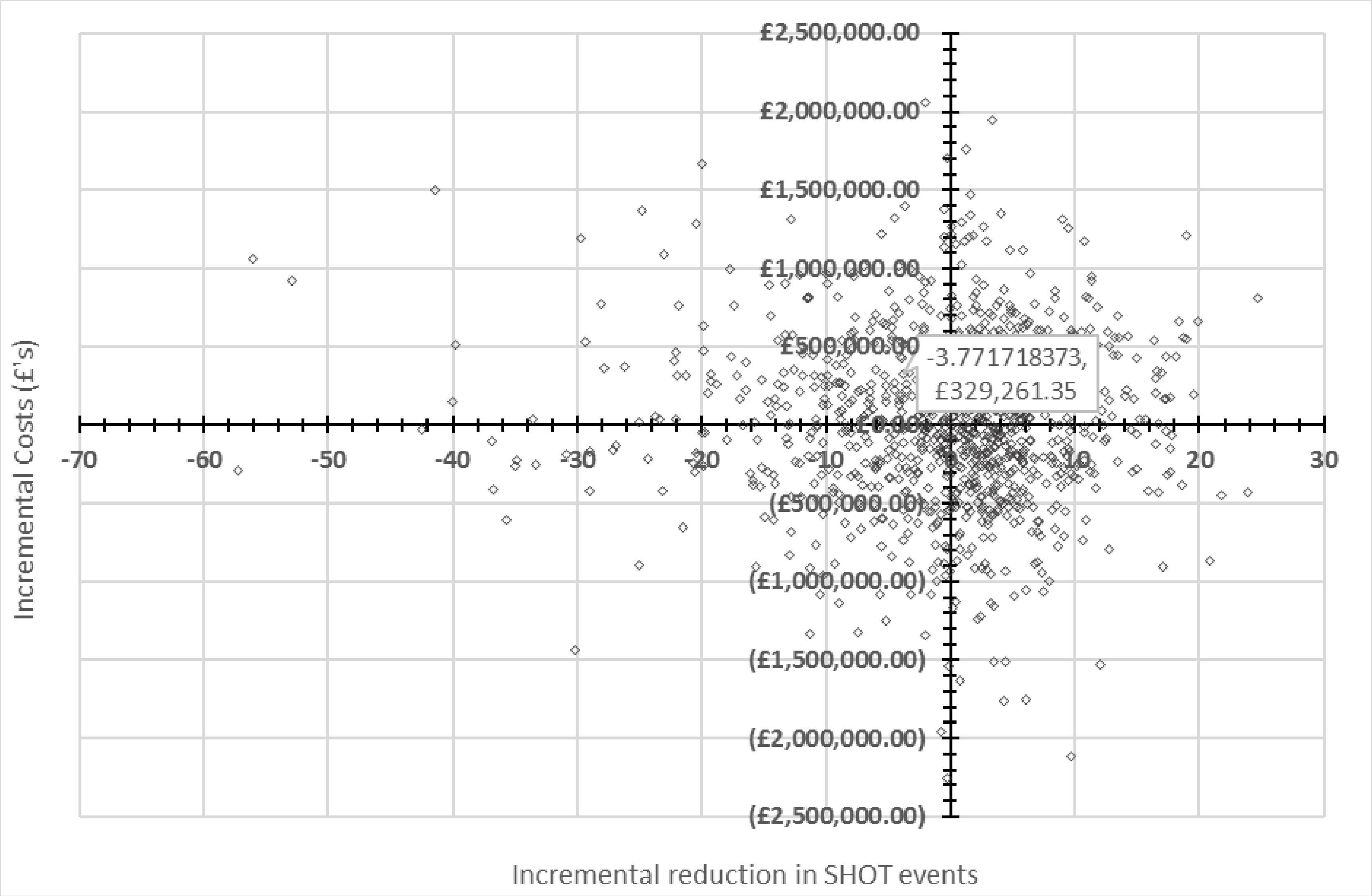

Figure 24: CEP for Enhanced vs Standard Follow-on Support for Trial 2 SHOT events

© 2022 Stanworth SJ et al. *JAMA Network Open*.

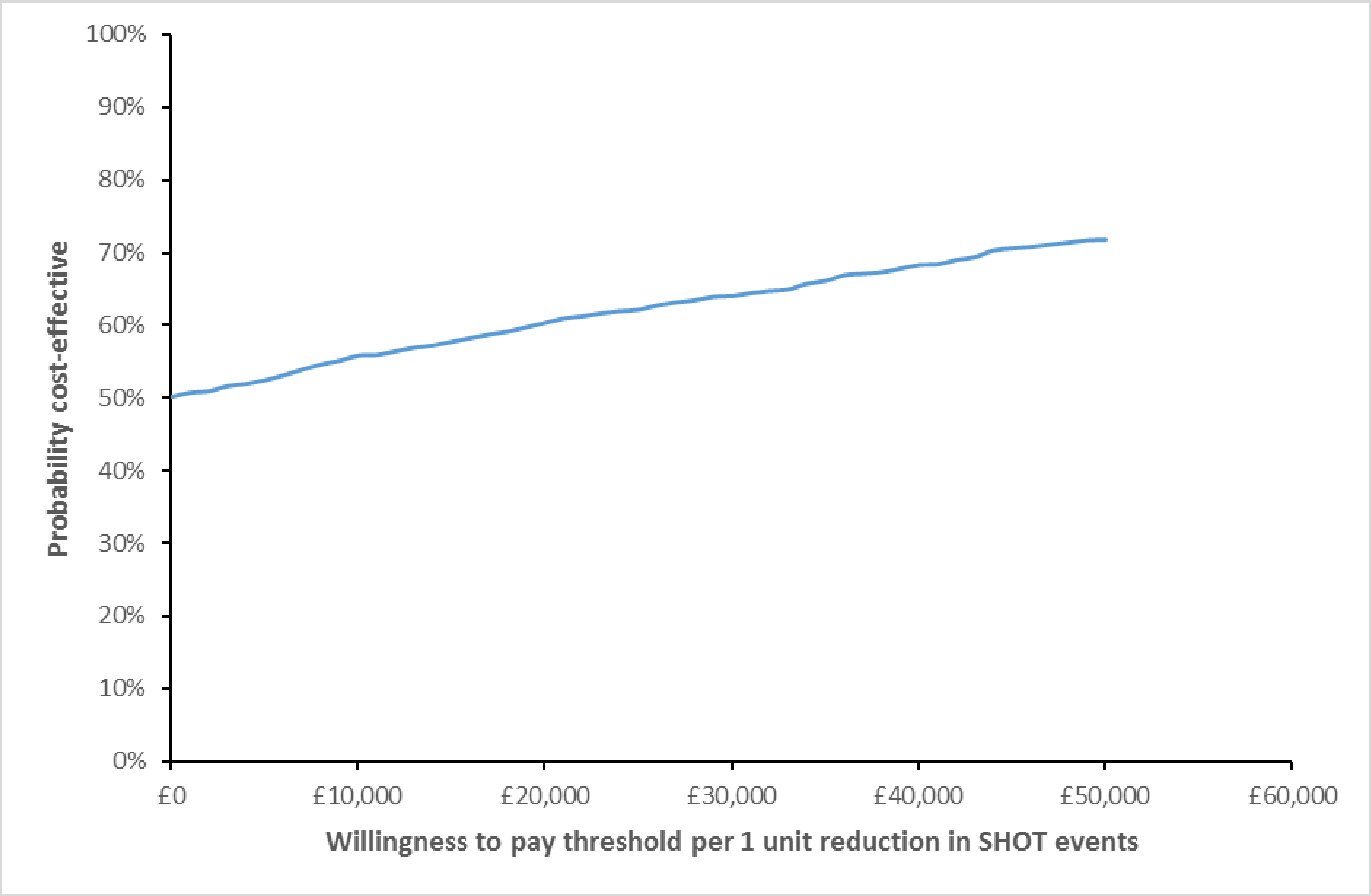

Figure 25: CEAC for Enhanced vs Standard Follow-on Support for Trial 2 SHOT events

© 2022 Stanworth SJ et al. *JAMA Network Open*.
