# Supplementary material for: Effectiveness of Enhanced Performance Feedback on Appropriate Use of Blood Transfusions: A Comparison of 2 Cluster Randomized Trials
Source: JAMA Netw Open. 2022 Feb 24;5(2):e220364. doi: 10.1001/jamanetworkopen.2022.0364 (PMC8874348; doi:10.1001/jamanetworkopen.2022.0364)
Supplement: Supplement 3. — Nonauthor Collaborators [file jamanetwopen-e220364-s003.pdf]

Supplemental Online Content: Nonauthor Collaborators

*Indicates required information. Only first name, last name, and suffix will appear in PubMed.

***Group Name(s): The AFFINITIE Collaborators**							
***First Name and Middle Initial(s)**	***Last Name**	***Suffix (eg, Jr, III)**	Academic Degrees	Institution	Location (city, state/province, country)	Role or Contribution, eg, chair, principal investigator	Group (if more than 1 Group listed in the byline) and/or Subgroup (eg, Steering Committee)
John	Bird			School of Health Sciences City, University of London; Faculty of Engineering, University of Bristol		Leading the development of the online toolkit for contribution	
Susan	Michie		DPhil	Division of Psychology and Language Sciences, University College London		Co-investigator on programme grant	
Liz	Glidewell		PhD	Hull York Medical School and Health Sciences, University of York		Co-investigator on programme grant	
Camilla	During		PhD	School of Health Sciences City, University of London		Intervention development	
Stephen	McIntyre		MSc	School of Health Sciences City, University of London		Intervention development	
Riya	Patel			Centre for Intelligent Healthcare, Coventry University		Intervention development	
James	Smith			School of Health Sciences City, University of London		Intervention development	
Alison	Deary		MSc	NHS Blood and Transplant		Trial Operations	
Michelle	Collinson		MSc	Clinical Trials Research Unit, Leeds Institute for Clinical Trials Research, University of Leeds		Statistical support	
Debbi	Poles			Serious Hazards of Transfusion (SHOT)		Outcome measuring	
Sue	Cotton			Blood Stocks Management Scheme (BSMS)		Outcome measuring	

Supplemental Online Content: Nonauthor Collaborators

*Indicates required information. Only first name, last name, and suffix will appear in PubMed.

***First Name and Middle Initial(s)**	***Last Name**	***Suffix (eg, Jr, III)**	Academic Degrees	Institution	Location (city, state/province, country)	Role or Contribution, eg, chair, principal investigator	Group (if more than 1 Group listed in the byline) and/or Subgroup (eg, Steering Committee)
Robert	Cicero		MSc	Clinical Trials Research Unit, Leeds Institute for Clinical Trials Research, University of Leeds		Statistical support	
Thomas	Smith		MSc	Clinical Trials Research Unit, Leeds Institute for Clinical Trials Research, University of Leeds		Statistical support	
Isabelle	Smith		MSc	Clinical Trials Research Unit, Leeds Institute for Clinical Trials Research, University of Leeds		Statistical support	
Ellen	Mason		MSc	Clinical Trials Research Unit, Leeds Institute for Clinical Trials Research, University of Leeds		Statistical support	
Zoe	Craig		MSc	Clinical Trials Research Unit, Leeds Institute for Clinical Trials Research, University of Leeds		Statistical support	
Karla	Diaz-Ordaz			Department of Medical Statistics, London School of Hygiene & Tropical Medicine		Statistical support	
