# Supplementary material for: Effectiveness of Enhanced Performance Feedback on Appropriate Use of Blood Transfusions: A Comparison of 2 Cluster Randomized Trials
Source: JAMA Netw Open. 2022 Feb 24;5(2):e220364. doi: 10.1001/jamanetworkopen.2022.0364 (PMC8874348; doi:10.1001/jamanetworkopen.2022.0364)
Supplement: Supplement 4. — Data Sharing Statement [file jamanetwopen-e220364-s004.pdf]

Data Sharing Statement

Stanworth. Effectiveness of Enhanced Performance Feedback on Appropriate Use of Blood Transfusions: A Comparison of 2 Cluster Randomized Trials. *JAMA Netw Open*. Published February 24, 2022. doi:10.1001/jamanetworkopen.2022.0364.

Data
Data available: Yes
Data types: Deidentified participant data
How to access data: Deidentified participant data will be made available on reasonable request to the first author, Simon J. Stanworth, at simon.stanworth@nhsbt.nhs.uk
When available: With publication

Supporting Documents
Document types: Statistical/analytic code
How to access documents: Statistical/analytic code will be made available on reasonable request to the first author, Simon J. Stanworth, at simon.stanworth@nhsbt.nhs.uk
When available: With publication

Additional Information
Who can access the data: Anyone upon reasonable request
Types of analyses: For any research purpose
Mechanisms of data availability: After approval of a proposal
